# Efficacy of multiple acupoint stimulation therapies for primary insomnia patients: a systematic review and network meta-analysis

**DOI:** 10.3389/fpsyt.2026.1678631

**Published:** 2026-04-13

**Authors:** Ying Wang, Hai-Lin Jiang, Jin-Ying Zhao, Ning-Ning Liu, Lin Yi, Ze-Rui Yang, Shu-Ming Zhao, Fu-Chun Wang

**Affiliations:** 1 School of Acupuncture-Moxibustion and Tuina, Changchun University of Chinese Medicine, Changchun, China; 2 Affiliated Hospital of Shandong University of Traditional Chinese Medicine, Jinan, China; 3 Graduate School, Beijing University of Chinese Medicine, Beijing, China; 4 Changchun University of Chinese Medicine, Changchun, China

**Keywords:** acupoint stimulation therapies, acupuncture points, individualized treatment, network meta-analysis, primary insomnia

## Abstract

**Background:**

Primary insomnia (PI) is a chronic sleep disorder with a complex pathogenesis, and various treatment options are available. In recent years, multiple acupoint stimulation therapies have gained increasing attention as non-pharmacological interventions. This systematic review and network meta-analysis aimed to assess the comparative efficacy of different acupoint stimulation therapies for PI.

**Methods:**

We searched eight Chinese and English databases and six trial registries from inception to September 22, 2024. Outcomes included total effective rate (TER), Pittsburgh Sleep Quality Index (PSQI) with its six subcomponents, and the change in PSQI scores (ΔPSQI) from baseline to post-treatment. A random-effects model was used for the network meta-analysis. Risk of bias and certainty of evidence were assessed using the ROB2 tool and CINeMA framework. The PROSPERO registration number is CRD42025640547.

**Results:**

A total of 95 RCTs involving 7,628 patients were included, comparing 14 acupoint stimulation therapies and involving 108 major acupoints. Body acupuncture combined with electroacupuncture ranked highest in improving the TER (SUCRA: 0.874), while moxibustion combined with tuina was most effective in reducing the total PSQI score (0.966). Moxibustion alone demonstrated the greatest improvement in the PSQI score difference (0.933). Electroacupuncture combined with auricular acupressure showed superior effectiveness in improving all six PSQI subcomponents. The most frequently used acupuncture points were Shenmen (HT 7, 62.1%), Baihui (GV 20, 56.3%), and Anmian (EX-HN 22, 45.8%), while the most commonly used ear points included Shenmen (TF_4_, 17.4%), Xin (CO_15_, 14.2%), and Pizhixia (AT_4_, 11.1%). Cluster analysis identified eight prevalent patterns of point compatibility.

**Conclusions:**

The tolerability and long-term efficacy of different acupoint stimulation therapies for primary insomnia remain unclear. These findings support individualized treatment and acupoint compatibility, laying the groundwork for optimizing intervention protocols and exploring neuroimmune mechanisms.

**Systematic Review Registration:**

https://www.crd.york.ac.uk/PROSPERO/view/CRD42025640547, identifier CRD42025640547.

## 1 Introduction

Primary insomnia (PI) is a chronic sleep disorder that occurs at least three times per week for a minimum of three months and is not associated with any known medical, psychiatric, or other sleep disorders (1). It is characterized by persistent difficulty in falling asleep, maintaining sleep, or frequently experiencing early morning awakenings, along with an inability to achieve restorative sleep despite having sufficient sleep opportunities (1). Approximately 10% of adults worldwide suffer from chronic insomnia, with PI estimated to account for 25% of these cases (2). Long-term insomnia may have widespread negative effects on individual health, including cognitive decline, memory impairment, emotional instability, and may also trigger anxiety, depression, and other mental disorders (3–5). Additionally, insomnia is closely associated with cardiovascular diseases, metabolic disorders, immune dysfunction, and chronic pain (6–9). Persistent sleep deprivation may impair neurological function, weakening decision-making ability and reaction speed, which not only diminishes an individual’s quality of life but also exacerbates physical and mental burdens (10, 11). Severe sleep disorders can reduce work efficiency and alertness, potentially leading to serious accidents and significant economic losses (12, 13). However, existing evidence on the effectiveness of pharmacological treatments is limited, and they are associated with risks such as side effects and dependency, with recommendations primarily for acute management of insomnia (14). The aims of treating insomnia are not only to alleviate symptoms but also to improve sleep quality, enhance daytime functioning, and reduce the mental burden on patients (15–18). In recent years, acupoint stimulation therapies, such as body acupuncture, moxibustion, and auricular acupressure, have gained increasing attention due to their significant efficacy and relatively low incidence of side effects, and have been widely applied in clinical practice (19–21). Nonetheless, comprehensive direct and indirect comparative studies on the relative efficacy of different acupoint stimulation therapies for PI, especially concerning sleep-related outcomes, remain scarce. Moreover, the therapeutic protocols included in existing studies are relatively limited. We employ network meta-analysis to evaluate the efficacy of various acupoint stimulation therapies, aiming to provide an objective evidence-based reference for clinical treatment plans for primary insomnia. This network meta-analysis will offer scientific evidence to guide the selection of individualized treatment protocols and acupoint combinations, benefiting clinicians, patients, and policymakers in making informed decisions regarding acupoint stimulation therapies for adult PI.

## 2 Materials and methods

We performed a systematic review and network meta-analysis of head-to-head randomized controlled trials (RCTs) in accordance with the Preferred Reporting Items for Systematic Reviews and Meta-Analyses (PRISMA) guidelines (22), and registered the review protocol with PROSPERO (registration number CRD42025640547) (**Appendix pp 1–4**). Ethical approval was not required for this study, as it is a systematic review and network meta-analysis of previously published data.

### 2.1 Search strategy and selection criteria

**Figure 1:**
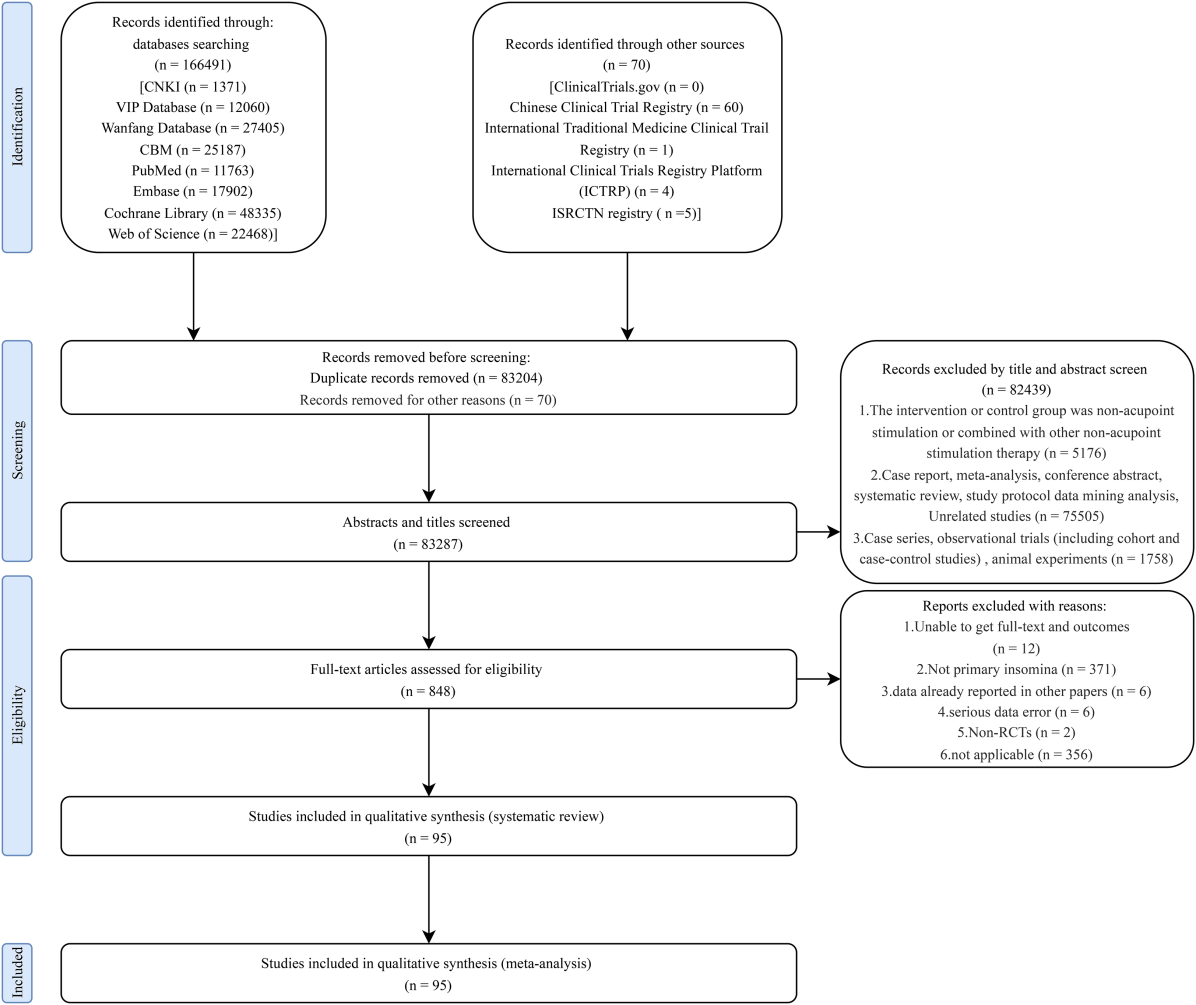
Flowchart of studies selection for inclusion in the meta.

We searched eight Chinese and English databases and six trial registries, with no language restriction, from inception to September 22, 2024. These details and search strategy, including search terms, can be found in the **Appendix (pp 5–8)**. We included RCTs in which participants (≥18 years) were diagnosed with PI based on standardized diagnostic criteria, including the Diagnostic and Statistical Manual of Mental Disorders (DSM-4, DSM-5), the International Classification of Diseases (ICD-10), the International Classification of Sleep Disorders (ICSD-3), the Chinese Classification of Mental Disorders (CCMD, CCMD-2, CCMD-2-R, CCMD-3, CCMD-3-R, CCMD-4), the Classification and Diagnostic Criteria of Diseases and Syndromes in Traditional Chinese Medicine (CDTE-TCM, 1994 edition), or other standardized criteria. We included all acupoint stimulation therapies recommended by international clinical insomnia guidelines (23, 24), and referred to various acupoint stimulation therapies reported in previous literature (**Appendix Table A.1**). Reported outcomes included at least one of the following: total effective rate (TER); Pittsburgh Sleep Quality Index (PSQI) and its six subcomponents–subjective sleep quality (SQ), sleep latency (SL), sleep duration (SD), sleep efficiency (SE), sleep disturbance index (SDI), and daytime dysfunction (DD); or change in PSQI scores (ΔPSQI). We only compared different acupoint stimulation protocols and excluded studies that involved the combined use of pharmacological treatments, traditional Chinese medicine, or other non-acupoint stimulation therapies. **Figure 1** illustrates the literature search, screening protocol and selection.

### 2.2 Study selection and data extraction

At least two reviewers (HLJ, JYZ, NNL and LY) independently screened the search results. Two reviewers independently extracted the data (HLJ, JYZ, NNL, LY and ZRY), and any discrepancies were discussed with a third reviewer (YW, SMZ or FCW) to resolve the issues. A standardized spreadsheet was used to extract study characteristics, participant characteristics and outcomes from the included literature (**Appendix Tables A.2–A.3**). The efficacy outcomes included TER (%), PSQI, SQ, SL, SD, SE, SDI, DD and ΔPSQI (25). If any information was missing or unclear, we contacted the authors of the original study by phone or email.

### 2.3 Statistical analysis

We conducted a network meta-analysis using a random-effects model within the frequentist framework and assessed statistical heterogeneity (26, 27). For continuous outcomes, we used mean difference (MD) as the effect measure, while for dichotomous outcomes, relative risk (RR) was used, with 95% confidence intervals (CIs) calculated. To ensure consistency, we performed global, local, and loop-specific inconsistency tests (28, 29). To identify potential sources of heterogeneity, we conducted a sensitivity analysis by removing individual studies. We used comparison-adjusted funnel plots to assess publication bias, and linear regression was applied to test for funnel plot asymmetry (30). The risk of bias in the included studies was assessed using the Cochrane Risk of Bias 2 (ROB 2) tool (31, 32). The quality of evidence was graded according to the Confidence in Network Meta-Analysis (CINeMA) framework (33, 34). Rankogram probability plots were used to visualize the distribution of probabilities for each treatment ranking, and the surface under the cumulativing ranking curve (SUCRA) was used to rank the treatments (35). All statistical analyses were performed using R (version 4.4.3) and Stata (version 17.0). Raw data and detailed data analysis methods can be found in the **Supplementary Material Table 1** and **Appendix (pp 9–10)**.

## 3 Results

### 3.1 Study selection and quality assessment

We identified 166,491 records from eight databases and additionally retrieved 70 relevant articles through manual searches of clinical trial registries. After merging all search results, duplicates and studies that did not meet the inclusion criteria were removed. A total of 848 studies underwent full-text evaluation, with 95 studies included in the analysis. The study selection and inclusion process is shown in **Figure 1**. The bias risk assessment for the RCTs is presented in the (**Appendix Figure A.1**). Overall, 32 studies (33.7%) were classified as having a high risk of bias, while 63 studies (66.3%) demonstrated some concerns regarding bias (**Appendix Table A.4**).

### 3.2 Study characteristics

**Figure 2:**
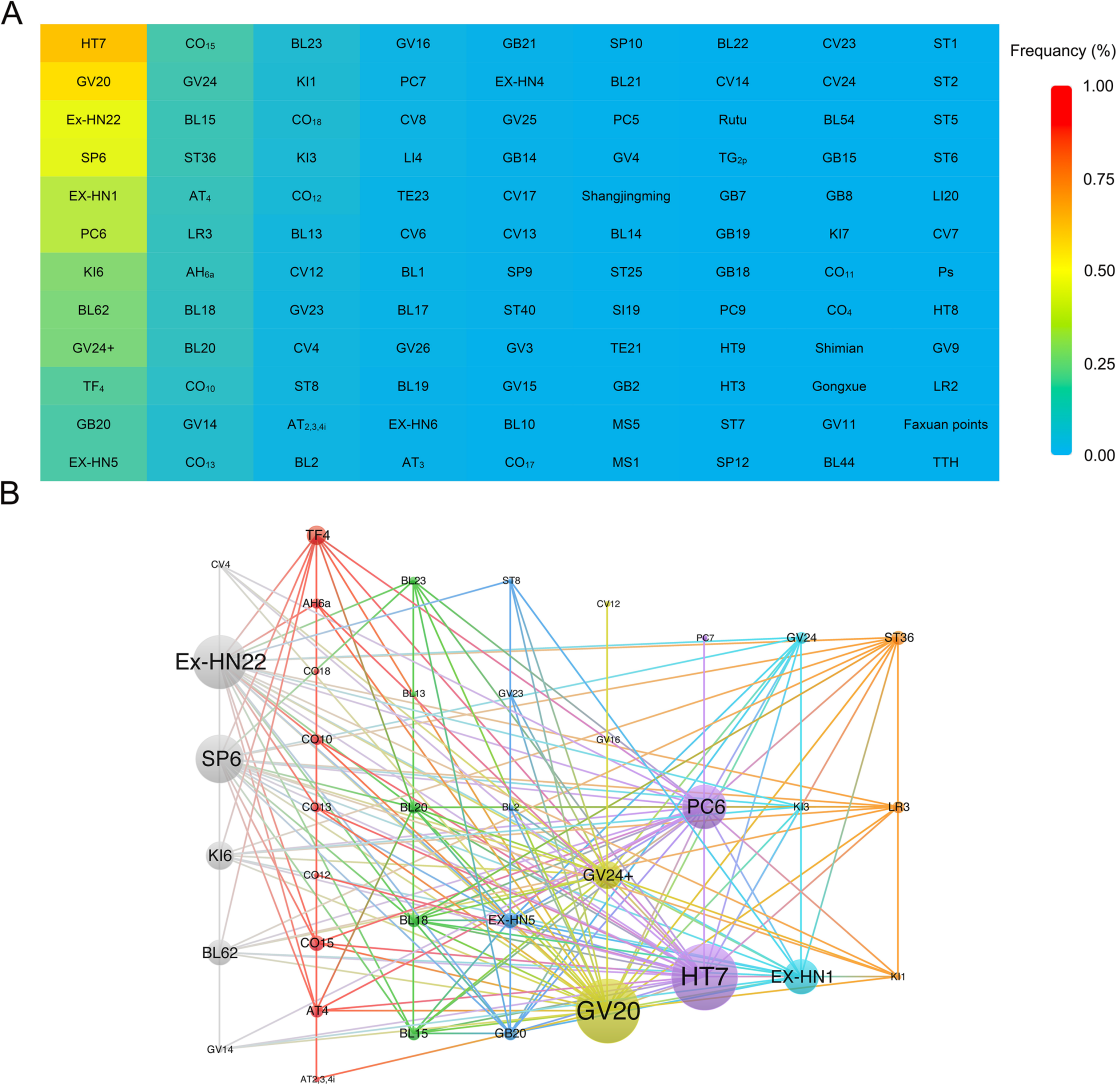
Frequency ranking chart of acupoints and cluster analysis of acupoint compatibility. **(A)** This chart presents the frequency of each acupoint across all treatment protocols. The colors from red to blue mean the frequency from large to small. **(B)** Nodes represent individual acupoints, with node size reflecting the frequency of each acupoint’s use in the treatment protocols. The thickness of the connecting lines indicates the strength of compatibility between acupoints. The network illustrates the relationships among acupoints, with cluster analysis categorizing acupoints into distinct groups. Acupoints sharing the same color belong to the same cluster, indicating their potential synergistic effects in the treatment of insomnia.

A total of 7,628 patients with primary insomnia were randomly assigned to intervention and control groups, with 3,829 and 3,799 participants, respectively. Ninety-one articles (95.8%) reported the gender of the participants, with 3,267 (42.8%) male patients. Fifteen studies (15.8%) classified patients with insomnia according to Traditional Chinese Medicine (TCM) syndromes, involving 16 different patterns. A total of 108 major acupoints were included across 95 studies. These acupoints were distributed across 11 meridians: the Large Intestine Meridian of Hand-Yangming (2), Stomach Meridian of Foot-Yangming (9), Spleen Meridian of Foot-Taiyin (4), Heart Meridian of Hand-Shaoyin (4), Small Intestine Meridian of Hand-Taiyang (1), Bladder Meridian of Foot-Taiyang (16), Kidney Meridian of Foot-Shaoyin (4), Pericardium Meridian of Hand-Jueyin (4), Sanjiao Meridian of Hand-Shaoyang (2), Gallbladder Meridian of Foot-Shaoyang (9), Liver Meridian of Foot-Jueyin (2), Du Meridian (13), and Ren Meridian (10), covering a total of 80 meridian points. In addition, 13 extra points and 15 ear points were identified (**Table 1**). The top 5 most frequently used acupoints were Shenmen (HT 7, 62.1%), Baihui (GV 20, 56.3%), Anmian (Ex-HN 22, 45.8%), Sanyinjiao (SP 6, 44.7%), and Sishencong (EX-HN 1, 36.3%), with Neiguan (PC 6, 36.3%) tied in frequency (**Figure 2A**). Ear acupoints such as Shenmen (TF_4_, 17.4%), Xin (CO_15_, 14.2%), and Pizhixia (AT_4_, 11.1%) also showed high usage rates (**Figure 2A**). **Figure 2B** revealed the cluster analysis of acupoint compatibility. Eighty-five studies (89.5%) reported the duration of illness in patients with PI, while 16 studies (16.8%) provided follow-up data. In 5 studies (5.3%) involving 371 patients, changes in inflammatory markers and neurotransmitter levels were observed, including IL-1β (Interleukin-1β), TNF-α (Tumor Necrosis Factor-α), GABA (Gamma-Aminobutyric Acid), IL-6 (Interleukin-6), CRP (C-reactive protein), and Glu (Glutamate). A total of 17 studies (17.9%) involving 1,575 patients reported 44 adverse events (AEs) (2.8%) related to treatment. **Table 1** and **Appendix Tables A.2–A.3** summarize the clinical and methodological characteristics of each study, along with the outcomes. Standard acupuncture point locations are provided in the (**Appendix Table A.5**).

**Table 1:** Classification and meridian attribution of acupoints.

Meridians and classification	Frequency of acupoints		Number of acupoints		Acupuncture points (Frequency)
	Frequency	Rate (%)	Numbers	Rate (%)	
The lung meridian of hand-Taiyin	0	0	0	0	NA
The large intestine meridian of hand-Yangming	6	0.47	2	1.85	LI4 (5), LI20 (1)
The stomach meridian of foot-Yangming	60	4.69	9	8.33	ST1 (1), ST2 (1), ST5 (1), ST6 (1), ST7 (1), ST8 (7), ST25 (2), ST36 (23), ST40 (3)
The spleen meridian of foot-Taiyin	91	7.11	4	3.70	SP6 (85), SP9 (3), SP10 (2), SP12 (1)
The heart meridian of hand-Shaoyin	121	9.46	4	3.70	HT3 (1), HT7 (118), HT8 (1), HT9 (1)
The small intestine meridian of hand-Taiyang	2	0.16	1	0.93	SI19 (2)
The bladder meridian of foot-Taiyang	155	12.12	16	14.81	BL1 (4), BL2 (6), BL10 (2), BL13 (8), BL14 (2), BL15 (23), BL17 (4), BL18 (19), BL19 (3), BL20 (19), BL21 (2), BL22 (1), BL23 (13), BL44 (1), BL54 (1), BL62 (47)
The kidney meridian of foot-Shaoyin	74	5.79	4	3.70	KI1 (12), KI3 (11), KI6 (50), KI7 (1)
The pericardium meridian of hand-Jueyin	77	6.02	4	3.70	PC5 (2), PC6 (69), PC7 (5), PC9 (1)
The Sanjiao meridian of hand-Shaoyang	6	0.47	2	1.85	TE21 (2), TE23 (4)
The gallbladder meridian of foot-Shaoyang	42	3.28	9	8.33	GB2 (2), GB7 (1), GB8 (1), GB14 (3), GB15 (1), GB18 (1), GB19 (1), GB20 (29), GB21 (3)
The liver meridian of foot-Jueyin	21	1.64	2	1.85	LR2 (1), LR3 (20)
The Du meridian	219	17.12	13	12.04	GV3 (2), GV4 (2), GV9 (1), GV11 (1), GV14 (14), GV15 (2), GV16 (5), GV20 (107), GV23 (7), GV24 (25), GV24+ (46), GV25 (3), GV26 (4)
The Ren meridian	34	2.66	10	9.26	CV4 (7), CV6 (4), CV7 (1), CV8 (5), CV12 (8), CV13 (3), CV14 (1), CV17 (3), CV23 (1), CV24 (1)
Extra points and Empirical acupoints	202	15.79	13	12.04	EX-HN1 (69), EX-HN4 (3), EX-HN5 (29), EX-HN6 (3), Ex-HN22 (87), MS1 (2), MS5 (2), Shangjingming (2), Rutu(1), Gongxue(1), TTH (1), Faxuan points (1)
Ear points	169	13.21	15	13.89	TF_4_ (33), AT_3_ (3), AT_2,3,4i_ (6), AT_4_ (21), AH_6a_ (20), CO_4_ (1), CO_10_ (17), CO_11_ (1), CO_12_ (10), CO_13_ (14), CO_15_ (27), CO_17_ (2), CO_18_ (12), TG_2p_ (1), Ps (1), Shimian (1)

Acupoints were classified into categories including those on the eleven meridians, eight extraordinary meridians, extra points, empirical acupoints, and ear points. Acupuncture points were assigned to its corresponding meridian system. The frequency of each acupoint’s usage was calculated.

### 3.3 Network meta-analysis

In our network meta-analysis, all outcomes exhibited high heterogeneity (I² > 50%). The results revealed significant inconsistency (P < 0.05) in both global and local inconsistency tests for SQ, SL, and SE. In the loop inconsistency test, SQ, SL, SD, SDI, and DD showed substantial inconsistency, suggesting significant uncertainty in these comparisons. For certain treatment comparisons, the confidence intervals did not cross 0 or 1, indicating potential statistical significance. The comparison-adjusted funnel plots did not show significant publication bias. Sensitivity analysis results indicated that excluding any individual study did not notably affect the heterogeneity of the outcomes, suggesting that potential sources of heterogeneity remain between studies. The CINEMA assessment downgraded much of the evidence to very low quality due to within-study bias and imprecision. Detailed results are available in the **Appendix Tables A.6–A.9** and **Figure A.2–A.52** and **Supplementary Material Table 2**.

### 3.4 Total effective rate

**Figure 3:**
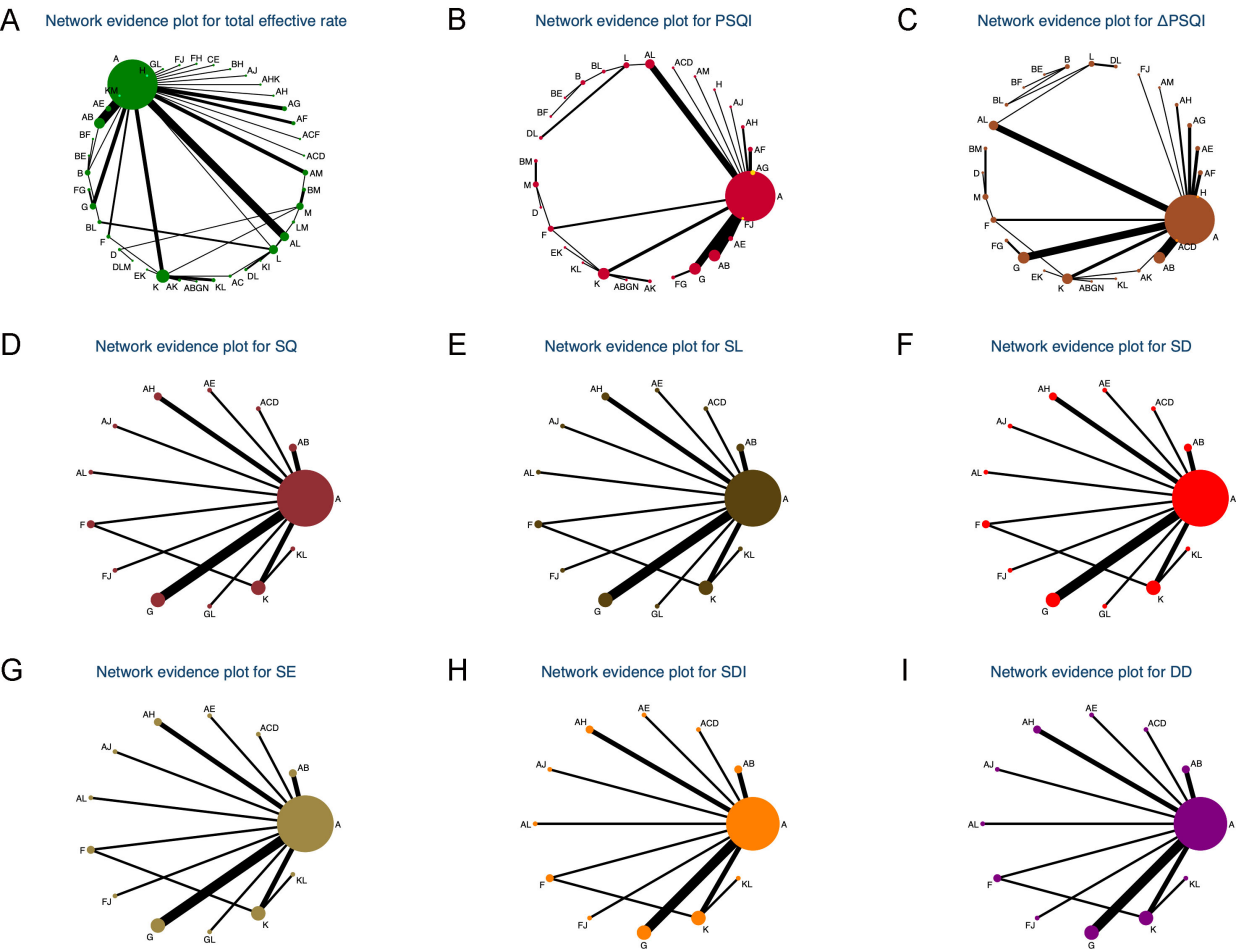
Network plots of multiple acupoint stimulation therapies, either as monotherapies or in combination. **(A–I)** Network plots for TER, PSQI, ΔPSQI, SQ, SL, SD, SE, SDI, and DD. The size of each node represents the total sample size for that treatment. Lines between nodes indicate direct comparisons; the width of the lines reflects the number of trials informing each comparison. **(A)** body acupuncture; **(B)** moxibustion; **(C)** cupping therapy; **(D)** guasha therapy; **(E)** bloodletting therapy; **(F)** specialized acupuncture needles; **(G)** specialized acupuncture point therapies; **(H)** scalp acupuncture; **(I)** ear acupuncture; **(J)** eye acupuncture; **(K)** electroacupuncture; **(L)** auricular acupressure; **(M)** tuina; **(N)** cerebral circulation electrical stimulation.

**Figure 4:**
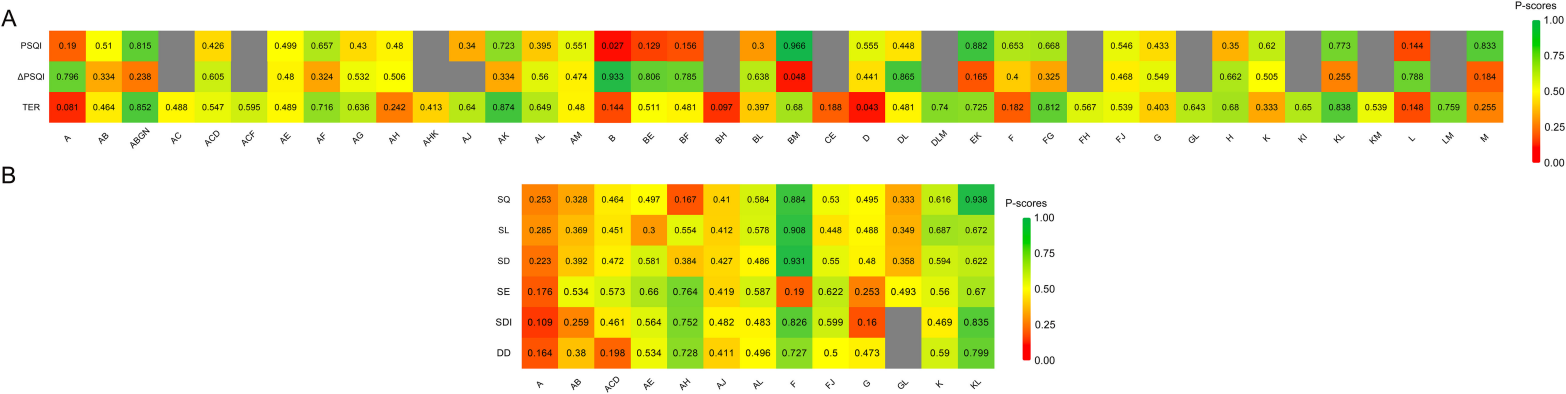
**(A, B)** The rankings of included acupoint stimulation therapies for different efficacy outcomes based on SURCA. The colors from green to red mean the SUCRA values from large to small, and grey means no data. TER, total effective rate; PSQI, Pittsburgh Sleep Quality Index; ΔPSQI, change in PSQI scores (calculated by subtracting the post-treatment score from the baseline score); SQ, subjective sleep quality; SL, sleep latency; SD, sleep duration; SE, sleep efficiency; SDI, sleep disturbance index; DD, daytime dysfunction. A, body acupuncture; B, moxibustion; C, cupping therapy; D, guasha therapy; E, bloodletting therapy; F, specialized acupuncture needles; G specialized acupuncture point therapies; H, scalp acupuncture; I, ear acupuncture; J, eye acupuncture; K, electroacupuncture; L, auricular acupressure; M, tuina; N, cerebral circulation electrical stimulation.

A total of 89 studies involving 7,261 patients were included, with 14 treatment modalities applied independently or in combination, forming 40 treatment regimens (**Figure 3A**). SUCRA analysis ranked body acupuncture combined with electroacupuncture (0.874) as the most effective approach for improving clinical efficacy, followed by body acupuncture combined with moxibustion, specialized acupuncture point therapies, and cerebral circulation electrical stimulation (0.852) (**Figure 4A**). Electroacupuncture combined with auricular acupressure (0.838) ranked third, while specialized acupuncture needles combined with specialized acupuncture point therapies (0.812) also showed notable efficacy (**Figure 4A**). Conversely, moxibustion combined with scalp acupuncture (0.097), body acupuncture alone (0.081), and guasha therapy (0.043) demonstrated limited effectiveness (**Figure 4A**). No significant differences were observed among the four most effective or the three least effective regimens (**Appendix Table A.10**).

### 3.5 PSQI and its subcomponents

#### 3.5.1 PSQI

62 studies involving 5,275 patients evaluated the efficacy of 13 distinct treatment strategies, either individually or in combination, resulting in 29 treatment regimens (**Figure 3B**). SUCRA analysis indicated that moxibustion combined with tuina (0.966) was the most effective approach for reducing PSQI, followed by bloodletting therapy combined with electroacupuncture (0.882) and tuina alone (0.833) (**Figure 4A**). In contrast, moxibustion alone (0.027) demonstrated limited effectiveness in improving PSQI (**Figure 4A**). Moxibustion combined with tuina significantly reduced PSQI compared to tuina alone (2.86 [0.32, 5.41]) (**Appendix Table A.11**). Compared with moxibustion alone, moxibustion combined with tuina (13.10 [5.16, 21.05]), bloodletting therapy combined with electroacupuncture (10.84 [3.55, 18.14]), and tuina alone (10.24 [2.71, 17.77]) all showed a significant reduction in PSQI (**Appendix Table A.11**). However, when compared with bloodletting therapy combined with electroacupuncture, neither moxibustion combined with tuina (2.26 [-4.00, 8.52]) nor tuina alone (0.60 [-5.12, 6.33]) demonstrated a statistically significant difference in reducing PSQI (**Appendix Table A.11**).

#### 3.5.2 SQ、SL、SD and SE

Across 19 studies involving 1,331 patients, 11 treatment strategies were applied individually or in combination, forming 13 distinct regimens (**Figures 3D–G**). SUCRA values indicated that electroacupuncture combined with auricular acupressure was the most effective approach for reducing SQ (0.938) and SD (0.622), followed by electroacupuncture alone (SQ: 0.616, SD: 0.594) (**Figure 4B**). For SL, specialized acupuncture needles exhibited the greatest potential in shortening sleep latency (0.908), with electroacupuncture (0.687) and electroacupuncture combined with auricular acupressure (0.672) ranking next (**Figure 4B**). Regarding SE, body acupuncture combined with scalp acupuncture (0.764) was the most effective regimen, followed by electroacupuncture combined with auricular acupressure (0.670) (**Figure 4B**). Conversely, body acupuncture alone demonstrated limited efficacy in reducing SQ (0.249), SL (0.285), SD (0.223), and SE (0.176), as shown in **Figure 4B**. No significant differences were observed among electroacupuncture combined with auricular acupressure, specialized acupuncture needles, and electroacupuncture alone in SQ, SL, SD, or SE (**Appendix Tables A.13–A.16**). However, compared to body acupuncture, electroacupuncture combined with auricular acupressure (1.72 [0.39, 3.06]) and specialized acupuncture needles (1.33 [0.46, 2.20]) resulted in significantly greater reductions in SQ scores (**Appendix Table A.13**). Specialized acupuncture needles also outperformed body acupuncture in reducing SL (1.70 [0.43, 2.98]) and SD (1.21 [0.41, 2.01]) (**Appendix Tables A.14–A.15**). Additionally, body acupuncture combined with scalp acupuncture significantly reduced SE compared to body acupuncture alone (0.55 [0.10, 0.99]) (**Appendix Table A.16**).

#### 3.5.3 SDI and DD

Total of 18 studies involving 1,291 patients were analyzed, incorporating 11 treatment strategies, either independently or in combination, resulting in 12 treatment regimens (**Figures 3H–I**). SUCRA values indicated that electroacupuncture combined with auricular acupressure was the most effective approach for improving SDI (0.835) and DD (0.799), while body acupuncture alone showed limited efficacy in SDI (0.109) and DD (0.164) (**Figure 4B**). Compared to body acupuncture, electroacupuncture combined with auricular acupressure significantly reduced SDI (0.80 [0.09, 1.51]) (**Appendix Table A.17**). However, for DD, electroacupuncture combined with auricular acupressure did not show a significant advantage over body acupuncture alone (0.84 [-0.14, 1.82]) (**Appendix Table A.18**). Notably, body acupuncture combined with scalp acupuncture (0.63 [0.07, 1.19]) and specialized acupuncture needles (0.64 [0.03, 1.26]) resulted in significantly greater improvements in DD than body acupuncture alone (**Appendix Table A.18**).

### 3.6 Change in PSQI scores

In 61 studies with 5,175 patients, 13 treatment methods were applied either independently or in combination, forming 28 treatment strategies (**Figure 3C**). SUCRA results indicated that among these regimens, moxibustion (0.933) was likely the most effective in improving the PSQI difference, followed by guasha therapy combined with auricular acupressure (0.865) and moxibustion combined with bloodletting therapy (0.806) (**Figure 4A**). In contrast, moxibustion combined with tuina (0.048) showed a relatively limited effect (**Figure 4A**). Compared to moxibustion combined with tuina, moxibustion (12.92 [3.43, 22.41]), guasha therapy combined with auricular acupressure (10.64 [2.65, 18.63]), and moxibustion combined with bloodletting therapy (10.91 [0.59, 21.23]) all contributed to significant improvements in PSQI difference (**Appendix Table A.12**). However, no significant differences were observed among moxibustion, guasha therapy combined with auricular acupressure, and moxibustion combined with bloodletting therapy in enhancing PSQI difference (**Appendix Table A.12**).

## 4 Discussion

This study systematically evaluated the efficacy of 14 different acupoint stimulation therapies, either as standalone treatments or in combination, for PI. Based on 95 randomized controlled trials involving 7,628 patients, we conducted a comprehensive comparison of these interventions across multiple sleep-related outcomes. The SUCRA rankings revealed variations in treatment efficacy across different sleep outcomes, which may be attributed to the number of included studies, variations in outcome assessment methods, and study heterogeneity. SUCRA reflects the probabilistic ranking of relative effectiveness rather than absolute clinical significance. Therefore, its interpretation should be integrated with effect sizes and patient characteristics. Additionally, the number of included studies and treatment composition may influence the stability and representativeness of SUCRA results. In the analysis of TER, 89 studies provided relevant data, while PSQI were reported in only 62 studies. Consequently, the SUCRA results for effectiveness are relatively more representative, whereas PSQI rankings may be more susceptible to fluctuations due to limited sample size. It is important to note that TER is often based on physicians’ subjective assessments of symptom improvement, making it susceptible to variations in study design and evaluation criteria. In contrast, PSQI and its six subdomains are patient-reported outcomes that systematically assess sleep quality from multiple dimensions. While PSQI and effectiveness outcomes are correlated, their rankings are not always entirely consistent.

When considering SUCRA rankings comprehensively, body acupuncture combined with electroacupuncture; body acupuncture combined with moxibustion, specialized acupuncture point therapies, and cerebral circulation electrical stimulation; and electroacupuncture combined with auricular acupressure consistently demonstrated superior performance in both TER and PSQI. These findings suggest that these interventions may serve as promising strategies for insomnia management. PSQI major reflects sleep quality, whereas the ΔPSQI emphasizes the magnitude of improvement, potentially offering a more nuanced evaluation of treatment efficacy. Although the network structures for PSQI and ΔPSQI are similar, differences in SUCRA rankings persisted due to variations in calculation methods, highlighting the complementary nature of these two measures in clinical practice.

Since PSQI is a composite score, its subdomains capture different aspects of sleep. Some interventions may significantly improve specific subdomains without substantially impacting the overall PSQI score. Notably, only 19 studies reported SQ, SD, SL, and SE data, while 18 studies provided SDI and DD data, resulting in relatively small sample sizes. As a result, SUCRA rankings for these subdomains may be more susceptible to individual study effects, leading to lower stability. Despite differences in SUCRA rankings across different outcomes, certain therapies demonstrated consistent superiority. For instance, electroacupuncture combined with auricular acupressure; specialized acupuncture needles; and body acupuncture combined with scalp acupuncture exhibited strong performance across multiple PSQI subdomains, indicating their potential as effective interventions for primary insomnia. Among these, electroacupuncture combined with auricular acupressure ranked highly in effectiveness (83.8%), PSQI (77.3%), SQ (93.5%), SL (67.2%), DD (79.9%), and SDI (83.5%), suggesting it as a preferred treatment option for PI. The specialized acupuncture needles demonstrated notable efficacy in improving SQ (88.2%), SL (90.8%), and SDI (82.4%), making it potentially more suitable for patients with difficulty initiating sleep and poor subjective sleep quality. Meanwhile, the body acupuncture combined with scalp acupuncture ranked highly in SE (76.4%), DD (72.8%), and SDI (74.9%), indicating its potential benefits in enhancing daytime functioning and sleep structure.

The heterogeneity of study populations may also contribute to differences in SUCRA rankings. As shown in **Appendix Table A6**, the I² statistics indicate substantial heterogeneity for several outcomes, ranging from 68.4% for sleep efficiency to 97.7% for PSQI. Variability in TCM syndromes, insomnia severity, age, and gender across studies may lead to differential effects of the same intervention across different populations. Most studies reported only short-term outcomes, with limited follow-up data, which may underestimate the long-term efficacy of some interventions and increase uncertainty regarding sustained effects. Intervention heterogeneity is another critical consideration. Despite all therapies being categorized as acupoint stimulation, substantial differences were observed in acupoint selection, stimulation methods, treatment frequency, and course.

Besides population and intervention variability, mechanistic and safety considerations also provide insights into treatment effects. Five studies (5.3%) reported biomarker changes in 371 patients with PI, mainly involving pro-inflammatory cytokines (IL-1β, TNF-α, IL-6, CRP) and neurotransmitters (GABA, Glu). These molecular markers may form a neuroinflammatory network implicated in the pathophysiology of insomnia. Chronic inflammation, characterized by elevated IL-1β, TNF-α, and IL-6, may disrupt the sleep-wake cycle through activation of the hypothalamic-pituitary-adrenal (HPA) axis, while increased CRP levels have been associated with impaired sleep continuity (8, 36). GABAergic dysfunction may lead to insufficient central inhibition, and excessive glutamatergic activation of the prefrontal-amygdala circuit can contribute to hyperarousal (37, 38). The interplay of these inflammatory mediators and neurotransmitter imbalances likely contributes to difficulties in sleep initiation and maintenance. Previous studies suggest that acupoint stimulation therapies may modulate these biomarkers to improve sleep quality (39–43). However, due to the limited number of studies reporting these biomarkers and potential heterogeneity in measurement methods and time points, further research is needed to validate these findings and identify potential therapeutic targets. Among the included studies, 17 (17.9%) reported AEs in 1,575 patients, with 44 cases (2.8%) of AEs documented. The types of AEs varied considerably, ranging from minor discomforts such as needling pain or needle shock to more serious events including burns or local infections. Furthermore, factors such as treatment protocols, stimulation intensity, and individual tolerance levels may influence the incidence of AEs, warranting further investigation in future research.

## 5 Strengths

This network meta-analysis demonstrates a high level of systematic rigor and comprehensiveness. We conducted an extensive search across 8 Chinese and English databases and 6 trial registries, initially identifying over 160,000 articles. Ultimately, 95 eligible RCTs were included, covering 14 protocols, providing a solid and robust evidence base for clinical practice. To enhance the homogeneity and specificity of the study, only research involving acupoint stimulation for the treatment of primary insomnia was included, excluding studies combining pharmacological treatments, traditional Chinese medicines, or other non-acupoint stimulation therapies, ensuring the specificity and clinical applicability of the conclusions.

In our acupuncture point analysis, we created a heatmap to visualize the frequency of acupoint usage and conducted clustering and sorting analyses based on co-occurrence frequency, systematically summarizing the patterns of point combinations in the treatment of primary insomnia. The results revealed that 11 meridians were involved, covering 108 acupuncture and ear points. The most frequently used acupuncture points were Shenmen (HT 7, 62.1%), Baihui (GV 20, 56.3%), Anmian (Ex-HN 22, 45.8%), Sanyinjiao (SP 6, 44.7%), Sishencong (Ex-HN 1, 36.3%), and Neiguan (PC 6, 36.3%). High-frequency acupuncture points were primarily located on the Du Meridian, the The bladder meridian of foot-Taiyang, and The heart meridian of hand-Shaoyin, indicating their central role in insomnia treatment. Moreover, this study underscores the importance of ear points in treating primary insomnia, with frequently used points such as Shenmen (TF_4_, 17.4%), Xin (CO_15_, 14.2%), and Pizhixia (AT_4_, 11.1%), highlighting the potential value of ear points therapy.

We further identified 38 core acupuncture (29) and ear point (9) combinations that co-occurred ≥5 times and summarized 8 common clinical combination patterns through clustering analysis. Among these, Shenmen (HT 7) was primarily combined with Neiguan (PC 6) and Daling (PC 7); Baihui (GV 20) was often paired with Yintang (GV 24+) and Fengfu (GV 16); Anmian (Ex-HN 22) was frequently combined with Sanyinjiao (SP6), Zhaohai (KI 6), and Shenmai (BL 62); and Sishencong (Ex-HN 1) was mainly used with Taixi (KI 3) and Shenting (GV 24). These combinations, according to TCM theory, help harmonize yin and yang and calm the spirit, thereby improving sleep quality.

Modern research also provides biological support for acupuncture point stimulation in treating insomnia, revealing its multi-pathway and multi-target mechanisms. Acupoint stimulation can improve sleep quality and alleviate insomnia symptoms by regulating neurobiological pathways, modulating inflammatory factors, adjusting neurotransmitters, and inhibiting apoptosis (40). Animal experiments have demonstrated that stimulation of Shenmen (HT 7) and Sanyinjiao (SP 6) can regulate the sympathetic-adrenal medullary system to exert sedative and calming effects (44). Additionally, stimulation of Baihui (GV 20) can regulate signaling pathways in the hypothalamus, temporal lobe, and frontal lobe, improving insomnia and related emotional issues (45).

Regarding outcomes, we not only analyzed total effective rate, PSQI, and the ΔPSQI, but also further explored the six subdomains of PSQI. Using SUCRA rankings, we compared the advantages of different protocols in various sleep dimensions, providing valuable insights for the selection of individualized treatment strategies in clinical practice. In terms of methodological quality control, we systematically assessed the risk of bias using the ROB2 tool and evaluated the quality of evidence using CINeMA, thereby enhancing the scientific rigor and credibility of the findings. Additionally, this study attempted to integrate changes in inflammatory factors and neurotransmitters as objective indicators from some of the included studies, exploring the potential mechanisms of acupuncture point stimulation and providing ideas and directions for future mechanistic research.

## 6 Limitations

Despite the comprehensive evaluation conducted in this study, several limitations should be considered when interpreting the findings. Methodological issues in some of the included trials may reduce confidence in the results. Several earlier studies provided limited information on randomization, allocation concealment, and blinding, and overall assessments using ROB 2 and CINeMA suggested that the quality of evidence for certain outcomes was low to moderate. These factors highlight the need for caution when interpreting the comparative efficacy of different acupoint stimulation therapies.

Heterogeneity across studies further complicates the interpretation of results. Variations in patient populations, including differences in TCM syndromes, insomnia severity, age, and gender, as well as inconsistencies in intervention protocols–such as acupoint selection, stimulation methods, treatment frequency, and duration–likely contribute to differential responses. In addition, some outcomes were supported by relatively few studies, limiting the stability of SUCRA rankings and widening confidence intervals. The lack of sufficient data also prevented formal subgroup analyses or meta-regression to explore potential effect modifiers, leaving some sources of variability unresolved.

Data on mechanistic pathways and safety were also limited. Only a small number of studies reported changes in inflammatory markers or neurotransmitters, constraining mechanistic interpretation, while adverse events were infrequently and heterogeneously reported, preventing quantitative synthesis and limiting the assessment of safety profiles. Together, these gaps underscore the need for more consistent reporting and comprehensive evaluation in future trials.

Moreover, most studies focused on short-term outcomes, with limited follow-up periods, which restricts the evaluation of sustained efficacy and relapse rates. Addressing these limitations in future research will require rigorously designed trials with standardized interventions, systematic safety monitoring, extended follow-up, and consistent biomarker assessment to provide more robust evidence to guide clinical practice.

## 7 Conclusion

This study systematically evaluated the clinical efficacy of different acupoint stimulation therapies for PI, providing relative rankings of various treatment protocols across different sleep parameters. It also revealed core acupoints and ear points combinations commonly used in insomnia treatment and explored their potential mechanisms in conjunction with modern research. Our meta-analysis not only provides important references for clinical individualized acupoint compatibility, but also suggests that future research can further explore the multi-level regulatory effects of acupoint stimulation on the neuroimmune system. However, the treatment of insomnia requires individualized strategies to optimize the comprehensive management outcomes. Future research should focus on improving study quality, extending follow-up durations, deepening mechanistic studies, and integrating objective monitoring methods to strengthen the evidence base for acupoint stimulation therapies, providing more robust support for the precise treatment of primary insomnia.

## Data Availability

The original contributions presented in the study are included in the article/**Supplementary Material**. Further inquiries can be directed to the corresponding author.

## Glossary

ROB 2: Risk of Bias 2
CINeMA: Confidence in Network Meta-Analysis
SUCRA: Surface Under the Cumulative Ranking
RCTs: Randomized Controlled Trials
PSQI: Pittsburgh Sleep Quality Index
ΔPSQI: Change in PSQI Scores
SQ: Subjective Sleep Quality
SL: Sleep Latency
SD: Sleep Duration
SE: Sleep Efficiency
SDI: Sleep Disturbance Index
DD: Daytime Dysfunction
PRISMA: Preferred Reporting Items for Systematic Reviews and Meta-Analyses
PI: Primary Insomnia
DSM: Diagnostic and Statistical Manual of Mental Disorders
ICD: International Classification of Diseases
ICSD: International Classification of Sleep Disorders
CCMD: Chinese Classification of Mental Disorders
CDTE-TCM: Classification and Diagnostic Criteria of Diseases and Syndromes in Traditional Chinese Medicine
TER: Total Effective Rate
MD: Mean Difference
RR: Relative Risk
CIs: Confidence Intervals
TCM: Traditional Chinese Medicine
IL-1β: Interleukin-1β
TNF-α: Tumor Necrosis Factor-α
GABA: Gamma-Aminobutyric Acid
IL-6: Interleukin-6
CRP: C-Reactive Protein
Glu: Glutamate
I²: I-squared (Measure of Heterogeneity)
HPA: hypothalamic-pituitary-adrenal
AEs: Adverse Events
