# Supplementary material for: Efficacy of multiple acupoint stimulation therapies for primary insomnia patients: a systematic review and network meta-analysis
Source: Front Psychiatry. 2026 Apr 13;17:1678631. doi: 10.3389/fpsyt.2026.1678631 (PMC13111453; doi:10.3389/fpsyt.2026.1678631)
Supplement: Supplementary file 1 [file DataSheet1.pdf]

Appendix Contents

Appendix	Title	Page(s)
-	PROSPERO Registration	1-4
-	Searching Strategies	5-8
-	Data Analysis Methods	9-10
Appendix Table A.1	Acupoint Stimulation Therapies	11
Appendix Tables A.2-A.3	Baseline Characteristics of Included Studies	12-73
Appendix Table A.4	Detailed Risk of Bias Ratings Based on ROB 2.0	74
Appendix Table A.5	Standard Acupuncture Point Locations	75-85
Appendix Table A.6	I-squared (%)	86
Appendix Table A.7	Prediction Interval for Total Effective Rate	87-104
Appendix Table A.8	Prediction Interval for PSQI	105-114
Appendix Table A.9	Prediction Interval for Change in PSQI Scores	115-124
Appendix Table A.10	League Table for Total Effective Rate	125
Appendix Table A.11	League Table for PSQI	126
Appendix Table A.12	League Table for Change in PSQI Scores	127
Appendix Table A.13	League Table for Sleep Quality (SQ)	128
Appendix Table A.14	League Table for Sleep Latency (SL)	129

Appendix Table A.15	League Table for Sleep Duration (SD)	130
Appendix Table A.16	League Table for Sleep Efficiency (SE)	131
Appendix Table A.17	League Table for Sleep Disturbances Index (SDI)	132
Appendix Table A.18	League Table for Daytime Dysfunction (DD)	133
Appendix Fig. A1	Risk of Bias Charts for Included Studies	134
Appendix Figs. A2-A10	Forest Plots for TER, PSQI, ΔPSQI, SQ, SL, SD, SE, SDI, DD	135-139
Appendix Figs. A11-A19	Funnel Plots for TER, PSQI, ΔPSQI, SQ, SL, SD, SE, SDI, DD	139-143
Appendix Figs. A20-A28	Loop Consistency Plots for TER, PSQI, ΔPSQI, SQ, SL, SD, SE, SDI, DD	144-148
Appendix Figs. A29-A37	Rankogram Plots for TER, PSQI, ΔPSQI, SQ, SL, SD, SE, SDI, DD	148-152
Appendix Figs. A38-A46	Cumulative Ranking Probability Plots for TER, PSQI, ΔPSQI, SQ, SL, SD, SE, SDI, DD	153-157
Appendix Figs. A47-A52	Prediction Interval Plots for SQ, SL, SD, SE, SDI, DD	157-160

Efficacy of multiple acupoint stimulation therapies for primary insomnia patients: A systematic review and network meta-analysis

Ying Wang, Jin-ying Zhao, Ning Yang, Yu-hong Xie, Tian-yu Jiang, Fu-chun Wang, Shu-ming Zhao

To enable PROSPERO to focus on COVID-19 submissions, this registration record has undergone basic automated checks for eligibility and is published exactly as submitted. PROSPERO has never provided peer review, and usual checking by the PROSPERO team does not endorse content. Therefore, automatically published records should be treated as any other PROSPERO registration. Further detail is provided here.

Citation

Ying Wang, Jin-ying Zhao, Ning Yang, Yu-hong Xie, Tian-yu Jiang, Fu-chun Wang, Shu-ming Zhao. Efficacy of multiple acupoint stimulation therapies for primary insomnia patients: A systematic review and network meta-analysis. PROSPERO 2025 CRD42025640547 Available from:
https://www.crd.york.ac.uk/prospero/display_record.php?ID=CRD42025640547

Review question

Are different acupoint stimulation therapies effective in treating primary insomnia?
What is the efficacy ranking of different acupoint stimulation therapies for primary insomnia?

Searches

From their inception date until September 2024, without language restriction. We used filters to reliably identify studies and undertook a comprehensive search of four Chinese electronic databases: China National Knowledge Infrastructure (CNKI), VIP Database, Wanfang Database, China biomedical literature service system (SinoMed), and four English electronic databases: Cochrane Library, Web of Science, PubMed, Embase. Searches were supplemented by retrieval of other sources, including the online trial registries such as the US ClinicalTrials.gov, Chinese Clinical Trial Registry, International Traditional Medicine Clinical Trail Registry, International Clinical Trials Registry Platform (ICTRP), and ISRCTN registry.

Types of study to be included

Inclusion criteria
1.P: Patients with primary insomnia.
2.I: The interventions included 13 types of acupoint stimulation therapies such as acupuncture, moxibustion, tuina, gua sha (technique in TCM), cupping technique (acupressure technique of Chinese medicine, with fired vacuum cup applied the skin), and bloodletting therapy.
3.C: The comparisons will only be acupoint stimulation therapies such as acupuncture, moxibustion and tuina.
4.O: studies must report one or more standard and valid sleep scale(s), such as PSQI, ISI, AIS, etc.
5.S: studies must RCTs.
6.Language: only studies published in English and Chinese.
Exclusion criteria
1. The comparison using non-acupoint stimulation or in combination with other non-acupoint stimulation therapies (including medications, herbs, proprietary Chinese medicines, etc.) will be excluded.
2. Case report, personal experience, meta-analysis, conference abstract, systematic review, study protocol data mining analysis, unrelated studies, case series, observational trials, and animal experiments.

Condition or domain being studied

Efficacy of different acupoint stimulation therapies in the treatment of primary insomnia. The main symptoms of primary insomnia include difficulty falling asleep, difficulty maintaining sleep, and inability to fall back asleep after waking up early. These symptoms can lead to fatigue, decreased concentration, and impaired memory, severely affecting the patient's life and work.

Participants/population

The main diagnosis was primary insomnia. However, patient will be excluded if they are comorbid with other disorder/disease.

Intervention(s), exposure(s)

The interventions included 13 types of acupoint stimulation therapies such as acupuncture, moxibustion, tuina, gua sha (technique in TCM), cupping technique (acupressure technique of Chinese medicine, with fired vacuum cup applied the skin), and bloodletting therapy.

Comparator(s)/control

The comparisons will only be acupoint stimulation therapies such as acupuncture, moxibustion and tuina. The comparison using non–acupoint stimulation or in combination with other non–acupoint stimulation therapies (including medications, herbs, proprietary Chinese medicines, etc.) will be excluded.

Main outcome(s)

Sleep scales such as PSQI, ISI, AIS, etc.

Additional outcome(s)

Statistics on clinical effectiveness and adverse events.

Data extraction (selection and coding)

Two authors of our review team plan to independently extract the data from the articles selected for inclusion, and to resolve differences in opinion through discussion with experts. Data will be recorded onto an electronic form, including categories for basic information about the studies (the first author's last name and the year the study was published), the sample sizes and grouping methods used, participant characteristics including age and disease duration, expressed as mean ± standard deviation, and details of the intervention methods involved, including treatment time, treatment cycles, the selection of acupoints, prescription in the control group, outcomes, and side effects.

Risk of bias (quality) assessment

Two authors of our review team will independently assess the methodological quality of included trials, and consensus will be reached by discussion with a third researcher in case of discrepancy. Methodological quality of RCTs will be assessed according to the risk of bias tool described in the Cochrane handbook for systematic reviews of interventions. Seven elements will be assessed: random sequence generation, allocation concealment, blinding of participants and personnel, blinding of outcome assessment, incomplete outcome data, selective reporting and other bias. The Standards for Reporting Interventions in Controlled Trials of Acupuncture (STRICTA) checklist will be used to assess the completeness and reporting quality of the evaluated intervention.

Strategy for data synthesis

When a couple of good multiple homogeneity studies are included, we will perform meta–analyses with Stata 17.0. Categorical variables were analyzed using a RR and continuous variables with a mean difference(MD). For each endpoint of interest, 95% confidence intervals (CI) wereestimated. Outcomes were compared between analyzed papers using therandom effects model. Statistical heterogeneity between papers wasanalyzed using the I^2 statistic, where p value < 0.05 or I^2 > 50% wasconsidered significant. Funnel plots were used to inspect potentialpublication bias (α = 0.05), and p values < 0.05 were considered asstatistically significant.

Analysis of subgroups or subsets

If data permitted, we plan to conduct subgroup analyses for different groups according to different types of acupoint stimulation therapies (moxibustion, tui na, gua sha, cupping, and bloodletting therapies, etc.) in the control group.

Contact details for further information

Ying Wang
wy1875226734@163.com

Organisational affiliation of the review

Changchun University of Chinese Medicine

Review team members and their organisational affiliations

Dr Ying Wang. Changchun University of Chinese Medicine
Jin-ying Zhao. Changchun University of Chinese Medicine
Ning Yang. Changchun University of Chinese Medicine
Yu-hong Xie. Changchun University of Chinese Medicine
Tian-yu Jiang. Changchun University of Chinese Medicine
Professor Fu-chun Wang. Changchun University of Chinese Medicine
Shu-ming Zhao. Changchun University of Chinese Medicine

Type and method of review

Network meta-analysis, Systematic review

Anticipated or actual start date

22 September 2024

Anticipated completion date

31 March 2025

Funding sources/sponsors

The Seventh Batch of Jilin Province Young Science and Technology Talents Support Project (QT202319).

Conflicts of interest

None known

Language

English

Country

China

Stage of review

Review Ongoing

Subject index terms status

Subject indexing assigned by CRD

Subject index terms

MeSH headings have not been applied to this record

Date of registration in PROSPERO

05 February 2025

Date of first submission

25 January 2025

Stage of review at time of this submission

Stage	Started	Completed
Preliminary searches	Yes	Yes
Piloting of the study selection process	Yes	No
Formal screening of search results against eligibility criteria	Yes	No
Data extraction	No	No
Risk of bias (quality) assessment	No	No
Data analysis	No	No

The record owner confirms that the information they have supplied for this submission is accurate and complete and they understand that deliberate provision of inaccurate information or omission of data may be construed as scientific misconduct.

The record owner confirms that they will update the status of the review when it is completed and will add publication details in due course.

Versions

05 February 2025
05 February 2025

PROSPERO
This information has been provided by the named contact for this review. CRD has accepted this information in good faith and registered the review in PROSPERO. The registrant confirms that the information supplied for this submission is accurate and complete. CRD bears no responsibility or liability for the content of this registration record, any associated files or external websites.

Search number	Searching Strategies	Query	Results
1	CNKI	SU%=('失眠'+'失眠症'+'不寐'+'早醒'+'睡眠障碍'+'睡眠异常'+'睡眠不足'+'睡眠困难'+'入睡困难'+'入睡和睡眠失调'+'入睡和睡眠障碍'+'睡眠起始功能障碍') AND SU%=('随机对照试验'+'随机对照'+'随机')	1371
2	VIP Database	(((((((((((题名或关键词=失眠 OR 题名或关键词=失眠症) OR 题名或关键词=不寐) OR 题名或关键词=早醒) OR 题名或关键词=睡眠障碍) OR 题名或关键词=睡眠异常) OR 题名或关键词=睡眠不足) OR 题名或关键词=睡眠困难) OR 题名或关键词=入睡困难) OR 题名或关键词=入睡和睡眠失调) OR 题名或关键词=入睡和睡眠障碍) OR 题名或关键词=睡眠起始功能障碍) AND ((任意字段=随机对照试验 OR 任意字段=随机对照) OR 任意字段=随机))	12060
3	WanFang	主题:(失眠 OR 失眠症 OR 不寐 OR 早醒 OR 睡眠障碍 OR 睡眠异常 OR 睡眠不足 OR 睡眠困难 OR 入睡困难 OR 入睡和睡眠失调 OR 入睡和睡眠障碍 OR 睡眠起始功能障碍) and 主题:(随机对照试验 OR 随机对照 OR 随机)	27405
4	CBM	序号 检索表达式 命中文献数 检索时间<br>1) "入睡和睡眠障碍"[不加权:扩展] 22896 2024-09-21 08:51:30.0<br>2) 失眠 39759 2024-09-21 08:54:12.0<br>3) 失眠症 39759 2024-09-21 08:54:18.0<br>4) 不寐 26084 2024-09-21 08:54:26.0<br>5) 早醒 39759 2024-09-21 08:54:33.0<br>6) 睡眠障碍 62352 2024-09-21 08:54:42.0<br>7) 睡眠异常 45423 2024-09-21 08:54:50.0<br>8) 睡眠不足 1282 2024-09-21 08:54:57.0<br>9) 睡眠困难 265 2024-09-21 08:55:05.0<br>10) 入睡困难 2090 2024-09-21 08:55:13.0<br>11) 入睡和睡眠失调 39759 2024-09-21 08:55:21.0<br>12) 入睡和睡眠障碍 39759 2024-09-21 08:55:29.0<br>13) 睡眠起始功能障碍 39759 2024-09-21 08:55:38.0<br>14) (#13) OR (#12) OR (#11) OR (#10) OR (#9) OR (#8) OR (#7) OR (#6) OR (#5) OR (#4) OR (#3) OR (#2) OR (#1) 79841 2024-09-21 08:55:51.0<br>15) "随机对照试验"[不加权:扩展] 198307 2024-09-21 08:56:15.0<br>16) 随机对照 274359 2024-09-21 08:56:24.0<br>17) 随机 1988014 2024-09-21 08:56:32.0<br>18) (#17) OR (#16) OR (#15) 1988240 2024-09-21 08:56:44.0<br>19) (#18) AND (#14) 25187 2024-09-21 08:57:08.0	25187

5	Pubmed	(("Sleep Initiation and Maintenance Disorders"[Mesh]) OR (((((((((((((((((insomnia[Title/Abstract]) OR (insomnias[Title/Abstract])) OR (insomina disorder[Title/Abstract])) OR (insomina disorders[Title/Abstract])) OR (sleep disorder[Title/Abstract])) OR (sleep disorders[Title/Abstract])) OR (sleep problem[Title/Abstract])) OR (sleep disturbance[Title/Abstract])) OR (sleep quality[Title/Abstract])) OR (sleeplessness[Title/Abstract])) OR (insufficient sleep[Title/Abstract])) OR (agrypnia[Title/Abstract])) OR (dyssomnia[Title/Abstract])) OR (dyssomnias[Title/Abstract])) OR (disorders of initiating[Title/Abstract] AND maintaining Sleep[Title/Abstract])) OR (DIMS[Title/Abstract])) OR (early awakening[Title/Abstract])) OR (Sleep Initiation Dysfunction[Title/Abstract])) OR (Sleep Initiation Dysfunctions[Title/Abstract]))) AND (randomized controlled trial[Publication Type] OR randomized[Title/Abstract] OR placebo[Title/Abstract])	11763
6	EMBASE	#23. #21 AND #22<br>#22. random:ti,ab OR placebo:ti,ab OR 'double blind':ti,ab<br>#21. #1 OR #2 OR #3 OR #4 OR #5 OR #6 OR #7 OR #8 OR #9 OR #10 OR #11 OR #12 OR #13 OR #14 OR #15 OR #16 OR #17 OR #18 OR #19 OR #20<br>#20. 'sleep initiation dysfunctions':ti,ab<br>#19. 'sleep initiation dysfunction':ti,ab<br>#18. 'early awakening':ti,ab<br>#17. 'sleep initiation and maintenance disorders':ti,ab<br>#16. 'dims':ti,ab<br>#15. 'disorders of initiating and maintaining sleep':ti,ab<br>#14. 'dyssomnias':ti,ab<br>#13. 'dyssomnia':ti,ab<br>#12. 'agrypnia':ti,ab<br>#11. 'insufficient sleep':ti,ab<br>#10. 'sleeplessness':ti,ab<br>#9. 'sleep quality':ti,ab<br>#8. 'sleep disturbance':ti,ab<br>#7. 'sleep problem':ti,ab<br>#6. 'sleep disorders':ti,ab<br>#5. 'sleep disorder':ti,ab<br>#4. 'insomina disorders':ti,ab<br>#3. 'insomina disorder':ti,ab<br>#2. 'insomnias':ti,ab<br>#1. 'insomnia'/exp OR 'insomnia'	17902

7	Cochrance libiary	#1 MeSH descriptor: [Sleep Initiation and Maintenance Disorders] explode all trees<br>#2 (DIMS):ti,ab,kw OR (early awakening):ti,ab,kw OR (Sleep Initiation Dysfunction):ti,ab,kw OR (Sleep Initiation Dysfunctions):ti,ab,kw (Word variations have been searched)<br>#3 (disorders of initiating and maintaining sleep):ti,ab,kw OR (dyssomnias):ti,ab,kw OR (dyssomnia):ti,ab,kw OR (agrypnia):ti,ab,kw OR (insufficient sleep):ti,ab,kw (Word variations have been searched)<br>#4 (sleeplessness):ti,ab,kw OR (sleep quality):ti,ab,kw OR (sleep disturbance):ti,ab,kw OR (sleep problem):ti,ab,kw OR (sleep disorders):ti,ab,kw (Word variations have been searched)<br>#5 (sleep disorder):ti,ab,kw OR (insomina disorders):ti,ab,kw OR (insomina disorder):ti,ab,kw OR (insomnias):ti,ab,kw OR (insomnia):ti,ab,kw (Word variations have been searched)<br>#6 #1 or #2 or #3 or #4 or #5	48335
8	Web of Science	#1 ((((((((((((((((((TS=(insomnia)) OR TS=(insomnias)) OR TS=(insomina disorder)) OR TS=(insomina disorders)) OR TS=(sleep disorder)) OR TS=(sleep disorders)) OR TS=(sleep problem)) OR TS=(sleep disturbance)) OR TS=(sleep quality)) OR TS=(sleeplessness)) OR TS=(insufficient sleep)) OR TS=(agrypnia)) OR TS=(dyssomnia)) OR TS=(dyssomnias)) OR TS=(disorders of initiating and maintaining sleep)) OR TS=(DIMS)) OR TS=(sleep initiation and maintenance disorders)) OR TS=(early awakening)) OR TS=(Sleep Initiation Dysfunction)) OR TS=(Sleep Initiation Dysfunctions)))<br>#2 TS=(random* controlled trial OR random* OR placebo)<br>#2 AND #1	22468
9	ClinicalTrials.gov	insomnia+with result	0
10	Chinese clinical trial registry	失眠+with/without result	60
11	International Traditional Medicine Clincal Trail Registry	失眠	1
12	International Clinical Trials Registry Platform (ICTRP)	insomnia	4

13	ISRCTN registry	insomnia	5

1.#If the data is in arm-based format, data preprocessing should be performed first：

network setup r n, studyvar(id) trtvar(t) format(augment) rr

network setup mean sd n,studyvar(study) trtvar(trt) format(augment)

2.#Perform the inconsistency model test by typing the following in the command line:

network meta i

network meta c

3.#Forest plot

network forest

4.#To create a pairwise comparison forest plot, you can type the following code in the command line (assuming you're using Stata):：

intervalplot, null(0) lab( )

5.#Node-splitting method

network sidesplit all, tau

6.#To create a network evidence plot

network map, improve

7.#Loop inconsistency

ifplot _y _stderr _t1 _t2 id, tau2(loop)

8.#To create a comparison-adjusted funnel plot using a random-effects model in stata

netfunnel _y _stderr _t1 _t2, bycomparison ytitle(Standard error of logor) addplot(lfit _stderr _ES_CEN)

9.#netleague

netleague, lab() sort()

10.#To create a prediction interval plot

```
intervalplot, eform pred null(1) lab()

intervalplot, pred null(0) lab()
```

11.#sucra

```
network rank min/max, all zero reps(5000) gen(prob)

sucra prob*, labels() rankog

sucra prob*, labels()
```

12.#I²

#Input Data

study, _y (MD), _stderr (SE)

#Calculating Weights and Q

```
gen w = 1 / (_stderr^2)

egen total_w = total(w)

gen wY = w * _y

egen total_wY = total(wY)

scalar Y_bar = total_wY / total_w

gen Q_component = w * (_y - Y_bar)^2

egen Q = total(Q_component)

scalar Q_total = Q[1]
```

#Degrees of freedom (df) and I²

```
list study

quietly count

display "Number of studies (k) = " r(N)

scalar df = r(N) - 1

scalar I2 = max(0, (Q_total - df)/Q_total * 100)
```

#Output the results

```
display "Q = " Q_total ", df = " df

display "I-squared = " I2 "%"
```

Table A1. Acupoint stimulation therapies.

Number	Acupoint stimulation therapies
1	Body acupuncture
2	Moxibustion
3	Cupping therapy
4	Guasha therapy
5	Bloodletting therapy
6	Specialized acupuncture needles
7	Specialized acupuncture point therapies
8	Scalp acupuncture
9	Ear acupuncture
10	Eye acupuncture
11	Electroacupuncture
12	auricular acupressure
13	Tuina
14	Cerebral circulation electrical stimulation

Table A2. Baseline characteristics of included studies.

Study ID	Sample size (I/C)	Gender (male /female)	Age (mean or range, years)	Duration (mean or range)	Regimen (I/C)	Diagnostic criteria	TCM syndrome type	Follow up	Adverse Events
Tang T et al. 2006[1]	30/30	32/28	I, 20~68<br>C, 21~69	I, 7d~1y<br>C, 6d~1y	AC/L	NA	NA	NA	NA
Xing QJ 2007[2]	40/40	45/35	I, 39.46 (18.3~64.9)<br>C, 39.9 (18~64.11)	I, 6.5m<br>C, 6.5m	AL/A	①CCMD-3-R<br>②Guidelines for Clinical Research of New Chinese Medicines (1993 edition)	①Hyperactivity of liver yang pattern<br>②Deficiency of the heart and spleen pattern<br>③Disharmony between the heart and the kidney<br>④Disharmony between the spleen and stomach pattern	30d	I, 0<br>C, 0
Wang YM et al. 2007[3]	38/38	NA	I, 35.3 (30~41)<br>C, 34.8 (29~41)	I, 2~4y and 6m<br>C, 2~4y and 6m	AB/A	CCMD-3	NA	NA	NA
Yang L et al. 2008[4]	63/60	48/75	I, 40.27±12.76<br>C, 39.63±11.91	I, 21.76±9.43m<br>C, 20.24±10.87 m	EK/K	Psychiatry (2002 edition)	NA	NA	NA
Xia Y et al. 2008[5]	30/30	26/34	I, 19~58<br>C, 18~63	I, 38d~7y<br>C, 28d~10y	AB/A	CCMD-2-R	NA	NA	NA

Table A2. Baseline characteristics of included studies (continued).

Study ID	Sample size (I/C)	Gender (male /female)	Age (mean or range, years)	Duration (mean or range)	Regimen (I/C)	Diagnostic criteria	TCM syndrome type	Follow up	Adverse Events
Gao XY et al. 2008[6]	100/100	105/95	I, 39.46±12.6<br>C, 39.90±13.40	>1m	AL/A	①CCMD<br>②Guidelines for Clinical Research of New Chinese Medicines (1993 edition)	①Hyperactivity of liver yang pattern<br>②Deficiency of the heart and spleen pattern<br>③Disharmony between the heart and the kidney<br>④Disharmony between the spleen and stomach pattern	30d	I, 0<br>C, 0
Liang YL et al. 2009[7]	50/50	42/58	I, 19~70<br>C, 17~68	I, 10d~8y<br>C, 1w~10y	AE/A	CCMD-3	NA	3m	NA
Li ZP et al. 2010[8]	30/30	28/32	I, 35.57±6.74<br>C, 36.69±7.30	I, 19.43±10.25<br>C, 20.55±11.46	AG/A	CCMD-3	NA	1m	NA
Chen FM 2010[9]	29/30	23/36	I, 43.34±10.47 (27~62)<br>C, 42.40±10.20 (25~63)	I, 18.83±1.63m (2m~5y)<br>C, 20.27±1.90m (2m~6y)	AE/A	①CCMD-4<br>②Traditional Chinese Internal Medicine (2002 edition)	①Liver fire disturbing heart syndrome<br>②Phlegm fire disturbing the heart pattern<br>③Deficiency of the heart and spleen pattern<br>④Disharmony between the heart and the kidney<br>⑤Qi deficiency of the heart and gallbladder pattern	NA	I, 0<br>C, 0

Table A2. Baseline characteristics of included studies (continued).

Study ID	Sample size (I/C)	Gender (male /female)	Age (mean or range, years)	Duration (mean or range)	Regimen (I/C)	Diagnostic criteria	TCM syndrome type	Follow up	Adverse Events
Shi YJ et al. 2011[10]	33/31	31/33	I, 44±15 (18~69) C, 40±16 (18~67)	I, 20.5±16.8m (1m~6y) C, 18.4±11.8m (1m~4y)	G/A	Classification and Diagnostic Criteria of Mental Disorders (2005 edition)	NA	NA	NA
Lian FC et al. 2011[11]	39/39	27/51	I, 47.5 C, 44.8	I, 2~6y C, 1~6y	CE/A	①Practical Internal Medicine ②Psychiatry ③Diagnostic Criteria for Internal Medicine Diseases	NA	NA	NA
Gu RX et al. 2011[12]	30/30	25/35	I, 45±6 C, 44±6	I, 3.43±2.45y C, 3.10±2.12y	G/A	CCMD	①Deficiency of the heart and spleen pattern ②Qi deficiency of the heart and gallbladder pattern ③Yin deficiency leading to fire hyperactivity ④Liver qi stagnation transforming into fire pattern ⑤Pattern of internal harassment of phlegm-heat	NA	NA

Table A2. Baseline characteristics of included studies (continued).

Study ID	Sample size (I/C)	Gender (male /female)	Age (mean or range, years)	Duration (mean or range)	Regimen (I/C)	Diagnostic criteria	TCM syndrome type	Follow up	Adverse Events
Li ZP et al. 2011a[13]	100/98	91/107	I, 35.58±9.87<br>C, 36.67±10.93	I, 21.59±7.87m<br>C, 22.76±8.39m	AB/A	CCMD-3	①Liver fire disturbing heart syndrome<br>②Phlegm fire disturbing the heart pattern<br>③Deficiency of the heart and spleen pattern<br>④Disharmony between the heart and the kidney<br>⑤Qi deficiency of the heart and gallbladder pattern	NA	I, 6 (burn 1, needle shock 1, acupuncture -induced haematoma 4)<br>C, 5 (needle shock 3, acupuncture -induced haematoma 2)
Li ZP et al. 2011b[14]	60/60	51/69	I, 43.67±13.99 (20~72)<br>C, 44.68±14.07 (22~65)	I, 29.08±47.61 (6~241)m<br>C, 29.85±77.50 (6~482)m	AG/A	CCMD-3	NA	NA	I, 1 (local infection 1)<br>C, 1 (hemorrhage 1)
Shi L et al. 2011[15]	40/40	32/48	I, 45.41±2.21 (22~57)<br>C, 44.21±2.35 (23~56)	I, 6.4±1.5 (0.3~15)y<br>C, 6.1±1.2 (0.4~14)y	G/A	Insomnia (2005 edition)	NA	6m and 1y	NA
Feng X et al. 2012[16]	30/30	22/38	I, 30~66<br>C, 30~68	I, 2m~9y<br>C, 2m~10y	AG/A	NA	NA	NA	NA

Table A2. Baseline characteristics of included studies (continued).

Study ID	Sample size (I/C)	Gender (male /female)	Age (mean or range, years)	Duration (mean or range)	Regimen (I/C)	Diagnostic criteria	TCM syndrome type	Follow up	Adverse Events
Li ZM et al. 2012[17]	30/30	25/35	I, 35±2 (20~59) C, 36±3 (21~58)	I, 3±0.2y (1m~6y) C, 3±0.4y (1m~5.5y)	AM/A	CDTE-TCM (1994 edition)	NA	NA	NA
Shi MW 2012[18]	20/20	19/21	I, 18~65 C, 18~65	≥1 m	AB/A	①CCMD-3 ②Guidelines for Clinical Research of New Chinese Medicines (1993 edition) ③CDTE-TCM (1994 edition)	①Liver qi stagnation transforming into fire pattern ②Yin deficiency leading to fire hyperactivity ③Deficiency of the heart and spleen pattern ④Pattern of heart deficiency with timidity	1m	I, 0 C, 0
Qiu FX et al. 2012[19]	20/20	19/21	25~75	1m~10y	AG/A	CDTE-TCM (1994 edition)	NA	NA	NA
Lin YH et al. 2012[20]	45/45	44/46	I, 19~68 C, 20~69	I, 1~15y C, 1~18y	AL/A	CDTE-TCM (1994 edition)	NA	NA	NA
Jian HL 2012[21]	30/30	29/31	I, 48.45±5.24 C, 47.82±5.31	I, 28.52±3.57m C, 27.31±3.61m	KL/K	①CCMD-3 ②CDTE-TCM (1994 edition)	①Liver qi stagnation transforming into fire pattern ②Phlegm fire disturbing the heart pattern ③Yin deficiency leading to fire hyperactivity ④Deficiency of the heart and spleen pattern ⑤Pattern of heart deficiency with timidity	NA	NA

Table A2. Baseline characteristics of included studies (continued).

Study ID	Sample size (I/C)	Gender (male /female)	Age (mean or range, years)	Duration (mean or range)	Regimen (I/C)	Diagnostic criteria	TCM syndrome type	Follow up	Adverse Events
Yao HF et al. 2012[22]	33/31	31/33	I, 43.5±15.4 (18~69)<br>C, 40.4±15.5 (18~67)	I, 20.5±16.8m (1m~6y)<br>C, 18.4±11.8m (1m~4y)	G/A	CCMD-2	NA	NA	NA
Gao XY et al. 2013[23]	57/59	49/71	I, 39±13<br>C, 40±13	>6m	AB/A	CCMD-3	NA	NA	NA
Zhang Y et al. 2013[24]	112/112	92/132	18~75	14d~30y	ABGN/K	CDTE-TCM (1994 edition	NA	NA	NA
Zhang HF et al. 2013[25]	33/31	31/33	I, 43.50±15.40 (18~69)<br>C, 40.40±15.5 (18~67)	I, 20.50±16.82 m (1m~6y)<br>C, 18.40±11.82 m (1y~4y)	G/A	Classification and Diagnostic Criteria of Mental Disorders (1995 edition)	NA	NA	NA
Guo AS et al. 2013[26]	35/35	43/27	40.0±16.21 (18~65)	3.9±2.73 (1~10)y	G/A	CCMD-3	NA	NA	I, 3 (pain 3)<br>C, 0

Table A2. Baseline characteristics of included studies (continued).

Study ID	Sample size (I/C)	Gender (male /female)	Age (mean or range, years)	Duration (mean or range)	Regimen (I/C)	Diagnostic criteria	TCM syndrome type	Follow up	Adverse Events
Cao JK 2013[27]	32/33	31/34	I, 42.59±13.37<br>C, 45.79±12.34	I, 19.19±10.24 m<br>C, 18.97±10.25 m	H/A	①CCMD-3<br>②CDTE-TCM (1994 edition)	①Liver qi stagnation transforming into fire pattern<br>②Phlegm fire disturbing the heart pattern<br>③Yin deficiency leading to fire hyperactivity<br>④Deficiency of the heart and spleen pattern<br>⑤Pattern of heart deficiency with timidity	NA	NA
Xiong QJ et al. 2013[28]	30/30	32/28	38.5±0.2 (18~66)	2.5±0.8y (1m~10y)	KM/A	NA	NA	NA	NA
Wei Y et al. 2013[29]	50/45	52/43	I, 37±5 (22~74)<br>C, 42±6 (24~76)	I, 3.12±0.91y (3m~8y)<br>C, 3.42±0.72y (8m~10y)	AB/A	Zhuang Medicine Internal Medicine (2006 edition)	NA	NA	I, 0<br>C, 0
Gu TT et al. 2013[30]	30/30	25/35	I, 45.62±4.17 (22~67)<br>C, 43.92±4.9 (21~65)	I, 35d~17y<br>C, 34d~18y	BL/B	ICD-10	NA	NA	NA
Sun YZ et al. 2014[31]	30/30	31/29	I, 42.7±8.4<br>C, 44.5±6.7	I, 13.2±7.4m<br>C, 14.6±7.1m	AK/K	CCMD-3	NA	NA	NA
Feng SS et al. 2014[32]	20/20	18/22	I, 45±4.17 (25~68)<br>C, 46±3.35 (23~71)	I, 3±6.18 (1~12)w<br>C, 4±6.24 (1~12)w	L/A	Traditional Chinese Internal Medicine (2007 edition)	NA	NA	NA

Table A2. Baseline characteristics of included studies (continued).

Study ID	Sample size (I/C)	Gender (male /female)	Age (mean or range, years)	Duration (mean or range)	Regimen (I/C)	Diagnostic criteria	TCM syndrome type	Follow up	Adverse Events
Fu DM et al. 2014[33]	60/60	56/64	I, 46.3 (21~54) C, 41.8 (18~56)	I, 1.3y (3w~7y) C, 1.5y (4w~3y)	AL/A	CDTE-TCM (1994 edition)	NA	NA	NA
Ma QY et al. 2014[34]	99/96	71/124	I, 38±13 (20~64) C, 37±12 (21~62)	I, 7.48±4.57 (0.5~20)y C, 7.13±4.92 (0.5~20)y	BL/L	CCMD-3	NA	NA	NA
Wang YQ et al. 2014[35]	40/40	42/38	52.68 (18~72)	1~3m	M/K	CCMD-3	NA	NA	NA
Xie ZY et al. 2015a[36]	30/30	33/27	I, 43.2±12.8 (20~63) C, 42.5±13.4 (21~61)	I, 11.5±5.3 (1~30)m C, 10.8±4.7 (1~28)m	LM/M	CCMD-3	①Yin deficiency leading to fire hyperactivity ②Liver qi stagnation transforming into fire pattern ③Deficiency of the heart and spleen pattern ④Qi deficiency of the heart and gallbladder pattern ⑤Phlegm-Humor Disturbing the Heart	NA	NA
Xie ZY et al. 2015b[37]	30/30	31/29	I, 43.3±13.8 C, 42.7±12.4	I, 11.5±5.3m C, 10.8±4.7m	BM/M	CCMD-3	NA	NA	NA
Zhao JP et al. 2015[38]	46/46	40/52	I, 46.0±20.1 C, 45.3±20.3	I, 12.1±4.9m C, 12.2±4.8m	AM/A	①CCMD-3 ②CDTE-TCM (1994 edition)	NA	NA	NA
Liu ZL et al. 2015[39]	40/40	43/37	I, 50±8 C, 50±9	I, 10.08±7.47y C, 9.47±8.92y	G/A	CCMD-3	NA	NA	NA

Table A2. Baseline characteristics of included studies (continued).

Study ID	Sample size (I/C)	Gender (male /female)	Age (mean or range, years)	Duration (mean or range)	Regimen (I/C)	Diagnostic criteria	TCM syndrome type	Follow up	Adverse Events
Xu LL et al. 2015[40]	36/33	26/43	I, 47 (25~68) C, 43 (17~71)	I, 3y (1m~10y) C, 4y (0.5~12y)	KI/L	ICD-10	NA	NA	NA
Chen P et al. 2015[41]	25/25	13/37	18~70	>30d	BL/L	CCMD-3	NA	NA	NA
Huang G 2015[42]	32/32	40/24	54.6±2.9 (46~79)	NA	AM/A	NA	NA	NA	NA
Li BK 2016[43]	50/50	41/59	I, 56.32±0.29 (26~73) C, 56.25±0.21 (25~72)	I, 6.43±0.31 (2~9)y C, 6.51±0.25 (2~8)y	AL/A	NA	NA	NA	NA
Wang FY 2016[44]	44/44	34/54	16~68	3m~10y	K/A	CCMD-3	①Deficiency of the heart and spleen pattern ②Yin deficiency leading to fire hyperactivity ③Pattern of stomach qi disharmony ④Pattern of liver fire flaming up	3m	NA
Liu LN 2016[45]	40/37	38/39	56 (35~86)	1m~6y	LM/L	Guidelines for Clinical Research of New Chinese Medicines (1993 edition)	NA	NA	NA

Table A2. Baseline characteristics of included studies (continued).

Study ID	Sample size (I/C)	Gender (male /female)	Age (mean or range, years)	Duration (mean or range)	Regimen (I/C)	Diagnostic criteria	TCM syndrome type	Follow up	Adverse Events
Wu J et al. 2016[46]	20/20	25/15	I, 37.6±9.4<br>C, 35.2±11.3	NA	GL/A	Expert consensus on the definition, diagnosis, and pharmacological treatment of insomnia (draft) (2004 edition)	NA	NA	NA
Zhou LL 2016[47]	36/36	34/38	I, 39.61±11.10<br>C, 38.94±9.92	I, 22.94±4.37m<br>C, 23.81±5.41m	AB/A	①CCMD-3<br>②CDTE-TCM(1994 edition)	NA	1m	NA
Chang XL 2016[48]	33/32	37/28	I, 47.14±8.36<br>C, 47.07±9.62	I, 1.89±1.32y<br>C, 1.93±1.21y	KL/K	①CCMD-3;<br>②CDTE-TCM (2000 edition)	①Liver qi stagnation transforming into fire pattern<br>②Phlegm fire disturbing the heart pattern<br>③Yin deficiency leading to fire hyperactivity<br>④Deficiency of the heart and spleen pattern<br>⑤Pattern of heart deficiency with timidity	NA	NA
Zhang SN et al. 2016[49]	40/40	36/44	42.45 (12~79)	4.6y (2m~17y)	AM/A	CCMD-3	NA	NA	NA
Pan XM et al. 2016[50]	62/60	31/91	I, 47.32±3.70 (25~76)<br>C, 8.13±3.85 (26~75)	I, 2.63±0.62y (1m~12y)<br>C, 2.79±0.71y (2m~13y)	K/A	NA	NA	NA	NA

Table A2. Baseline characteristics of included studies (continued).

Study ID	Sample size (I/C)	Gender (male /female)	Age (mean or range, years)	Duration (mean or range)	Regimen (I/C)	Diagnostic criteria	TCM syndrome type	Follow up	Adverse Events
Ning SY et al. 2016[51]	60/60	63/57	I, 40.3±11.4 (21~65) C, 40.9±11.8 (23~65)	I, 35.9±15.8 (10~73)m C, 38.7±16.3 (9~82)m	AL/A	①CCMD-3 ②DSM-4	NA	NA	NA
Zhao YQ et al. 2016[52]	43/43	35/51	49.5±12.7 (28~71)	22.5±4.6 (15~30)d	B/A	CCMD-3	NA	NA	NA
Ma WH et al. 2017[53]	35/35	NA	25~70	NA	AB/A	①CCMD-3 ②Guidelines for Clinical Research of New Chinese Medicines (2002 edition)	NA	1m	NA
Hong YF et al. 2017[54]	30/30	20/40	I, 50±11 C, 54±13	NA	BH/A	ICD-10	NA	NA	NA
Chen Y et al. 2018[55]	30/30	27/33	I, 45.3±11.2 C, 42.1±11.6	I, 56.7±35.1 C, 46.1±33.6	BE/B	DSM-4	NA	NA	I, 0 C, 0
Lu CL et al. 2018[56]	34/34	21/47	38~66	>6m	AC/K	CDTE-TCM	NA	NA	NA
Zhao JY et al. 2018[57]	30/30	NA	18~75	NA	K/A	CCMD-3	NA	NA	NA
Lu WJ 2018[58]	30/30	18/42	I, 41.52±6.02 C, 40.83±7.13	I, 2.08±1.07y (3m~5y) C, 2.25±1.10y (2m~4y)	AE/A	①CCMD-3 ②CDTE-TCM	NA	NA	NA

Table A2. Baseline characteristics of included studies (continued).

Study ID	Sample size (I/C)	Gender (male /female)	Age (mean or range, years)	Duration (mean or range)	Regimen (I/C)	Diagnostic criteria	TCM syndrome type	Follow up	Adverse Events
Yao YJ 2019[59]	30/30	31/29	I, 38.14±9.76<br>C, 38.69±11.08	I, 30.36±18.33 m<br>C, 36.79±20.14 m	AE/A	①DSM-4<br>②Traditional Chinese Internal Medicine (2004 edition)	①Liver qi stagnation transforming into fire pattern<br>②Phlegm fire disturbing the heart pattern<br>③Deficiency of the heart and spleen pattern<br>④Disharmony between the heart and the kidney<br>⑤Qi deficiency of the heart and gallbladder pattern	NA	I, 0<br>C, 0
Li YT 2019[60]	30/30	17/43	I, 50.27±9.51 (30~65)<br>C, 51.10±10.82 (32~65)	I, 14.57±7.80 (2~36)m<br>C, 14.37±8.95 (1~40)m	FJ/A	①Guideline for the evaluation and treatment of insomnia in Chinese adults (2018 edition)<br>②Guidelines for Diagnosis and Treatment of Common Internal Medicine Diseases in Traditional Chinese Medicine (2008 edition)	①Deficiency of the heart and spleen pattern<br>②Phlegm fire disturbing the heart pattern<br>③Liver qi stagnation transforming into fire pattern	NA	NA

Table A2. Baseline characteristics of included studies (continued).

Study ID	Sample size (I/C)	Gender (male /female)	Age (mean or range, years)	Duration (mean or range)	Regimen (I/C)	Diagnostic criteria	TCM syndrome type	Follow up	Adverse Events
Hong QY et al. 2019[61]	30/30	21/39	I, 46.2±5.3 (25~63) C, 45.4±6.1 (24~65)	I, 25.7±13.1m (12m~6y) C, 26.4±14.5m (15m~6.5y)	AF/A	①DSM-5 ②CDTE-TCM (1994 edition)	NA	1m	I, 1 (acupuncture-induced haematoma 1) C, 1 (pain 1)
Wu XQ et al. 2019[62]	39/41	29/51	I, 55.2±10.11 (29~78) C, 54.3±12.2 (31~80)	I, 3.55±1.22 (0.5~12)y C, 5.12±2.15 (0.6~12)y	ACD/A	CDTE-TCM (1994 edition)	①Liver qi stagnation pattern ②Phlegm fire disturbing the heart pattern ③An excess of heat in the heart ④Deficiency of the heart and spleen pattern ⑤Qi deficiency of the heart and gallbladder pattern	NA	NA
Yan QY 2019[63]	20/20	18/22	I, 30~75 C, 29~75	I, 1m~2 or 3y C, 2m~4y	AB/B	Guidelines for Clinical Research of New Chinese Medicines	NA	NA	NA
Zhou Y et al. 2019[64]	46/43	40/49	I, 52.37±11.28 C, 51.85±12.13	I, 8.28±5.43y C, 8.79±5.12y	FG/G	CCMD-3	NA	NA	NA
Zhou YB et al. 2020[65]	30/30	24/36	I, 42.17±8.36 C, 40.57±10.23	I, 14.22±5.66w C, 15.65±6.34w	AL/A	CCMD-3	NA	NA	NA

Table A2. Baseline characteristics of included studies (continued).

Study ID	Sample size (I/C)	Gender (male /female)	Age (mean or range, years)	Duration (mean or range)	Regimen (I/C)	Diagnostic criteria	TCM syndrome type	Follow up	Adverse Events
Wu HH 2020[66]	60/60	54/66	I, 44±8 (23~74) C, 45±7 (20~75)	I, 5.2±2.2 (1~22)m C, 5.3±2.1 (1~23)m	ACF/A	CCMD-3	NA	NA	NA
Shi XD et al. 2020[67]	52/55	35/72	I, 54.3±7.2 C, 52.3±5.2	I, 2.86±2.98y C, 2.62±2.76y	AM/M	①CCMD-3 ②Guidelines for Clinical Research of New Chinese Medicines (1993 edition) ③CDTE-TCM (1994 edition)	NA	NA	NA
Wu JL et al. 2020[68]	35/34	30/39	I, 42±10 C, 43±10	I, 8.97±6.63m C, 8.44±6.43m	K/A	①DSM-5 ②Guidelines for Clinical Research of New Chinese Medicines (1993 edition)	NA	2m	NA
Li ZB et al. 2020[69]	29/29	19/39	I, 56.34±5.51 (21~79) C, 56.23±5.45 (22~78)	I, 3.56±1.31m C, 3.48±1.28m	AK/K	CCMD-3	NA	NA	NA
Cao WT 2020[70]	50/50	42/58	I, 49.56±6.32 (22~71) C, 50.06±6.71 (20~73)	I, 10.23±2.58 (3~20)w C, 9.85±2.18 (3~19)w	AJ/A	NA	NA	NA	I, 0 C, 0

Table A2. Baseline characteristics of included studies (continued).

Study ID	Sample size (I/C)	Gender (male /female)	Age (mean or range, years)	Duration (mean or range)	Regimen (I/C)	Diagnostic criteria	TCM syndrome type	Follow up	Adverse Events
Li JY 2020[71]	32/32	27/37	I, 20~60<br>C, 20< or 20~60	I, 3~36 or >36w<br>C, 3~36 or >36w	F/A	①ICSD-3<br>②Traditional Chinese Internal Medicine (1985 edition)	①Liver fire disturbing heart syndrome<br>②Phlegm fire disturbing the heart pattern<br>③Deficiency of the heart and spleen pattern<br>④Qi deficiency of the heart and gallbladder pattern<br>⑤Yin deficiency leading to fire hyperactivity	NA	NA
Wang YP 2020[72]	44/44	48/36	I, 38.54±2.11 (23~62)<br>C, 37.49±2.16 (22~60)	NA	M/D	NA	NA	NA	NA
Zhang XY et al. 2020[73]	32/30	27/37	I, 44.21±10.80 (23~69)<br>C, 43.52±11.51 (20~67)	I, 5.54±2.92 (0.3~17)y<br>C, 5.72±3.10 (0.4~15)y	F/K	①CCMD-3<br>②CDTE-TCM (1994 edition)	NA	1m	NA
Guo SX et al. 2020[74]	30/30	37/23	I, 56.88±4.96 (45~69)<br>C, 57.46±4.89 (47~68)	I, 6.14±2.25 (1~12)y<br>C, 5.93±2.17 (1~11)y	DL/L	NA	NA	NA	NA
Chen XM 2020[75]	30/30	34/26	I, 57.12±1.1 (31~76)<br>C, 57.05±1.2 (34~76)	NA	AG/A	Guideline for the evaluation and treatment of insomnia in Chinese adults (2012 edition)	NA	NA	NA

Table A2. Baseline characteristics of included studies (continued).

Study ID	Sample size (I/C)	Gender (male /female)	Age (mean or range, years)	Duration (mean or range)	Regimen (I/C)	Diagnostic criteria	TCM syndrome type	Follow up	Adverse Events
Wei M 2020[76]	29/27	19/37	I, 39.31±11.41<br>C, 37.52±11.05	I, 3.67±2.99y<br>C, 3.43±2.73y	M/F	①ICSD-3<br>②Interpretation of Clinical Diagnosis and Treatment Guidelines of Traditional Chinese Medicine: Neurology Volume (2015 edition)	NA	NA	I, 1 (daytime drowsiness 1)<br>C, 1 (pain 1)
Ye YW et al. 2021[77]	57/57	58/56	I, 35.65±4.21 (22~56)<br>C, 34.65±4.19 (24~58)	I, 2.32±0.89 (1~6)y<br>C, 2.51±0.78 (1~5)y	BM/M	Traditional Chinese medicine diagnosis, syndrome differentiation, and treatment of insomnia	NA	NA	I, 3 (dizziness 2, vomiting 1)<br>C, 5 (dizziness 3, vomiting 1, dry mouth 1)
Chen Y et al. 2021[78]	40/40	32/48	I, 42.93±13.10 (21~62)<br>C, 42.27±13.56 (20~61)	>6m	FH/A	NA	NA	NA	NA
Cao WQ et al. 2021[79]	30/30	33/27	I, 46.46±4.42 (21~57)<br>C, 46.25±4.23 (22~56)	I, 13.45±2.44 (1~17)y<br>C, 13.25±2.42 (1~15)y	AF/A	NA	NA	NA	NA

Table A2. Baseline characteristics of included studies (continued).

Study ID	Sample size (I/C)	Gender (male /female)	Age (mean or range, years)	Duration (mean or range)	Regimen (I/C)	Diagnostic criteria	TCM syndrome type	Follow up	Adverse Events
Lan L et al. 2021[80]	50/50	34/66	I, 51.32±9.21<br>C, 50.52±8.01	NA	AL/L	①CCMD-3<br>②Traditional Chinese Internal Medicine (2007 edition)	NA	NA	I, 0<br>C, 0
Xie J et al. 2021[81]	20/20	25/15	I, 39.07±5.71<br>C, 38.92±5.62	I, 3.11±0.82y<br>C, 3.05±0.80y	AB/A	Standards for Diagnosis and Treatment of Zhuang Medicine Diseases (2006 edition)	NA	NA	NA
Jing RZ et al. 2021[82]	44/44	28/60	I, 69.27±14.14 (34~93)<br>C, 71.05±12.24 (45~92)	NA	FG/G	CCMD-3	NA	1 m	NA
Liu LL et al. 2022[83]	40/40	28/52	I, 51.10±10.65 (26~68)<br>C, 51.43±11.48 (28~67)	I, 16.38±12.05 (3~48)m<br>C, 11.18±6.83 (1~36)m	AHK/A	①DSM-4<br>②CDTE-TCM (2016 edition)	NA	NA	NA

Table A2. Baseline characteristics of included studies (continued).

Study ID	Sample size (I/C)	Gender (male /female)	Age (mean or range, years)	Duration (mean or range)	Regimen (I/C)	Diagnostic criteria	TCM syndrome type	Follow up	Adverse Events
Gao M et al. 2022[84]	50/50	45/55	I, 51.9±3.4 (26~73) C, 53.4±3.7 (26~73)	I, 17.3±2.3 (2~40)m C, 18.7±2.1 (2~40)m	KL/K	NA	NA	NA	I, 3 (dizziness 1, fatigue 1, pain 1) C, 10 (excitation 2, dizziness 3, fatigue 2, headache 3)
Xu XY et al. 2022[85]	32/31	29/34	I, 44±11 (18~62) C, 43±11 (19~61)	I, 14.4±4.9 (5~26)m C, 14.2±5.7 (3~27)m	AH/A	CCMD-3	NA	NA	I, 1 (acupuncture-induced haematoma 1) C, 2 (acupuncture-induced haematoma 2)
Xie QL 2022[86]	30/30	24/36	I, 42.45±12.47 C, 42.26±12.53	NA	DLM/D	NA	NA	NA	NA

Table A2. Baseline characteristics of included studies (continued).

Study ID	Sample size (I/C)	Gender (male /female)	Age (mean or range, years)	Duration (mean or range)	Regimen (I/C)	Diagnostic criteria	TCM syndrome type	Follow up	Adverse Events
Wang YH et al. 2023[87]	72/72	NA	I, 48.1±6.5 (36~65) C, 47.3±6.4 (35~65)	I, 3.8±0.5 (0.5~7.9)y C, 3.7±0.4 (0.5~7.8)y	AL/A	①DSM-4 ②Fundamentals and Clinical Treatment of Sleep Disorders with Integrated Traditional Chinese and Western Medicine (2011 edition)	NA	NA	NA
Zhao JJ et al. 2023[88]	30/30	27/32	I, 39.50±11.93 (20~61) C, 39.40±11.79 (20~60)	I, 10.93±2.81m C, 10.27±2.53m	AB/A	Guideline for the evaluation and treatment of insomnia in Chinese adults (2017 edition)	NA	1m	NA
Yang WQ 2023[89]	32/31	42/21	I, 62.18±2.52 (55~70) C, 61.17±2.25 (56~69)	I, 5.26±3.45 (3~15)y C, 5.32±3.56 (2~16)y	BF/B	NA	NA	NA	NA
Jiang L 2023[90]	21/21	25/17	I, 45.25±2.15 (25~70) C, 45.29±2.17 (26~71)	I, 15.23±2.12 (2~20)m C, 15.77±2.17 (3~22)m	AF/A	NA	NA	NA	NA

Table A2. Baseline characteristics of included studies (continued).

Study ID	Sample size (I/C)	Gender (male /female)	Age (mean or range, years)	Duration (mean or range)	Regimen (I/C)	Diagnostic criteria	TCM syndrome type	Follow up	Adverse Events
Wang SQ 2023[91]	29/29	23/35	I, 47.69±10.07<br>C, 15.17±11.30	I, 21.15±7.57m<br>C, 19.30±10.17 m	F/A	①CCMD-3<br>②Guidelines for Clinical Research of New Chinese Medicines (2002 edition)	NA	1m	NA
Liu DL et al. 2024[92]	40/40	35/45	I, 38.69±5.93 (19~66)<br>C, 38.97±5.78 (20~65)	>3m	AE/A	NA	NA	NA	NA
Fan J et al. 2024[93]	100/100	112/88	I, 55.90±3.49 (44~64)<br>C, 54.20±3.70 (43~65)	I, 5.79±1.71 (1.5~9.1)m<br>C, 5.64±1.62 (1.4~8.7)m	DL/L	Expert consensus on the diagnosis and treatment of insomnia with depression and anxiety in Chinese adult (2020 edition)	NA	NA	NA
Liu YY et al. 2024[94]	30/29	27/32	I, 47.08±12.28 (23~65)<br>C, 42.90±12.48 (22~68)	I, 36.80±26.56 (2~108)m<br>C, 34.93±25.74 (2~120)m	AH/A	①ICSD-3<br>②Guideline for the evaluation and treatment of insomnia in Chinese adults (2017 edition)	NA	NA	NA

Table A2. Baseline characteristics of included studies (continued).

Study ID	Sample size (I/C)	Gender (male /female)	Age (mean or range, years)	Duration (mean or range)	Regimen (I/C)	Diagnostic criteria	TCM syndrome type	Follow up	Adverse Events
Zhuang J et al. 2024[95]	30/30	34/26	I, 46.50±10.45 (22~65)<br>C, 47.53±10.93 (21~64)	>1m	BM/M	①CCMD-3<br>②CDTE-TCM (2012 edition)	NA	1m	NA

[1] Tang T, Wang JL, Gao JX. Observations on the efficacy of shallow puncture plus cupping for treating 30 insomnia patients. Shanghai J Acupunct Moxibustion. 2006;25(11):10–11. (In Chinese)

[2] Xing QJ. Clinical study on acupuncture of defensive Qi adjustment and brain invigoration for treatment of insomnia [dissertation]. Zhengzhou: Henan University of Chinese Medicine; 2007. (In Chinese)

[3] Wang YM, Wu HY, Huang GQ. Observation on therapeutic effect of acupuncture plus moxibustion for insomnia. J Acupunct Tuina Sci. 2007;5(5):281–283. (In Chinese)

[4] Yang L, Lai XS, Feng SL. Clinical observation on 63 cases of insomnia treated with bloodletting therapy combined with electroacupuncture. New Chin Med. 2008;40(1):62–63. (In Chinese)

[5] Xia Y, Gao JX. Treatment of 30 cases of insomnia with moxibustion mainly at Baihui (GV20). J Clin Acupunct Moxibustion. 2008;24(11):23–24. (In Chinese)

[6] Gao XY, Wang XM, Wei YL, Shao SJ, Li XR, Zhang HJ, et al. Multicenter clinical study on regulating defensive Qi and invigorating brain acupuncture therapy for insomnia. In: Proceedings of the Symposium on Standardized Research Approaches in Acupuncture Diagnosis and Treatment; 2008. p. 165–168. (In Chinese)

[7] Liang YL, Lu LQ, Yang XQ, Liang JL. Nursing observation of bloodletting and cupping combined with acupuncture for insomnia. Nurs Pract Res. 2009;6(6):79–80. (In Chinese)

[8] Li ZP, Yan XY, Zhu XY. Clinical study on acupoint catgut embedding therapy for insomnia. Liaoning J Tradit Chin Med. 2010;37(10):2020–2022. (In Chinese)

[9] Chen FM. Clinical observation of bloodletting therapy combined with acupuncture for simple insomnia [dissertation]. Guangzhou: Guangzhou University of Chinese Medicine; 2010. (In Chinese)

[10] Shi YJ, Li HJ, Zhang HF. Clinical observation on catgut embedding at scalp acupoints for treating insomnia. Shanghai J Acupunct Moxibustion. 2011;30(11):738–740. (In

Chinese)

[11] Lian FC, Peng LH. Clinical observation of ear apex bloodletting combined with cupping along Back-Shu points for the treatment of 78 cases of insomnia. Chin Manip Rehabil Med. 2011;2(9):194–195. (In Chinese)

[12] Gu RX, Jiao Y, Xu DD. Clinical study on insomnia treated by catgut embedding applied to Shu-Mu acupoints jointly. Shanghai J Acupunct Moxibustion. 2011;30(2):101–103. (In Chinese)

[13] Li ZP, Zhu XY, Yan XY. Therapeutic observation on improving efficacy of moxibustion at Baihui for insomnia. World J Acupunct Moxibustion. 2011;21(1):25–29. (In Chinese)

[14] Li ZP, Zhou X, Liang ZH, Zhu XP, Fu WB. Efficacy analysis of acupuncture combined with thread embedding at acupoints for insomnia. New Traditional Chinese Medicine. 2011;43(11):84–86. (In Chinese)

[15] Shi L, Zhang QX, Zhang JL, Xie LQ, Chen J. Clinical study on gastrodin acupoint injection for treating insomnia. Chinese Journal of Geriatric Health Care. 2011;9(3):41–43. (In Chinese)

[16] Feng X, Yin JJ. Observation on the therapeutic effect of acupuncture combined with acupoint injection for insomnia. Chinese and Foreign Medical Research. 2012;10(29):116-116. (In Chinese)

[17] Li ZM, Wang W, Zhang Y. Controlled clinical observation of Treating Insomnia by Acupuncture Combined with Massage. Practical Journal of Chinese Internal Medicine. 2012;26(4):68-69. (In Chinese)

[18] Shi MW. Penetrating needling therapy on insomnia treated with acupuncture curative effect research [dissertation]. Henan University of Chinese Medicine; 2012. (In Chinese)

[19] Qiu FX, Yang XM. Observation on effect of acupuncture in coordination to point application to treat insomnia patients. Chin Gen Pract Nurs. 2012;10(1):10-11. (In Chinese)

[20] Lin YH, Chen DS. Clinical observation of ear acupuncture combined with needling for the treatment of insomnia. In: Proceedings of the 14th National Academic Seminar on Auricular Acupuncture and the Establishment of the Auricular Acupuncture Committee of the Guangdong Acupuncture Association; 2012; Guangdong. p. 112-113. (In Chinese)

[21] Jian H. Clinical observation of therapeutic efficacy of electric acupuncture and pressing seeds on ear acupoints for insomnia [D]. Guangzhou: Guangzhou University of Chinese Medicine; 2012. (In Chinese)

[22] Yao HF, Zhang HF, Chen XL. Observation on therapeutic effect of scalp-acupoint catgut embedding for 33 cases of insomnia patients. Acupunct Res. 2012;37(5):394-397. (In Chinese)

[23] Gao XY, Xu CX, Wang PY, Ren S, Zhou YL, Yang XG, et al. Curative effect of acupuncture and moxibustion on insomnia: A randomized clinical trial. J Tradit Chin Med. 2013;33(4):428-432.

[24] Zhang Y, Li LD, Qu J. Clinical observation on the treatment of insomnia with the "three-in-one" brain balance therapy. Clin J Tradit Chin Med. 2013;25(3):223-224. (In Chinese)

[25] Zhang HF, Yao HF, Zhou SJ. Effect of head acupoints catgut embedding on Pittsburgh sleep quality index in patients with insomnia. Chin Arch Tradit Chin Med. 2013;31(11):2420-2422. (In Chinese)

[26] Guo AS, Li AH, Feng LF, Chen X, Sun L. Clinical study of acupoint catgut-embedding therapy on patients with insomnia. J Nanjing Univ Tradit Chin Med. 2013;29(4):331-334. (In Chinese)

[27] Cao JK. Clinical observation of acupuncturing the area of spiritual emotion to treat primary insomnia. Guangzhou University of Chinese Medicine; 2013. (In Chinese)

[28] Xiong QJ, Zeng J. Clinical observation on electroacupuncture combined with acupoint pressure for the treatment of insomnia. Modern Diagnosis and Treatment. 2013;24(1):66-67. (In Chinese)

[29] Wei Y, Tang HQ, Li KM, Li XH. Observation on the therapeutic efficacy of insomnia treated with medicated thread moxibustion of Zhuang medicine plus acupuncture. Shanghai J Acupunct Moxibustion. 2013;32(11):906-907. (In Chinese)

[30] Gu TT, Zhong WQ. Clinical observation on effect of warm needling therapy in back-Shu points combined with auricular therapy in treating 30 cases of insomnia. World Chin Med. 2013;8(11):1342-1347. (In Chinese)

[31] Sun YZ, Yu T. Clinical research on the first area of Sun's abdominal acupuncture treatment for insomnia. J Clin Acupunct Moxibustion. 2014;30(12):42-44. (In Chinese)

[32] Feng SS, Zhou HF. Clinical observation on auricular point pressing seeds treatment of insomnia. J Liaoning Univ Tradit Chin Med. 2014;16(9):177-179. (In Chinese)

[33] Fu DM, Ke R. Acupuncturing at Shenmen point combined with stimulated auricular points for insomnia: clinical research on 60 cases. J Clin Acupunct Moxibustion.

2014;30(10):22-24. (In Chinese)

[34] Ma QY, Li Y, Cao LY, Zhang YY. Therapeutic observation of moxibustion at the governor vessel plus auricular point sticking for insomnia. Shanghai J Acupunct Moxibustion. 2014;33(7):624-626. (In Chinese)

[35] Wang YQ, Zhou JH, Chen YC. Clinical observation of Tujia San Shui acupoint therapy for insomnia. J Pract Tradit Chin Med. 2014;30(4):314. (In Chinese)

[36] Xie ZY, Wu X. Clinical observation on acupuncture point massage combined with auricular point pressing for insomnia in 30 cases. Hunan J Tradit Chin Med. 2015;31(8):80-81. (In Chinese)

[37] Xie ZY, Wu X, Hong J. Observation on the efficacy of acupoint massage plus moxibustion for refractory insomnia. J Acupunct Tuina Sci. 2015;13(1):44-48. (In Chinese)

[38] Zhao JP, Hong Y, Hong J. Clinical observation on acupuncture plus tuina for insomnia. J Acupunct Tuina Sci. 2015;13(4):232-235. (In Chinese)

[39] Liu ZL, Wang S. Clinical observation of thread embedding at tender points on the Governor vessel for intractable insomnia. Shanghai J Acupunct Moxibustion. 2015;34(12):1188-1189. (In Chinese)

[40] Xu LL, Xu FY. Therapeutic observation of auricular acupuncture with electroacupuncture and auricular acupressure for insomnia. Chin J Trauma Disabil Med. 2015;23(17):24-25. (In Chinese)

[41] Chen P. Clinical observation on 50 cases of insomnia treated with Du Meridian moxibustion combined with auricular point pressing therapy. Fam Psychol Doctor. 2015;11(3):1. (In Chinese)

[42] Huang G. Observation on the efficacy of acupuncture and acupressure combined therapy for sleep disorders. Fam Psychol Doctor. 2015. (In Chinese)

[43] Li BK. Study on clinical effect of acupuncture combined with ear point tapping in treatment of anhypnia. Chin Foreign Med Treat. 2016;36(10):158–159.

[44] Wang FY. Clinical observation on electro acupuncture of sleeping points in the treatment of insomnia. Guangming J Chin Med. 2016;31(16):2387–2389.

[45] Liu LN. Clinical observation of acupoint massage combined with auricular seed embedding for tranquilizing yang and nourishing yin in the treatment of insomnia. Dajia Jiankang (Mid-Month Edition). 2016;10(4):110. (In Chinese)

[46] Wu J, Yao ZJ, Ye P, et al. Effect of catgut embedding combined with ear acupoint sticking on PSQI in patients with insomnia. J Clin Acupunct. 2016;32(5):35–37. (In Chinese)

[47] Zhou LL. A study on the clinical efficacy of guiding fire to origin with moxibustion in combination with acupuncture in the treatment of primary insomnia and its impact on

patients' serum γ-aminobutyric acid [dissertation]. Fujian: Fujian University of Traditional Chinese Medicine; 2016. (In Chinese)

[48] Chang XL. Electric-acupuncture combined with auricular acupressure in the treatment of primary insomnia for 38 cases. Chin J Mod Distance Educ Chin Med. 2016;14(22):112–114. (In Chinese)

[49] Zhang SN, Ouyang LZ, Wang XZ, Zeng FK, Fang Q, Li JW, et al. Clinical observation on the efficacy of acupuncture combined with tuina for insomnia. In: Proceedings of the Annual Conference of Hunan Acupuncture Association; 2016. p. 93–95. (In Chinese)

[50] Pan XM, Zhang L, Yang Y, Duan YL, Liu YC, Zhang Y. Clinical observation on the efficacy of electroacupuncture in the treatment of moderate to severe insomnia. J Cardiovasc Dis Integr Tradit Chin West Med (Electron Ed). 2016;4(12):51–52,54. (In Chinese)

[51] Ning SY, Yang YH, Chu LL, Zhang B, Gao X, Dong SQ. Acupuncture combined with ear seed treatment for intractable insomnia. J Clin Acupunct Moxibustion. 2016;32(4):14–16. (In Chinese)

[52] Zhao YQ, Alatengqimuge. Acupuncture combined with Mongolian medicine warm needling in the treatment of 43 cases of insomnia. Food Health Care. 2016;3(12):93–94. (In Chinese)

[53] Ma WH, Zhou Y, Ma XL, Niu XL, Liu TZ. Study on the effect of acupuncture combined with moxibustion on insomnia. Jilin J Chin Med. 2017;37(11):1158–1160. (In Chinese)

[54] Hong YF, Du CH, Xu H, Ni WM, Qin LF. Clinical observation of needling Qin's eight head acupoints for insomnia. Shanghai J Acupunct Moxibustion. 2017;36(6):715–718. (In Chinese)

[55] Chen Y, Gao XY, Sun CY. Pricking and penetrating moxibustion therapy in patients with refractory insomnia: a randomized and controlled clinical trial. J Tradit Chin Med. 2018;38(5):754–762.

[56] Lu CL, Liu L, Liang Y, Pan XJ. Clinical observation on the treatment of insomnia with Youlong cupping combined with acupuncture in 34 cases. Yunnan J Tradit Chin Med Pharm. 2018;39(12):58–59. (In Chinese)

[57] Zhao JY, Ji CC. Clinical observation on the effect of scalp electroacupuncture on sleep quality of patients with primary insomnia. Mod Chin Med. 2018;38(3):14–17. (In Chinese)

[58] Lu WJ. The clinical observation on treating primary insomnia by regulating heel vessels (acupuncture) with beeding therapy [D]. North China University of Science and

Technology; 2018. (In Chinese)

[59] Yao JY. Clinical observation on primary insomnia treated by pricking collaterals and cupping combined with acupuncture [D]. Heilongjiang: Heilongjiang University of Chinese Medicine; 2019. (In Chinese)

[60] Li YT. Clinical observation on the therapeutic effect of eye acupuncture in treating insomnia [D]. Liaoning: Liaoning University of Traditional Chinese Medicine; 2019. (In Chinese)

[61] Hong QY, Yang HM, Zhao JP, Wang SG, Yao J, Yang Y. Acupuncture at Wuzangshu combined with Geshu acupoints with fire needles and filiform needles for chronic insomnia. Chinese General Practice. 2019;22(3):336-340. (In Chinese)

[62] Wu XQ, Gao J, Sun X, Wang YJ. Observation on the curative effect of acupuncture for purging the south, tonifying the north and tranquilizing the spirit with cupping and guasha on intractable insomnia. Modern Journal of Integrated Traditional Chinese and Western Medicine. 2019;28(15):1626-1629. (In Chinese)

[63] Yan QY. Effect of thunder fire moxibustion combined with the acupuncture treatment of meridian flow at specific time points for insomnia. Family Medicine. 2019;(11):390-391. (In Chinese)

[64] Zhou Y, Miao F, Qu Z, Gao J. Observation on therapeutic effect of acupuncture combined with medicine on primary insomnia. J Extern Ther Tradit Chinese Med. 2019;28(5):38-40. (In Chinese)

[65] Zhou YB, Yu XP. Auricular acupuncture combined with conventional acupuncture in the treatment of insomnia. Yishou Baodian. 2020;(8):129-178. (In Chinese)

[66] Wu HH. Clinical observation on the therapeutic effect of triple therapy in the treatment of 60 cases of insomnia. China Health Care and Nutrition. 2020;30(10):372. (In Chinese)

[67] Shi XD, Lu XG, Zhao M, Zhao J, Gao WL, Fan ZY, et al. Clinical study on the treatment of insomnia by Lu’s acupuncture plus Zhu’s thumb pushing manipulation. Chinese Manipulation and Rehabilitation Medicine. 2020;11(15):56-59. (In Chinese)

[68] Wu JL, Qi MH, Liang J, Yuan Z, Liu LY, Mei RJ, et al. Clinical observation of electroacupuncture at nape acupoints for primary insomnia. Shanghai Journal of Acupuncture and Moxibustion. 2020;39(2):153-157. (In Chinese)

[69] Li ZB, Huang W, Liao JM. Study on the therapeutic effect of Sun’s abdominal acupuncture in treating insomnia. Shenzhen Journal of Integrated Traditional Chinese and Western Medicine. 2020;30(2):9-11. (In Chinese)

[70] Cao WT. Discussion on the clinical effectiveness of eye acupuncture combined with wrist and ankle acupuncture in the treatment of insomnia. Electronic Journal of Clinical Medical Literature. 2020;7(88):53-54. (In Chinese)

[71] Li JY. Clinical observation of Lingnan fire acupuncture in treating insomnia. [Dissertation]. Guangdong: Guangzhou University of Chinese Medicine; 2020.

[72] Wang YP. Analysis of the clinical effect of three-part tui-na combined with traditional Chinese medicine acupoint selection and scraping therapy on insomnia. Yangsheng Baojian Zhinan. 2020(1):249. (In Chinese)

[73] Zhang XY, Tang Q, Tang HL, Wang C. Study on the efficacy difference between pestle acupuncture and electroacupuncture in the treatment of primary insomnia. Shizhen Guoyi Guoyao. 2020, 31(9):2166-2168. (In Chinese)

[74] Guo SX, Lu GL. Application of holographic scraping therapy in the treatment of insomnia. Henan Medical Research. 2020, 29(22):4165-4167. (In Chinese)

[75] Chen XM. Clinical efficacy and nursing experience of acupuncture combined with acupoint application in the treatment of insomnia. Yangsheng Baojian Zhinan. 2020(32):169-170. (In Chinese)

[76] Wei M. The clinical observation of TaiChi Tuina in the treatment of chronic insomnia and its effect on serum CRP and IL-6. [Dissertation]. Hubei: Hubei University of Traditional Chinese Medicine; 2020. (In Chinese)

[77] Ye YW, Fang YY. The effects of thunder fire moxibustion combined with meridian dredging on sleep status and inflammatory factors in patients with insomnia. Modern Medicine and Health Research (Electronic Edition). 2021;5(11):34-36. (In Chinese)

[78] Chen Y, Zhang MZ. Observe the clinical effect of acupotomology head acupuncture therapy on intractable insomnia and its effect on sleep quality of patients. World Journal of Sleep Medicine. 2021;8(4):618-619. (In Chinese)

[79] Cao WQ, Zhao M. Clinical efficacy evaluation of fire needle on five zangshu plus diaphragmatic shu combined with filiform acupuncture in the treatment of chronic insomnia. Health Women. 2021;(36):92, 94. (In Chinese)

[80] Lan L, Zhu LL, Nong YP, Chen SY, Liao WY, Mo QM, et al. Clinical study on twelve terrestrial branch hologram needle inserting umbilical acupuncture combined with auricular acupoint pressing magnetic bead in the treatment of insomnia. Guangming Journal of Chinese Medicine. 2021;36(14):2385-2387. (In Chinese)

[81] Xie J, Huang XJ. Observation on the nursing effect of Zhuang medicine line-point moxibustion in the treatment of Nian Lao Nuo (insomnia) syndrome. Health Care Journal. 2021;22(10):120-121. (In Chinese)

[82] Jing RZ, Liu WY, Chen LJ, Yi ZH, Jin YZ, Chen JD. Clinical study on intradermal needle-embedding combined with point application with Fructus Evodiae for insomnia. New Chinese Medicine. 2021;53(22):167-170. (In Chinese)

[83] Liu LL, Shi L. Clinical observation on scalp acupuncture combined with Zhongwan (RN12) and Yinjiao (RN7) electroacupuncture in the treatment of intractable insomnia. Chinese Medicine Modern Distance Education of China. 2022;20(9):122-124. (In Chinese)

[84] Gao M, Gu YM, Wu QX. Observation of therapeutic effect of electroacupuncture combined with auricular point sticking on insomnia. Henan Traditional Chinese Medicine. 2022;42(5):777-780. (In Chinese)

[85] Xu XY, Ma JH, Ou JX, Gan HX, Zhou DJ, Yang ZH, et al. Effect of Fang's scalp acupuncture on perceived stress and sleep structure in insomnia patients: a randomized controlled trial. Zhongguo Zhen Jiu. 2022;42(04):371-376. (In Chinese)

[86] Xie QL. Effect of ear scraping combined with ear acupoint pressure on improving insomnia: a clinical observation. Zhongwen Keji Qikan Database (Citation Edition) Medical and Health, 2022(12):289-292. (In Chinese)

[87] Wang YH, Zhao M, Yang GF, Li FQ, Liu JH, Liu XZ, et al. Acupuncture combined with auricular acupoint pressing beans for primary insomnia. Acta Chinese Medicine, 2023, 38(9):1992-1998. (In Chinese)

[88] Zhao JJ, Huang RL, Li TT. Clinical observation of acupuncture combined with Du meridian moxibustion in the treatment of chronic insomnia. Shanxi Journal of Traditional Chinese Medicine, 2023, 39(9):39-40. (In Chinese)

[89] Yang WQ. Clinical efficacy analysis of treatment of insomnia with acupuncture at key and symptomatic points combined with embedded needle therapy. Chinese Science and Technology Periodicals Database (Full-text Edition) Medicine and Health, 2023(10):117-120. (In Chinese)

[90] Jiang L. Efficacy analysis of conventional acupuncture combined with press-needle therapy in the treatment of primary insomnia. Chinese Science and Technology Periodicals Database (Full-text Edition) Medicine and Health, 2023(6):156-158. (In Chinese)

[91] Wang SQ. The Study of Fu's Subcutaneous Needling Therapy in the Treatment of Insomnia [D]. Guangdong: Guangzhou University of Chinese Medicine, 2023. (In Chinese)

[92] Liu DL, Wei QL, Liu M. Clinical analysis of acupuncture combined with bloodletting and cupping in the treatment of insomnia. Inner Mongolia Journal of Traditional Chinese Medicine. 2024;43(6):132–134. (In Chinese)

[93] Fan J, Liu ZY. Effects of ear holographic copper needle scraping combined with ear pressure beans in treatment of insomnia patients. Med J Chin People's Health. 2024;36(11):98–101. (In Chinese)

[94] Liu YY, An Q, An JM, Liu YF, Ma KJ, Zhao ZT. Effect of Fang's scalp acupuncture combined with Nazi method of midnight-noon ebb-flow on serum GABA and Glu and sleep quality in patients with insomnia. J Clin Acupunct Moxibustion. 2024;40(6):43–47. (In Chinese)

[95] Zhuang J, Luo PR, Li YF, Zhou C, Xiong J, Zhang N, et al. Clinical effect of head meridian manipulation combined with thermosensitive moxibustion at acupoints for insomnia. Pract Clin Integr Tradit Chin West Med. 2024;24(3):18–21. (In Chinese)

Table A3. Baseline characteristics of included studies.

Study ID	Acupoints selection			Intervention details	Control details	Outcomes
	Variation in acupoints	Intervention acupoints	Control acupoints			
Tang T et al. 2006[1]	I, fixed<br>C, semi-standardized	Acupuncture points (GV14, BL15, BL18, BL20, BL13, BL23, BL19, BL22)	Ear points (TF_4, CO_{15}, AT2,3,4i)	≤ 6 min/session	Pressing 2-3 times/day, 3-5 min/session, change every 2 days, 2 weeks/course.	①Total effective rate
Xing QJ 2007[2]	Semi-standardized	①Acupuncture points (GV20, GV14, BL62, KI6)<br>②Ear points (AT2,3,4i, TF_4)	Acupuncture points (EX-HN1, HT7, SP6)	A: 30 min/session, 1 manipulation/10 min, 1 session/day, 7 days/course for 2 courses.<br>L: pressing 3 times/day, 24 h/session, 1 session/3 days for 4 sessions.	30 min/session, 1 manipulation/10 min, 1 session/day,7 days/course for 2 courses.	①Total effective rate<br>②PSQI
Wang YM et al. 2007[3]	Semi-standardized	Acupuncture points (GV20, HT7, SP6)	Acupuncture points (GV20, HT7, SP6, GV24+, Ex-HN22, BL62, KI6)	15-20 min/session, 1 session/day, 10 sessions/course for 3 courses.	30-60 min/session, 1 session/day, 10 sessions/course for 3 courses.	①Total effective rate<br>②PSQI
Yang L et al. 2008[4]	Semi-standardized	Acupuncture points (EX-HN1, HT7, PC6, SP6, KI3, BL62, KI6, GV20, GV14)	Acupuncture points (EX-HN1, HT7, PC6, SP6, KI3, BL62, KI6)	K: 30 min/session, 1 session/2 days, 5 sessions/course for 3 courses (continuous dense wave)<br>E: 1 session/2 days, bloodletting 1-5ml/point, 5 sessions/course for 3 courses	30 min/session, 1 session/2 days, 5 sessions/course for 3 courses (continuous dense wave)	①Total effective rate<br>②PSQI

Table A3. Baseline characteristics of included studies.

Study ID	Acupoints selection			Intervention details	Control details	Outcomes
	Variation in acupoints	Intervention acupoints	Control acupoints			
Xia Y et al. 2008[5]	Semi-standardized	Acupuncture points (GV20, EX-HN1, Ex-HN22, PC6, HT7, SP6)	Acupuncture points (GV20, EX-HN1, Ex-HN22, PC6, HT7, SP6)	A: 30 min/session, 1-2 manipulations/session, 1 session/day,10 days/course for 2 courses.<br>B: 10-15 min/session, 1 session/day,10 days/course for 2 courses.	30 min/session, 1-2 manipulations/session, 1 session/day,10 days/course for 2 courses.	①Total effective rate
Gao XY et al. 2008[6]	Semi-standardized	①Acupuncture points (GV20, GV14, BL62, KI6)<br>②Ear points ($AT_{2,3,4i}$, TF_4)	Acupuncture points (EX-HN1, HT7, SP6)	A: 30 min/session, 1 manipulation/10 min, 1 session/day, 15 days/course for 1 course.<br>L: pressing 2 times/day, 10-15 min/session, 1 session/3 days, 15 days/course for 1 course.	30 min/session, 1 manipulation/10 min, 1 session/day, 15 days/course for 1 course.	①Total effective rate<br>②PSQI
Liang YL et al. 2009[7]	Fixed	Acupuncture points (GV14, BL15, BL18, EX-HN1, GV20, PC6, HT7, ST36, SP6, KI1)	Acupuncture points (EX-HN1, GV20, PC6, HT7, ST36, SP6, KI1)	A: 30 min/session, 1 session/day, 14 days/course for 2 courses.<br>E: 10-15 min/session, bloodletting 2-5ml/point, 2 sessions/week, 4 sessions/course for 2 courses.	30 min/session, 1 session/day, 14 days/course for 2 courses.	①Total effective rate
Li ZP et al. 2010[8]	Fixed	Acupuncture points (GV20, Ex-HN22, HT7, PC6, ST36, SP6, LR3, BL62, KI6, BL15, BL18, BL20, BL23)	Acupuncture points (GV20, Ex-HN22, HT7, PC6, ST36, SP6, LR3, BL62, KI6)	A: 30 min/session, 5 sessions/week, 6 weeks/course for 1 course.<br>G: 1 session/2 weeks, 6 weeks/course for 1 course.	30 min/session, 5 sessions/week, 6 weeks/course for 1 course.	①Total effective rate<br>②PSQI

Table A3. Baseline characteristics of included studies (continued).

Study ID	Acupoints selection			Intervention details	Control details	Outcomes
	Variation in acupoints	Intervention acupoints	Control acupoints			
Chen FM 2010[9]	Semi-standardized	Acupuncture points (GV20, GV14, EX-HN6, HT7, PC6, Ex-HN22)	Acupuncture points (HT7, PC6, GV20, Ex-HN22)	A: 30 min/session, 1 manipulation/10-15 min, 1 session/2 days, 6 sessions/course for 2 courses. E: Bloodletting 1-5ml/point, 1 session/2 days, 6 sessions/course for 2 courses.	30 min/session, 1 manipulation/10-15 min, 1 session/2 days, 6 sessions/course for 2 courses.	①Total effective rate ②AIS
Shi YJ et al. 2011[10]	Fixed	Acupuncture points (GV20, GV24, GB20)	Acupuncture points (GV20, GV24, GB20, HT7, PC6, SP6)	1 session/week, 4 weeks/course for 1 course	30 min/session, 3 sessions/week, 4 weeks/course for 1 course.	①PSQI
Lian FC et al. 2011[11]	Fixed	Acupuncture points (EX-HN6)	Acupuncture points (HT7, PC6, PC7, BL15, SP6,LR3)	Bloodletting 20 drops/point, 1 session/2 days, 12 sessions/course for 3 courses.	30 min/session, 1 session/day, 12 sessions/course for 3 courses.	①Total effective rate
Gu RX et al. 2011[12]	Semi-standardized	Acupuncture points (BL15, CV14)	Acupuncture points (HT7, PC6, GV20, Ex-HN22)	1 session/15 days, 2 sessions/course for 2 courses.	30 min/session, 1 session/2 day, 15 sessions/course for 2 courses.	①Total effective rate ②PSQI
Li ZP et al. 2011a[13]	Fixed	Acupuncture points (GV20, Ex-HN22, HT7, PC6, ST36, SP6, LR3, BL62, KI6)	Acupuncture points (GV20, Ex-HN22, HT7, PC6, ST36, SP6, LR3, BL62, KI6)	A: 30 min/session, 1 session/day for 5 days, 4 weeks/course for 1 course. B: 5-10min/session, 1 session/day for 5 days, 4 weeks/course for 1 course.	30 min/session, 1 session/day for 5 days, 4 weeks/course for 1 course.	①Total effective rate ②PSQI

Table A3. Baseline characteristics of included studies (continued).

Study ID	Acupoints selection			Intervention details	Control details	Outcomes
	Variation in acupoints	Intervention acupoints	Control acupoints			
Li ZP et al. 2011b[14]	Fixed	Acupuncture points (GV20, Ex-HN22, HT7, PC6, ST36, SP6, LR3, BL15, BL18, BL20, BL23)	Acupuncture points (GV20, Ex-HN22, HT7, PC6, ST36, SP6, LR3)	A: 30 min/session, 5 sessions/week, 2 weeks/course for 4 courses.<br>G: 1 session/2 weeks	30 min/session, 5 sessions/week, 2 weeks/course for 4 courses.	①Total effective rate<br>②PSQI
Shi L et al. 2011[15]	I, fixed<br>C, semi-standardized	Acupuncture points (ST36, SP6)	Acupuncture points (KI6, BL62, HT7, GV24+, EX-HN1, Ex-HN22)	1 session/day, 10 sessions/course for 2 courses.	1 session/day, 10 sessions/course for 2 courses.	①Total effective rate<br>②PSQI
Feng X et al. 2012[16]	Semi-standardized	Acupuncture points (GV20, EX-HN1, Ex-HN22, HT7, SP6)	Acupuncture points (GV20, EX-HN1, Ex-HN22, HT7, SP6)	A: 40 min/session, 1 session/day, 15 sessions/course<br>G: 1 session/day, 15 sessions/course	40 min/session, 1 session/day, 15 sessions/course	①Total effective rate
Li ZM et al. 2012[17]	Semi-standardized	Acupuncture points (EX-HN1, GV20, HT7, SP6)	Acupuncture points (EX-HN1, GV20, HT7, SP6)	A: 30 min/session, 2-3 manipulations/session, 1 session/2 days, 10 sessions/course for 2 courses.<br>M: 1 session/2 days, 10 sessions/course for 2 courses.	30 min/session, 2-3 manipulations/session, 1 session/2 days, 10 sessions/course for 2 courses.	①Total effective rate

Table A3. Baseline characteristics of included studies (continued).

Study ID	Acupoints selection			Intervention details	Control details	Outcomes
	Variation in acupoints	Intervention acupoints	Control acupoints			
Shi MW 2012[18]	Fixed	Acupuncture points (BL13, BL15, BL20, BL18, BL23)	Acupuncture points (BL13, BL15, BL20, BL18, BL23)	40 min/session, 1 session/day, 5 sessions/week, 3 weeks/course for 1 course.	40 min/session, 1 session/day, 5 sessions/week, 3 weeks/course for 1 course.	①Total effective rate ②PSQI ③SAS ④SDS ⑤Traditional Chinese Medicine Symptom Score Scale
Qiu FX et al. 2012[19]	Semi-standardized	Acupuncture points (EX-HN1, PC6, HT7, Ex-HN22, ST36, SP6, KI1)	Acupuncture points (EX-HN1, PC6, HT7, Ex-HN22, ST36, SP6)	A: 30 min/session, 1 session/day, 7 days/course for 4 courses. G: 1 session/day, 7 days/course for 4 courses.	30 min/session, 1 session/day, 7 days/course for 4 courses.	①Total effective rate
Lin YH et al. 2012[20]	Semi-standardized	①Acupuncture points (PC6, HT7, GV20, SP6) ②Ear points (TF_4, AT_4, CO_{15}, CO_{12}, CO_{13}, CO_{10}, AT_3, CO_{18})	Acupuncture points (PC6, HT7, GV20, SP6)	A: 30 min/session, 1 session/day, 10 sessions/course L: pressing 5-7 times/day, 3 min/session, 1 session/2 days, 5 sessions/course.	30 min/session, 1 session/day, 10 sessions/course	①Total effective rate ②SPIEGEL

Table A3. Baseline characteristics of included studies (continued).

Study ID	Acupoints selection			Intervention details	Control details	Outcomes
	Variation in acupoints	Intervention acupoints	Control acupoints			
Jian HL 2012[21]	Semi-standardized	①Acupuncture points (GV20, Ex-HN22, HT7, PC6, ST36, SP6, LR3, BL62, KI6) ②Ear points (CO_{15}, TF_4, AT_4)	Acupuncture points (GV20, Ex-HN22, HT7, PC6, ST36, SP6, LR3, BL62, KI6)	K: 30 min/session, 1 session/ 2 days, 3 sessions/week, 4 weeks/course (continuous wave, 80/minute) L: pressing 5-6 times/day, 2-3 min/session, pressing 1 time 30 min before bedtime, 1 session/2 days, 3 sessions/week, 4 weeks/course.	30 min/session, 1 session/ 2 days, 3 sessions/week, 4 weeks/course (continuous wave, 80/minute)	①Total effective rate ②PSQI ③SPIEGEL
Yao HF et al. 2012[22]	Fixed	Acupuncture points (GV20, GV24, GB20)	Acupuncture points (GV20, GV24, GB20, HT7, PC6, SP6)	1 session/week, 4 weeks/course for 1 course.	30 min/session, 3 sessions/week, 4 weeks/course for 1 course.	①PSQI
Gao XY et al. 2013[23]	Semi-standardized	Acupuncture points (GV20, EX-HN1, BL62, KI6)	Acupuncture points (HT7, PC6, SP6)	A: 1 session/day, 1 manipulation/10 min, 15 days/course B: 40 min/session	1 session/day, 1 manipulation/10 min, 15 days/course	①Total effective rate ②PSQI
Zhang Y et al. 2013[24]	I, fixed C, Semi-standardized	Acupuncture points (EX-HN1, Rutu, PC6, KI1)	Acupuncture points (EX-HN1)	30 min/session, 1 session/day, 12 days/course for 2 courses	30 min/session, 1 session/day, 12 days/course for 2 courses. (continuous wave, 6-8Hz)	①Total effective rate ②PSQI
Zhang HF et al. 2013[25]	Fixed	Acupuncture points (GV20, GV24, GB20)	Acupuncture points (GV20, GV24, GB20, HT7, PC6, SP6)	1 session/week, 4 weeks/course for 1 course	30 min/session, 3 sessions/week, 4 weeks/course for 1 course	①PSQI

Table A3. Baseline characteristics of included studies (continued).

Study ID	Acupoints selection			Intervention details	Control details	Outcomes
	Variation in acupoints	Intervention acupoints	Control acupoints			
Guo AS et al. 2013[26]	Semi-standardized	Acupuncture points (HT7, SP6, GV20, Ex-HN22)	Acupuncture points (HT7, SP6, GV20, Ex-HN22)	1 session/ 2 weeks for 3 sessions	30 min/session, 1 manipulation/10 min, 1 session/day, 5 sessions/week for 6 weeks	①Total effective rate ②PSQI ③IL-1β ④TNF-α
Cao JK 2013[27]	Fixed	①Scalp acupuncture ②Acupuncture points (GV20, HT7, Ex-HN22, PC6)	Acupuncture points (GV20, HT7, Ex-HN22, PC6)	1 session/day, 10 sessions/course, a total of 10 days for 1st course; 1 session/2 days, 3 sessions/week, 10 sessions/course for 2nd course	1 session/day, 10 sessions/course, a total of 10 days for 1st course; 1 session/2 days, 3 sessions/week, 10 sessions/course for 2nd course	①Total effective rate ②PSQI
Xiong QJ et al. 2013[28]	Semi-standardized	Acupuncture points (HT7, SP6, PC6, GV20, EX-HN1, GB20)	Acupuncture points (HT7, SP6, PC6, GV20, EX-HN1)	K: 30 min/session, 1 session/day, 10 days/course for 20 days<br>M: pressing 5-6 times/point, 15 s/point for 20 days	30 min/session, 1 session/day, 10 days/course for 20 days	①Total effective rate
Wei Y et al. 2013[29]	Fixed	Acupuncture points (ST36)	Acupuncture points (SP6, EX-HN5, PC6, LR3, GV24, KI6, BL62)	A: 30 min/session, 1 manipulation/10 min, 1 session/day, 10 sessions/course for 2 courses<br>B: 8 moxibustion cones, 1 session/day, 10 sessions/course for 2 courses	30 min/session, 1 manipulation/10 min, 1 session/day, 10 sessions/course for 2 courses	①Total effective rate ②PSQI

Table A3. Baseline characteristics of included studies (continued).

Study ID	Acupoints selection			Intervention details	Control details	Outcomes
	Variation in acupoints	Intervention acupoints	Control acupoints			
Gu TT et al. 2013[30]	I, semi-standardized<br>C, fixed	①Acupuncture points (BL13, BL15, BL18, BL20, BL23, GB20, Ex-HN22, EX-HN5)<br>②Ear points (TF_4, AH_{6a}, AT_4)	①Acupuncture points (BL13, BL15, BL18, BL20, BL23, GB20, Ex-HN22, EX-HN5)<br>②Ear points (TF_4, AH_{6a}, AT_4)	B: 1 moxibustion cone/point, 1 session/2 days, 9 sessions/course for 2 courses<br>L: pressing 3-4 times/day, 3-5min/session, pressing 1 time 30 min before bedtime, change every 4 days, 1 month/course	1 session/2 days, 9 sessions/course for 2 courses	①Total effective rate<br>②PSQI
Sun YZ et al. 2014[31]	Semi-standardized	①Abdominal acupuncture<br>②Acupuncture points (GV24+, EX-HN1, Ex-HN22, HT7, BL62, KI6)	Acupuncture points (GV24+, EX-HN1, Ex-HN22, HT7, BL62, KI6)	30 min/session, 1 session/day, 10 sessions/course for 2 courses (continuous wave)	30 min/session, 1 session/day, 10 sessions/course for 2 courses (continuous wave)	①Total effective rate<br>②PSQI
Feng SS et al. 2014[32]	I, fixed<br>C, semi-standardized	Ear points	Acupuncture points (EX-HN1, GV20, GV24+, GV24, HT7, Ex-HN22)	for 4 weeks	30 min/session, 5 sessions/week for 4 weeks	①Total effective rate
Fu DM et al. 2014[33]	Semi-standardized	①Acupuncture points (HT7)<br>②Ear points (AH_{6a}, CO_{15}, CO_{13}, CO_{18}, CO_{10}, TF_4)	Acupuncture points (HT7)	A: 25 min/session, 1 session/day, 15 days/course for 6 courses<br>L: pressing 3 times/day, 15 sessions/course for 6 courses	25 min/session, 1 session/day, 15 days/course for 6 courses	①Total effective rate<br>②PSQI

Table A3. Baseline characteristics of included studies (continued).

Study ID	Acupoints selection			Intervention details	Control details	Outcomes
	Variation in acupoints	Intervention acupoints	Control acupoints			
Ma QY et al. 2014[34]	Fixed	①The Du meridian [From GV14 to GV3]<br>②Ear points (CO_{15}, TF_4, AH_{6a}, AT_4)<br>③Acupuncture points (Ex-HN22)	①Ear points (CO_{15}, TF_4, AH_{6a}, AT_4)<br>②Acupuncture points (Ex-HN22)	B: 20 min/session, 1 session/2 days, 10 sessions/course for 2 courses<br>L:pressing 3 times/day, change every 2 days, 10 sessions/course for 2 courses	Pressing 3 times/day, change every 2 days, 10 sessions/course for 2 courses	①Total effective rate<br>②PSQI
Wang YQ et al. 2014[35]	Fixed	Acupuncture points (BL2, GV24+, EX-HN5, GB20, GB21)	Acupuncture points (EX-HN1, EX-HN5)	1 session/day, 5 sessions/course for 3 courses	30 min/session, 1 session/day, 5 sessions/course for 3 courses (continuous wave + intermittent wave, 80Hz)	①Total effective rate
Xie ZY et al. 2015a[36]	I, semi-standardized<br>C, fixed	①Acupuncture points (EX-HN5, GV24+, GV24, GV23, GV20, GB20, GV16)<br>②Ear points (TF_4, AT_4, AH_{6a}, CO_{15}, TG_{2p})	Acupuncture points (EX-HN5, GV24+, GV24, GV23, GV20, GB20, GV16)	L: pressing 4-5 times/day, change every 3-5 days for 4 weeks<br>M: 20 min/session, 1 session/day for 4 weeks	20 min/session, 1 session/day for 4 weeks	①Total effective rate
Xie ZY et al. 2015b[37]	Fixed	Acupuncture points (BL2, EX-HN4, TE23, GV25, GV15, EX-HN5, GV24+, GV24, GV20, GB20, GV16, BL10, KI1)	Acupuncture points (BL2, EX-HN4, TE23, GV25, GV15, EX-HN5, GV24+, GV24, GV20, GB20, GV16, BL10)	20min/session, 1 session/day for 4 weeks	20min/session, 1 session/day for 4 weeks	①Total effective rate

Table A3. Baseline characteristics of included studies (continued).

Study ID	Acupoints selection			Intervention details	Control details	Outcomes
	Variation in acupoints	Intervention acupoints	Control acupoints			
Zhao JP et al. 2015[38]	Semi-standardized	Acupuncture points (HT7, SP6, EX-HN1, GV20, GV24+, EX-HN5, PC6, GB20, Ex-HN22, GB21, CV12, CV6, CV4, GV14)	Acupuncture points (HT7, SP6, EX-HN1, GV20, GV24+, EX-HN5, PC6, GB20, Ex-HN22)	30 min/session,1 manipulation/session, 1 session/day, 10 sessions/course for 3 courses	30 min/session,1 manipulation/session, 1 session/day, 10 sessions/course for 3 courses	①Total effective rate
Liu ZL et al. 2015[39]	Fixed	The Du meridian	Acupuncture points (HT7, PC6, SP6, KI3, ST36, SP9, BL62, KI6, EX-HN1)	1 session/14 days, 6 sessions for 3 month	30-40 min/session, 1 session/day, 6 sessions/week for 3 m	①Total effective rate ②PSQI
Xu LL et al. 2015[40]	Fixed	Ear points (TF_4, CO_{15}, CO_{12}, CO_{13}, CO_{10}, AT_4, CO_{18}, CO_{17})	Ear points (TF_4, CO_{15}, CO_{12}, CO_{13}, CO_{10}, AT_4, CO_{18}, CO_{17})	30 min/session, 1 session/day, change every 2 days, 10days/course for 3 courses (continuous wave, 2Hz)	Pressing at least 3 times/day, 20 times/point, change every 2 days, 10days/course for 3 courses	①Total effective rate
Chen P et al. 2015[41]	Fixed	①Ear points (CO_{15}, TF4, AH6a, AT_4) ②Ex-HN22 ③The Du meridian [From GV14 to GV3]	①Acupuncture points (Ex-HN22) ②Ear points (CO15, TF4, AH_{6a}, AT_4)	B: 20 min/session, 1 session/2 day, 10 sessions/course for 2 courses L: Pressing 3 times/day, change every 2 days, 10 days/course	Pressing 3 times/day, change every 2 days, 10 days/course	①Total effective rate

Table A3. Baseline characteristics of included studies (continued).

Study ID	Acupoints selection			Intervention details	Control details	Outcomes
	Variation in acupoints	Intervention acupoints	Control acupoints			
Huang G 2015[42]	Semi-standardized	Acupuncture points (GV20, EX-HN1, HT7, GV24+, Ex-HN22, KI6, BL62, EX-HN5, GB14, GB7, GB19, HT7, PC6, GB20, GB18, PC9, HT9, HT3, HT7, PC7, CV17, ST7, CV13, CV8, CV4, SP12, SP10, ST36, KI3, LR3, KI1, BL18, BL23, BL21, BL20)	Acupuncture points (GV20, EX-HN1, HT7, GV24+, Ex-HN22, KI6, BL62)	A: 40 min/session, 1 session/day for 2 weeks<br>M: for 2 weeks	40 min/session, 1 session/day for 2 weeks	①Total effective rate
Li BK 2016[43]	Semi-standardized	①Acupuncture points (HT7, GV20, PC6, PC7)<br>②Ear points (TF_4, CO_{15}, AT2,3,4i)	Acupuncture points (HT7, GV20, PC6, PC7)	A: 30 min/session, 1 session/2 days<br>L: Pressing 2 times/day	30 min/session, 1 session/2 days	①Total effective rate
Wang FY 2016[44]	Semi-standardized	Acupuncture points (Ex-HN22)	Acupuncture points (Ex-HN22)	30 min/session, 1 session/day, 5 sessions/week, 15 sessions/course for 2 courses (continuous wave)	30 min/session, 1 session/day, 5 sessions/week, 15 sessions/course for 2 courses	①Total effective rate
Liu LN 2016[45]	Fixed	①Acupuncture points (GV24+, EX-HN5, GV20, GB20, Ex-HN22, CV23, CV24)<br>②Ear points (CO_{15}, CO_{13}, CO_{10}, TF_4, CO_{18}, AH_{6a})	Ear points (CO_{15}, CO_{13}, CO_{10}, TF_4, CO_{18}, AH_{6a})	M: 2-3 manipulations, 30-50 times/manipulation, 120-160 times/ min, 1 session/day, 7 days/course<br>L: pressing 3-4 times/day, 30-60 times/session, change every 2-3 days	Pressing 3-4 times/day, 30-60 times/session, change every 2-3 days	①Total effective rate

Table A3. Baseline characteristics of included studies (continued).

Study ID	Acupoints selection			Intervention details	Control details	Outcomes
	Variation in acupoints	Intervention acupoints	Control acupoints			
Wu J et al. 2016[46]	Semi-standardized	Ear points (CO_{18}, CO_{15}, AH_{6a}, TF_4)	Acupuncture points (GV20, Ex-HN22, HT7, PC6, SP6, KI6, BL62, GV24+)	G: 1 session/2 weeks for 2 weeks<br>L: pressing 3times/day, 10 times/point, 4 days/session for 2 weeks	30 min/session, 3 sessions/week for 2 weeks	①Total effective rate<br>②PSQI
Zhou LL 2016[47]	Semi-standardized	Acupuncture points (KI1, GV24+, EX-HN1, Ex-HN22, HT7, KI6, BL62)	Acupuncture points (GV24+, EX-HN1, Ex-HN22, HT7, KI6, BL62)	A: 30 min/session, 1 manipulation/10 min, 1 session/2 days, 10 sessions/course for 2 courses<br>B: 30 min/session, 10 sessions/course for 2 courses	30 min/session, 1 manipulation/10 min, 1 session/2 days, 10 sessions/course for 2 courses	①Total effective rate<br>②PSQI<br>③GABA
Chang XL 2016[48]	Semi-standardized	①Acupuncture points (GV20, GV24, Ex-HN22, HT7, PC6)<br>②Ear points (TF_4, AT_4, AH_{6a}, CO_{18}, CO_{15}, CO_{13}, CO_{10}, AT_3)	Acupuncture points (GV20, GV24, Ex-HN22, HT7, PC6)	K: 30 min/session, 1 session/day, 7 days/course for 4 courses (continuous wave)<br>L: 5 times/day, 2 min/session	30 min/session, 1 session/day, 7 days/course for 4 courses (continuous wave)	①Total effective rate
Zhang SN et al. 2016[49]	Fixed	①Acupuncture points: (GV20, EX-HN1, Ex-HN22, GV23, GV24+, HT7, CV4, PC6, SP6, KI3, EX-HN5, GV24, ST8, GB14, BL1, GB20, BL15, BL18)<br>②The bladder meridian of foot-Taiyang	Acupuncture points (GV20, EX-HN1, Ex-HN22, GV23, GV24+, HT7, CV4, PC6, SP6, KI3)	A: 30 min/session, 1 manipulation/10 min, 1 session/days, 10 sessions/course for 3 courses<br>M: 1 session/days, 10 sessions/course for 3 courses	30 min/session, 1 manipulation/10 min, 1 session/days, 10 sessions/course for 3 courses	①Total effective rate<br>②PSQI

Table A3. Baseline characteristics of included studies (continued).

Study ID	Acupoints selection			Intervention details	Control details	Outcomes
	Variation in acupoints	Intervention acupoints	Control acupoints			
Pan XM et al. 2016[50]	Fixed	Acupuncture points (EX-HN1, GB20, GB20)	Acupuncture points (EX-HN1, GB20, GB20)	30 min/session, 1 session/days, 10 sessions/course for 3 courses (continuous wave)	30 min/session, 1 session/days, 10 sessions/course for 3 courses	①Total effective rate ②PSQI
Ning SY et al. 2016[51]	Semi-standardized	①Acupuncture points (GV20, Ex-HN22, EX-HN1, HT7, PC6, SP6, KI6, BL62) ②Ear points (CO_{15}, TF_4, AT_4)	Acupuncture points (GV20, Ex-HN22, EX-HN1, HT7, PC6, SP6, KI6, BL62)	A: 30 min/session, 1 manipulation/5 min, 1 session/2 days, 10 sessions/course for 3 courses L: pressing 3 times/day, 3-5min/session, change every 5 days, 2 sessions/course for 3 courses	30 min/session, 1 manipulation/5 min, 1 session/2 days, 10 sessions/course for 3 courses	①Total effective rate ②PSQI
Zhao YQ et al. 2016[52]	Fixed	Acupuncture points (GV20, EX-HN1, GV24, PC6, SP6, KI3)	Acupuncture points (GV20, EX-HN1, GV24, PC6, SP6, KI3)	1-2 moxibustion cones/session, 2 weeks/course for 1 course	30 min/session, 1 session/day, 2 weeks/course for 1 course	①Total effective rate
Ma WH et al. 2017[53]	Fixed	①Acupuncture points (GV24+, GV20, GV24, Ex-HN22, PC6, HT7, CV13, CV12, CV4, CV6, SP6) ②The Du meridian [From GV14 to BL54] ③The bladder meridian of foot-Taiyang	Acupuncture points (GV24+, GV20, GV24, Ex-HN22, PC6, HT7, CV13, CV12, CV4, CV6, SP6)	A: 30 min/session, 5 days/week for 4 courses B: 3 sessions/week for 4 courses	30 min/session, 5 days/week for 4 courses	①Total effective rate ②PSQI ③ESS ④AIS

Table A3. Baseline characteristics of included studies (continued).

Study ID	Acupoints selection			Intervention details	Control details	Outcomes
	Variation in acupoints	Intervention acupoints	Control acupoints			
Hong YF et al. 2017[54]	Fixed	Acupuncture points (GB20, GV20, GB15, GB8, GV24+)	Acupuncture points (Ex-HN22, HT7, KI6, BL62)	30 min/session, 3 sessions/week, 4 weeks/course for 2 courses	30 min/session, 3 sessions/week, 4 weeks/course for 2 courses	①Total effective rate ②SPIEGEL
Chen Y et al. 2018[55]	Fixed	Acupuncture points (BL15, BL17, BL18, BL20)	Acupuncture points (BL15, BL17, BL18, BL20)	B: 30 min/session, 1 session/day, 5 days/week, 7 days/course for 4 courses E: 10 min/session, bloodletting 2-5ml/point, 7 days/course, 1 session/course for 4 courses	30 min/session, 1 session/day, 5 days/week, 7 days/course for 4 courses	①Total effective rate ②PSQI ③Traditional Chinese Medicine Symptom Score Scale
Lu CL et al. 2018[56]	Fixed	①Acupuncture points (GV20, EX-HN1, GV26, GV24, EX-HN5, Ex-HN22, LI4, HT7,SP9, SP6, KI3, PC5, MS5, MS1) ②The Du meridian ③The bladder meridian of foot-Taiyang	Acupuncture points (GV20, EX-HN1, GV26, GV24, EX-HN5, Ex-HN22, LI4, HT7,SP9, SP6, KI3, PC5, MS5, MS1)	A: 30 min/session, 1 session/day, 15 days/course C: 1 session/day, 6 sessions/course, 15 days/course for 2 courses	30 min/session, 1 session/day, 15 days/course (intermittent wave)	①Total effective rate
Zhao JY et al. 2018[57]	Fixed	Acupuncture points (GV24+, GV24, GV20, EX-HN1)	Acupuncture points (HT7, SP6, ST36, LI4, LR3, KI6, BL62)	30 min/session, 3 sessions/week, 6 sessions/course for 4 courses (sparse wave)	30 min/session, 1 manipulation/15 min, 3 sessions/week, 6 sessions/course for 4 courses	①Total effective rate ②PSQI

Table A3. Baseline characteristics of included studies (continued).

Study ID	Acupoints selection			Intervention details	Control details	Outcomes
	Variation in acupoints	Intervention acupoints	Control acupoints			
Lu WJ 2018[58]	Fixed	Acupuncture points (BL62, KI6, BL1, EX-HN6)	Acupuncture points (EX-HN1, GV20, Ex-HN22, HT7, PC6, SP6, LR2)	A: 30-40 min/session, 1 session/day, 5 sessions/week, 2 weeks/course for 2 courses<br>E: Bloodletting 5-10 drops/point, 1 session/2 days, 3 sessions/week	30-40 min/session, 1 session/day, 5 sessions/week, 2 weeks/course for 2 courses	①Total effective rate<br>②PSQI<br>③AIS<br>④Traditional Chinese Medicine Symptom Score Scale
Yao YJ 2019[59]	Fixed	Acupuncture points (GV20, EX-HN1, ST8,EX-HN5, Ex-HN22, GV26, PC6, HT7, GV14)	Acupuncture points (GV20, EX-HN1, ST8,EX-HN5, Ex-HN22, GV26, PC6, HT7)	A: 50 min/session, 1 session/day, 2 weeks/course for 2 courses<br>E: Bloodletting 1-5 ml/point, 5-8 min/session, 1 session/day for 5 days, 2 weeks/course for 2 course	50 min/session, 1 session/day, 2 weeks/course for 2 courses	①Total effective rate<br>②PSQI
Li YT 2019[60]	Semi-standardized	Eye acupuncture	Acupuncture points (EX-HN1, GV24+, Ex-HN22, HT7)	24 h/session, 5 days/course for 2 courses	20 min/session, 1 session/day, 5 days/course for 2 courses	①Total effective rate<br>②PSQI
Hong QY et al. 2019[61]	Semi-standardized	Acupuncture points (BL18, BL15, BL13, BL20, BL23, BL17, GV20, EX-HN1, HT7, SP6)	Acupuncture points (GV20, EX-HN1, HT7, SP6)	A: 30 min/session, 1 session/day, 5 sessions/week for 4 weeks<br>F: 1 session/2 days, 3 sessions/week for 4 weeks	30 min/session, 1 session/day, 5 sessions/week for 4 weeks	①Total effective rate<br>②PSQI<br>③SAS<br>④SDS

Table A3. Baseline characteristics of included studies (continued).

Study ID	Acupoints selection			Intervention details	Control details	Outcomes
	Variation in acupoints	Intervention acupoints	Control acupoints			
Wu XQ et al. 2019[62]	Semi-standardized	①Acupuncture points (PC6, PC7, HT7, KI3, KI7, KI6, GV20, EX-HN1, GV24+, GV24, GB20, SP6, LR3, SP10, GV14, BL13, BL15, BL18, BL19, BL20, BL23, GV4) ②The Du meridian ③The bladder meridian of foot-Taiyang	Acupuncture points (EX-HN1, HT7, SP6)	A: 40 min/session, 3 sessions/week for 8 weeks C, D: 10 min/session, 1 session/week for 8 weeks	40 min/session, 3 sessions/week for 8 weeks	①Total effective rate ②PSQI
Yan QY 2019[63]	Semi-standardized	Acupuncture points (KI6, BL62, HT7, GV24+, EX-HN1, Ex-HN22)	Acupuncture points (KI6, BL62, HT7, GV24+, EX-HN1, Ex-HN22)	6 or 9 sessions/course	2 days/course for 22 days	①Total effective rate
Zhou Y et al. 2019[64]	Fixed	①Acupuncture points (ST36, SP6, Shimian, Ex-HN22,KI1, BL15, BL18, BL19, BL20, BL21, BL23, GV20, HT7, GV24+) ②Ear points (AH_{6a}, TF_4, AT_4, CO_{15}, CO_{12}, CO_{11}, CO_{13}, CO_4, CO_{10}, AT2,3,4i)	Acupuncture points (ST36, SP6, Ex-HN22, KI1)	F: pressing 4-5 times/day, 2 min/session, pressing 30 min before bedtime, 2d or 2-3d/session, 2-3 sessions/week, 4 weeks/course for 2 courses G: 1 session/1-2 days, 4 weeks/course for 2 courses	1 session/1-2 days, 4 weeks/course for 2 courses	①Total effective rate ②PSQI ③ISI

Table A3. Baseline characteristics of included studies (continued).

Study ID	Acupoints selection			Intervention details	Control details	Outcomes
	Variation in acupoints	Intervention acupoints	Control acupoints			
Zhou YB et al. 2020[65]	Fixed	①Acupuncture points (GV20, GV24+, EX-HN1, Ex-HN22, PC6, HT7, KI6, BL62) ②Ear points (TF_4, AH_{6a}, AT_4, CO_{15}, CO_{10}, CO_{12}, CO_{13})	Acupuncture points (GV20, GV24+, EX-HN1, Ex-HN22, PC6, HT7, KI6, BL62)	A: 50 min/session, 1 session/day, 6 sessions/week for 4 weeks L: 3days/session for 4 weeks	50 min/session, 1 session/day, 6 sessions/week for 4 weeks	①Total effective rate ②PSQI
Wu HH 2020[66]	Semi-standardized	①Acupuncture points (GV20, HT7, SP6, KI6, BL62, Ex-HN22) ②The Du meridian ③The bladder meridian of foot-Taiyang	Acupuncture points (GV20, HT7, SP6, KI6, BL62, Ex-HN22)	1 session/ 2 days, 10 sessions/course for 2 courses	30 min/session, 1 session/ 2 days, 10 sessions/course for 2 courses	①Total effective rate
Shi XD et al. 2020[67]	I, semi-standardized C, fixed	①Acupuncture points (GV20, EX-HN5, GV24+, CV17, PC6, Shangjingming, HT7, BL15, BL14, BL20, SP6) ②The Du meridian ③The bladder meridian of foot-Taiyang	①Acupuncture points (GV20, EX-HN5, GV24+, Shangjingming, CV17, PC6, HT7, BL15, BL14, BL20) ②The Du meridian ③The bladder meridian of foot-Taiyang	2 sessions/week, 10 sessions/course for 2 courses	2 sessions/week, 10 sessions/course for 2 courses	①Total effective rate ②SAS ③SDS ④SF-36
Wu JL et al. 2020[68]	I, fixed C, semi-standardized	Acupuncture points (GB20, Gongxue, EX-HN1, EX-HN5)	Acupuncture points (BL62, KI6, HT7, SP6, Ex-HN22, EX-HN1)	30 min/session, 1 session/day, 6 sessions/week for 4 weeks (sparse wave, 2Hz)	30 min/session, 1 session/day, 6 sessions/week for 4 weeks	①Total effective rate ②PSQI ③HAMD ④HAMA

Table A3. Baseline characteristics of included studies (continued).

Study ID	Acupoints selection			Intervention details	Control details	Outcomes
	Variation in acupoints	Intervention acupoints	Control acupoints			
Li ZB et al. 2020[69]	Semi-standardized	①Acupuncture points (EX-HN1, HT7, KI6, GV24+, Ex-HN22, BL62) ②Abdominal acupuncture	Acupuncture points (EX-HN1, HT7, SP6, BL62, KI6)	30 min/session, 1 session/day, 10 sessions/course for 2 courses (continuous wave)	30 min/session, 1 session/day, 10 sessions/course for 2 courses.	①Total effective rate ②PSQI
Cao WT 2020[70]	I, fixed C, semi-standardized	①Eye acupuncture ②Wrist-ankle acupuncture	Acupuncture points (KI6, BL62, HT7, SP6, EX-HN1)	30 min/session, 10 days/course for 2 courses	10 days/course for 2 courses	①Total effective rate ②PSQI
Li JY 2020[71]	Fixed	Acupuncture points (GV20, GV24+, HT7, SP6)	Acupuncture points (GV20, GV24+, HT7, SP6)	2 sessions/week for 4 weeks	2 sessions/week for 4 weeks	①Total effective rate ②PSQI
Wang YP 2020[72]	Fixed	①Acupuncture points (GV24+, GV24, GV20, EX-HN5, CV12, CV6, CV4, CV8) ②The Du meridian ③The bladder meridian of foot-Taiyang	Acupuncture points (GV24+, EX-HN5, GB20, BL23, BL15, BL18)	1session/2 days, 20 days/course for 2 courses	3-5min/point, 1session/2 days, 20 days/course for 2 courses	①Total effective rate ②PSQI
Zhang XY et al. 2020[73]	I, fixed C, semi-standardized	①Acupuncture points (GV20, GV24+, GV16, GV11, BL44, GV14, GV4) ②The bladder meridian of foot-Taiyang	Acupuncture points (KI6, BL62, HT7, SP6, Ex-HN22, EX-HN1)	1 session/day, 5 days/week for 4 weeks	30 min/session, 1 manipulation/15 min, 1 session/day, 5 days/week for 4 weeks (dense-sparse waves)	①Total effective rate ②PSQI

Table A3. Baseline characteristics of included studies (continued).

Study ID	Acupoints selection			Intervention details	Control details	Outcomes
	Variation in acupoints	Intervention acupoints	Control acupoints			
Guo SX et al. 2020[74]	Fixed	①Acupuncture points (HT7, BL15, BL18, BL20)+Scalp acupuncture ②Ear points (CO_{12}, CO_{10}, CO_{18}, TF_4, AT_4)	Ear points (CO_{12}, CO_{10}, CO_{18}, TF_4, AT_4)	L: pressing 3-4 times/day, 20 s/point for 21 days<br>D: 21 days	Pressing 3-4 times/day, 20 s/point for 21 days	①Total effective rate ②PSQI ③SOL ④TST ⑤REM ⑥NREM ⑦rCBF ⑧SSWF
Chen XM 2020[75]	Fixed	Acupuncture points (PC6, HT7, ST36, SP6, LR3, ST40, CV12)	Acupuncture points (PC6, HT7, ST36, SP6, LR3, ST40)	A: 25 min/session, 5 sessions/week for 10 days<br>G: 1 session/day, 2h/session, 10 days/course	25 min/session, 5 sessions/week for 10 days	①PSQI ②SOL ③TST ④Number of Awakenings
Wei M 2020[76]	Fixed	Acupuncture points (GV24+, GV24, GV20, GB14, EX-HN1, BL2, EX-HN5, Ex-HN22, BL1, EX-HN4, ST1, ST2, ST5, ST6, GB21)	Ear points (TF_4, CO_{15}, CO_{10}, AT_4, AH_{6a})	1 session/day, 5 sessions/week for 4 weeks	Pressing 3 times/day, 2-3min/session, change every 3 days (2 times/week) for 4 weeks	①PSQI ②ISI ③CRP ④IL-6 ⑤SOL ⑥TST ⑦SE ⑧Number of Awakenings

Table A3. Baseline characteristics of included studies (continued).

Study ID	Acupoints selection			Intervention details	Control details	Outcomes
	Variation in acupoints	Intervention acupoints	Control acupoints			
Ye YW et al. 2021[77]	Fixed	Acupuncture points (CV12, CV8, ST25, BL1, GV23, LI20, GV24+, GV25)	Acupuncture points (CV12, CV8, ST25)	B: 1 session/day, 15 min/session for 4 weeks.<br>M: 1 session/day, 5 days/week for 4 weeks.	1 session/day, 5 days/week for 4 weeks.	①Total effective rate<br>②PSQI<br>③AIS<br>④TNF-α<br>⑤IL-6<br>⑥IL-1β
Chen Y et al. 2021[78]	Fixed	Scalp acupuncture	Acupuncture points (GV20, EX-HN5, EX-HN1)	30 min/session, 1 session/2-3 days, 7 sessions/course	30 min/session, 1 session/2-3 days, 7 sessions/course	①Total effective rate<br>②AIS
Cao WQ et al. 2021[79]	Semi-standardized	Acupuncture points (BL18, BL15, BL20, BL13, BL23, BL17, HT7, PC6, Ex-HN22, GV20)	Acupuncture points (HT7, PC6, Ex-HN22, GV20)	A: 30 min/session, 1 session/day, 5 days/week for 4 weeks<br>F: 1 session/2 days, 3 sessions/week for 4 weeks	30 min/session, 1 session/day, 5 days/week for 4 weeks	①Total effective rate<br>②PSQI<br>③SAS<br>④SDS
Lan L et al. 2021[80]	Fixed	①Umbilicus needling therapy<br>②Ear points (TF_4, AH_{6a}, CO_{18}, CO_{15}, CO_{12}, CO_{13}, CO_{10})	Ear points (TF_4, AH_{6a}, CO_{18}, CO_{15}, CO_{12}, CO_{13}, CO_{10})	A: 55 min/session, 1 session/day, 5 days/week for 8 weeks<br>L: pressing 3 times/day, 1 min/point, 1 session/2 days for 8 weeks	Pressing 3 times/day, 1 min/point, 1 session/2 days for 8 weeks	①Total effective rate<br>②PSQI
Xie J et al. 2021[81]	I, semi-standardized<br>C, fixed	Acupuncture points (GV20, HT7, Faxuan points, TTH, SP6, PC6)	Acupuncture points (GV20, HT7)	1 session/day, 10 days/course for 2 courses	30 min/session, 1 manipulation/10 min, 1 session/day, 10 days/course for 2 courses	①Total effective rate<br>②PSQI<br>③AIS

Table A3. Baseline characteristics of included studies (continued).

Study ID	Acupoints selection			Intervention details	Control details	Outcomes
	Variation in acupoints	Intervention acupoints	Control acupoints			
Jing RZ et al. 2021[82]	Fixed	Acupuncture points (KI1, Ex-HN22, HT7, SP6)	Acupuncture points (KI1)	G: 1 session/day for 7 days<br>F: pressing 4 times/day, 2-3min/session, change every 48-72h for 2 times	1 session/day for 7 days	①Total effective rate<br>②PSQI
Liu LL et al. 2022[83]	Fixed	①Acupuncture points (GV20, EX-HN1, CV12, CV7)<br>②Scalp acupuncture	Acupuncture points (GV20, EX-HN1, GV24+, Ex-HN22, HT7, PC6, SP6, BL62, KI6)	30 min/session, manipulation 1 min/point, 1 session/day, 10 sessions/course for 3 courses	30 min/session, manipulation 1 min/point, 1 session/day, 10 sessions/course for 3 courses	①Total effective rate
Gao M et al. 2022[84]	Semi-standardized	①Acupuncture points (SP6, HT7, GV20, PC6, Ex-HN22)<br>②Ear points (TF_4, CO_{15}, AT2,3,4i, CO_{10}, AT_4, CO_{12}, CO_{13})	Acupuncture points (SP6, HT7, GV20, PC6, Ex-HN22)	K: 30 min/session, 1 session/day for 10 days<br>L: pressing 4 times/day, 3 min/session, pressing 1 session before bedtime, change every 4 days for 10 days	30 min/session, 1 session/day for 10 days	①Total effective rate<br>②PSQI<br>③QOL<br>④SDS<br>⑤SAS<br>⑥Traditional Chinese Medicine Symptom Score Scale

Table A3. Baseline characteristics of included studies (continued).

Study ID	Acupoints selection			Intervention details	Control details	Outcomes
	Variation in acupoints	Intervention acupoints	Control acupoints			
Xu XY et al. 2022[85]	Fixed	①Acupuncture points (GV20, HT7, KI6, SP6, BL62)<br>②Scalp acupuncture	Acupuncture points (GV20, HT7, KI6, SP6, BL62)	30 min/session, 1 manipulation/10 min, 1 session/day, 6 days/week for 2 weeks	30 min/session, 1 manipulation/10 min, 1 session/day, 6 days/week for 2 weeks	①Total effective rate<br>②PSQI<br>③CPSS<br>④PSG (TST, WASO, SOL, SE, REM, NREM)
Xie QL 2022[86]	Fixed	①Acupuncture points (SI19, TE21, GB2, GV20, LR3, ST40)<br>②Ear points (CO_{15}, AT_4, TF_4, AT_3, Ps)	Acupuncture points (SI19, TE21, GB2)	D: 30 min/session<br>LM: pressing 3 times/day, 1 min/session, pressing 30 min before bedtime, change every 2 days, 7 days/course for 2 courses	30 min/session	①Total effective rate<br>②AIS

Table A3. Baseline characteristics of included studies (continued).

Study ID	Acupoints selection			Intervention details	Control details	Outcomes
	Variation in acupoints	Intervention acupoints	Control acupoints			
Wang YH et al. 2023[87]	I, semi-standardized C, fixed	①Acupuncture points (HT7, PC6, Ex-HN22, BL20, LR3, GV20) ②Ear points (CO_{15}, AH_{6a}, TF_4, AT_4)	Acupuncture points (HT7, PC6, Ex-HN22, BL20, LR3, GV20)	A: 30 min/session for 8 weeks L: pressing 90s/session, 3-6 sessions/day for 8 weeks	30 min/session for 8 weeks	①Total effective rate ②PSQI ③GAD-7 ④PHQ-9 ⑤Traditional Chinese Medicine Symptom Score Scale ⑥PSG (SOL, TST, SE)
Zhao JJ et al. 2023[88]	Fixed	①Acupuncture points (EX-HN1, Ex-HN22, HT7, BL62, KI6, SP6) ②The Du meridian	Acupuncture points (EX-HN1, Ex-HN22, HT7, BL62, KI6, SP6)	A: 30 min/session, 1 session/2 days, 3 sessions/week, 3 weeks/course B: 40 min/session, 3 moxibustion cones/session, 1 session/week, 3 weeks/course	30 min/session, 1 session/2 days, 3 sessions/week, 3 weeks/course	①Total effective rate ②PSQI ③Average Thermal Value of the Frontal and Spinal Regions
Yang WQ 2023[89]	Fixed	Acupuncture points (Ex-HN22, GV20, PC6, HT7, ST36, SP6, LR3)	Acupuncture points (Ex-HN22, GV20, PC6, HT7, ST36, SP6, LR3)	20-30 min/session, 1 session/day, 7 days/course for 4 courses	20-30 min/session, 1 session/day, 7 days/course for 4 courses	①Total effective rate ②PSQI ③ISI

Table A3. Baseline characteristics of included studies (continued).

Study ID	Acupoints selection			Intervention details	Control details	Outcomes
	Variation in acupoints	Intervention acupoints	Control acupoints			
Jiang L 2023[90]	Fixed	Acupuncture points (GV20, EX-HN1, ST8,EX-HN5, Ex-HN22, LI4, GV24+, KI6)	Acupuncture points (GV20, EX-HN1, ST8,EX-HN5, Ex-HN22, LI4, GV24+, KI6)	A: 30 min/session, 1 session/day, 6 sessions/week for 4 weeks<br>F: pressing 3-5 times/day, 1 session/2 days for 4 weeks	30 min/session, 1 session/day, 6 sessions/week for 4 weeks	①Total effective rate ②PSQI ③SDS ④SAS ⑤ISI
Wang SQ 2023[91]	Fixed	Fu's subcutaneous needling	Acupuncture points (KI6, BL62, Ex-HN22, HT7, EX-HN1, SP6)	1 session/2 days, 10 sessions/course for 1 course	1 session/2 days, 10 sessions/course for 1 course	①Total effective rate ②PSQI ③HAMA ④AIS ⑤WHOQOL -100 ⑥Traditional Chinese Medicine Symptom Score Scale
Liu DL et al. 2024[92]	Fixed	Acupuncture points (GV20, HT7, SP6, KI6, BL62, Ex-HN22, GV14, BL15, BL18)	Acupuncture points (GV20, HT7, SP6, Ex-HN22, KI6, BL62)	A: 30 min/session, 1 session/day for 2 weeks<br>E: bloodletting 2-5ml/point, 2 sessions/week for 2 weeks	30 min/session, 1 session/day for 2 weeks	①Total effective rate ②PSQI ③SAS ④SDS

Table A3. Baseline characteristics of included studies (continued).

Study ID	Acupoints selection			Intervention details	Control details	Outcomes
	Variation in acupoints	Intervention acupoints	Control acupoints			
Fan J et al. 2024[93]	Fixed	Ear points (CO_{15}, AH_{6a}, TF_4, CO_{13}, CO_{10})	Ear points (CO_{15}, AH_{6a}, TF_4, CO_{13}, CO_{10})	L: Pressing 5 times/day, 2 min/session for 4 weeks D: 10 min/session, 2 sessions/week for 4 weeks	Pressing 5 times/day, 2 min/session for 4 weeks	①Total effective rate ②PSQI ③AIS ④Traditional Chinese Medicine Symptom Score Scale ⑤SOL ⑥TST ⑦Number of Awakenings
Liu YY et al. 2024[94]	Fixed	①Acupuncture points (HT8, HT7) ②Scalp acupuncture	Acupuncture points (GV20, HT7, SP6, KI6, BL62, Ex-HN22)	30 min/session, 1 manipulation/10 min, manipulation 30s/point, 1 session/day, 5 days/course for 2 courses	30 min/session, 1 manipulation/10 min, manipulation 30s/point, 1 session/day, 5 days/course for 2 courses	①PSQI ②GABA ③Glu
Zhuang J et al. 2024[95]	Fixed	Acupuncture points (GV24+, GV23, ST8, BL2, TE23, EX-HN5, GV20, GB20, BL15, GV9, CV8, KI1)	Acupuncture points (GV24+, GV23, ST8, BL2, TE23, EX-HN5, GV20, GB20)	1 session/day, 5 days/courses for 3 courses.	1 session/day, 5 days/courses for 3 courses.	①Total effective rate ②PSQI ③Traditional Chinese Medicine Symptom Score Scale

[1] Tang T, Wang JL, Gao JX. Observations on the efficacy of shallow puncture plus cupping for treating 30 insomnia patients. Shanghai J Acupunct Moxibustion. 2006;25(11):10–11. (In Chinese)

[2] Xing QJ. Clinical study on acupuncture of defensive Qi adjustment and brain invigoration for treatment of insomnia [dissertation]. Zhengzhou: Henan University of Chinese Medicine; 2007. (In Chinese)

[3] Wang YM, Wu HY, Huang GQ. Observation on therapeutic effect of acupuncture plus moxibustion for insomnia. J Acupunct Tuina Sci. 2007;5(5):281–283. (In Chinese)

[4] Yang L, Lai XS, Feng SL. Clinical observation on 63 cases of insomnia treated with bloodletting therapy combined with electroacupuncture. New Chin Med. 2008;40(1):62–63. (In Chinese)

[5] Xia Y, Gao JX. Treatment of 30 cases of insomnia with moxibustion mainly at Baihui (GV20). J Clin Acupunct Moxibustion. 2008;24(11):23–24. (In Chinese)

[6] Gao XY, Wang XM, Wei YL, Shao SJ, Li XR, Zhang HJ, et al. Multicenter clinical study on regulating defensive Qi and invigorating brain acupuncture therapy for insomnia. In: Proceedings of the Symposium on Standardized Research Approaches in Acupuncture Diagnosis and Treatment; 2008. p. 165–168. (In Chinese)

[7] Liang YL, Lu LQ, Yang XQ, Liang JL. Nursing observation of bloodletting and cupping combined with acupuncture for insomnia. Nurs Pract Res. 2009;6(6):79–80. (In Chinese)

[8] Li ZP, Yan XY, Zhu XY. Clinical study on acupoint catgut embedding therapy for insomnia. Liaoning J Tradit Chin Med. 2010;37(10):2020–2022. (In Chinese)

[9] Chen FM. Clinical observation of bloodletting therapy combined with acupuncture for simple insomnia [dissertation]. Guangzhou: Guangzhou University of Chinese Medicine; 2010. (In Chinese)

[10] Shi YJ, Li HJ, Zhang HF. Clinical observation on catgut embedding at scalp acupoints for treating insomnia. Shanghai J Acupunct Moxibustion. 2011;30(11):738–740. (In Chinese)

[11] Lian FC, Peng LH. Clinical observation of ear apex bloodletting combined with cupping along Back-Shu points for the treatment of 78 cases of insomnia. Chin Manip Rehabil Med. 2011;2(9):194–195. (In Chinese)

[12] Gu RX, Jiao Y, Xu DD. Clinical study on insomnia treated by catgut embedding applied to Shu-Mu acupoints jointly. Shanghai J Acupunct Moxibustion. 2011;30(2):101–103. (In Chinese)

[13] Li ZP, Zhu XY, Yan XY. Therapeutic observation on improving efficacy of moxibustion at Baihui for insomnia. World J Acupunct Moxibustion. 2011;21(1):25–29. (In Chinese)

[14] Li ZP, Zhou X, Liang ZH, Zhu XP, Fu WB. Efficacy analysis of acupuncture combined with thread embedding at acupoints for insomnia. New Traditional Chinese Medicine.

2011;43(11):84–86. (In Chinese)

[15] Shi L, Zhang QX, Zhang JL, Xie LQ, Chen J. Clinical study on gastrodin acupoint injection for treating insomnia. Chinese Journal of Geriatric Health Care. 2011;9(3):41–43. (In Chinese)

[16] Feng X, Yin JJ. Observation on the therapeutic effect of acupuncture combined with acupoint injection for insomnia. Chinese and Foreign Medical Research. 2012;10(29):116-116. (In Chinese)

[17] Li ZM, Wang W, Zhang Y. Controlled clinical observation of Treating Insomnia by Acupuncture Combined with Massage. Practical Journal of Chinese Internal Medicine. 2012;26(4):68-69. (In Chinese)

[18] Shi MW. Penetrating needling therapy on insomnia treated with acupuncture curative effect research [dissertation]. Henan University of Chinese Medicine; 2012. (In Chinese)

[19] Qiu FX, Yang XM. Observation on effect of acupuncture in coordination to point application to treat insomnia patients. Chin Gen Pract Nurs. 2012;10(1):10-11. (In Chinese)

[20] Lin YH, Chen DS. Clinical observation of ear acupuncture combined with needling for the treatment of insomnia. In: Proceedings of the 14th National Academic Seminar on Auricular Acupuncture and the Establishment of the Auricular Acupuncture Committee of the Guangdong Acupuncture Association; 2012; Guangdong. p. 112-113. (In Chinese)

[21] Jian H. Clinical observation of therapeutic efficacy of electric acupuncture and pressing seeds on ear acupoints for insomnia [D]. Guangzhou: Guangzhou University of Chinese Medicine; 2012. (In Chinese)

[22] Yao HF, Zhang HF, Chen XL. Observation on therapeutic effect of scalp-acupoint catgut embedding for 33 cases of insomnia patients. Acupunct Res. 2012;37(5):394-397. (In Chinese)

[23] Gao XY, Xu CX, Wang PY, Ren S, Zhou YL, Yang XG, et al. Curative effect of acupuncture and moxibustion on insomnia: A randomized clinical trial. J Tradit Chin Med. 2013;33(4):428-432.

[24] Zhang Y, Li LD, Qu J. Clinical observation on the treatment of insomnia with the "three-in-one" brain balance therapy. Clin J Tradit Chin Med. 2013;25(3):223-224. (In Chinese)

[25] Zhang HF, Yao HF, Zhou SJ. Effect of head acupoints catgut embedding on Pittsburgh sleep quality index in patients with insomnia. Chin Arch Tradit Chin Med. 2013;31(11):2420-2422. (In Chinese)

[26] Guo AS, Li AH, Feng LF, Chen X, Sun L. Clinical study of acupoint catgut-embedding therapy on patients with insomnia. J Nanjing Univ Tradit Chin Med. 2013;29(4):331-334. (In Chinese)

[27] Cao JK. Clinical observation of acupuncturing the area of spiritual emotion to treat primary insomnia. Guangzhou University of Chinese Medicine; 2013. (In Chinese)

[28] Xiong QJ, Zeng J. Clinical observation on electroacupuncture combined with acupoint pressure for the treatment of insomnia. Modern Diagnosis and Treatment. 2013;24(1):66-67. (In Chinese)

[29] Wei Y, Tang HQ, Li KM, Li XH. Observation on the therapeutic efficacy of insomnia treated with medicated thread moxibustion of Zhuang medicine plus acupuncture. Shanghai J Acupunct Moxibustion. 2013;32(11):906-907. (In Chinese)

[30] Gu TT, Zhong WQ. Clinical observation on effect of warm needling therapy in back-Shu points combined with auricular therapy in treating 30 cases of insomnia. World Chin Med. 2013;8(11):1342-1347. (In Chinese)

[31] Sun YZ, Yu T. Clinical research on the first area of Sun's abdominal acupuncture treatment for insomnia. J Clin Acupunct Moxibustion. 2014;30(12):42-44. (In Chinese)

[32] Feng SS, Zhou HF. Clinical observation on auricular point pressing seeds treatment of insomnia. J Liaoning Univ Tradit Chin Med. 2014;16(9):177-179. (In Chinese)

[33] Fu DM, Ke R. Acupuncturing at Shenmen point combined with stimulated auricular points for insomnia: clinical research on 60 cases. J Clin Acupunct Moxibustion. 2014;30(10):22-24. (In Chinese)

[34] Ma QY, Li Y, Cao LY, Zhang YY. Therapeutic observation of moxibustion at the governor vessel plus auricular point sticking for insomnia. Shanghai J Acupunct Moxibustion. 2014;33(7):624-626. (In Chinese)

[35] Wang YQ, Zhou JH, Chen YC. Clinical observation of Tujia San Shui acupoint therapy for insomnia. J Pract Tradit Chin Med. 2014;30(4):314. (In Chinese)

[36] Xie ZY, Wu X. Clinical observation on acupuncture point massage combined with auricular point pressing for insomnia in 30 cases. Hunan J Tradit Chin Med. 2015;31(8):80-81. (In Chinese)

[37] Xie ZY, Wu X, Hong J. Observation on the efficacy of acupoint massage plus moxibustion for refractory insomnia. J Acupunct Tuina Sci. 2015;13(1):44-48. (In Chinese)

[38] Zhao JP, Hong Y, Hong J. Clinical observation on acupuncture plus tuina for insomnia. J Acupunct Tuina Sci. 2015;13(4):232-235. (In Chinese)

[39] Liu ZL, Wang S. Clinical observation of thread embedding at tender points on the Governor vessel for intractable insomnia. Shanghai J Acupunct Moxibustion. 2015;34(12):1188-1189. (In Chinese)

[40] Xu LL, Xu FY. Therapeutic observation of auricular acupuncture with electroacupuncture and auricular acupressure for insomnia. Chin J Trauma Disabil Med. 2015;23(17):24-25. (In Chinese)

[41] Chen P. Clinical observation on 50 cases of insomnia treated with Du Meridian moxibustion combined with auricular point pressing therapy. Fam Psychol Doctor. 2015;11(3):1. (In Chinese)

[42] Huang G. Observation on the efficacy of acupuncture and acupressure combined therapy for sleep disorders. Fam Psychol Doctor. 2015. (In Chinese)

[43] Li BK. Study on clinical effect of acupuncture combined with ear point tapping in treatment of anhypnia. Chin Foreign Med Treat. 2016;36(10):158–159.

[44] Wang FY. Clinical observation on electro acupuncture of sleeping points in the treatment of insomnia. Guangming J Chin Med. 2016;31(16):2387–2389.

[45] Liu LN. Clinical observation of acupoint massage combined with auricular seed embedding for tranquilizing yang and nourishing yin in the treatment of insomnia. Dajia Jiankang (Mid-Month Edition). 2016;10(4):110. (In Chinese)

[46] Wu J, Yao ZJ, Ye P, et al. Effect of catgut embedding combined with ear acupoint sticking on PSQI in patients with insomnia. J Clin Acupunct. 2016;32(5):35–37. (In Chinese)

[47] Zhou LL. A study on the clinical efficacy of guiding fire to origin with moxibustion in combination with acupuncture in the treatment of primary insomnia and its impact on patients' serum γ-aminobutyric acid [dissertation]. Fujian: Fujian University of Traditional Chinese Medicine; 2016. (In Chinese)

[48] Chang XL. Electric-acupuncture combined with auricular acupressure in the treatment of primary insomnia for 38 cases. Chin J Mod Distance Educ Chin Med. 2016;14(22):112–114. (In Chinese)

[49] Zhang SN, Ouyang LZ, Wang XZ, Zeng FK, Fang Q, Li JW, et al. Clinical observation on the efficacy of acupuncture combined with tuina for insomnia. In: Proceedings of the Annual Conference of Hunan Acupuncture Association; 2016. p. 93–95. (In Chinese)

[50] Pan XM, Zhang L, Yang Y, Duan YL, Liu YC, Zhang Y. Clinical observation on the efficacy of electroacupuncture in the treatment of moderate to severe insomnia. J Cardiovasc Dis Integr Tradit Chin West Med (Electron Ed). 2016;4(12):51–52,54. (In Chinese)

[51] Ning SY, Yang YH, Chu LL, Zhang B, Gao X, Dong SQ. Acupuncture combined with ear seed treatment for intractable insomnia. J Clin Acupunct Moxibustion.

2016;32(4):14–16. (In Chinese)

[52] Zhao YQ, Alatengqimuge. Acupuncture combined with Mongolian medicine warm needling in the treatment of 43 cases of insomnia. Food Health Care. 2016;3(12):93–94. (In Chinese)

[53] Ma WH, Zhou Y, Ma XL, Niu XL, Liu TZ. Study on the effect of acupuncture combined with moxibustion on insomnia. Jilin J Chin Med. 2017;37(11):1158–1160. (In Chinese)

[54] Hong YF, Du CH, Xu H, Ni WM, Qin LF. Clinical observation of needling Qin's eight head acupoints for insomnia. Shanghai J Acupunct Moxibustion. 2017;36(6):715–718. (In Chinese)

[55] Chen Y, Gao XY, Sun CY. Pricking and penetrating moxibustion therapy in patients with refractory insomnia: a randomized and controlled clinical trial. J Tradit Chin Med. 2018;38(5):754–762.

[56] Lu CL, Liu L, Liang Y, Pan XJ. Clinical observation on the treatment of insomnia with Youlong cupping combined with acupuncture in 34 cases. Yunnan J Tradit Chin Med Pharm. 2018;39(12):58–59. (In Chinese)

[57] Zhao JY, Ji CC. Clinical observation on the effect of scalp electroacupuncture on sleep quality of patients with primary insomnia. Mod Chin Med. 2018;38(3):14–17. (In Chinese)

[58] Lu WJ. The clinical observation on treating primary insomnia by regulating heel vessels (acupuncture) with beeding therapy [D]. North China University of Science and Technology; 2018. (In Chinese)

[59] Yao JY. Clinical observation on primary insomnia treated by pricking collaterals and cupping combined with acupuncture [D]. Heilongjiang: Heilongjiang University of Chinese Medicine; 2019. (In Chinese)

[60] Li YT. Clinical observation on the therapeutic effect of eye acupuncture in treating insomnia [D]. Liaoning: Liaoning University of Traditional Chinese Medicine; 2019. (In Chinese)

[61] Hong QY, Yang HM, Zhao JP, Wang SG, Yao J, Yang Y. Acupuncture at Wuzangshu combined with Geshu acupoints with fire needles and filiform needles for chronic insomnia. Chinese General Practice. 2019;22(3):336-340. (In Chinese)

[62] Wu XQ, Gao J, Sun X, Wang YJ. Observation on the curative effect of acupuncture for purging the south, tonifying the north and tranquilizing the spirit with cupping and

guasha on intractable insomnia. Modern Journal of Integrated Traditional Chinese and Western Medicine. 2019;28(15):1626-1629. (In Chinese)

[63] Yan QY. Effect of thunder fire moxibustion combined with the acupuncture treatment of meridian flow at specific time points for insomnia. Family Medicine. 2019;(11):390-391. (In Chinese)

[64] Zhou Y, Miao F, Qu Z, Gao J. Observation on therapeutic effect of acupuncture combined with medicine on primary insomnia. J Extern Ther Tradit Chinese Med. 2019;28(5):38-40. (In Chinese)

[65] Zhou YB, Yu XP. Auricular acupuncture combined with conventional acupuncture in the treatment of insomnia. Yishou Baodian. 2020;(8):129-178. (In Chinese)

[66] Wu HH. Clinical observation on the therapeutic effect of triple therapy in the treatment of 60 cases of insomnia. China Health Care and Nutrition. 2020;30(10):372. (In Chinese)

[67] Shi XD, Lu XG, Zhao M, Zhao J, Gao WL, Fan ZY, et al. Clinical study on the treatment of insomnia by Lu's acupuncture plus Zhu's thumb pushing manipulation. Chinese Manipulation and Rehabilitation Medicine. 2020;11(15):56-59. (In Chinese)

[68] Wu JL, Qi MH, Liang J, Yuan Z, Liu LY, Mei RJ, et al. Clinical observation of electroacupuncture at nape acupoints for primary insomnia. Shanghai Journal of Acupuncture and Moxibustion. 2020;39(2):153-157. (In Chinese)

[69] Li ZB, Huang W, Liao JM. Study on the therapeutic effect of Sun's abdominal acupuncture in treating insomnia. Shenzhen Journal of Integrated Traditional Chinese and Western Medicine. 2020;30(2):9-11. (In Chinese)

[70] Cao WT. Discussion on the clinical effectiveness of eye acupuncture combined with wrist and ankle acupuncture in the treatment of insomnia. Electronic Journal of Clinical Medical Literature. 2020;7(88):53-54. (In Chinese)

[71] Li JY. Clinical observation of Lingnan fire acupuncture in treating insomnia. [Dissertation]. Guangdong: Guangzhou University of Chinese Medicine; 2020.

[72] Wang YP. Analysis of the clinical effect of three-part tui-na combined with traditional Chinese medicine acupoint selection and scraping therapy on insomnia. Yangsheng Baojian Zhinan. 2020(1):249. (In Chinese)

[73] Zhang XY, Tang Q, Tang HL, Wang C. Study on the efficacy difference between pestle acupuncture and electroacupuncture in the treatment of primary insomnia. Shizhen Guoyi Guoyao. 2020, 31(9):2166-2168. (In Chinese)

[74] Guo SX, Lu GL. Application of holographic scraping therapy in the treatment of insomnia. Henan Medical Research. 2020, 29(22):4165-4167. (In Chinese)

[75] Chen XM. Clinical efficacy and nursing experience of acupuncture combined with acupoint application in the treatment of insomnia. Yangsheng Baojian Zhinan. 2020(32):169-170. (In Chinese)

[76] Wei M. The clinical observation of TaiChi Tuina in the treatment of chronic insomnia and its effect on serum CRP and IL-6. [Dissertation]. Hubei: Hubei University of Traditional Chinese Medicine; 2020. (In Chinese)

[77] Ye YW, Fang YY. The effects of thunder fire moxibustion combined with meridian dredging on sleep status and inflammatory factors in patients with insomnia. Modern Medicine and Health Research (Electronic Edition). 2021;5(11):34-36. (In Chinese)

[78] Chen Y, Zhang MZ. Observe the clinical effect of acupotomology head acupuncture therapy on intractable insomnia and its effect on sleep quality of patients. World Journal of Sleep Medicine. 2021;8(4):618-619. (In Chinese)

[79] Cao WQ, Zhao M. Clinical efficacy evaluation of fire needle on five zangshu plus diaphragmatic shu combined with filiform acupuncture in the treatment of chronic insomnia. Health Women. 2021;(36):92, 94. (In Chinese)

[80] Lan L, Zhu LL, Nong YP, Chen SY, Liao WY, Mo QM, et al. Clinical study on twelve terrestrial branch hologram needle inserting umbilical acupuncture combined with auricular acupoint pressing magnetic bead in the treatment of insomnia. Guangming Journal of Chinese Medicine. 2021;36(14):2385-2387. (In Chinese)

[81] Xie J, Huang XJ. Observation on the nursing effect of Zhuang medicine line-point moxibustion in the treatment of Nian Lao Nuo (insomnia) syndrome. Health Care Journal. 2021;22(10):120-121. (In Chinese)

[82] Jing RZ, Liu WY, Chen LJ, Yi ZH, Jin YZ, Chen JD. Clinical study on intradermal needle-embedding combined with point application with Fructus Evodiae for insomnia. New Chinese Medicine. 2021;53(22):167-170. (In Chinese)

[83] Liu LL, Shi L. Clinical observation on scalp acupuncture combined with Zhongwan (RN12) and Yinjiao (RN7) electroacupuncture in the treatment of intractable insomnia. Chinese Medicine Modern Distance Education of China. 2022;20(9):122-124. (In Chinese)

[84] Gao M, Gu YM, Wu QX. Observation of therapeutic effect of electroacupuncture combined with auricular point sticking on insomnia. Henan Traditional Chinese Medicine. 2022;42(5):777-780. (In Chinese)

[85] Xu XY, Ma JH, Ou JX, Gan HX, Zhou DJ, Yang ZH, et al. Effect of Fang's scalp acupuncture on perceived stress and sleep structure in insomnia patients: a randomized controlled trial. Zhongguo Zhen Jiu. 2022;42(04):371-376. (In Chinese)

[86] Xie QL. Effect of ear scraping combined with ear acupoint pressure on improving insomnia: a clinical observation. Zhongwen Keji Qikan Database (Citation Edition) Medical and Health, 2022(12):289-292. (In Chinese)

[87] Wang YH, Zhao M, Yang GF, Li FQ, Liu JH, Liu XZ, et al. Acupuncture combined with auricular acupoint pressing beans for primary insomnia. Acta Chinese Medicine, 2023, 38(9):1992-1998. (In Chinese)

[88] Zhao JJ, Huang RL, Li TT. Clinical observation of acupuncture combined with Du meridian moxibustion in the treatment of chronic insomnia. Shanxi Journal of Traditional Chinese Medicine, 2023, 39(9):39-40. (In Chinese)

[89] Yang WQ. Clinical efficacy analysis of treatment of insomnia with acupuncture at key and symptomatic points combined with embedded needle therapy. Chinese Science and Technology Periodicals Database (Full-text Edition) Medicine and Health, 2023(10):117-120. (In Chinese)

[90] Jiang L. Efficacy analysis of conventional acupuncture combined with press-needle therapy in the treatment of primary insomnia. Chinese Science and Technology Periodicals Database (Full-text Edition) Medicine and Health, 2023(6):156-158. (In Chinese)

[91] Wang SQ. The Study of Fu's Subcutaneous Needling Therapy in the Treatment of Insomnia [D]. Guangdong: Guangzhou University of Chinese Medicine, 2023. (In Chinese)

[92] Liu DL, Wei QL, Liu M. Clinical analysis of acupuncture combined with bloodletting and cupping in the treatment of insomnia. Inner Mongolia Journal of Traditional Chinese Medicine. 2024;43(6):132–134. (In Chinese)

[93] Fan J, Liu ZY. Effects of ear holographic copper needle scraping combined with ear pressure beans in treatment of insomnia patients. Med J Chin People’s Health. 2024;36(11):98–101. (In Chinese)

[94] Liu YY, An Q, An JM, Liu YF, Ma KJ, Zhao ZT. Effect of Fang's scalp acupuncture combined with Nazi method of midnight-noon ebb-flow on serum GABA and Glu and sleep quality in patients with insomnia. J Clin Acupunct Moxibustion. 2024;40(6):43–47. (In Chinese)

[95] Zhuang J, Luo PR, Li YF, Zhou C, Xiong J, Zhang N, et al. Clinical effect of head meridian manipulation combined with thermosensitive moxibustion at acupoints for insomnia. Pract Clin Integr Tradit Chin West Med. 2024;24(3):18–21. (In Chinese)

Table A4. Detailed risk of bias ratings based on ROB 2.0.

Study	Bias arising from the randomization process	Bias due to deviations from intended interventions	Bias due to missing outcome data	Bias in measurement of the outcome	Bias in selection of the reported result	Overall risk of bias
Tang T 2006	Some concerns	Some concerns	Low risk	Low risk	Some concerns	Some concerns
Xing QJ 2007	Low risk	Some concerns	Low risk	Low risk	Some concerns	Some concerns
Wang YM 2007	Low risk	Some concerns	Low risk	Low risk	Some concerns	Some concerns
Yang L 2008	High risk	Some concerns	Low risk	Low risk	Some concerns	High risk
Xia Y 2008	High risk	Some concerns	Low risk	Low risk	Some concerns	High risk
Gao XY 2008	High risk	Some concerns	Low risk	Low risk	Some concerns	High risk
Liang YL 2009	High risk	Some concerns	Low risk	Low risk	Some concerns	High risk
Li ZP 2010	Low risk	Some concerns	Low risk	Low risk	Some concerns	Some concerns
Chen FM 2010	Low risk	Some concerns	Low risk	Low risk	Some concerns	Some concerns
Shi YJ 2011	High risk	Some concerns	Low risk	Low risk	Some concerns	High risk
Lian FC 2011	Some concerns	Some concerns	Low risk	Low risk	Some concerns	Some concerns
Gu RX 2011	Some concerns	Some concerns	Low risk	Low risk	Some concerns	Some concerns
Li ZP 2011	Some concerns	Some concerns	Some concerns	Low risk	Some concerns	Some concerns
Li ZP 2011	Low risk	Some concerns	Some concerns	Low risk	Some concerns	Some concerns
Shi L 2011	High risk	Some concerns	Low risk	Low risk	Some concerns	High risk
Feng X 2012	High risk	Some concerns	Low risk	Low risk	Some concerns	High risk
Li ZM 2012	Some concerns	Some concerns	Low risk	Low risk	Some concerns	Some concerns
Shi MW 2012	Low risk	Some concerns	Low risk	Low risk	Some concerns	Some concerns
Qiu FX 2012	High risk	Some concerns	Low risk	Low risk	Some concerns	High risk
Lin YH 2012	Low risk	Some concerns	Low risk	Low risk	Some concerns	Some concerns
Jian HL 2012	Low risk	Some concerns	Low risk	Low risk	Some concerns	Some concerns
YaoHF 2012	Low risk	Some concerns	Low risk	Low risk	Some concerns	Some concerns
Gao XY 2013	Low risk	Some concerns	Low risk	Low risk	Some concerns	Some concerns
Zhang Y 2013	High risk	Some concerns	Low risk	Low risk	Some concerns	High risk
Zhang HF 2013	High risk	Some concerns	Low risk	Low risk	Some concerns	High risk
Guo AS 2013	Some concerns	Some concerns	Low risk	Low risk	Some concerns	Some concerns
Cao JK 2013	Low risk	Some concerns	Some concerns	Low risk	Some concerns	Some concerns
Xiong QJ 2013	High risk	Some concerns	Low risk	Low risk	Some concerns	High risk
Wei Y 2013	Low risk	Some concerns	Low risk	Low risk	Some concerns	Some concerns
Gu TT 2013	Low risk	Some concerns	Low risk	Low risk	Some concerns	Some concerns
Sun YZ 2014	Low risk	Some concerns	Low risk	Low risk	Some concerns	Some concerns
Feng SS 2014	Some concerns	Some concerns	Low risk	Low risk	Some concerns	Some concerns
Fu DM 2014	Low risk	Some concerns	Low risk	Low risk	Some concerns	Some concerns
Ma QY 2014	High risk	Some concerns	Some concerns	Low risk	Some concerns	High risk
Wang YQ 2014	High risk	Some concerns	Low risk	Low risk	Some concerns	High risk
Xie ZY 2015	Low risk	Some concerns	Low risk	Low risk	Some concerns	Some concerns
Xie ZY 2015	Low risk	Some concerns	Low risk	Low risk	Some concerns	Some concerns
Zhao JP 2015	Low risk	Some concerns	Some concerns	Low risk	Some concerns	Some concerns
Liu ZL 2015	Low risk	Some concerns	Low risk	Low risk	Some concerns	Some concerns
Xu LL 2015	Some concerns	Some concerns	Low risk	Low risk	Some concerns	Some concerns
Chen P 2015	High risk	Some concerns	Low risk	Low risk	Some concerns	High risk
Huang G 2015	High risk	Some concerns	Low risk	Low risk	Some concerns	High risk
Li BK 2016	Low risk	Some concerns	Low risk	Low risk	Some concerns	Some concerns
Wang FY 2016	High risk	Some concerns	Low risk	Low risk	Some concerns	High risk
Liu LN 2016	Low risk	Some concerns	Low risk	Low risk	Some concerns	Some concerns
Wu J 2016	Low risk	Some concerns	Low risk	Low risk	Some concerns	Some concerns
Zhou LL 2016	Low risk	Some concerns	Low risk	Low risk	Some concerns	Some concerns
Chang XL 2016	Some concerns	Some concerns	Low risk	Low risk	Some concerns	Some concerns
Zhang SN 2016	High risk	Some concerns	Low risk	Low risk	Some concerns	High risk
Pan XM 2016	Low risk	Some concerns	Some concerns	Low risk	Some concerns	Some concerns
Ning SY 2016	Low risk	Some concerns	Low risk	Low risk	Some concerns	Some concerns
Zhao YQ 2016	High risk	Some concerns	Low risk	Low risk	Some concerns	High risk
Ma WH 2017	High risk	Some concerns	Low risk	Low risk	Some concerns	High risk
Hong YF 2017	Some concerns	Some concerns	Low risk	Low risk	Some concerns	Some concerns
Chen Y 2018	Low risk	Some concerns	Low risk	Low risk	Some concerns	Some concerns
Lu CL 2018	High risk	Some concerns	Low risk	Low risk	Some concerns	High risk
Zhao JY 2018	Some concerns	Some concerns	Low risk	Low risk	Some concerns	Some concerns
Lu WJ 2018	Low risk	Some concerns	Low risk	Low risk	Some concerns	Some concerns
Yao JY 2019	Low risk	Some concerns	Low risk	Low risk	Some concerns	Some concerns
Li YT 2019	Low risk	Some concerns	Low risk	Low risk	Some concerns	Some concerns
Hong QY 2019	Low risk	Some concerns	Low risk	Low risk	Some concerns	Some concerns
Wu XQ 2019	High risk	Some concerns	Some concerns	Low risk	Some concerns	High risk
Yan QY 2019	High risk	Some concerns	Low risk	Low risk	Some concerns	High risk
Zhou Y 2019	High risk	Some concerns	Low risk	Low risk	Some concerns	High risk
Zhou YB 2020	High risk	Some concerns	Low risk	Low risk	Some concerns	High risk
Wu HH 2020	High risk	Some concerns	Low risk	Low risk	Some concerns	High risk
Shi XD 2020	Low risk	Some concerns	Some concerns	Low risk	Some concerns	Some concerns
Wu JL 2020	Low risk	Some concerns	Some concerns	Low risk	Some concerns	Some concerns
Li ZB 2020	Low risk	Some concerns	Low risk	Low risk	Some concerns	Some concerns
Cao WT 2020	Low risk	Some concerns	Low risk	Low risk	Some concerns	Some concerns
Li JY 2020	Low risk	Some concerns	Low risk	Low risk	Some concerns	Some concerns
Wang YP 2020	Some concerns	Some concerns	Some concerns	Low risk	Some concerns	Some concerns
Zhang XY 2020	Some concerns	Some concerns	Some concerns	Low risk	Some concerns	Some concerns
Guo SX 2020	Low risk	Some concerns	Low risk	Low risk	Some concerns	Some concerns
Chen XM 2020	Low risk	Some concerns	Low risk	Low risk	Some concerns	Some concerns
Wei M 2020	Low risk	Some concerns	Some concerns	Low risk	Some concerns	Some concerns
Ye YW 2021	Low risk	Some concerns	Low risk	Low risk	Some concerns	Some concerns
Chen Y 2021	High risk	Some concerns	Low risk	Low risk	Some concerns	High risk
Cao WQ 2021	Low risk	Some concerns	Low risk	Low risk	Some concerns	Some concerns
Lan L 2021	Low risk	Some concerns	Low risk	Low risk	Some concerns	Some concerns
Xie J 2021	Low risk	Some concerns	Low risk	Low risk	Some concerns	Some concerns
Jing RZ 2021	High risk	Some concerns	Some concerns	Low risk	Some concerns	High risk
Liu LL 2022	High risk	Some concerns	Low risk	Low risk	Some concerns	High risk
Gao M 2022	High risk	Some concerns	Low risk	Low risk	Some concerns	High risk
Xu XY 2022	Low risk	Some concerns	Some concerns	Low risk	Some concerns	Some concerns
Xie QL 2022	Low risk	Some concerns	Low risk	Low risk	Some concerns	Some concerns
Wang YH 2023	Low risk	Some concerns	Low risk	Low risk	Some concerns	Some concerns
Zhao JJ 2023	High risk	Some concerns	Low risk	Low risk	Some concerns	High risk
Yang WQ 2023	Low risk	Some concerns	Low risk	Low risk	Some concerns	Some concerns
Jiang L 2023	High risk	Some concerns	Low risk	Low risk	Some concerns	High risk
Wang SQ 2023	Low risk	Some concerns	Some concerns	Low risk	Some concerns	Some concerns
Liu DL 2024	Low risk	Some concerns	Low risk	Low risk	Some concerns	Some concerns
Fan J 2024	Low risk	Some concerns	Low risk	Low risk	Some concerns	Some concerns
Liu YY 2024	Low risk	Some concerns	Some concerns	Low risk	Some concerns	Some concerns
Zhuang J 2024	High risk	Some concerns	Low risk	Low risk	Some concerns	High risk

Table A5. Standard acupuncture point locations.

Acupuncture points/ Ear points	Standard acupuncture point locations
LI4 (Hegu)	On the dorsum of the hand, radial to the midpoint of the second metacarpal bone.
LI20 (Yingxiang)	On the face, in the nasolabial sulcus, at the same level as the midpoint of lateral border of the ala of the nose.
ST1 (Chengqi)	On the face, between the eyeball and the infraorbital margin, directly inferior to the pupil.
ST2 (Sibai)	On the face, in the infraorbital foramen.
ST5 (Daying)	On the face, anterior to the angle of the mandible, in the depression anterior to the masseter attachment, over the facial artery.
ST6 (Jiache)	On the face, one fingerbreadth (middle finger) anterosuperior to the angle of the mandible.
ST7 (Ruzhong)	In the anterior thoracic region, at the centre of the nipple.
ST8 (Touwei)	On the head, 0.5 B-cun directly superior to the anterior hairline at the corner of the forehead, 4.5 B-cun lateral to the anterior median line.
ST25 (Tianshu)	On the upper abdomen, 2 B-cun lateral to the centre of the umbilicus.
ST36 (Zusanli)	On the anterior aspect of the leg, on the line connecting ST35 with ST41, 3 B-cun inferior to ST35.
ST40 (Fenglong)	On the anterolateral aspect of the leg, lateral border of the tibialis anterior muscle, 8 B-cun superior to the prominence of the lateral malleolus.
SP6 (Sanyinjiao)	On the tibial aspect of the leg, posterior to the medial border of the tibia, 3 B-cun superior to the prominence of the medial malleolus.

Table A5. Standard acupuncture point locations (continued).

Acupuncture points/ Ear points	Standard acupuncture point locations
SP9 (Yinlingquan)	On the tibial aspect of the leg, in the depression between the inferior border of the medial condyle of the tibia and the medial border of the tibia.
SP10 (Xuehai)	On the anteromedial aspect of the thigh, on the bulge of the vastus medialis muscle, 2 B-cun superior to the medial end of the base of the patella.
SP12 (Chongmen)	In the groin region, at the inguinal crease, lateral to the femoral artery.
HT3 (Shaohai)	On the anteromedial aspect of the elbow, just anterior to the medial epicondyle of the humerus, at the same level as the cubital crease.
HT7 (Shenmen)	On the anteromedial aspect of the wrist, radial to the flexor carpi ulnaris tendon, on the palmar wrist crease.
HT8 (Shaofu)	On the palm of the hand, in the depression between the fourth and fifth metacarpal bones, proximal to the fifth metacarpophalangeal joint.
HT9 (Shaochong)	On the little finger, radial to the distal phalanx, 0.1 F-cun proximal-lateral to the radial corner of the little fingernail, at the intersection of the vertical line of the radial border of the nail and horizontal line of the base of the little fingernail.
SI19 (Tinggong)	On the face, in the depression between the anterior border of the centre of the tragus and the posterior border of the condylar process of the mandible.
BL1 (Jingming)	On the face, in the depression between the superomedial parts of the inner canthus of the eye and the medial wall of the orbit.
BL2 (Cuanzhu)	On the head, in the depression at the medial end of the eyebrow.

Table A5. Standard acupuncture point locations (continued).

Acupuncture points/ Ear points	Standard acupuncture point locations
BL10 (Tianzhu)	In the posterior region of the neck, at the same level as the superior border of the spinous process of the second cervical vertebra (C2), in the depression lateral to the trapezius muscle.
BL13 (Feishu)	In the upper back region, at the same level as the inferior border of the spinous process of the third thoracic vertebra (T3), 1.5 B-cun lateral to the posterior median line.
BL14 (Jueyinshu)	In the upper back region, at the same level as the inferior border of the spinous process of the fourth thoracic vertebra (T4), 1.5 B-cun lateral to the posterior median line.
BL15 (Xinshu)	In the upper back region, at the same level as the inferior border of the spinous process of the fifth thoracic vertebra (T5), 1.5 B-cun lateral to the posterior median line.
BL17 (Geshu)	In the upper back region, at the same level as the inferior border of the spinous process of the seventh thoracic vertebra (T7), 1.5 B-cun lateral to the posterior median line.
BL18 (Ganshu)	In the upper back region, at the same level as the inferior border of the spinous process of the ninth thoracic vertebra (T9), 1.5 B-cun lateral to the posterior median line.
BL19 (Danshu)	In the upper back region, at the same level as the inferior border of the spinous process of the tenth thoracic vertebra (T10), 1.5 B-cun lateral to the posterior median line.
BL20 (Pishu)	In the upper back region, at the same level as the inferior border of the spinous process of the 11th thoracic vertebra (T11), 1.5 B-cun lateral to the posterior median line.

Table A5. Standard acupuncture point locations (continued).

Acupuncture points/ Ear points	Standard acupuncture point locations
BL21 (Weishu)	In the upper back region, at the same level as the inferior border of the spinous process of the 12th thoracic vertebra (T12), 1.5 B-cun lateral to the posterior median line.
BL22 (Sanjiaoshu)	In the lumbar region, at the same level as the inferior border of the spinous process of the first lumbar vertebra (L1), 1.5 B-cun lateral to the posterior median line.
BL23 (Shenshu)	In the lumbar region, at the same level as the inferior border of the spinous process of the second lumbar vertebra (L2), 1.5 B-cun lateral to the posterior median line.
BL44 (Shentang)	In the upper back region, at the same level as the inferior border of the spinous process of the fifth thoracic vertebra (T5), 3 B-cun lateral to the posterior median line.
BL62 (Shenmai)	On the lateral aspect of the foot, directly inferior to the prominence of the lateral malleolus, in the depression between the inferior border of the lateral malleolus and the calcaneus.
BL54 (Zhibian)	In the buttock region, at the same level as the fourth posterior sacral foramen, 3 B-cun lateral to the median sacral crest.
KI1 (Yongquan)	On the sole of the foot, in the deepest depression of the sole when the toes are flexed.
KI3 (Taixi)	On the posteromedial aspect of the ankle, in the depression between the prominence of the medial malleolus and the calcaneal tendon.
KI6 (Zhaohai)	On the medial aspect of the foot, 1 B-cun inferior to the prominence of the medial malleolus, in the depression inferior to the medial malleolus.

Table A5. Standard acupuncture point locations (continued).

Acupuncture points/ Ear points	Standard acupuncture point locations
KI7 (Fuliu)	On the posteromedial aspect of the leg, anterior to the calcaneal tendon, 2 B-cun superior to the prominence of the medial malleolus.
PC5 (Jianshi)	On the anterior aspect of the forearm, between the tendons of the palmaris longus and the flexor carpi radialis, 3 B-cun proximal to the palmar wrist crease.
PC6 (Neiguan)	On the anterior aspect of the forearm, between the tendons of the palmaris longus and the flexor carpi radialis, 2 B-cun proximal to the palmar wrist crease.
PC7 (Daling)	On the anterior aspect of the wrist, between the tendons of palmaris longus and the flexor carpi radialis, on the palmar wrist crease.
PC9 (Zhongchong)	On the middle finger, at the centre of the tip of the middle finger.
TE21 (Ermen)	On the face, in the depression between the supratragic notch and the condylar process of the mandible.
TE23 (Sizhukong)	On the head, in the depression at the lateral end of the eyebrow.
GB2 (Tinghui)	On the face, in the depression between the intertragic notch and the condylar process of the mandible.
GB7 (Zhengying)	On the head, 2.5 B-cun within the anterior hairline, directly superior to the centre of the pupil.
GB8 (Shuaigu)	On the head, directly superior to the auricular apex, 1.5 B-cun superior to the temporal hairline.
GB14 (Yangbai)	On the head, 1 B-cun superior to the eyebrow, directly superior to the centre of the pupil.

Table A5.Standard acupuncture point locations (continued).

Acupuncture points/ Ear points	Standard acupuncture point locations
GB15 (Toulinqi)	On the head, 0.5 B-cun within the anterior hairline, directly superior to the centre of the pupil.
GB18 (Chengling)	On the head, 4 B-cun within the anterior hairline, directly superior to the centre of the pupil.
GB19 (Naokong)	On the head, at the same level as the superior border of the external occipital protuberance, directly superior to gB20.
GB20 (Fengchi)	In the anterior region of the neck, inferior to the occipital bone, in the depression between the origins of sternocleidomastoid and the trapezius muscles.
GB21 (Jianjing)	In the posterior region of the neck, at the midpoint of the line connecting the spinous process of the seventh cervical vertebra (C7) with the lateral end of the acromion.
LR2 (Xingjian)	On the dorsum of the foot, between the first and second toes, proximal to the web margin, at the border between the red and white flesh.
LR3 (Taichong)	On the dorsum of the foot, between the first and second metatarsal bones, in the depression distal to the junction of the bases of the two bones, over the dorsalis pedis artery.
GV3 (Yaoyangguan)	In the lumbar region, in the depression inferior to the spinous process of the fourth lumbar vertebra (L4), on the posterior median line.
GV4 (Mingmen)	In the lumbar region, in the depression inferior to the spinous process of the second lumbar vertebra (L2), on the posterior median line.

Table A5.Standard acupuncture point locations (continued).

Acupuncture points/ Ear points	Standard acupuncture point locations
GV9 (Zhiyang)	In the upper back region, in the depression inferior to the spinous process of the seventh thoracic vertebra (T7), on the posterior median line.
GV11 (Shendao)	In the upper back region, in the depression inferior to the spinous process of the fifth thoracic vertebra (T5), on the posterior median line.
GV14 (Dazhui)	In the posterior region of the neck, in the depression inferior to the spinous process of the seventh cervical vertebra (C7), on the posterior median line.
GV15 (Yamen)	In the posterior region of the neck, in the depression superior to the spinous process of the second cervical vertebra (C2), on the posterior median line.
GV16 (Fengfu)	In the posterior region of the neck, directly inferior to the external occipital protuberance, in the depression between the trapezius muscles.
GV20 (Baihui)	On the head, 5 B-cun superior to the anterior hairline, on the anterior median line.
GV23 (Shangxing)	On the head, 1 B-cun superior to the anterior hairline, on the anterior median line.
GV24 (Shenting)	On the head, 0.5 B-cun superior to the anterior hairline, on the anterior median line.
GV25 (Suliao)	On the face, at the tip of the nose.
GV26 (Shuigou)	On the face, at the midpoint of the philtrum midline.
GV24+ (Yintang)	Midpoint of the line connecting the left and right Cuanzhu (BL2).

Table A5.Standard acupuncture point locations (continued).

Acupuncture points/ Ear points	Standard acupuncture point locations
CV4 (Guanyuan)	In the lumbar region, at the same level as the inferior border of the spinous process of the fifth lumbar vertebra (L5), 1.5 B-cun lateral to the posterior median line.
CV6 (Qihai)	On the lower abdomen, 1.5 B-cun inferior to the centre of the umbilicus, on the anterior median line.
CV7 (Yinjiao)	On the lower abdomen, 1 B-cun inferior to the centre of the umbilicus, on the anterior median line.
CV8 (Shenque)	On the upper abdomen, in the centre of the umbilicus.
CV12 (Zhongwan)	On the upper abdomen, 4 B-cun superior to the centre of the umbilicus, on the anterior median line.
CV13 (Shangwan)	On the upper abdomen, 5 B-cun superior to the centre of the umbilicus, on the anterior median line.
CV14 (Juque)	On the upper abdomen, 6 B-cun superior to the centre of the umbilicus, on the anterior median line.
CV17 (Danzhong)	In the anterior thoracic region, at the same level as the fourth intercostal space, on the anterior median line.
CV23 (Lianquan)	In the anterior region of the neck, superior to superior border to thyroid cartilage, in the depression superior to the hyoid bone, on the anterior median line.
CV24 (Chengjiang)	On the face, in the depression in the centre of the mentolabial sulcus.
EX-HN1 (Sishencong)	Located 1 inch to the front, back, left, and right of the Baihui (GV20), totaling 4 acupoints.
EX-HN4 (Yuyao)	Located directly above the pupil, in the middle of the eyebrow.

Table A5. Standard acupuncture point locations (continued).

Acupuncture points/ Ear points	Standard acupuncture point locations
EX-HN5 (Taiyang)	Located on the head, between the end of the eyebrow and the outer canthus of the eye, approximately one horizontal finger (middle finger) behind.
EX-HN6 (Erjian)	Located at the highest point of the external ear.
Ex-HN22 (Anmian)	Located at the midpoint of the line connecting Yifeng (TE7) and Fengchi (GB20) in the neck region.
MS1 (Ezhongxian)	Draw a straight line downward from Shenting (GV24) along the meridian, with a length of 1 B-cun.
MS5 (Dingzhongxian)	A straight line along the midline of the head, from Baihui (GV20) to Qianding (GV21).
Shangjingming	Located on the face, about 0.2 cun above the inner canthus of the eye, at the medial edge of the supraorbital rim, approximately 0.2 B-cun above the Jingming (BL1) along the Bladder Meridian.
Rutu	The Rutu point is located on the posterior edge of the ear, near the root of the ear, specifically on the back of the ear's helix, close to the region where the mastoid bone is located.
Shimian	Located at the tail end of the posterior groove of the helix crus and the junction with the posterior groove of the antihelix.
Gongxue	1.5 cun below Fengchi (GB20), at the level of the mouth.
TTH (Tianhuan points)	Refers to a series of acupoints located on the top of the head, forming concentric rings around the highest point of the head. Starting from the center, acupoints are placed 1, 2, and 3 cun away, forming three rings. Each ring corresponds to the positions of the 1-12 o'clock on a clock, totaling 36 acupoints

Table A5. Standard acupuncture point locations (continued).

Acupuncture points/ Ear points	Standard acupuncture point locations
Faxuan points	Located at the crown of the head, where the hair forms a whirlpool or spiral pattern
TF_4 (Shenmen)	Located in the upper part of the posterior third of the triangular fossa, specifically in the fourth region (zone 4) of the triangular fossa.
$AT_{2,3,4i}$ (Yuanzhong)	Located on the free margin of the antihelix, at the midpoint between the apex of the antihelix and the notch of the antihelix, specifically at the junction of zones 2, 3, and 4 of the antihelix.
AT_3 (Zhen)	Located at the posterior part of the outer surface of the antihelix, specifically in the third region (zone 3) of the antihelix.
AT_4 (Pizhixia)	Located on the inner surface of the antihelix, specifically in the fourth zone of the antihelix.
AH_{6a} (Jiaogan)	Located at the junction of the anterior end of the lower crus of the antihelix and the inner edge of the antihelix, specifically at the anterior part of zone 6 of the antihelix.
CO_4 (Wei)	Located at the point where the crus of the helix disappears, specifically in zone 4 of the auricle.
CO_{10} (Shen)	Located in the posterior part beneath the lower crus of the antihelix, specifically in zone 10 of the auricle.
CO_{11} (Yidan)	Located in the upper posterior part of the auricular concha, specifically in zone 11 of the auricle.
CO_{12} (Gan)	Located in the lower posterior part of the auricular concha, specifically in zone 12 of the auricle.
CO_{13} (Pi)	Located below the BD line in the upper posterior part of the auricular concha, specifically in zone 13 of the auricle.

Table A5. Standard acupuncture point locations (continued).

Acupuncture points/ Ear points	Standard acupuncture point locations
CO_{15} (Xin)	Located in the central depression of the auricular cavity, specifically in zone 15 of the auricle.
CO_{17} (Sanjiao)	Located posterior and inferior to the external ear canal, between the CO14 and CO18, specifically in zone 17 of the auricle.
CO_{18} (Neifenmi)	Located within the intertragic notch at the bottom of the auricular concha, specifically in zone 18 of the auricle.
TG_{2p} (Shenshangxian)	Located at the tip of the lower free edge of the tragus, specifically at the posterior edge of zone 2 of the auricle.
Ps (Erbeigou)	Located at the groove of the antihelix and the grooves of the upper and lower antihelix legs.

Table A6. I-squared (%)

Outcomes	I-squared (%)
Total effective rate	72.406972
PSQI	97.67268
Change in PSQI scores	95.863763
Sleep Qualitsy	92.969579
Sleep Latency	96.118944
Sleep Duration	83.903595
Sleep Efficiency	68.393952
Sleep Disturbance Index	87.976615
Daytime Dysfunction	78.729041

Table A7. Prediction interval for total effective rate.

Comparison	Effect Size	Standard Error	LCI	UCI	LPrI	UPrI
AB vs A	0.1695202	0.030821	0.1091122	0.2299283	0.0925602	0.2464802
ABGN vs A	0.3365315	0.0789097	0.1818713	0.4911916	0.1685481	0.5045148
AC vs A	0.1754936	0.0730374	0.0323428	0.3186443	0.0191338	0.3318533
ACD vs A	0.1995421	0.1022567	-0.0008772	0.3999615	-0.0151429	0.4142272
ACF vs A	0.2186892	0.0848404	0.0524052	0.3849733	0.0389068	0.3984716
AE vs A	0.1774393	0.0413798	0.0963364	0.2585422	0.0818154	0.2730633
AF vs A	0.2671748	0.0778054	0.114679	0.4196706	0.1013821	0.4329675
AG vs A	0.230796	0.0574815	0.1181343	0.3434576	0.1048208	0.3567712
AH vs A	0.0736118	0.0855229	-0.0940099	0.2412336	-0.1075317	0.2547553
AHK vs A	0.145182	0.0934285	-0.0379344	0.3282985	-0.0517709	0.342135
AJ vs A	0.2392297	0.0935707	0.0558345	0.4226249	0.0419917	0.4364677
AK vs A	0.3592518	0.0905723	0.1817334	0.5367702	0.1680191	0.5504844
AL vs A	0.231813	0.0315651	0.1699466	0.2936794	0.1535891	0.3100369
AM vs A	0.1728143	0.0449694	0.0846759	0.2609528	0.0705715	0.2750571
B vs A	0.0309263	0.0733323	-0.1128024	0.174655	-0.1260156	0.1878681
BE vs A	0.1850708	0.1286184	-0.0670167	0.4371582	-0.0828945	0.4530361
BF vs A	0.1750617	0.1141927	-0.0487519	0.3988753	-0.0636969	0.4138204
BH vs A	2.51E-13	0.0524472	-0.1027946	0.1027946	-0.1163248	0.1163248
BL vs A	0.1443154	0.0742006	-0.0011151	0.2897459	-0.0143414	0.3029722
BM vs A	0.2528257	0.089113	0.0781674	0.427484	0.064512	0.4411394
CE vs A	0.0540672	0.0571259	-0.0578975	0.166032	-0.0712227	0.1793572
D vs A	-0.1029694	0.1192279	-0.3366518	0.130713	-0.3519105	0.1459717
DL vs A	0.1754984	0.0794721	0.0197359	0.3312608	0.0063984	0.3445983
DLM vs A	0.3198772	0.1873795	-0.0473798	0.6871343	-0.0677573	0.7075117
EK vs A	0.2694727	0.0758998	0.1207118	0.4182336	0.1074553	0.4314901
F vs A	0.053404	0.0484069	-0.0414718	0.1482799	-0.0552689	0.1620769
FG vs A	0.3114263	0.0767185	0.1610609	0.4617917	0.1477878	0.4750647
FH vs A	0.2097205	0.1039818	0.0059199	0.4135211	-0.0084376	0.4278786
FJ vs A	0.1967103	0.1139199	-0.0265685	0.4199891	-0.041497	0.4349176
G vs A	0.1464711	0.0507444	0.047014	0.2459282	0.0333825	0.2595597
GL vs A	0.268264	0.1902589	-0.1046366	0.6411647	-0.1252521	0.6617801
H vs A	0.2719337	0.1416604	-0.0057155	0.5495829	-0.0225133	0.5663807
K vs A	0.1229613	0.0459566	0.032888	0.2130346	0.0188803	0.2270422
K vs A	0.2470145	0.1155005	0.0206376	0.4733913	0.0056125	0.4884165
I vs A	0.3263597	0.0743376	0.1806607	0.4720587	0.1674322	0.4852872
KL vs A	0.1967103	0.1139199	-0.0265685	0.4199891	-0.041497	0.4349176
KM vs A	0.036784	0.0627486	-0.086201	0.1597691	-0.0993976	0.1729657
L vs A	0.2933122	0.0982007	0.1008423	0.4857821	0.0867827	0.4998417
LM vs A	0.0842467	0.073599	-0.0600047	0.2284981	-0.0732218	0.2417151
ABGN vs AB	0.1670112	0.0843866	0.0016166	0.3324058	-0.0118665	0.3458889
AC vs AB	0.0059733	0.0795155	-0.1498743	0.1618209	-0.1632128	0.1751595
ACD vs AB	0.0300219	0.1068006	-0.1793034	0.2393472	-0.1938159	0.2538597
ACF vs AB	0.049169	0.0902653	-0.1277477	0.2260856	-0.1414494	0.2397873
AE vs AB	0.0079191	0.0518273	-0.0936606	0.1094988	-0.1072259	0.1230641

Table A7. Prediction interval for total effective rate (continued).

Comparison	Effect Size	Standard Error	LCI	UCI	LPrI	UPrI
AF vs AB	0.0976546	0.0832705	-0.0655525	0.2608617	-0.0789993	0.2743084
AG vs AB	0.0612757	0.0648961	-0.0659182	0.1884697	-0.0790938	0.2016453
AH vs AB	-0.0959084	0.0909071	-0.274083	0.0822662	-0.2878111	0.0959943
AHK vs AB	-0.0243382	0.098381	-0.2171614	0.1684849	-0.2312298	0.1825533
AJ vs AB	0.0697094	0.098516	-0.1233785	0.2627974	-0.1374535	0.2768724
AK vs AB	0.1897315	0.0953796	0.002791	0.376672	-0.011134	0.390597
AL vs AB	0.0622928	0.0441852	-0.0243086	0.1488941	-0.038495	0.1630805
AM vs AB	0.0032941	0.0522431	-0.0991006	0.1056887	-0.1126421	0.1192303
B vs AB	-0.138594	0.0776423	-0.2907702	0.0135822	-0.3040633	0.0268754
BE vs AB	0.0155505	0.1311237	-0.2414472	0.2725483	-0.2574968	0.2885979
BF vs AB	0.0055415	0.1170073	-0.2237886	0.2348716	-0.2389073	0.2499902
BH vs AB	-0.1695202	0.0608329	-0.2887505	-0.05029	-0.3019783	-0.0370622
BL vs AB	-0.0252048	0.0789809	-0.1800047	0.129595	-0.1933296	0.1429199
BM vs AB	0.0833055	0.0932957	-0.0995508	0.2661618	-0.1133814	0.2799923
CE vs AB	-0.115453	0.06491	-0.2426742	0.0117682	-0.2558497	0.0249437
D vs AB	-0.2724896	0.1225918	-0.5127652	-0.0322141	-0.5282409	-0.0167384
DL vs AB	0.0059781	0.0836156	-0.1579055	0.1698617	-0.1713633	0.1833195
DLM vs AB	0.150357	0.1895377	-0.2211301	0.5218441	-0.2416859	0.5423999
EK vs AB	0.0999524	0.0815791	-0.0599397	0.2598446	-0.073335	0.2732399
F vs AB	-0.1161162	0.0570616	-0.227955	-0.0042775	-0.2412823	0.0090499
FG vs AB	0.141906	0.0818916	-0.0185986	0.3024106	-0.032003	0.3158151
FH vs AB	0.0402003	0.1084534	-0.1723646	0.2527651	-0.1869706	0.2673712
FJ vs AB	0.0271901	0.1180156	-0.2041162	0.2584963	-0.219298	0.2736782
G vs AB	-0.0230492	0.0590513	-0.1387875	0.0926892	-0.1520557	0.1059573
GL vs AB	0.0987437	0.1927392	-0.2790181	0.4765056	-0.2998395	0.497327
H vs AB	0.1024135	0.1449745	-0.1817312	0.3865582	-0.1987721	0.403599
K vs AB	-0.046559	0.0548301	-0.154024	0.0609061	-0.1674373	0.0743194
K vs AB	0.0774942	0.1192451	-0.1562218	0.3112102	-0.1714815	0.32647
I vs AB	0.1568395	0.0802213	-0.0003915	0.3140704	-0.0137486	0.3274276
KL vs AB	0.0271901	0.1180156	-0.2041162	0.2584963	-0.219298	0.2736782
KM vs AB	-0.1327362	0.0694002	-0.2687581	0.0032856	-0.281932	0.0164595
L vs AB	0.1237919	0.1024454	-0.0769974	0.3245813	-0.091273	0.3388569
LM vs AB	-0.0852736	0.078932	-0.2399774	0.0694302	-0.2533011	0.082754
AC vs ABGN	-0.1610379	0.0910031	-0.3394006	0.0173249	-0.3531328	0.031057
ACD vs ABGN	-0.1369893	0.1291633	-0.3901448	0.1161662	-0.4060598	0.1320812
ACF vs ABGN	-0.1178422	0.1158647	-0.3449329	0.1092484	-0.3599805	0.124296
AE vs ABGN	-0.1590921	0.0891355	-0.3337946	0.0156103	-0.3474508	0.0292666
AF vs ABGN	-0.0693566	0.1107364	-0.2863961	0.1476828	-0.3011344	0.1624212
AG vs ABGN	-0.1057355	0.0975702	-0.2969696	0.0854986	-0.3109984	0.0995274
AH vs ABGN	-0.2629196	0.1163654	-0.4909916	-0.0348477	-0.5060702	-0.0197691
AHK vs ABGN	-0.1913494	0.1222932	-0.4310397	0.0483408	-0.4464958	0.063797
AJ vs ABGN	-0.0973018	0.1224019	-0.337205	0.1426015	-0.3526683	0.1580648
AK vs ABGN	0.0227203	0.1010263	-0.1752876	0.2207282	-0.1894893	0.2349299
AL vs ABGN	-0.1047185	0.0843448	-0.2700313	0.0605944	-0.283513	0.0740761

Table A7. Prediction interval for total effective rate (continued).

Comparison	Effect Size	Standard Error	LCI	UCI	LPrI	UPrI
AM vs ABGN	-0.1637171	0.0885985	-0.337367	0.0099328	-0.3510023	0.023568
B vs ABGN	-0.3056052	0.1053005	-0.5119904	-0.09922	-0.5264197	-0.0847907
BE vs ABGN	-0.1514607	0.1491754	-0.443839	0.1409177	-0.4611925	0.1582712
BF vs ABGN	-0.1614697	0.1369327	-0.429853	0.1069135	-0.4463103	0.1233708
BH vs ABGN	-0.3365315	0.0947494	-0.5222368	-0.1508261	-0.5361328	-0.1369302
BL vs ABGN	-0.192216	0.1038239	-0.3957072	0.0112751	-0.4100562	0.0256242
BM vs ABGN	-0.0837057	0.1094109	-0.2981472	0.1307357	-0.3128083	0.1453968
CE vs ABGN	-0.2824642	0.0974172	-0.4733984	-0.09153	-0.4874198	-0.0775086
D vs ABGN	-0.4395008	0.1351217	-0.7043344	-0.1746672	-0.7206633	-0.1583384
DL vs ABGN	-0.1610331	0.1064089	-0.3695908	0.0475245	-0.3840814	0.0620151
DLM vs ABGN	-0.0166542	0.1978732	-0.4044786	0.3711702	-0.4257287	0.3924203
EK vs ABGN	-0.0670588	0.0881121	-0.2397553	0.1056378	-0.2533718	0.1192543
F vs ABGN	-0.2831274	0.0879733	-0.455552	-0.1107029	-0.4691632	-0.0970917
FG vs ABGN	-0.0251052	0.1099057	-0.2405164	0.1903061	-0.2552062	0.2049959
FH vs ABGN	-0.1268109	0.1305334	-0.3826516	0.1290297	-0.3986605	0.1450386
FJ vs ABGN	-0.1398212	0.1385802	-0.4114334	0.1317911	-0.4280085	0.1483662
G vs ABGN	-0.1900604	0.0937657	-0.3738379	-0.0062829	-0.3876894	0.0075686
GL vs ABGN	-0.0682675	0.2059738	-0.4719687	0.3354338	-0.4939013	0.3573664
H vs ABGN	-0.0645977	0.1621555	-0.3824166	0.2532211	-0.4007648	0.2715693
K vs ABGN	-0.2135702	0.0641467	-0.3392955	-0.0878449	-0.3524768	-0.0746635
K vs ABGN	-0.089517	0.1356451	-0.3553765	0.1763425	-0.3717423	0.1927084
I vs ABGN	-0.0101717	0.0867925	-0.1802819	0.1599384	-0.193849	0.1735056
KL vs ABGN	-0.1398212	0.1385802	-0.4114334	0.1317911	-0.4280085	0.1483662
KM vs ABGN	-0.2997474	0.09485	-0.4856501	-0.1138447	-0.4995506	-0.0999442
L vs ABGN	-0.0432193	0.1192294	-0.2769046	0.190466	-0.2921633	0.2057248
LM vs ABGN	-0.2522848	0.0972589	-0.4429086	-0.0616609	-0.4569224	-0.0476471
ACD vs AC	0.0240486	0.1256618	-0.2222441	0.2703412	-0.2379226	0.2860197
ACF vs AC	0.0431956	0.111948	-0.1762184	0.2626097	-0.1910284	0.2774197
AE vs AC	0.0019458	0.0838741	-0.1624444	0.1663359	-0.1759105	0.1798021
AF vs AC	0.0916812	0.106878	-0.1177957	0.3011582	-0.1323126	0.3156751
AG vs AC	0.0553024	0.0930582	-0.1270882	0.2376931	-0.1409084	0.2515132
AH vs AC	-0.1018818	0.1124661	-0.3223113	0.1185478	-0.3371522	0.1333887
AHK vs AC	-0.0303116	0.118589	-0.2627417	0.2021186	-0.2779598	0.2173367
AJ vs AC	0.0637361	0.1187011	-0.1689137	0.2963859	-0.1841389	0.3116111
AK vs AC	0.1837582	0.1012845	-0.0147557	0.3822721	-0.0289707	0.3964871
AL vs AC	0.0563194	0.0775648	-0.0957048	0.2083437	-0.1089962	0.221635
AM vs AC	-0.0026793	0.0845598	-0.1684134	0.1630549	-0.1819022	0.1765437
B vs AC	-0.1445673	0.096429	-0.3335646	0.04443	-0.3475387	0.0584041
BE vs AC	0.0095772	0.1430513	-0.2707983	0.2899527	-0.2876977	0.3068521
BF vs AC	-0.0004318	0.1302343	-0.2556863	0.2548227	-0.2716747	0.270811
BH vs AC	-0.1754936	0.0899176	-0.3517288	0.0007417	-0.3654163	0.0144292
BL vs AC	-0.0311782	0.0911376	-0.2098046	0.1474482	-0.2235423	0.161186
BM vs AC	0.0773321	0.1062081	-0.130832	0.2854962	-0.1453114	0.2999757
CE vs AC	-0.1214264	0.0927245	-0.3031631	0.0603104	-0.3169686	0.0741159

Table A7. Prediction interval for total effective rate (continued).

Comparison	Effect Size	Standard Error	LCI	UCI	LPrI	UPrI
D vs AC	-0.278463	0.1323977	-0.5379577	-0.0189683	-0.5540956	-0.0028304
DL vs AC	4.77E-06	0.0924638	-0.1812209	0.1812304	-0.1950151	0.1950246
DLM vs AC	0.1443837	0.1960232	-0.2398146	0.528582	-0.2609099	0.5496772
EK vs AC	0.0939791	0.0884062	-0.0792939	0.2672521	-0.0929217	0.2808799
F vs AC	-0.1220896	0.0839707	-0.2866691	0.04249	-0.3001385	0.0559594
FG vs AC	0.1359327	0.1062298	-0.0722738	0.3441392	-0.0867545	0.3586199
FH vs AC	0.034227	0.1270696	-0.2148249	0.2832788	-0.2305978	0.2990517
FJ vs AC	0.0212167	0.1353226	-0.2440107	0.2864441	-0.2603537	0.3027872
G vs AC	-0.0290225	0.0890413	-0.2035403	0.1454953	-0.2171928	0.1591478
GL vs AC	0.0927704	0.2037963	-0.306663	0.4922038	-0.3284115	0.5139523
H vs AC	0.0964401	0.1593804	-0.2159398	0.4088201	-0.234072	0.4269523
K vs AC	-0.0525323	0.0645507	-0.1790492	0.0739847	-0.1922273	0.0871627
K vs AC	0.0715209	0.1243897	-0.1722785	0.3153203	-0.1878724	0.3309142
I vs AC	0.1508662	0.0870259	-0.0197014	0.3214337	-0.0332771	0.3350094
KL vs AC	0.0212167	0.1353226	-0.2440107	0.2864441	-0.2603537	0.3027872
KM vs AC	-0.1387095	0.0779079	-0.2914061	0.0139871	-0.3047053	0.0272863
L vs AC	0.1178186	0.1110894	-0.0999127	0.3355499	-0.1146719	0.3503091
LM vs AC	-0.0912469	0.0934376	-0.2743812	0.0918874	-0.2882181	0.1057243
ACF vs ACD	0.019147	0.1328695	-0.2412725	0.2795666	-0.2574433	0.2957374
AE vs ACD	-0.0221028	0.1103119	-0.2383102	0.1941045	-0.2530237	0.208818
AF vs ACD	0.0676327	0.1284917	-0.1842064	0.3194717	-0.2000756	0.3353409
AG vs ACD	0.0312538	0.1173054	-0.1986605	0.2611681	-0.2137977	0.2763054
AH vs ACD	-0.1259303	0.1333064	-0.387206	0.1353453	-0.4034073	0.1515467
AHK vs ACD	-0.0543601	0.138511	-0.3258368	0.2171165	-0.342407	0.2336867
AJ vs ACD	0.0396875	0.138607	-0.2319772	0.3113523	-0.2485543	0.3279293
AK vs ACD	0.1597096	0.1366007	-0.1080229	0.4274421	-0.1244566	0.4438758
AL vs ACD	0.0322708	0.1070177	-0.1774799	0.2420216	-0.1920046	0.2565463
AM vs ACD	-0.0267278	0.111708	-0.2456715	0.1922158	-0.2604672	0.2070115
B vs ACD	-0.1686159	0.1258335	-0.4152449	0.0780132	-0.4309349	0.0937031
BE vs ACD	-0.0144714	0.1643141	-0.3365211	0.3075784	-0.3550384	0.3260957
BF vs ACD	-0.0244804	0.1532854	-0.3249142	0.2759534	-0.3425782	0.2936174
BH vs ACD	-0.1995421	0.1149223	-0.4247857	0.0257014	-0.4397753	0.040691
BL vs ACD	-0.0552268	0.1263414	-0.3028514	0.1923979	-0.3185754	0.2081219
BM vs ACD	0.0532835	0.1356376	-0.2125612	0.3191283	-0.2289266	0.3354937
CE vs ACD	-0.1454749	0.1171315	-0.3750485	0.0840987	-0.3901749	0.0992251
D vs ACD	-0.3025115	0.1570723	-0.6103677	0.0053446	-0.6283215	0.0232985
DL vs ACD	-0.0240438	0.1295077	-0.2778742	0.2297866	-0.2938128	0.2457251
DLM vs ACD	0.1203351	0.2134655	-0.2980495	0.5387197	-0.3206196	0.5612898
EK vs ACD	0.0699305	0.1273468	-0.1796646	0.3195257	-0.1954563	0.3353173
F vs ACD	-0.1461381	0.1131356	-0.3678798	0.0756035	-0.3827609	0.0904846
FG vs ACD	0.1118841	0.1278364	-0.1386707	0.3624389	-0.1544954	0.3782637
FH vs ACD	0.0101784	0.1458377	-0.2756583	0.2960151	-0.292763	0.3131197
FJ vs ACD	-0.0028319	0.1530822	-0.3028675	0.2972037	-0.320516	0.3148523
G vs ACD	-0.0530711	0.1141552	-0.2768112	0.1706691	-0.291754	0.1856118

Table A7. Prediction interval for total effective rate (continued).

Comparison	Effect Size	Standard Error	LCI	UCI	LPrI	UPrI
GL vs ACD	0.0687218	0.2159974	-0.3546253	0.492069	-0.3774121	0.5148557
H vs ACD	0.0723916	0.1747114	-0.2700365	0.4148197	-0.2893813	0.4341644
K vs ACD	-0.0765809	0.112109	-0.2963105	0.1431488	-0.3111301	0.1579684
K vs ACD	0.0474723	0.1542621	-0.2548758	0.3498205	-0.2726143	0.3675589
I vs ACD	0.1268176	0.1264219	-0.1209648	0.3746	-0.1366942	0.3903294
KL vs ACD	-0.0028319	0.1530822	-0.3028675	0.2972037	-0.320516	0.3148523
KM vs ACD	-0.1627581	0.1199742	-0.3979033	0.0723871	-0.4132096	0.0876934
L vs ACD	0.09377	0.1417738	-0.1841015	0.3716416	-0.2009076	0.3884477
LM vs ACD	-0.1152955	0.125989	-0.3622295	0.1316385	-0.3779299	0.1473389
AE vs ACF	-0.0412499	0.0943937	-0.2262582	0.1437584	-0.2401379	0.1576382
AF vs ACF	0.0484856	0.1151155	-0.1771366	0.2741078	-0.192138	0.2891092
AG vs ACF	0.0121068	0.1024793	-0.188749	0.2129625	-0.2030264	0.2272399
AH vs ACF	-0.1450774	0.120466	-0.3811863	0.0910316	-0.3965242	0.1063694
AHK vs ACF	-0.0735072	0.1262013	-0.3208572	0.1738428	-0.3365718	0.1895574
AJ vs ACF	0.0205405	0.1263066	-0.2270159	0.2680969	-0.2427376	0.2838186
AK vs ACF	0.1405625	0.1241017	-0.1026722	0.3837973	-0.1182471	0.3993722
AL vs ACF	0.0131238	0.090522	-0.1642962	0.1905437	-0.1780083	0.2042559
AM vs ACF	-0.0458749	0.0960215	-0.2340736	0.1423239	-0.2480285	0.1562788
B vs ACF	-0.1877629	0.1121406	-0.4075545	0.0320286	-0.4223759	0.0468501
BE vs ACF	-0.0336184	0.1540798	-0.3356093	0.2683724	-0.3533338	0.286097
BF vs ACF	-0.0436275	0.1422598	-0.3224516	0.2351967	-0.3392932	0.2520382
BH vs ACF	-0.2186892	0.0997426	-0.4141812	-0.0231972	-0.4283174	-0.009061
BL vs ACF	-0.0743738	0.1127103	-0.2952819	0.1465343	-0.3101375	0.1613898
BM vs ACF	0.0341365	0.1230407	-0.2070188	0.2752918	-0.2225239	0.2907969
CE vs ACF	-0.164622	0.1022803	-0.3650877	0.0358437	-0.3793546	0.0501106
D vs ACF	-0.3216586	0.1463324	-0.6084649	-0.0348523	-0.6256062	-0.017711
DL vs ACF	-0.0431909	0.1162485	-0.2710336	0.1846519	-0.286105	0.1997233
DLM vs ACF	0.101188	0.2056914	-0.3019597	0.5043358	-0.3238685	0.5262445
EK vs ACF	0.0507835	0.1138362	-0.1723313	0.2738982	-0.1872547	0.2888217
F vs ACF	-0.1652852	0.0976786	-0.3567318	0.0261614	-0.3707659	0.0401955
FG vs ACF	0.0927371	0.1143836	-0.1314507	0.3169248	-0.1464074	0.3318815
FH vs ACF	-0.0089687	0.1342017	-0.2719992	0.2540619	-0.2882633	0.270326
FJ vs ACF	-0.0219789	0.1420409	-0.300374	0.2564162	-0.3171996	0.2732418
G vs ACF	-0.0722181	0.0988579	-0.265976	0.1215397	-0.280068	0.1356317
GL vs ACF	0.0495748	0.2083179	-0.3587208	0.4578704	-0.3808523	0.4800019
H vs ACF	0.0532445	0.1651228	-0.2703902	0.3768793	-0.2889712	0.3954602
K vs ACF	-0.0957279	0.0964878	-0.2848405	0.0933847	-0.2988175	0.1073616
K vs ACF	0.0283253	0.1433117	-0.2525606	0.3092111	-0.2694791	0.3261296
I vs ACF	0.1076705	0.1128005	-0.1134145	0.3287555	-0.1282754	0.3436165
KL vs ACF	-0.0219789	0.1420409	-0.300374	0.2564162	-0.3171996	0.2732418
KM vs ACF	-0.1819052	0.1055238	-0.3887281	0.0249177	-0.4031696	0.0393593
L vs ACF	0.074623	0.1297739	-0.1797292	0.3289752	-0.195686	0.3449319
LM vs ACF	-0.1344425	0.1123152	-0.3545763	0.0856912	-0.3694081	0.1005231
AF vs AE	0.0897355	0.0881843	-0.0831025	0.2625735	-0.0967218	0.2761927

Table A7. Prediction interval for total effective rate (continued).

Comparison	Effect Size	Standard Error	LCI	UCI	LPrI	UPrI
AG vs AE	0.0533566	0.0708719	-0.0855497	0.192263	-0.0987342	0.2054474
AH vs AE	-0.1038275	0.0950076	-0.290039	0.082384	-0.3039468	0.0962918
AHK vs AE	-0.0322573	0.102182	-0.2325304	0.1680158	-0.2467922	0.1822776
AJ vs AE	0.0617904	0.1023121	-0.1387377	0.2623184	-0.1530063	0.276587
AK vs AE	0.1818124	0.0996082	-0.0134162	0.377041	-0.0275456	0.3911705
AL vs AE	0.0543737	0.0520219	-0.0475874	0.1563347	-0.0611414	0.1698887
AM vs AE	-0.004625	0.0614049	-0.1249763	0.1157263	-0.1381935	0.1289435
B vs AE	-0.1465131	0.0841916	-0.3115255	0.0184994	-0.3250021	0.031976
BE vs AE	0.0076314	0.1351047	-0.2571689	0.2724318	-0.2734966	0.2887595
BF vs AE	-0.0023776	0.1214519	-0.240419	0.2356637	-0.2558205	0.2510652
BH vs AE	-0.1774393	0.0668056	-0.308376	-0.0465027	-0.3215442	-0.0333344
BL vs AE	-0.0331239	0.0849968	-0.1997145	0.1334666	-0.2132181	0.1469703
BM vs AE	0.0753864	0.0983766	-0.1174282	0.2682009	-0.1314963	0.2822691
CE vs AE	-0.1233721	0.0705383	-0.2616247	0.0148805	-0.2748064	0.0280621
D vs AE	-0.2804087	0.1262715	-0.5278965	-0.032921	-0.5436158	-0.0172017
DL vs AE	-0.001941	0.0897457	-0.1778394	0.1739574	-0.19152	0.187638
DLM vs AE	0.1424379	0.1919382	-0.2337541	0.5186299	-0.2545089	0.5393847
EK vs AE	0.0920334	0.0864823	-0.0774688	0.2615355	-0.0910247	0.2750914
F vs AE	-0.1240353	0.0637234	-0.2489309	0.0008603	-0.2621163	0.0140456
FG vs AE	0.1339869	0.0872784	-0.0370755	0.3050494	-0.0506606	0.3186345
FH vs AE	0.0322812	0.1119129	-0.1870641	0.2516265	-0.201872	0.2664344
FJ vs AE	0.019271	0.1212024	-0.2182814	0.2568233	-0.2336667	0.2722087
G vs AE	-0.0309683	0.0655209	-0.1593868	0.0974503	-0.1725589	0.1106224
GL vs AE	0.0908246	0.1947068	-0.2907937	0.472443	-0.311779	0.4934283
H vs AE	0.0944944	0.1475803	-0.1947577	0.3837464	-0.2119919	0.4009806
K vs AE	-0.0544781	0.0618904	-0.1757809	0.0668248	-0.18899	0.0800339
K vs AE	0.0695751	0.1226712	-0.170856	0.3100063	-0.1863369	0.3254871
I vs AE	0.1489204	0.0851012	-0.0178749	0.3157157	-0.0313821	0.3292229
KL vs AE	0.019271	0.1212024	-0.2182814	0.2568233	-0.2336667	0.2722087
KM vs AE	-0.1406553	0.0751349	-0.2879169	0.0066063	-0.3011591	0.0198486
L vs AE	0.1158728	0.1065874	-0.0930346	0.3247803	-0.1075351	0.3392808
LM vs AE	-0.0931927	0.0845342	-0.2588767	0.0724913	-0.2723646	0.0859793
AG vs AF	-0.0363788	0.0966389	-0.2257876	0.1530299	-0.2397717	0.167014
AH vs AF	-0.193563	0.1156194	-0.4201728	0.0330469	-0.4352053	0.0480793
AHK vs AF	-0.1219928	0.1215836	-0.3602922	0.1163066	-0.3757023	0.1317167
AJ vs AF	-0.0279451	0.1216929	-0.2664588	0.2105686	-0.2818759	0.2259857
AK vs AF	0.0920769	0.1193271	-0.1417999	0.3259538	-0.1570649	0.3412188
AL vs AF	-0.0353618	0.0840056	-0.2000098	0.1292862	-0.2134803	0.1427567
AM vs AF	-0.0943605	0.089279	-0.2693441	0.0806231	-0.283006	0.094285
B vs AF	-0.2362485	0.1069405	-0.445848	-0.026649	-0.4603684	-0.0121287
BE vs AF	-0.082104	0.1503374	-0.3767599	0.2125519	-0.3942008	0.2299927
BF vs AF	-0.0921131	0.1381977	-0.3629757	0.1787495	-0.3795234	0.1952972
BH vs AF	-0.2671748	0.0938317	-0.4510816	-0.0832681	-0.464936	-0.0694136
BL vs AF	-0.1228594	0.1074273	-0.333413	0.0876942	-0.3479607	0.1022419

Table A7. Prediction interval for total effective rate (continued).

Comparison	Effect Size	Standard Error	LCI	UCI	LPrI	UPrI
BM vs AF	-0.0143491	0.1179964	-0.2456179	0.2169197	-0.2607986	0.2321004
CE vs AF	-0.2131076	0.0965249	-0.4022929	-0.0239223	-0.4162716	-0.0099436
D vs AF	-0.3701442	0.1421955	-0.6488422	-0.0914462	-0.665679	-0.0746094
DL vs AF	-0.0916765	0.1108738	-0.3089851	0.1256322	-0.3237316	0.1403787
DLM vs AF	0.0527024	0.2027691	-0.3447178	0.4501227	-0.3663796	0.4717844
EK vs AF	0.0022979	0.1086122	-0.2105781	0.2151739	-0.2251933	0.229789
F vs AF	-0.2137708	0.0915525	-0.3932104	-0.0343312	-0.4069656	-0.020576
FG vs AF	0.0442515	0.1090067	-0.1693977	0.2579006	-0.1840355	0.2725384
FH vs AF	-0.0574543	0.1298688	-0.3119924	0.1970838	-0.3279556	0.2130471
FJ vs AF	-0.0704645	0.1379544	-0.3408502	0.1999212	-0.3573804	0.2164514
G vs AF	-0.1207037	0.0928009	-0.3025902	0.0611827	-0.316399	0.0749915
GL vs AF	0.0010892	0.2055533	-0.4017878	0.4039662	-0.4236849	0.4258632
H vs AF	0.0047589	0.161621	-0.3120124	0.3215302	-0.3303188	0.3398367
K vs AF	-0.1442135	0.0902653	-0.3211302	0.0327032	-0.3348319	0.0464048
K vs AF	-0.0201603	0.139309	-0.293201	0.2528803	-0.3098285	0.2695079
I vs AF	0.0591849	0.107557	-0.151623	0.2699928	-0.166178	0.2845479
KL vs AF	-0.0704645	0.1379544	-0.3408502	0.1999212	-0.3573804	0.2164514
KM vs AF	-0.2303908	0.1000201	-0.4264265	-0.034355	-0.4405768	-0.0202047
L vs AF	0.0261374	0.1252274	-0.2193037	0.2715785	-0.2349533	0.287228
LM vs AF	-0.1829281	0.1068694	-0.3923882	0.026532	-0.4069046	0.0410483
AH vs AG	-0.1571842	0.1030451	-0.3591488	0.0447804	-0.3734561	0.0590878
AHK vs AG	-0.085614	0.109695	-0.3006123	0.1293844	-0.3152899	0.144062
AJ vs AG	0.0084337	0.1098162	-0.2068021	0.2236695	-0.2214867	0.2383541
AK vs AG	0.1284558	0.1072214	-0.0816943	0.3386059	-0.0962304	0.353142
AL vs AG	0.001017	0.0656102	-0.1275766	0.1296106	-0.1407482	0.1427823
AM vs AG	-0.0579817	0.0725403	-0.200158	0.0841947	-0.2133605	0.0973972
B vs AG	-0.1998697	0.0931922	-0.382523	-0.0172164	-0.396349	-0.0033904
BE vs AG	-0.0457252	0.1408895	-0.3218635	0.2304131	-0.3386053	0.247155
BF vs AG	-0.0557343	0.1278559	-0.3063272	0.1948587	-0.3221532	0.2106847
BH vs AG	-0.230796	0.0778128	-0.3833062	-0.0782857	-0.3966032	-0.0649887
BL vs AG	-0.0864806	0.0937996	-0.2703244	0.0973633	-0.2841774	0.1112163
BM vs AG	0.0220297	0.105837	-0.185407	0.2294664	-0.1998659	0.2439253
CE vs AG	-0.1767288	0.0810401	-0.3355644	-0.0178931	-0.3489442	-0.0045133
D vs AG	-0.3337654	0.1322468	-0.5929644	-0.0745664	-0.6090918	-0.058439
DL vs AG	-0.0552976	0.0978427	-0.2470659	0.1364706	-0.2611079	0.1505127
DLM vs AG	0.0890813	0.1959209	-0.2949166	0.4730791	-0.3160033	0.4941658
EK vs AG	0.0386767	0.0951525	-0.1478187	0.2251721	-0.1617332	0.2390866
F vs AG	-0.177392	0.0750876	-0.324561	-0.0302229	-0.3378024	-0.0169815
FG vs AG	0.0806303	0.095682	-0.106903	0.2681636	-0.120842	0.2821026
FH vs AG	-0.0210754	0.1188122	-0.2539431	0.2117922	-0.2691753	0.2270244
FJ vs AG	-0.0340857	0.1276004	-0.2841778	0.2160065	-0.2999866	0.2318152
G vs AG	-0.0843249	0.0766089	-0.2344757	0.0658259	-0.2477464	0.0790966
GL vs AG	0.037468	0.1987526	-0.3520799	0.4270159	-0.3734037	0.4483397
H vs AG	0.0411377	0.1528783	-0.2584983	0.3407737	-0.2761313	0.3584068

Table A7. Prediction interval for total effective rate (continued).

Comparison	Effect Size	Standard Error	LCI	UCI	LPrI	UPrI
K vs AG	-0.1078347	0.0735201	-0.2519315	0.0362621	-0.2651474	0.049478
K vs AG	0.0162185	0.1290442	-0.2367035	0.2691405	-0.2526103	0.2850473
I vs AG	0.0955637	0.0939326	-0.0885407	0.2796682	-0.1023996	0.2935271
KL vs AG	-0.0340857	0.1276004	-0.2841778	0.2160065	-0.2999866	0.2318152
KM vs AG	-0.1940119	0.0851435	-0.3608902	-0.0271337	-0.3743989	-0.013625
L vs AG	0.0625162	0.1137464	-0.1604226	0.285455	-0.1753405	0.3003729
LM vs AG	-0.1465493	0.0932241	-0.3292652	0.0361666	-0.3430926	0.049994
AHK vs AH	0.0715702	0.1266611	-0.1766811	0.3198214	-0.1924265	0.3355669
AJ vs AH	0.1656179	0.1267661	-0.0828391	0.4140748	-0.0985916	0.4298273
AK vs AH	0.2856399	0.1245692	0.0414887	0.5297912	0.0258829	0.545397
AL vs AH	0.1582012	0.091162	-0.0204731	0.3368755	-0.0342119	0.3506142
AM vs AH	0.0992025	0.0966251	-0.0901792	0.2885842	-0.1041627	0.3025677
B vs AH	-0.0426855	0.1126578	-0.2634909	0.1781198	-0.2783432	0.1929722
BE vs AH	0.111459	0.1544566	-0.1912705	0.4141884	-0.2090238	0.4319417
BF vs AH	0.1014499	0.1426679	-0.1781741	0.3810739	-0.1950454	0.3979453
BH vs AH	-0.0736118	0.1003238	-0.2702429	0.1230192	-0.2844085	0.1371849
BL vs AH	0.0707036	0.1132249	-0.1512132	0.2926204	-0.1660997	0.3075068
BM vs AH	0.1792139	0.1235123	-0.0628658	0.4212935	-0.0784018	0.4368296
CE vs AH	-0.0195446	0.1028471	-0.2211213	0.1820321	-0.2354181	0.1963289
D vs AH	-0.1765812	0.1467292	-0.4641651	0.1110027	-0.481336	0.1281735
DL vs AH	0.1018865	0.1167475	-0.1269344	0.3307074	-0.1420368	0.3458098
DLM vs AH	0.2462654	0.2059739	-0.1574359	0.6499668	-0.1793686	0.6718994
EK vs AH	0.1958609	0.1143457	-0.0282526	0.4199744	-0.043207	0.4349287
F vs AH	-0.0202078	0.098272	-0.2128174	0.1724018	-0.2268805	0.1864649
FG vs AH	0.2378145	0.1148907	0.0126327	0.4629962	-0.0023549	0.4779839
FH vs AH	0.1361087	0.1346342	-0.1277695	0.399987	-0.144064	0.4162815
FJ vs AH	0.1230985	0.1424496	-0.1560977	0.4022946	-0.1729531	0.41915
G vs AH	0.0728593	0.0994442	-0.1220478	0.2677663	-0.136169	0.2818876
GL vs AH	0.1946522	0.2085968	-0.21419	0.6034943	-0.2363452	0.6256496
H vs AH	0.1983219	0.1654745	-0.1260022	0.522646	-0.1446109	0.5412546
K vs AH	0.0493495	0.0970885	-0.1409404	0.2396394	-0.154946	0.2536449
K vs AH	0.1734027	0.1437168	-0.1082772	0.4550825	-0.1252254	0.4720307
I vs AH	0.2527479	0.1133148	0.0306551	0.4748408	0.0157632	0.4897327
KL vs AH	0.1230985	0.1424496	-0.1560977	0.4022946	-0.1729531	0.41915
KM vs AH	-0.0368278	0.1060733	-0.2447277	0.1710722	-0.2591996	0.1855441
L vs AH	0.2197004	0.1302211	-0.0355284	0.4749291	-0.0515158	0.4909166
LM vs AH	0.0106349	0.1128316	-0.2105111	0.2317808	-0.2253738	0.2466436
AJ vs AHK	0.0940477	0.1322284	-0.1651153	0.3532107	-0.1812415	0.3693368
AK vs AHK	0.2140697	0.1301239	-0.0409683	0.4691078	-0.0569491	0.4850886
AL vs AHK	0.086631	0.0986166	-0.106654	0.279916	-0.120734	0.293996
AM vs AHK	0.0276323	0.1036877	-0.1755918	0.2308564	-0.1899335	0.2451981
B vs AHK	-0.1142557	0.1187708	-0.3470423	0.1185308	-0.3622719	0.1337605
BE vs AHK	0.0398888	0.1589704	-0.2716874	0.351465	-0.2897879	0.3695654
BF vs AHK	0.0298797	0.1475427	-0.2592987	0.3190582	-0.2765301	0.3362896

Table A7. Prediction interval for total effective rate (continued).

Comparison	Effect Size	Standard Error	LCI	UCI	LPrI	UPrI
BH vs AHK	-0.145182	0.1071428	-0.3551781	0.0648141	-0.3697098	0.0793458
BL vs AHK	-0.0008666	0.1193089	-0.2347077	0.2329745	-0.2499715	0.2482383
BM vs AHK	0.1076437	0.1291124	-0.1454119	0.3606993	-0.1613234	0.3766108
CE vs AHK	-0.0911148	0.1095091	-0.3057488	0.1235192	-0.3204156	0.138186
D vs AHK	-0.2481514	0.1514733	-0.5450337	0.0487309	-0.5625603	0.0662575
DL vs AHK	0.0303163	0.1226568	-0.2100866	0.2707193	-0.2255666	0.2861992
DLM vs AHK	0.1746952	0.2093799	-0.2356819	0.5850723	-0.2579036	0.6072941
EK vs AHK	0.1242907	0.120373	-0.1116361	0.3602175	-0.126968	0.3755494
F vs AHK	-0.091778	0.1052241	-0.2980134	0.1144575	-0.3124385	0.1288826
FG vs AHK	0.1662443	0.1208909	-0.0706975	0.403186	-0.0860627	0.4185513
FH vs AHK	0.0645385	0.1397895	-0.2094438	0.3385209	-0.226106	0.355183
FJ vs AHK	0.0515283	0.1473317	-0.2372365	0.340293	-0.2544521	0.3575087
G vs AHK	0.0012891	0.1063197	-0.2070937	0.2096718	-0.2215793	0.2241574
GL vs AHK	0.123082	0.2119607	-0.2923534	0.5385174	-0.314795	0.5609589
H vs AHK	0.1267517	0.1696954	-0.2058452	0.4593486	-0.2247882	0.4782917
K vs AHK	-0.0222207	0.1041196	-0.2262914	0.1818499	-0.2406563	0.1962149
K vs AHK	0.1018325	0.1485572	-0.1893344	0.3929993	-0.2066415	0.4103065
I vs AHK	0.1811777	0.1193941	-0.0528305	0.4151859	-0.0680997	0.4304551
KL vs AHK	0.0515283	0.1473317	-0.2372365	0.340293	-0.2544521	0.3575087
KM vs AHK	-0.108398	0.1125445	-0.3289812	0.1121853	-0.3438268	0.1270309
L vs AHK	0.1481302	0.1355443	-0.1175318	0.4137922	-0.1338906	0.4301509
LM vs AHK	-0.0609353	0.1189357	-0.294045	0.1721743	-0.3092851	0.1874144
AK vs AJ	0.1200221	0.130226	-0.1352162	0.3752604	-0.151204	0.3912481
AL vs AJ	-0.0074167	0.0987514	-0.2009658	0.1861324	-0.2150525	0.2002191
AM vs AJ	-0.0664154	0.1038158	-0.2698906	0.1370599	-0.2842392	0.1514085
B vs AJ	-0.2083034	0.1188827	-0.4413093	0.0247025	-0.456546	0.0399392
BE vs AJ	-0.0541589	0.159054	-0.365899	0.2575812	-0.3840059	0.2756881
BF vs AJ	-0.064168	0.1476328	-0.353523	0.2251871	-0.3707611	0.2424252
BH vs AJ	-0.2392297	0.1072669	-0.4494689	-0.0289905	-0.4640076	-0.0144518
BL vs AJ	-0.0949143	0.1194203	-0.3289737	0.1391452	-0.3442447	0.1544161
BM vs AJ	0.013596	0.1292153	-0.2396614	0.2668534	-0.2555799	0.2827719
CE vs AJ	-0.1851625	0.1096305	-0.4000343	0.0297094	-0.4147082	0.0443832
D vs AJ	-0.3421991	0.1515611	-0.6392534	-0.0451448	-0.6567866	-0.0276116
DL vs AJ	-0.0637313	0.1227652	-0.3043467	0.176884	-0.3198337	0.192371
DLM vs AJ	0.0806476	0.2094434	-0.329854	0.4911491	-0.3520812	0.5133763
EK vs AJ	0.030243	0.1204835	-0.2059002	0.2663862	-0.2212392	0.2817252
F vs AJ	-0.1858257	0.1053504	-0.3923087	0.0206573	-0.4067407	0.0350894
FG vs AJ	0.0721966	0.1210008	-0.1649607	0.3093539	-0.180333	0.3247262
FH vs AJ	-0.0295092	0.1398846	-0.3036779	0.2446596	-0.320347	0.2613286
FJ vs AJ	-0.0425194	0.1474219	-0.331461	0.2464222	-0.3486834	0.2636446
G vs AJ	-0.0927586	0.1064447	-0.3013863	0.1158691	-0.315879	0.1303617
GL vs AJ	0.0290343	0.2120234	-0.386524	0.4445926	-0.408971	0.4670396
H vs AJ	0.032704	0.1697738	-0.3000464	0.3654545	-0.3189957	0.3844038
K vs AJ	-0.1162684	0.1042472	-0.3205892	0.0880524	-0.3349611	0.1024243

Table A7. Prediction interval for total effective rate (continued).

Comparison	Effect Size	Standard Error	LCI	UCI	LPrI	UPrI
K vs AJ	0.0077848	0.1486467	-0.2835574	0.299127	-0.3008713	0.3164409
I vs AJ	0.08713	0.1195055	-0.1470963	0.3213564	-0.1623727	0.3366328
KL vs AJ	-0.0425194	0.1474219	-0.331461	0.2464222	-0.3486834	0.2636446
KM vs AJ	-0.2024456	0.1126626	-0.4232604	0.0183691	-0.438113	0.0332217
L vs AJ	0.0540825	0.1356424	-0.2117717	0.3199367	-0.2281374	0.3363024
LM vs AJ	-0.154983	0.1190474	-0.3883117	0.0783457	-0.4035589	0.0935929
AL vs AK	-0.1274388	0.0953451	-0.3143118	0.0594343	-0.3282352	0.0733577
AM vs AK	-0.1864374	0.0991245	-0.3807179	0.007843	-0.3948231	0.0219482
B vs AK	-0.3283255	0.1143016	-0.5523525	-0.1042985	-0.5673041	-0.0893468
BE vs AK	-0.174181	0.1556597	-0.4792684	0.1309065	-0.4971138	0.1487518
BF vs AK	-0.18419	0.1439696	-0.4663652	0.0979851	-0.483332	0.1149519
BH vs AK	-0.3592518	0.1046615	-0.5643846	-0.1541189	-0.578779	-0.1397246
BL vs AK	-0.2149363	0.1129422	-0.436299	0.0064263	-0.4511684	0.0212957
BM vs AK	-0.1064261	0.1180975	-0.3378929	0.1250408	-0.3530799	0.1402278
CE vs AK	-0.3051845	0.1070827	-0.5150628	-0.0953063	-0.5295911	-0.080778
D vs AK	-0.4622211	0.1422472	-0.7410206	-0.1834217	-0.7578612	-0.1665811
DL vs AK	-0.1837534	0.1153215	-0.4097794	0.0422726	-0.4247935	0.0572867
DLM vs AK	-0.0393745	0.2028059	-0.4368667	0.3581177	-0.4585316	0.3797826
EK vs AK	-0.0897791	0.0986937	-0.2832151	0.103657	-0.297299	0.1177409
F vs AK	-0.3058477	0.098569	-0.4990395	-0.112656	-0.5131171	-0.0985783
FG vs AK	-0.0478255	0.1185559	-0.2801909	0.1845399	-0.2954069	0.1997559
FH vs AK	-0.1495312	0.1378969	-0.4198042	0.1207417	-0.4363303	0.1372679
FJ vs AK	-0.1625415	0.1455372	-0.4477891	0.1227062	-0.4648715	0.1397886
G vs AK	-0.2127807	0.1037714	-0.4161689	-0.0093924	-0.4305151	0.0049538
GL vs AK	-0.0909878	0.2107173	-0.5039861	0.3220106	-0.5263218	0.3443463
H vs AK	-0.087318	0.1681398	-0.416866	0.2422299	-0.4356854	0.2610493
K vs AK	-0.2362905	0.0780481	-0.389262	-0.083319	-0.4025644	-0.0700165
K vs AK	-0.1122373	0.1427457	-0.3920136	0.1675391	-0.4088907	0.1844161
I vs AK	-0.032892	0.0975176	-0.224023	0.158239	-0.2380493	0.1722652
KL vs AK	-0.1625415	0.1455372	-0.4477891	0.1227062	-0.4648715	0.1397886
KM vs AK	-0.3224677	0.1047534	-0.5277805	-0.1171549	-0.5421799	-0.1027555
L vs AK	-0.0659396	0.1272487	-0.3153425	0.1834633	-0.3311275	0.1992483
LM vs AK	-0.2750051	0.1069378	-0.4845993	-0.0654109	-0.4991195	-0.0508907
AM vs AL	-0.0589987	0.0548975	-0.1665959	0.0485985	-0.1800062	0.0620089
B vs AL	-0.2008867	0.0777074	-0.3531904	-0.048583	-0.366485	-0.0352884
BE vs AL	-0.0467422	0.1311622	-0.3038154	0.210331	-0.3198677	0.2263833
BF vs AL	-0.0567513	0.1170504	-0.2861659	0.1726633	-0.3012872	0.1877847
BH vs AL	-0.231813	0.0612132	-0.3517887	-0.1118373	-0.3650093	-0.0986167
BL vs AL	-0.0874976	0.076807	-0.2380366	0.0630414	-0.2513114	0.0763163
BM vs AL	0.0210127	0.0936558	-0.1625493	0.2045747	-0.1763959	0.2184213
CE vs AL	-0.1777458	0.0652666	-0.3056659	-0.0498257	-0.3188393	-0.0366523
D vs AL	-0.3347824	0.12264	-0.5751524	-0.0944124	-0.5906312	-0.0789336
DL vs AL	-0.0563146	0.0810696	-0.2152082	0.1025789	-0.2285889	0.1159596
DLM vs AL	0.0880643	0.1895689	-0.2834839	0.4596124	-0.3040423	0.4801708

Table A7. Prediction interval for total effective rate (continued).

Comparison	Effect Size	Standard Error	LCI	UCI	LPrI	UPrI
EK vs AL	0.0376597	0.0815359	-0.1221477	0.1974671	-0.1355417	0.2108611
F vs AL	-0.178409	0.0576275	-0.2913567	-0.0654613	-0.3046657	-0.0521523
FG vs AL	0.0796133	0.0830356	-0.0831335	0.2423601	-0.0965729	0.2557994
FH vs AL	-0.0220925	0.1086672	-0.2350763	0.1908914	-0.2496946	0.2055097
FJ vs AL	-0.0351027	0.118212	-0.2667941	0.1965887	-0.2819883	0.2117829
G vs AL	-0.0853419	0.0597921	-0.2025324	0.0318485	-0.2157823	0.0450985
GL vs AL	0.036451	0.1928596	-0.3415468	0.4144488	-0.3623782	0.4352802
H vs AL	0.0401207	0.1451344	-0.2443376	0.324579	-0.2613902	0.3416317
K vs AL	-0.1088517	0.0547661	-0.2161912	-0.0015122	-0.2296073	0.0119039
K vs AL	0.0152015	0.1165191	-0.2131718	0.2435748	-0.22826	0.258663
I vs AL	0.0945467	0.0800744	-0.0623961	0.2514896	-0.0757493	0.2648428
KL vs AL	-0.0351027	0.118212	-0.2667941	0.1965887	-0.2819883	0.2117829
KM vs AL	-0.1950289	0.0646041	-0.3216506	-0.0684073	-0.3348282	-0.0552297
L vs AL	0.0614992	0.1008068	-0.1360786	0.259077	-0.150269	0.2732673
LM vs AL	-0.1475663	0.0790069	-0.302417	0.0072843	-0.3157426	0.0206099
B vs AM	-0.141888	0.0853564	-0.3091835	0.0254074	-0.3226995	0.0389234
BE vs AM	0.0122565	0.1358337	-0.2539727	0.2784857	-0.2703519	0.2948649
BF vs AM	0.0022474	0.1222624	-0.2373824	0.2418772	-0.2528366	0.2573314
BH vs AM	-0.1728143	0.0690866	-0.3082215	-0.0374071	-0.3213938	-0.0242348
BL vs AM	-0.0284989	0.0850024	-0.1951005	0.1381027	-0.2086043	0.1516065
BM vs AM	0.0800114	0.0896177	-0.0956361	0.2556589	-0.1093116	0.2693344
CE vs AM	-0.1187471	0.0727023	-0.2612409	0.0237467	-0.2744454	0.0369513
D vs AM	-0.2757837	0.119924	-0.5108305	-0.040737	-0.5261335	-0.0254339
DL vs AM	0.002684	0.0881961	-0.1701772	0.1755452	-0.1837969	0.1891649
DLM vs AM	0.1470629	0.1878236	-0.2210645	0.5151904	-0.2414786	0.5356045
EK vs AM	0.0966584	0.0859289	-0.0717591	0.2650759	-0.0852952	0.278612
F vs AM	-0.1194103	0.0651757	-0.2471524	0.0083318	-0.2603263	0.0215057
FG vs AM	0.138612	0.0878208	-0.0335136	0.3107376	-0.047119	0.324343
FH vs AM	0.0369062	0.1132893	-0.1851367	0.2589491	-0.200027	0.2738395
FJ vs AM	0.023896	0.1224744	-0.2161495	0.2639414	-0.2316175	0.2794094
G vs AM	-0.0263432	0.0673798	-0.1584052	0.1057187	-0.1715732	0.1188867
GL vs AM	0.0954497	0.1955012	-0.2877256	0.4786249	-0.3087772	0.4996766
H vs AM	0.0991194	0.1486267	-0.1921836	0.3904224	-0.209496	0.4077348
K vs AM	-0.049853	0.0611141	-0.1696345	0.0699284	-0.1828569	0.0831508
K vs AM	0.0742002	0.1229232	-0.1667249	0.3151252	-0.1822222	0.3306225
I vs AM	0.1535454	0.0846862	-0.0124365	0.3195273	-0.0259296	0.3330204
KL vs AM	0.023896	0.1224744	-0.2161495	0.2639414	-0.2316175	0.2794094
KM vs AM	-0.1360303	0.0755453	-0.2840963	0.0120358	-0.2973461	0.0252855
L vs AM	0.1204979	0.1031977	-0.0817659	0.3227616	-0.0960814	0.3370771
LM vs AM	-0.0885676	0.0747207	-0.2350175	0.0578822	-0.2482525	0.0711172
BE vs B	0.1541445	0.1056677	-0.0529604	0.3612494	-0.0674099	0.3756989
BF vs B	0.1441354	0.0875386	-0.027437	0.3157079	-0.0410318	0.3293027
BH vs B	-0.0309263	0.0901573	-0.2076313	0.1457787	-0.2213286	0.159476
BL vs B	0.1133891	0.0694688	-0.0227671	0.2495454	-0.0359414	0.2627197

Table A7. Prediction interval for total effective rate (continued).

Comparison	Effect Size	Standard Error	LCI	UCI	LPrI	UPrI
BM vs B	0.2218994	0.1117758	0.0028229	0.4409759	-0.0119768	0.4557757
CE vs B	0.0231409	0.092957	-0.1590514	0.2053333	-0.1728671	0.2191489
D vs B	-0.1338957	0.1369832	-0.4023779	0.1345865	-0.4188387	0.1510474
DL vs B	0.1445721	0.0907196	-0.0332351	0.3223792	-0.0469554	0.3360995
DLM vs B	0.288951	0.1991488	-0.1013735	0.6792755	-0.1227305	0.7006325
EK vs B	0.2385464	0.1030643	0.0365441	0.4405487	0.0222357	0.4548571
F vs B	0.0224777	0.0873043	-0.1486356	0.1935911	-0.1622216	0.2071771
FG vs B	0.2805	0.1061723	0.0724061	0.4885939	0.0579287	0.5030714
FH vs B	0.1787942	0.1272393	-0.0705902	0.4281787	-0.0863746	0.4439631
FJ vs B	0.165784	0.135482	-0.0997558	0.4313238	-0.1161101	0.4476781
G vs B	0.1155448	0.0891926	-0.0592694	0.290359	-0.072928	0.3040175
GL vs B	0.2373377	0.2039022	-0.1623032	0.6369786	-0.1840606	0.658736
H vs B	0.2410074	0.1595158	-0.0716377	0.5536526	-0.0897804	0.5717953
K vs B	0.092035	0.0835073	-0.0716362	0.2557062	-0.0850905	0.2691605
K vs B	0.2160882	0.1234515	-0.0258723	0.4580486	-0.0414043	0.4735807
I vs B	0.2954335	0.1019141	0.0956855	0.4951814	0.0814378	0.5094292
KL vs B	0.165784	0.135482	-0.0997558	0.4313238	-0.1161101	0.4476781
KM vs B	0.0058578	0.0764007	-0.1438849	0.1556004	-0.1571514	0.1688669
L vs B	0.2623859	0.1132019	0.0405142	0.4842576	0.0256291	0.4991427
LM vs B	0.0533204	0.0998293	-0.1423414	0.2489822	-0.156482	0.2631228
BF vs BE	-0.0100091	0.1372176	-0.2789506	0.2589324	-0.2954282	0.2754101
BH vs BE	-0.1850708	0.1389007	-0.4573111	0.0871696	-0.4739093	0.1037677
BL vs BE	-0.0407554	0.1264568	-0.2886061	0.2070953	-0.3043378	0.2228271
BM vs BE	0.0677549	0.1538147	-0.2337163	0.3692261	-0.2514206	0.3869304
CE vs BE	-0.1310036	0.140734	-0.4068372	0.1448301	-0.4235677	0.1615606
D vs BE	-0.2880402	0.1730015	-0.6271169	0.0510365	-0.6463242	0.0702438
DL vs BE	-0.0095724	0.1392671	-0.2825309	0.263386	-0.2991554	0.2800105
DLM vs BE	0.1348065	0.2254448	-0.3070573	0.5766702	-0.3306574	0.6002703
EK vs BE	0.0844019	0.1476054	-0.2048993	0.3737032	-0.2221354	0.3909392
F vs BE	-0.1316668	0.1370661	-0.4003114	0.1369779	-0.4167782	0.1534447
FG vs BE	0.1263555	0.149792	-0.1672314	0.4199423	-0.1846312	0.4373421
FH vs BE	0.0246497	0.1653932	-0.299515	0.3488145	-0.3181172	0.3674167
FJ vs BE	0.0116395	0.1718151	-0.3251119	0.3483909	-0.3442241	0.3675032
G vs BE	-0.0385997	0.1382765	-0.3096166	0.2324172	-0.3261699	0.2489705
GL vs BE	0.0831932	0.2296544	-0.3669212	0.5333076	-0.3908861	0.5572725
H vs BE	0.0868629	0.1913383	-0.2881533	0.4618791	-0.3088583	0.4825841
K vs BE	-0.0621095	0.1346795	-0.3260764	0.2018574	-0.3423741	0.2181551
K vs BE	0.0619437	0.1624978	-0.2565461	0.3804335	-0.274921	0.3988084
I vs BE	0.1412889	0.1468046	-0.1464427	0.4290206	-0.1636191	0.446197
KL vs BE	0.0116395	0.1718151	-0.3251119	0.3483909	-0.3442241	0.3675032
KM vs BE	-0.1482867	0.130393	-0.4038523	0.1072788	-0.4198515	0.123278
L vs BE	0.1082414	0.1548543	-0.1952675	0.4117503	-0.2130512	0.429534
LM vs BE	-0.1008241	0.1453651	-0.3857344	0.1840862	-0.4028041	0.2011559
BH vs BF	-0.1750617	0.125661	-0.4213527	0.0712292	-0.4370311	0.0869077

Table A7. Prediction interval for total effective rate (continued).

Comparison	Effect Size	Standard Error	LCI	UCI	LPrI	UPrI
BL vs BF	-0.0307463	0.1117525	-0.2497773	0.1882846	-0.2645756	0.203083
BM vs BF	0.077764	0.1419727	-0.2004973	0.3560253	-0.217318	0.3728459
CE vs BF	-0.1209945	0.1276845	-0.3712516	0.1292626	-0.3870661	0.1450771
D vs BF	-0.2780311	0.1625632	-0.5966492	0.040587	-0.6150293	0.058967
DL vs BF	0.0004366	0.1260659	-0.2466481	0.2475213	-0.2623536	0.2632268
DLM vs BF	0.1448155	0.2175377	-0.2815505	0.5711816	-0.3044693	0.5941004
EK vs BF	0.094411	0.1352207	-0.1706167	0.3594386	-0.1869525	0.3757745
F vs BF	-0.1216577	0.1236301	-0.3639683	0.1206528	-0.379512	0.1361966
FG vs BF	0.1363645	0.1376042	-0.1333347	0.4060638	-0.1498399	0.422569
FH vs BF	0.0346588	0.1544415	-0.268041	0.3373587	-0.2857932	0.3551108
FJ vs BF	0.0216486	0.1613001	-0.2944937	0.3377909	-0.3127752	0.3560723
G vs BF	-0.0285907	0.1249706	-0.2735286	0.2163473	-0.2891611	0.2319798
GL vs BF	0.0932023	0.2218973	-0.3417086	0.5281131	-0.3650024	0.5514069
H vs BF	0.096872	0.181955	-0.2597533	0.4534973	-0.2796856	0.4734295
K vs BF	-0.0521004	0.1209787	-0.2892143	0.1850134	-0.3045852	0.2003843
K vs BF	0.0719527	0.1513368	-0.2246619	0.3685674	-0.2421781	0.3860836
I vs BF	0.151298	0.1343461	-0.1120154	0.4146114	-0.1282896	0.4308856
KL vs BF	0.0216486	0.1613001	-0.2944937	0.3377909	-0.3127752	0.3560723
KM vs BF	-0.1382777	0.1161879	-0.3660018	0.0894464	-0.3810694	0.104514
L vs BF	0.1182505	0.1430984	-0.1622173	0.3987182	-0.1791202	0.4156211
LM vs BF	-0.090815	0.1327716	-0.3510426	0.1694125	-0.3672065	0.1855765
BL vs BH	0.1443154	0.0908649	-0.0337765	0.3224074	-0.0475029	0.3361337
BM vs BH	0.2528257	0.1034013	0.0501629	0.4554886	0.0358365	0.4698149
CE vs BH	0.0540672	0.0775505	-0.0979289	0.2060634	-0.11122	0.2193544
D vs BH	-0.1029694	0.1302536	-0.3582618	0.1523229	-0.3742514	0.1683126
DL vs BH	0.1754984	0.0952183	-0.0111261	0.3621228	-0.0250436	0.3760403
DLM vs BH	0.3198772	0.194581	-0.0614946	0.7012491	-0.0824694	0.7222239
EK vs BH	0.2694727	0.0922577	0.0886509	0.4502945	0.0748656	0.4640798
F vs BH	0.053404	0.0713718	-0.0864822	0.1932902	-0.0996714	0.2064794
FG vs BH	0.3114263	0.0929324	0.1292822	0.4935704	0.1154676	0.507385
FH vs BH	0.2097205	0.11646	-0.0185368	0.4379779	-0.0336213	0.4530624
FJ vs BH	0.1967103	0.1254131	-0.0490948	0.4425154	-0.0647567	0.4581773
G vs BH	0.1464711	0.0729774	0.0034381	0.2895041	-0.0097701	0.3027123
GL vs BH	0.268264	0.1973554	-0.1185455	0.6550735	-0.1397522	0.6762802
H vs BH	0.2719337	0.1510575	-0.0241335	0.5680009	-0.0416286	0.5854961
K vs BH	0.1229613	0.0697332	-0.0137132	0.2596358	-0.0268891	0.2728116
K vs BH	0.2470145	0.1268506	-0.0016081	0.4956371	-0.0173663	0.5113953
I vs BH	0.3263597	0.0909768	0.1480484	0.504671	0.1343174	0.518402
KL vs BH	0.1967103	0.1254131	-0.0490948	0.4425154	-0.0647567	0.4581773
KM vs BH	0.036784	0.0817808	-0.1235034	0.1970714	-0.1369046	0.2104727
L vs BH	0.2933122	0.1113287	0.0751119	0.5115125	0.0603386	0.5262858
LM vs BH	0.0842467	0.0903743	-0.0928838	0.2613771	-0.1065899	0.2750832
BM vs BL	0.1085103	0.1090167	-0.1051585	0.3221791	-0.1197969	0.3368175
CE vs BL	-0.0902482	0.0936435	-0.273786	0.0932896	-0.287632	0.1071356

Table A7. Prediction interval for total effective rate (continued).

Comparison	Effect Size	Standard Error	LCI	UCI	LPrI	UPrI
D vs BL	-0.2472848	0.1348052	-0.5114981	0.0169284	-0.5278046	0.033235
DL vs BL	0.0311829	0.0739327	-0.1137224	0.1760883	-0.1269445	0.1893104
DLM vs BL	0.1755618	0.1976571	-0.2118389	0.5629627	-0.2330709	0.5841946
EK vs BL	0.1251573	0.1015553	-0.0738874	0.324202	-0.0881165	0.338431
F vs BL	-0.0909114	0.0874983	-0.262405	0.0805822	-0.2759982	0.0941755
FG vs BL	0.1671109	0.106567	-0.0417567	0.3759784	-0.0562561	0.3904778
FH vs BL	0.0654051	0.1277417	-0.184964	0.3157743	-0.2007823	0.3315926
FJ vs BL	0.0523949	0.1359539	-0.2140698	0.3188596	-0.2304576	0.3352473
G vs BL	0.0021557	0.089836	-0.1739196	0.1782309	-0.1876038	0.1919151
GL vs BL	0.1239486	0.204216	-0.2763075	0.5242046	-0.2980914	0.5459886
H vs BL	0.1276183	0.1599168	-0.1858128	0.4410495	-0.2039867	0.4592233
K vs BL	-0.0213541	0.0816374	-0.1813604	0.1386522	-0.1947574	0.1520492
K vs BL	0.1026991	0.1120074	-0.1168314	0.3222296	-0.131645	0.3370431
I vs BL	0.1820443	0.1004133	-0.0147621	0.3788508	-0.0289324	0.393021
KL vs BL	0.0523949	0.1359539	-0.2140698	0.3188596	-0.2304576	0.3352473
KM vs BL	-0.1075314	0.056056	-0.2173992	0.0023364	-0.2307626	0.0156999
L vs BL	0.1489968	0.1055026	-0.0577845	0.3557781	-0.0722249	0.3702185
LM vs BL	-0.0600687	0.0968188	-0.2498301	0.1296926	-0.2638227	0.1436853
CE vs BM	-0.1987585	0.1058513	-0.4062232	0.0087062	-0.4206829	0.0231659
D vs BM	-0.3557951	0.1062448	-0.5640312	-0.1475591	-0.5785127	-0.1330776
DL vs BM	-0.0773274	0.1103826	-0.2936733	0.1390186	-0.308391	0.1537362
DLM vs BM	0.0670515	0.1794007	-0.2845673	0.4186704	-0.3042914	0.4383945
EK vs BM	0.016647	0.1072607	-0.19358	0.226874	-0.2081184	0.2414124
F vs BM	-0.1994217	0.09914	-0.3937325	-0.0051109	-0.4078385	0.0089951
FG vs BM	0.0586006	0.1170201	-0.1707547	0.2879558	-0.1858741	0.3030752
FH vs BM	-0.0431052	0.1369428	-0.3115082	0.2252979	-0.3279662	0.2417559
FJ vs BM	-0.0561154	0.1446335	-0.339592	0.2273611	-0.3566076	0.2443768
G vs BM	-0.1063546	0.102358	-0.3069726	0.0942633	-0.3212436	0.1085344
GL vs BM	0.0154383	0.2100942	-0.3963389	0.4272154	-0.4186214	0.449498
H vs BM	0.019108	0.1673582	-0.3089081	0.3471241	-0.3276656	0.3658816
K vs BM	-0.1298644	0.0886339	-0.3035836	0.0438548	-0.3172202	0.0574914
K vs BM	-0.0058112	0.1393486	-0.2789295	0.267307	-0.2955599	0.2839374
I vs BM	0.073534	0.1062338	-0.1346804	0.2817485	-0.1491613	0.2962293
KL vs BM	-0.0561154	0.1446335	-0.339592	0.2273611	-0.3566076	0.2443768
KM vs BM	-0.2160417	0.1000746	-0.4121842	-0.0198991	-0.4263372	-0.0057461
L vs BM	0.0404865	0.1067833	-0.1688049	0.2497779	-0.1833164	0.2642894
LM vs BM	-0.168579	0.0498896	-0.2663608	-0.0707972	-0.2800491	-0.0571089
D vs CE	-0.1570366	0.1322069	-0.4161574	0.1020841	-0.4322821	0.1182088
DL vs CE	0.1214311	0.0978733	-0.0703971	0.3132593	-0.0844406	0.3273029
DLM vs CE	0.26581	0.1958939	-0.1181351	0.6497551	-0.1392195	0.6708395
EK vs CE	0.2154055	0.0949956	0.0292176	0.4015933	0.0153104	0.4155006
F vs CE	-0.0006632	0.0748772	-0.1474199	0.1460935	-0.1606576	0.1593312
FG vs CE	0.2573591	0.0956509	0.0698868	0.4448314	0.0559492	0.4587689
FH vs CE	0.1556533	0.1186406	-0.0768779	0.3881846	-0.0920993	0.4034059

Table A7. Prediction interval for total effective rate (continued).

Comparison	Effect Size	Standard Error	LCI	UCI	LPrI	UPrI
FJ vs CE	0.1426431	0.1274406	-0.1071359	0.3924221	-0.1229339	0.40822
G vs CE	0.0924039	0.0764092	-0.0573554	0.2421631	-0.070622	0.2554297
GL vs CE	0.2141968	0.19865	-0.1751501	0.6035436	-0.1964653	0.6248589
H vs CE	0.2178665	0.152745	-0.0815081	0.5172411	-0.0991311	0.5348641
K vs CE	0.0688941	0.073317	-0.0748046	0.2125927	-0.0880175	0.2258056
K vs CE	0.1929473	0.1288555	-0.0596049	0.4454994	-0.0754989	0.4613934
I vs CE	0.2722925	0.093752	0.0885419	0.4560431	0.074691	0.469894
KL vs CE	0.1426431	0.1274406	-0.1071359	0.3924221	-0.1229339	0.40822
KM vs CE	-0.0172832	0.0848573	-0.1836004	0.1490341	-0.1970993	0.162533
L vs CE	0.239245	0.1136079	0.0165776	0.4619124	0.001668	0.4768219
LM vs CE	0.0301795	0.0931675	-0.1524255	0.2127844	-0.1662505	0.2266094
DL vs D	0.2784677	0.136066	0.0117834	0.5451521	-0.0046123	0.5615478
DLM vs D	0.4228466	0.1445614	0.1395115	0.7061818	0.1225011	0.7231922
EK vs D	0.3724421	0.1333865	0.1110093	0.6338749	0.0948024	0.6500818
F vs D	0.1563734	0.1269402	-0.0924248	0.4051716	-0.108189	0.4209358
FG vs D	0.4143957	0.1414538	0.1371514	0.69164	0.1203686	0.7084227
FH vs D	0.3126899	0.1582008	0.002622	0.6227579	-0.0154189	0.6407988
FJ vs D	0.2996797	0.1649031	-0.0235244	0.6228838	-0.0420881	0.6414474
G vs D	0.2494405	0.1294736	-0.0043231	0.503204	-0.0202592	0.5191402
GL vs D	0.3712334	0.2245301	-0.0688375	0.8113043	-0.0923585	0.8348253
H vs D	0.3749031	0.1851566	0.0120029	0.7378033	-0.0081916	0.7579978
K vs D	0.2259307	0.1189247	-0.0071575	0.4590189	-0.0223969	0.4742582
K vs D	0.3499839	0.1602586	0.0358828	0.6640849	0.0176824	0.6822853
I vs D	0.4293291	0.1325441	0.1695474	0.6891108	0.1533993	0.705259
KL vs D	0.2996797	0.1649031	-0.0235244	0.6228838	-0.042088	0.6414474
KM vs D	0.1397534	0.127592	-0.1103222	0.3898291	-0.1261304	0.4056373
L vs D	0.3962816	0.1329957	0.1356149	0.6569483	0.1194353	0.6731279
LM vs D	0.1872161	0.0938029	0.0033657	0.3710664	-0.0104874	0.3849196
DLM vs DL	0.1443789	0.1985192	-0.2447117	0.5334694	-0.2660159	0.5547737
EK vs DL	0.0939743	0.1041967	-0.1102473	0.298196	-0.1246165	0.3125651
F vs DL	-0.1220943	0.0916092	-0.3016451	0.0574565	-0.3154027	0.071214
FG vs DL	0.1359279	0.1098153	-0.079306	0.3511619	-0.0939906	0.3658465
FH vs DL	0.0342222	0.1308741	-0.2222863	0.2907307	-0.2383187	0.3067631
FJ vs DL	0.0212119	0.1389012	-0.2510295	0.2934534	-0.2676277	0.3100516
G vs DL	-0.0290273	0.0940702	-0.2134016	0.155347	-0.2272667	0.1692122
GL vs DL	0.0927656	0.2061899	-0.3113592	0.4968905	-0.3333101	0.5188414
H vs DL	0.0964354	0.1624299	-0.2219214	0.4147921	-0.240291	0.4331618
K vs DL	-0.0525371	0.0849005	-0.2189389	0.1138648	-0.2324392	0.1273651
K vs DL	0.0715161	0.1087193	-0.1415699	0.2846021	-0.1561912	0.2992234
I vs DL	0.1508614	0.1031443	-0.0512977	0.3530205	-0.0656104	0.3673331
KL vs DL	0.0212119	0.1389012	-0.2510295	0.2934534	-0.2676277	0.3100516
KM vs DL	-0.1387143	0.0491569	-0.23506	-0.0423686	-0.2488004	-0.0286282
L vs DL	0.1178138	0.1045025	-0.0870074	0.3226351	-0.1013931	0.3370208
LM vs DL	-0.0912517	0.0985665	-0.2844385	0.1019352	-0.298516	0.1160127

Table A7. Prediction interval for total effective rate (continued).

Comparison	Effect Size	Standard Error	LCI	UCI	LPrI	UPrI
EK vs DLM	-0.0504046	0.1966924	-0.4359146	0.3351055	-0.4570659	0.3562567
F vs DLM	-0.2664732	0.1923789	-0.6435289	0.1105824	-0.6643203	0.1313739
FG vs DLM	-0.008451	0.2022497	-0.4048532	0.3879512	-0.4264711	0.4095692
FH vs DLM	-0.1101567	0.2142972	-0.5301715	0.3098581	-0.5528127	0.3324992
FJ vs DLM	-0.1231669	0.2192916	-0.5529706	0.3066367	-0.57604	0.3297062
G vs DLM	-0.1734062	0.1940597	-0.5537563	0.206944	-0.5746877	0.2278753
GL vs DLM	-0.0516133	0.2670384	-0.574999	0.4717725	-0.602255	0.4990284
H vs DLM	-0.0479435	0.2349015	-0.508342	0.412455	-0.5327635	0.4368764
K vs DLM	-0.196916	0.1871872	-0.563796	0.1699642	-0.5841577	0.1903258
K vs DLM	-0.0728628	0.2158211	-0.4958643	0.3501388	-0.5186359	0.3729104
I vs DLM	0.0064825	0.1961221	-0.3779098	0.3908748	-0.3990133	0.4119783
KL vs DLM	-0.1231669	0.2192916	-0.5529706	0.3066367	-0.57604	0.3297062
KM vs DLM	-0.2830932	0.1928098	-0.6609935	0.0948071	-0.6818208	0.1156344
L vs DLM	-0.0265651	0.1964284	-0.4115576	0.3584275	-0.4326867	0.3795566
LM vs DLM	-0.2356306	0.1723242	-0.5733797	0.1021186	-0.5925327	0.1212716
F vs EK	-0.2160687	0.0852841	-0.3832223	-0.048915	-0.3967358	-0.0354015
FG vs EK	0.0419536	0.1077651	-0.1692622	0.2531694	-0.1838291	0.2677362
FH vs EK	-0.0597522	0.1287362	-0.3120704	0.1925661	-0.3279563	0.2084519
FJ vs EK	-0.0727624	0.1368887	-0.3410594	0.1955345	-0.3575135	0.2119887
G vs EK	-0.1230016	0.0912473	-0.301843	0.0558397	-0.3155853	0.0695821
GL vs EK	-0.0012087	0.2048396	-0.4026869	0.4002694	-0.4245235	0.4221061
H vs EK	0.002461	0.1607123	-0.3125293	0.3174513	-0.3307649	0.335687
K vs EK	-0.1465114	0.0604064	-0.2649058	-0.028117	-0.2781422	-0.0148806
K vs EK	-0.0224582	0.1339166	-0.2849299	0.2400135	-0.301174	0.2562575
I vs EK	0.056887	0.0840658	-0.107879	0.2216531	-0.1213514	0.2351255
KL vs EK	-0.0727624	0.1368887	-0.3410593	0.1955345	-0.3575135	0.2119887
KM vs EK	-0.2326887	0.0923612	-0.4137133	-0.051664	-0.4275031	-0.0378742
L vs EK	0.0238395	0.1172592	-0.2059844	0.2536634	-0.2211187	0.2687977
LM vs EK	-0.185226	0.0948334	-0.3710961	0.0006441	-0.3849959	0.0145439
FG vs F	0.2580222	0.0905592	0.0805294	0.4355151	0.0668157	0.4492288
FH vs F	0.1563165	0.1146972	-0.0684858	0.3811189	-0.0834617	0.3960947
FJ vs F	0.1433063	0.1237779	-0.0992939	0.3859065	-0.1148474	0.40146
G vs F	0.0930671	0.0700716	-0.0442708	0.2304049	-0.0574489	0.243583
GL vs F	0.21486	0.1963204	-0.1699209	0.5996408	-0.191041	0.6207609
H vs F	0.2185297	0.1497027	-0.0748821	0.5119416	-0.0922752	0.5293346
K vs F	0.0695573	0.0602043	-0.048441	0.1875556	-0.0616817	0.2007962
K vs F	0.1936105	0.1243413	-0.0500939	0.4373148	-0.0656847	0.4529056
I vs F	0.2729557	0.0839162	0.1084831	0.4374284	0.0950155	0.4508959
KL vs F	0.1433063	0.1237779	-0.0992939	0.3859065	-0.1148474	0.40146
KM vs F	-0.01662	0.0778316	-0.1691671	0.1359271	-0.1824645	0.1492245
L vs F	0.2399082	0.107973	0.0282851	0.4515313	0.0137064	0.46611
LM vs F	0.0308427	0.0855296	-0.1367923	0.1984776	-0.1503143	0.2119996
FH vs FG	-0.1017057	0.1292205	-0.3549733	0.1515618	-0.3708921	0.1674807
FJ vs FG	-0.114716	0.1373443	-0.3839059	0.1544739	-0.4003925	0.1709606

Table A7. Prediction interval for total effective rate (continued).

Comparison	Effect Size	Standard Error	LCI	UCI	LPrI	UPrI
G vs FG	-0.1649552	0.0573503	-0.2773598	-0.0525506	-0.2906776	-0.0392328
GL vs FG	-0.0431623	0.2051443	-0.4452377	0.3589132	-0.4671002	0.3807756
H vs FG	-0.0394926	0.1611005	-0.3552438	0.2762587	-0.3735096	0.2945246
K vs FG	-0.188465	0.0892441	-0.3633803	-0.0135497	-0.3770409	0.0001109
K vs FG	-0.0644118	0.1387449	-0.3363468	0.2075232	-0.3529338	0.2241102
I vs FG	0.0149335	0.1067281	-0.1942499	0.2241168	-0.2087583	0.2386252
KL vs FG	-0.114716	0.1373443	-0.3839059	0.1544739	-0.4003925	0.1709606
KM vs FG	-0.2746422	0.0992329	-0.4691351	-0.0801494	-0.4832457	-0.0660387
L vs FG	-0.0181141	0.1245027	-0.2621348	0.2259066	-0.2777362	0.241508
LM vs FG	-0.2271796	0.1058805	-0.4347015	-0.0196577	-0.4491628	-0.0051964
FJ vs FH	-0.0130102	0.1542399	-0.3153149	0.2892944	-0.3330517	0.3070312
G vs FH	-0.0632495	0.1157031	-0.2900234	0.1635244	-0.305061	0.1785621
GL vs FH	0.0585435	0.2168195	-0.3664149	0.4835018	-0.3892721	0.506359
H vs FH	0.0622132	0.1757267	-0.2822048	0.4066312	-0.3016313	0.4260577
K vs FH	-0.0867592	0.1136848	-0.3095773	0.1360588	-0.3244915	0.150973
K vs FH	0.0372939	0.155411	-0.2673061	0.3418939	-0.2851323	0.3597202
I vs FH	0.1166392	0.1278213	-0.133886	0.3671644	-0.1497097	0.3829881
KL vs FH	-0.0130102	0.1542399	-0.3153149	0.2892944	-0.3330517	0.3070312
KM vs FH	-0.1729365	0.121448	-0.4109701	0.0650971	-0.4263714	0.0804984
L vs FH	0.0835917	0.1430231	-0.1967284	0.3639117	-0.2136258	0.3808091
LM vs FH	-0.1254738	0.1273932	-0.3751599	0.1242123	-0.3909547	0.140007
G vs FJ	-0.0502392	0.1247106	-0.2946674	0.194189	-0.3102826	0.2098042
GL vs FJ	0.0715537	0.2217571	-0.3630822	0.5061895	-0.3863639	0.5294713
H vs FJ	0.0752234	0.1817839	-0.2810665	0.4315133	-0.3009848	0.4514316
K vs FJ	-0.073749	0.1228403	-0.3145116	0.1670136	-0.3300035	0.1825055
K vs FJ	0.0503042	0.1622286	-0.2676579	0.3682663	-0.2860118	0.3866202
I vs FJ	0.1296494	0.1360287	-0.136962	0.3962608	-0.153355	0.4126539
KL vs FJ	9.13E-14	0.161107	-0.315764	0.315764	-0.3340303	0.3340303
KM vs FJ	-0.1599263	0.1300582	-0.4148356	0.0949831	-0.4308118	0.1109593
L vs FJ	0.0966019	0.1504032	-0.1981829	0.3913867	-0.2156287	0.4088325
LM vs FJ	-0.1124636	0.1356265	-0.3782867	0.1533595	-0.3946512	0.169724
GL vs G	0.1217929	0.1969098	-0.2641431	0.5077289	-0.2853125	0.5288983
H vs G	0.1254626	0.1504747	-0.1694624	0.4203877	-0.1869136	0.4378389
K vs G	-0.0235098	0.0683906	-0.157553	0.1105334	-0.1707227	0.1237031
K vs G	0.1005434	0.126184	-0.1467727	0.3478595	-0.1624861	0.3635729
I vs G	0.1798887	0.0899719	0.0035469	0.3562304	-0.0101428	0.3699201
KL vs G	0.0502392	0.1247106	-0.194189	0.2946674	-0.2098042	0.3102826
KM vs G	-0.109687	0.0807429	-0.2679403	0.0485662	-0.2813117	0.0619377
L vs G	0.1468411	0.1104994	-0.0697338	0.363416	-0.0844583	0.3781405
LM vs G	-0.0622244	0.0892464	-0.2371442	0.1126954	-0.2508048	0.126356
H vs GL	0.0036697	0.2372048	-0.4612432	0.4685826	-0.4858656	0.4932051
K vs GL	-0.1453027	0.1957306	-0.5289276	0.2383222	-0.5499984	0.259393
K vs GL	-0.0212495	0.2225732	-0.457485	0.414986	-0.480837	0.438338
I vs GL	0.0580957	0.2042658	-0.342258	0.4584495	-0.3640461	0.4802376

Table A7. Prediction interval for total effective rate (continued).

Comparison	Effect Size	Standard Error	LCI	UCI	LPrI	UPrI
KL vs GL	-0.0715537	0.2217571	-0.5061895	0.3630822	-0.5294713	0.3863639
KM vs GL	-0.2314799	0.2003393	-0.6241378	0.161178	-0.645595	0.1826351
L vs GL	0.0250482	0.2141071	-0.394594	0.4446904	-0.4172189	0.4673153
LM vs GL	-0.1840173	0.2039982	-0.5838465	0.2158119	-0.605612	0.2375774
K vs H	-0.1489724	0.1489284	-0.4408667	0.1429218	-0.4582017	0.1602568
K vs H	-0.0249192	0.1827786	-0.3831587	0.3333203	-0.4031583	0.3533198
I vs H	0.054426	0.1599804	-0.2591298	0.3679819	-0.2773086	0.3861606
KL vs H	-0.0752234	0.1817839	-0.4315133	0.2810665	-0.4514316	0.3009848
KM vs H	-0.2351497	0.1549356	-0.5388179	0.0685186	-0.5566078	0.0863085
L vs H	0.0213785	0.1723689	-0.3164584	0.3592153	-0.3356149	0.3783719
LM vs H	-0.187687	0.1596386	-0.5005729	0.1251988	-0.5187251	0.143351
K vs K	0.1240532	0.1195192	-0.1102002	0.3583066	-0.1254774	0.3735838
I vs K	0.2033984	0.0584648	0.0888095	0.3179874	0.0755255	0.3312714
KL vs K	0.073749	0.1228403	-0.1670136	0.3145116	-0.1825055	0.3300035
KM vs K	-0.0861772	0.0698698	-0.2231195	0.050765	-0.2362962	0.0639417
L vs K	0.1703509	0.1005032	-0.0266317	0.3673335	-0.0408065	0.3815083
LM vs K	-0.0387146	0.0731061	-0.182	0.1045708	-0.19521	0.1177807
I vs K	0.0793453	0.1330269	-0.1813827	0.3400733	-0.1975646	0.3562551
KL vs K	-0.0503042	0.1622286	-0.3682663	0.2676579	-0.3866202	0.2860118
KM vs K	-0.2102304	0.0969715	-0.4002911	-0.0201697	-0.4142911	-0.0061697
L vs K	0.0462977	0.1341261	-0.2165846	0.30918	-0.2328433	0.3254387
LM vs K	-0.1627678	0.1299394	-0.4174444	0.0919088	-0.4334125	0.1078769
KL vs I	-0.1296494	0.1360287	-0.3962608	0.136962	-0.4126539	0.153355
KM vs I	-0.2895757	0.0910665	-0.4680628	-0.1110886	-0.4817975	-0.0973538
L vs I	-0.0330475	0.1162683	-0.2609292	0.1948341	-0.2760018	0.2099067
LM vs I	-0.242113	0.0936449	-0.4256536	-0.0585724	-0.4394997	-0.0447263
KM vs KL	-0.1599263	0.1300582	-0.4148356	0.0949831	-0.4308118	0.1109593
L vs KL	0.0966019	0.1504032	-0.1981829	0.3913867	-0.2156287	0.4088325
LM vs KL	-0.1124636	0.1356265	-0.3782867	0.1533595	-0.3946512	0.169724
L vs KM	0.2565281	0.0926634	0.0749113	0.438145	0.0611084	0.4519479
LM vs KM	0.0474626	0.0864936	-0.1220617	0.216987	-0.135618	0.2305433
LM vs L	-0.2090655	0.0942819	-0.3938546	-0.0242764	-0.4077293	-0.0104017

Table A8. Prediction interval for PSQI.

Comparison	Effect Size	Standard Error	LCI	UCI	LPrI	UPrI
AB vs A	-2.204483	0.6065595	-3.393318	-1.015648	-5.933261	1.524295
ABGN vs A	-4.96836	2.029365	-8.945842	-0.9908785	-10.40818	0.4714562
ACD vs A	-1.54	1.769818	-5.00878	1.92878	-6.586477	3.506477
AE vs A	-2.14107	1.029984	-4.159801	-0.1223388	-6.240141	1.958001
AF vs A	-3.159136	1.044269	-5.205865	-1.112407	-7.273297	0.9550251
AG vs A	-1.692011	1.027836	-3.706532	0.3225111	-5.788826	2.404804
AH vs A	-1.991131	1.255163	-4.451206	0.4689435	-6.344842	2.36258
AJ vs A	-0.8699999	1.723862	-4.248706	2.508707	-5.84949	4.10949
AK vs A	-3.816551	1.637466	-7.025925	-0.6071766	-8.672534	1.039432
AL vs A	-1.492981	0.7615689	-2.985629	-0.0003338	-5.338872	2.35291
AM vs A	-2.48	1.789532	-5.987419	1.027418	-7.555475	2.595474
B vs A	4.894544	3.116283	-1.213258	11.00235	-2.384638	12.17373
BE vs A	3.088497	3.564116	-3.897042	10.07404	-5.004752	11.18175
BF vs A	2.563649	3.622802	-4.536912	9.66421	-5.637872	10.76517
BL vs A	-0.0098825	2.553327	-5.014311	4.994546	-6.30556	6.285795
BM vs A	-8.209201	2.590988	-13.28745	-3.130957	-14.56892	-1.849486
D vs A	-2.700785	2.836056	-8.259353	2.857783	-9.4838	4.08223
DL vs A	-1.536478	2.265718	-5.977203	2.904248	-7.353518	4.280563
EK vs A	-5.94769	2.033475	-9.933228	-1.962152	-11.39392	-0.5014569
F vs A	-3.142401	1.094805	-5.288179	-0.9966229	-7.311154	1.026352
FG vs A	-3.311761	1.466478	-6.186005	-0.4375175	-7.933506	1.309983
FJ vs A	-2.47	1.775838	-5.950578	1.010578	-7.525315	2.585314
G vs A	-1.764333	0.695626	-3.127735	-0.4009312	-5.557608	2.028941
H vs A	-0.9300003	1.763522	-4.38644	2.526439	-5.967248	4.107248
K vs A	-2.94899	0.99294	-4.895116	-1.002863	-7.009645	1.111666
KL vs A	-4.507834	1.985019	-8.3984	-0.6172677	-9.87877	0.8631019
L vs A	1.38726	1.902696	-2.341955	5.116475	-3.857565	6.632085
M vs A	-5.344955	2.243428	-9.741994	-0.9479172	-11.12575	0.4358355
ABGN vs AB	-2.763877	2.117886	-6.914857	1.387103	-8.343058	2.815303
ACD vs AB	0.6644829	1.870874	-3.002363	4.331329	-4.532237	5.861203
AE vs AB	0.0634126	1.19529	-2.279312	2.406138	-4.219237	4.346062
AF vs AB	-0.9546531	1.207696	-3.321693	1.412387	-5.251837	3.34253
AG vs AB	0.5124723	1.193445	-1.826637	2.851582	-3.768025	4.79297
AH vs AB	0.2133515	1.394038	-2.518914	2.945616	-4.313651	4.740354
AJ vs AB	1.334483	1.827461	-2.247274	4.916241	-3.797212	6.466178
AK vs AB	-1.612068	1.745958	-5.034082	1.809946	-6.623658	3.399521
AL vs AB	0.7115014	0.973638	-1.196794	2.619797	-3.329556	4.752558
AM vs AB	-0.2755171	1.889534	-3.978936	3.427902	-5.500401	4.949367
B vs AB	7.099027	3.174824	0.8764867	13.32157	-0.2852148	14.48327
BE vs AB	5.29298	3.615417	-1.793108	12.37907	-2.894899	13.48086
BF vs AB	4.768132	3.67328	-2.431365	11.96763	-3.526775	13.06304
BL vs AB	2.1946	2.624439	-2.949205	7.338406	-4.222231	8.611432
BM vs AB	-6.004718	2.660925	-11.22004	-0.7894007	-12.4841	0.4746634
D vs AB	-0.496302	2.900114	-6.18042	5.187817	-7.391685	6.399081

Table A8. Prediction interval for PSQI (continued).

Comparison	Effect Size	Standard Error	LCI	UCI	LPrI	UPrI
DL vs AB	0.6680053	2.345558	-3.929203	5.265213	-5.279933	6.615944
EK vs AB	-3.743208	2.121824	-7.901907	0.4154916	-9.328644	1.842229
F vs AB	-0.9379184	1.251478	-3.390769	1.514933	-5.287189	3.411352
FG vs AB	-1.107279	1.587097	-4.217931	2.003374	-5.892797	3.678239
FJ vs AB	-0.2655174	1.87657	-3.943527	3.412492	-5.47082	4.939785
G vs AB	0.4401497	0.9230908	-1.369075	2.249374	-3.550978	4.431278
H vs AB	1.274483	1.864919	-2.380692	4.929657	-3.913276	6.462242
K vs AB	-0.7445068	1.163208	-3.024354	1.53534	-4.990044	3.501031
KL vs AB	-2.303351	2.075404	-6.371068	1.764366	-7.815347	3.208645
L vs AB	3.591743	1.997085	-0.3224712	7.505957	-1.79787	8.981356
M vs AB	-3.140473	2.323885	-7.695203	1.414258	-9.052719	2.771774
ACD vs ABGN	3.42836	2.692689	-1.849214	8.705935	-3.105683	9.962402
AE vs ABGN	2.82729	2.27574	-1.63308	7.287659	-3.006094	8.660673
AF vs ABGN	1.809224	2.282362	-2.664123	6.28257	-4.03497	7.653418
AG vs ABGN	3.276349	2.274778	-1.182134	7.734833	-2.555464	9.108163
AH vs ABGN	2.977228	2.386155	-1.699549	7.654006	-3.037878	8.992334
AJ vs ABGN	4.09836	2.662709	-1.120454	9.317174	-2.384086	10.58081
AK vs ABGN	1.151809	2.197371	-3.154959	5.458577	-4.554504	6.858121
AL vs ABGN	3.475379	2.167607	-0.7730523	7.723809	-2.183115	9.133872
AM vs ABGN	2.48836	2.705688	-2.814691	7.79141	-4.068105	9.044825
B vs ABGN	9.862904	3.718961	2.573875	17.15193	1.483285	18.24252
BE vs ABGN	8.056857	4.101521	0.0180225	16.09569	-1.038891	17.15261
BF vs ABGN	7.532009	4.152608	-0.6069546	15.67097	-1.66019	16.72421
BL vs ABGN	4.958477	3.261697	-1.434331	11.35129	-2.582476	12.49943
BM vs ABGN	-3.240841	3.191959	-9.496965	3.015282	-10.65592	4.174235
D vs ABGN	2.267575	3.394018	-4.384578	8.919728	-5.513805	10.04896
DL vs ABGN	3.431882	3.041801	-2.529938	9.393703	-3.7143	10.57806
EK vs ABGN	-0.9793308	2.506459	-5.891899	3.933237	-7.195728	5.237066
F vs ABGN	1.825959	2.1613	-2.410111	6.062028	-3.822434	7.474351
FG vs ABGN	1.656598	2.504018	-3.251186	6.564383	-4.555683	7.86888
FJ vs ABGN	2.49836	2.69665	-2.786977	7.783696	-4.042511	9.03923
G vs ABGN	3.204027	2.14548	-1.001038	7.409091	-2.419083	8.827136
H vs ABGN	4.03836	2.688555	-1.231112	9.307832	-2.488558	10.56528
K vs ABGN	2.01937	1.769942	-1.449652	5.488392	-3.027288	7.066028
KL vs ABGN	0.4605257	2.469378	-4.379366	5.300418	-5.693482	6.614533
L vs ABGN	6.35562	2.781928	0.9031409	11.8081	-0.3329743	13.04421
M vs ABGN	-0.3765956	2.916938	-6.093689	5.340497	-7.3016	6.548408
AE vs ACD	-0.6010703	2.047712	-4.614511	3.41237	-6.069571	4.86743
AF vs ACD	-1.619136	2.054934	-5.646732	2.40846	-7.098957	3.860685
AG vs ACD	-0.1520106	2.046632	-4.163336	3.859314	-5.61882	5.314799
AH vs ACD	-0.4511315	2.169721	-4.703707	3.801444	-6.113014	5.210751
AJ vs ACD	0.6700001	2.470618	-4.172323	5.512323	-5.486089	6.826089
AK vs ACD	-2.276551	2.411131	-7.00228	2.449178	-8.333175	3.780072
AL vs ACD	0.0470185	1.926718	-3.72928	3.823317	-5.234363	5.3284

Table A8. Prediction interval for PSQI (continued).

Comparison	Effect Size	Standard Error	LCI	UCI	LPrI	UPrI
AM vs ACD	-0.9400001	2.516879	-5.872993	3.992993	-7.173984	5.293984
B vs ACD	6.434544	3.583779	-0.5895343	13.45862	-1.694946	14.56403
BE vs ACD	4.628497	3.979344	-3.170874	12.42787	-4.237325	13.49432
BF vs ACD	4.103649	4.031991	-3.798908	12.00621	-4.861119	13.06842
BL vs ACD	1.530118	3.106724	-4.55895	7.619185	-5.731953	8.792189
BM vs ACD	-6.669201	3.13775	-12.81908	-0.5193241	-13.98686	0.6484545
D vs ACD	-1.160785	3.342973	-7.712892	5.391322	-8.849179	6.527609
DL vs ACD	0.0035224	2.875019	-5.631411	5.638455	-6.847761	6.854805
EK vs ACD	-4.407691	2.695789	-9.691339	0.8759584	-10.94708	2.131696
F vs ACD	-1.602401	2.08107	-5.681224	2.476422	-7.123327	3.918524
FG vs ACD	-1.771762	2.298437	-6.276616	2.733092	-7.64225	4.098727
FJ vs ACD	-0.9300003	2.507161	-5.843945	3.983945	-7.147582	5.287581
G vs ACD	-0.2243332	1.901618	-3.951436	3.50277	-5.467524	5.018857
H vs ACD	0.6099997	2.498453	-4.286877	5.506876	-5.592902	6.812901
K vs ACD	-1.40899	2.029331	-5.386406	2.568426	-6.848754	4.030775
KL vs ACD	-2.967834	2.659428	-8.180217	2.244549	-9.444644	3.508976
L vs ACD	2.92726	2.598558	-2.165821	8.020341	-3.445362	9.299881
M vs ACD	-3.804955	2.857486	-9.405524	1.795614	-10.62549	3.015577
AF vs AE	-1.018066	1.466763	-3.892868	1.856736	-5.640188	3.604056
AG vs AE	0.4490597	1.455095	-2.402874	3.300993	-4.15761	5.05573
AH vs AE	0.1499388	1.623669	-3.032393	3.332271	-4.686626	4.986504
AJ vs AE	1.27107	2.008125	-2.664782	5.206923	-4.135674	6.677815
AK vs AE	-1.675481	1.934414	-5.466863	2.115901	-6.968616	3.617655
AL vs AE	0.6480888	1.280965	-1.862556	3.158734	-3.736944	5.033122
AM vs AE	-0.3389298	2.064774	-4.385813	3.707953	-5.834201	5.156342
B vs AE	7.035614	3.282099	0.6028184	13.46841	-0.5422767	14.61351
BE vs AE	5.229567	3.709971	-2.041843	12.50098	-3.133367	13.5925
BF vs AE	4.704719	3.766384	-2.677259	12.0867	-3.763037	13.17247
BL vs AE	2.131188	2.753255	-3.265094	7.527469	-4.507594	8.769969
BM vs AE	-6.068131	2.788178	-11.53286	-0.6034018	-12.7676	0.6313406
D vs AE	-0.5597147	3.017278	-6.473471	5.354042	-7.662276	6.542847
DL vs AE	0.6045927	2.488856	-4.273476	5.482661	-5.58215	6.791335
EK vs AE	-3.80662	2.279407	-8.274176	0.6609347	-9.645988	2.032748
F vs AE	-1.001331	1.503126	-3.947404	1.944742	-5.672064	3.669403
FG vs AE	-1.170691	1.792072	-4.683088	2.341706	-6.249913	3.908531
FJ vs AE	-0.32893	2.052917	-4.352572	3.694712	-5.805587	5.147727
G vs AE	0.376737	1.242913	-2.059327	2.812801	-3.962248	4.715722
H vs AE	1.21107	2.042272	-2.79171	5.21385	-4.248915	6.671055
K vs AE	-0.8079194	1.430596	-3.611836	1.995997	-5.38238	3.766541
KL vs AE	-2.366764	2.236279	-6.749791	2.016263	-8.135957	3.402429
L vs AE	3.52833	2.163599	-0.7122464	7.768907	-2.123744	9.180405
M vs AE	-3.203885	2.468548	-8.04215	1.63438	-9.3565	2.948729
AG vs AF	1.467125	1.465253	-1.404718	4.338968	-3.152993	6.087244
AH vs AF	1.168005	1.632769	-2.032164	4.368173	-3.681358	6.017368

Table A8. Prediction interval for PSQI (continued).

Comparison	Effect Size	Standard Error	LCI	UCI	LPrI	UPrI
AJ vs AF	2.289136	2.015489	-1.66115	6.239422	-3.129058	7.707331
AK vs AF	-0.657415	1.942207	-4.464072	3.149242	-5.962475	4.647645
AL vs AF	1.666155	1.29246	-0.8670206	4.19933	-2.732965	6.065275
AM vs AF	0.679136	2.071937	-3.381785	4.740057	-4.827401	6.185673
B vs AF	8.05368	3.286571	1.61212	14.49524	0.4676866	15.63967
BE vs AF	6.247633	3.713924	-1.031525	13.52679	-2.122637	14.6179
BF vs AF	5.722785	3.770281	-1.666831	13.1124	-2.752222	14.19779
BL vs AF	3.149254	2.758595	-2.257494	8.556001	-3.498794	9.797301
BM vs AF	-5.050065	2.793563	-10.52535	0.4252172	-11.75891	1.658782
D vs AF	0.4583511	3.022238	-5.465126	6.381828	-6.653026	7.569728
DL vs AF	1.622658	2.494767	-3.266995	6.512311	-4.574035	7.819351
EK vs AF	-2.788555	2.286018	-7.269067	1.691958	-8.638722	3.061614
F vs AF	0.0167348	1.513022	-2.948735	2.982204	-4.667346	4.700815
FG vs AF	-0.1526255	1.800242	-3.681036	3.375785	-5.243919	4.938668
FJ vs AF	0.6891357	2.06012	-3.348626	4.726898	-4.798825	6.177097
G vs AF	1.394803	1.254697	-1.064357	3.853963	-2.958346	5.747951
H vs AF	2.229136	2.049514	-1.787837	6.246109	-3.242188	7.700459
K vs AF	0.2101463	1.441104	-2.614367	3.034659	-4.37809	4.798383
KL vs AF	-1.348698	2.243036	-5.744967	3.047571	-7.128852	4.431456
L vs AF	4.546396	2.170407	0.2924768	8.800315	-1.116585	10.20938
M vs AF	-2.185819	2.474604	-7.035954	2.664316	-8.348602	3.976963
AH vs AG	-0.2991209	1.622307	-3.478785	2.880543	-5.133774	4.535533
AJ vs AG	0.8220106	2.007024	-3.111684	4.755706	-4.583024	6.227046
AK vs AG	-2.124541	1.933282	-5.913705	1.664623	-7.415947	3.166866
AL vs AG	0.1990291	1.279237	-2.308229	2.706288	-4.183894	4.581952
AM vs AG	-0.7879895	2.063704	-4.832774	3.256795	-6.281579	4.7056
B vs AG	6.586555	3.281423	0.1550838	13.01803	-0.9901116	14.16322
BE vs AG	4.780508	3.709373	-2.489729	12.05075	-3.581316	13.14233
BF vs AG	4.25566	3.765795	-3.125163	11.63648	-4.210999	12.72232
BL vs AG	1.682128	2.75245	-3.712575	7.076831	-4.955256	8.319512
BM vs AG	-6.517191	2.787391	-11.98038	-1.054004	-13.21529	0.1809106
D vs AG	-1.008774	3.01655	-6.921103	4.903554	-8.110042	6.092493
DL vs AG	0.155533	2.487965	-4.720789	5.031856	-6.029711	6.340777
EK vs AG	-4.25568	2.278446	-8.721353	0.209992	-10.09348	1.582119
F vs AG	-1.450391	1.501661	-4.393591	1.49281	-6.119152	3.21837
FG vs AG	-1.619751	1.790833	-5.129718	1.890216	-6.697144	3.457642
FJ vs AG	-0.7779897	2.05184	-4.799522	3.243542	-6.252959	4.696979
G vs AG	-0.0723227	1.241127	-2.504888	2.360242	-4.409169	4.264524
H vs AG	0.7620102	2.04119	-3.238648	4.762669	-4.696281	6.220302
K vs AG	-1.256979	1.429065	-4.057895	1.543936	-5.829437	3.315479
KL vs AG	-2.815824	2.235302	-7.196934	1.565287	-8.583431	2.951784
L vs AG	3.07927	2.162575	-1.159299	7.31784	-2.571165	8.729706
M vs AG	-3.652945	2.467657	-8.489464	1.183574	-9.804065	2.498174
AJ vs AH	1.121132	2.132401	-3.058298	5.30056	-4.481128	6.723391

Table A8. Prediction interval for PSQI (continued).

Comparison	Effect Size	Standard Error	LCI	UCI	LPrI	UPrI
AK vs AH	-1.82542	2.063179	-5.869177	2.218338	-7.318185	3.667346
AL vs AH	0.49815	1.468136	-2.379344	3.375644	-4.125796	5.122096
AM vs AH	-0.4888686	2.185832	-4.77302	3.795283	-6.176612	5.198875
B vs AH	6.885675	3.359563	0.3010531	13.4703	-0.8329074	14.60426
BE vs AH	5.079628	3.778672	-2.326434	12.48569	-3.410996	13.57025
BF vs AH	4.55478	3.834076	-2.959871	12.06943	-4.039111	13.14867
BL vs AH	1.981249	2.845157	-3.595157	7.557655	-4.81769	8.780189
BM vs AH	-6.21807	2.879	-11.86081	-0.5753341	-13.07634	0.6402022
D vs AH	-0.7096535	3.101393	-6.788271	5.368965	-7.962186	6.542879
DL vs AH	0.4546538	2.590158	-4.621963	5.53127	-5.903646	6.812953
EK vs AH	-3.956559	2.389652	-8.640191	0.7270727	-9.977469	2.064352
F vs AH	-1.15127	1.66554	-4.415669	2.113129	-6.047029	3.744489
FG vs AH	-1.32063	1.930286	-5.10392	2.46266	-6.607458	3.966198
FJ vs AH	-0.4788688	2.174634	-4.741074	3.783336	-6.14863	5.190893
G vs AH	0.2267982	1.435039	-2.585827	3.039424	-4.35348	4.807076
H vs AH	1.061131	2.164589	-3.181385	5.303647	-4.592528	6.71479
K vs AH	-0.9578583	1.60042	-4.094625	2.178908	-5.761901	3.846184
KL vs AH	-2.516703	2.348556	-7.119787	2.086382	-8.469588	3.436183
L vs AH	3.378391	2.279406	-1.089162	7.845944	-2.460975	9.217757
M vs AH	-3.353824	2.570679	-8.392263	1.684615	-9.678971	2.971323
AK vs AJ	-2.946551	2.377603	-7.606566	1.713464	-8.947474	3.054372
AL vs AJ	-0.6229815	1.884592	-4.316713	3.07075	-5.840394	4.59443
AM vs AJ	-1.61	2.484778	-6.480076	3.260076	-7.789883	4.569883
B vs AJ	5.764544	3.561308	-1.215492	12.74458	-2.323534	13.85262
BE vs AJ	3.958497	3.959119	-3.801233	11.71823	-4.869368	12.78636
BF vs AJ	3.433649	4.012031	-4.429788	11.29708	-5.493582	12.36088
BL vs AJ	0.8601174	3.080775	-5.178092	6.898326	-6.355565	8.0758
BM vs AJ	-7.339201	3.11206	-13.43873	-1.239675	-14.61082	-0.0675796
D vs AJ	-1.830785	3.318872	-8.335655	4.674085	-9.475378	5.813807
DL vs AJ	-0.6664777	2.846959	-6.246415	4.913459	-7.468571	6.135615
EK vs AJ	-5.077691	2.665843	-10.30265	0.1472662	-11.56552	1.410142
F vs AJ	-2.272401	2.04213	-6.274903	1.730101	-7.732164	3.187362
FG vs AJ	-2.441761	2.26324	-6.877631	1.994108	-8.254766	3.371243
FJ vs AJ	-1.6	2.474934	-6.450782	3.250781	-7.763337	4.563336
G vs AJ	-0.8943333	1.858923	-4.537755	2.749089	-6.073082	4.284416
H vs AJ	-0.0600004	2.466112	-4.893491	4.77349	-6.208527	6.088526
K vs AJ	-2.07899	1.989379	-5.978101	1.820121	-7.456669	3.298689
KL vs AJ	-3.637834	2.629068	-8.790713	1.515045	-10.06259	2.78692
L vs AJ	2.25726	2.567479	-2.774907	7.289426	-4.062448	8.576967
M vs AJ	-4.474956	2.829252	-10.02019	1.070277	-11.24607	2.296164
AL vs AK	2.32357	1.805964	-1.216055	5.863194	-2.776192	7.423332
AM vs AK	1.336551	2.425638	-3.417613	6.090714	-4.744255	7.417357
B vs AK	8.711095	3.52047	1.8111	15.61109	0.6981516	16.72404
BE vs AK	6.905048	3.922439	-0.7827913	14.59289	-1.854057	15.66415

Table A8. Prediction interval for PSQI (continued).

Comparison	Effect Size	Standard Error	LCI	UCI	LPrI	UPrI
BF vs AK	6.3802	3.975827	-1.412278	14.17268	-2.479019	15.23942
BL vs AK	3.806669	3.033429	-2.138744	9.752081	-3.324612	10.93795
BM vs AK	-4.39265	2.958299	-10.19081	1.40551	-11.39066	2.605362
D vs AK	1.115766	3.175267	-5.107643	7.339176	-6.269273	8.500806
DL vs AK	2.280073	2.795635	-3.19927	7.759417	-4.432384	8.99253
EK vs AK	-2.13114	2.201224	-6.445459	2.18318	-7.84366	3.581381
F vs AK	0.6741498	1.79839	-2.85063	4.198929	-4.414404	5.762703
FG vs AK	0.5047895	2.198443	-3.80408	4.813659	-5.20325	6.212829
FJ vs AK	1.346551	2.415553	-3.387846	6.080947	-4.717439	7.41054
G vs AK	2.052218	1.779357	-1.435259	5.539694	-3.008271	7.112707
H vs AK	2.886551	2.406513	-1.830128	7.60323	-3.162386	8.935487
K vs AK	0.8675613	1.302209	-1.684721	3.419843	-3.543568	5.278691
KL vs AK	-0.6912831	2.158902	-4.922654	3.540088	-6.33584	4.953273
L vs AK	5.203811	2.510405	0.2835079	10.12411	-1.019243	11.42686
M vs AK	-1.528404	2.659228	-6.740396	3.683587	-8.004871	4.948062
AM vs AL	-0.9870186	1.944843	-4.79884	2.824803	-6.296115	4.322078
B vs AL	6.387526	3.026522	0.4556516	12.3194	-0.7314684	13.50652
BE vs AL	4.581479	3.48615	-2.251249	11.41421	-3.368451	12.53141
BF vs AL	4.05663	3.545925	-2.893255	11.00651	-4.003125	12.11639
BL vs AL	1.483099	2.442029	-3.303189	6.269387	-4.625087	7.591285
BM vs AL	-6.71622	2.700624	-12.00935	-1.423094	-13.26395	-0.1684938
D vs AL	-1.207803	2.93655	-6.963336	4.547729	-8.167392	5.751785
DL vs AL	-0.0434961	2.137843	-4.233592	4.1466	-5.654425	5.567433
EK vs AL	-4.454709	2.171456	-8.710684	-0.1987338	-10.11937	1.209954
F vs AL	-1.64942	1.333668	-4.26336	0.9645206	-6.099692	2.800852
FG vs AL	-1.81878	1.652404	-5.057432	1.419871	-6.695883	3.058323
FJ vs AL	-0.9770188	1.932249	-4.764157	2.81012	-6.266846	4.312809
G vs AL	-0.2713518	1.03141	-2.292878	1.750175	-4.371922	3.829219
H vs AL	0.5629811	1.920936	-3.201985	4.327947	-4.709583	5.835546
K vs AL	-1.456008	1.25145	-3.908805	0.9967884	-5.805245	2.893229
KL vs AL	-3.014853	2.126154	-7.182038	1.152333	-8.607172	2.577466
L vs AL	2.880241	1.748221	-0.5462089	6.306692	-2.134647	7.89513
M vs AL	-3.851974	2.369193	-8.495506	0.7915584	-9.838968	2.13502
B vs AM	7.374544	3.593556	0.331304	14.41778	-0.7729784	15.52207
BE vs AM	5.568497	3.988151	-2.248135	13.38513	-3.313863	14.45086
BF vs AM	5.043649	4.040683	-2.875945	12.96324	-3.937474	14.02477
BL vs AM	2.470118	3.117997	-3.641044	8.581279	-4.812134	9.752369
BM vs AM	-5.729201	3.148912	-11.90096	0.4425524	-13.06688	1.608482
D vs AM	-0.2207849	3.353452	-6.793429	6.35186	-7.928244	7.486674
DL vs AM	0.9435225	2.887196	-4.715278	6.602323	-5.929148	7.816193
EK vs AM	-3.467691	2.708772	-8.776787	1.841405	-10.02948	3.0941
F vs AM	-0.6624012	2.097862	-4.774135	3.449332	-6.209845	4.885043
FG vs AM	-0.8317615	2.313651	-5.366435	3.702912	-6.727196	5.063673
FJ vs AM	0.0099998	2.521116	-4.931296	4.951296	-6.231141	6.251141

Table A8. Prediction interval for PSQI (continued).

Comparison	Effect Size	Standard Error	LCI	UCI	LPrI	UPrI
G vs AM	0.7156668	1.919979	-3.047424	4.478757	-4.555439	5.986773
H vs AM	1.55	2.512456	-3.374323	6.474323	-4.676516	7.776515
K vs AM	-0.4689897	2.046547	-4.480148	3.542169	-5.935666	4.997687
KL vs AM	-2.027834	2.672588	-7.26601	3.210342	-8.527264	4.471595
L vs AM	3.86726	2.612025	-1.252215	8.986735	-2.52835	10.26287
M vs AM	-2.864955	2.869738	-8.489538	2.759627	-9.706971	3.97706
BE vs B	-1.806047	1.738117	-5.212695	1.600601	-6.806224	3.194129
BF vs B	-2.330895	1.848826	-5.954527	1.292737	-7.494503	2.832712
BL vs B	-4.904427	1.801281	-8.434873	-1.37398	-9.997256	0.1884035
BM vs B	-13.10375	4.052815	-21.04712	-5.160374	-22.10771	-4.099786
D vs B	-7.595329	4.21368	-15.85399	0.663332	-16.90306	1.712397
DL vs B	-6.431022	2.773002	-11.866	-0.9960382	-13.10409	0.2420493
EK vs B	-10.84223	3.721206	-18.13566	-3.548805	-19.22602	-2.458447
F vs B	-8.036945	3.303069	-14.51084	-1.563049	-15.65285	-0.4210367
FG vs B	-8.206306	3.44401	-14.95644	-1.456171	-16.07903	-0.3335809
FJ vs B	-7.364544	3.586756	-14.39446	-0.3346321	-15.49952	0.7704347
G vs B	-6.658877	3.192915	-12.91688	-0.400879	-14.07568	0.7579213
H vs B	-5.824544	3.580674	-12.84254	1.193448	-13.94831	2.29922
K vs B	-7.843534	3.270821	-14.25423	-1.432841	-15.401	-0.2860667
KL vs B	-9.402378	3.694973	-16.64439	-2.160365	-17.73749	-1.067267
L vs B	-3.507284	2.484118	-8.376066	1.361497	-9.686056	2.671488
M vs B	-10.2395	3.839898	-17.76556	-2.713437	-18.84426	-1.634741
BF vs BE	-0.5248481	2.537558	-5.498371	4.448675	-6.7938	5.744104
BL vs BE	-3.098379	2.501187	-8.000615	1.803857	-9.305888	3.109129
BM vs BE	-11.2977	4.406483	-19.93425	-2.66115	-20.97167	-1.623721
D vs BE	-5.789282	4.554873	-14.71667	3.138105	-15.7466	4.168038
DL vs BE	-4.624975	3.269868	-11.0338	1.783849	-12.18072	2.930766
EK vs BE	-9.036187	4.103558	-17.07901	-0.9933614	-18.13578	0.0634025
F vs BE	-6.230898	3.72854	-13.5387	1.076907	-14.6283	2.166508
FG vs BE	-6.400259	3.853939	-13.95384	1.153324	-15.03123	2.230717
FJ vs BE	-5.558497	3.982025	-13.36312	2.246128	-14.42935	3.312358
G vs BE	-4.85283	3.631304	-11.97006	2.264395	-13.07006	3.364403
H vs BE	-4.018497	3.976547	-11.81239	3.775393	-12.87907	4.842075
K vs BE	-6.037487	3.700011	-13.28938	1.214402	-14.38194	2.306969
KL vs BE	-7.596331	4.079785	-15.59256	0.3999015	-16.6511	1.458435
L vs BE	-1.701237	3.028755	-7.637488	4.235014	-8.824203	5.421729
M vs BE	-8.433453	4.211482	-16.68781	-0.1790993	-17.73702	0.8701112
BL vs BF	-2.573531	2.580934	-7.632069	2.485006	-8.916121	3.769059
BM vs BF	-10.77285	4.454076	-19.50268	-2.043021	-20.53757	-1.008129
D vs BF	-5.264434	4.600933	-14.2821	3.753229	-15.30994	4.781075
DL vs BF	-4.100126	3.332377	-10.63146	2.431213	-11.76925	3.569002
EK vs BF	-8.511339	4.15462	-16.65424	-0.3684343	-17.70734	0.6846594
F vs BF	-5.70605	3.784673	-13.12387	1.711772	-14.20785	2.795746
FG vs BF	-5.87541	3.908282	-13.5355	1.784682	-14.608	2.857185

Table A8. Prediction interval for PSQI (continued).

Comparison	Effect Size	Standard Error	LCI	UCI	LPrI	UPrI
FJ vs BF	-5.033649	4.034637	-12.94139	2.874094	-14.00339	3.936096
G vs BF	-4.327982	3.688926	-11.55814	2.902181	-12.65188	3.995919
H vs BF	-3.493649	4.029231	-11.3908	4.403499	-12.45322	5.465927
K vs BF	-5.512639	3.756562	-12.87537	1.850089	-13.96212	2.936847
KL vs BF	-7.071483	4.131139	-15.16837	1.025401	-16.22313	2.080161
L vs BF	-1.176389	3.096133	-7.244699	4.891921	-8.419516	6.066738
M vs BF	-7.908604	4.261256	-16.26051	0.4433041	-17.30649	1.489286
BM vs BL	-8.199319	3.63777	-15.32922	-1.069421	-16.4285	0.029868
D vs BL	-2.690902	3.816177	-10.17047	4.788666	-11.2514	5.869598
DL vs BL	-1.526595	2.113929	-5.66982	2.61663	-7.099495	4.046305
EK vs BL	-5.937808	3.264257	-12.33563	0.4600178	-13.48339	1.607777
F vs BL	-3.132519	2.778205	-8.577701	2.312663	-9.814636	3.549599
FG vs BL	-3.301879	2.944417	-9.072831	2.469074	-10.27536	3.671601
FJ vs BL	-2.460118	3.110157	-8.555914	3.635679	-9.728333	4.808097
G vs BL	-1.754451	2.64633	-6.941162	3.43226	-8.20878	4.699879
H vs BL	-0.9201178	3.103142	-7.002163	5.161928	-8.175778	6.335543
K vs BL	-2.939107	2.739758	-8.308934	2.430719	-9.554489	3.676275
KL vs BL	-4.497952	3.234313	-10.83709	1.841185	-11.98941	2.993502
L vs BL	1.397142	1.717543	-1.969181	4.763465	-3.573207	6.367491
M vs BL	-5.335073	3.398961	-11.99691	1.326767	-13.12547	2.455326
D vs BM	5.508416	2.169282	1.256702	9.760131	-0.152762	11.16959
DL vs BM	6.672724	3.441988	-0.0734485	13.4189	-1.196302	14.54175
EK vs BM	2.261511	3.194585	-3.999762	8.522783	-5.158296	9.681317
F vs BM	5.0668	2.351141	0.4586484	9.674952	-0.8903527	11.02395
FG vs BM	4.89744	2.977369	-0.9380951	10.73298	-2.134319	11.9292
FJ vs BM	5.739201	3.141149	-0.4173386	11.89574	-1.584552	13.06295
G vs BM	6.444868	2.682869	1.186541	11.70319	-0.0722552	12.96199
H vs BM	7.279201	3.134203	1.136276	13.42213	-0.0320938	14.5905
K vs BM	5.260211	2.656335	0.0538907	10.46653	-1.211287	11.73171
KL vs BM	3.701367	3.164463	-2.500865	9.9036	-3.664248	11.06698
L vs BM	9.596461	3.214638	3.295887	15.89704	2.140515	17.05241
M vs BM	2.864246	1.298143	0.3199325	5.408559	-1.541868	7.27036
DL vs D	1.164307	3.630033	-5.950426	8.27904	-7.050576	9.37919
EK vs D	-3.246906	3.396489	-9.903902	3.410091	-11.03279	4.538983
F vs D	-0.4416163	2.61902	-5.574802	4.691569	-6.849181	5.965948
FG vs D	-0.6109766	3.192882	-6.86891	5.646957	-8.027716	6.805763
FJ vs D	0.2307846	3.346164	-6.327576	6.789145	-7.463413	7.924983
G vs D	0.9364517	2.920209	-4.787053	6.659956	-5.994317	7.86722
H vs D	1.770785	3.339644	-4.774797	8.316367	-5.911555	9.453124
K vs D	-0.2482048	2.89601	-5.92428	5.427871	-7.136369	6.639959
KL vs D	-1.807049	3.368182	-8.408565	4.794466	-9.541328	5.92723
L vs D	4.088045	3.415229	-2.605682	10.78177	-3.73206	11.90815
M vs D	-2.644171	1.737996	-6.050581	0.7622393	-7.644171	2.35583
EK vs DL	-4.411213	3.044546	-10.37841	1.555987	-11.56228	2.739856

Table A8. Prediction interval for PSQI (continued).

Comparison	Effect Size	Standard Error	LCI	UCI	LPrI	UPrI
F vs DL	-1.605924	2.516421	-6.538019	3.326172	-7.839134	4.627287
FG vs DL	-1.775284	2.698824	-7.064881	3.514313	-8.319904	4.769336
FJ vs DL	-0.9335227	2.878728	-6.575727	4.708681	-7.791318	5.924273
G vs DL	-0.2278556	2.370042	-4.873053	4.417341	-6.216256	5.760545
H vs DL	0.6064773	2.871147	-5.020868	6.233822	-6.238011	7.450966
K vs DL	-1.412512	2.473896	-6.261259	3.436235	-7.574105	4.749081
KL vs DL	-2.971357	3.012416	-8.875584	2.93287	-10.06528	4.122567
L vs DL	2.923738	1.232399	0.5082794	5.339196	-1.402685	7.25016
M vs DL	-3.808478	3.188553	-10.05793	2.440971	-11.21742	3.600467
F vs EK	2.80529	2.165178	-1.438382	7.048961	-2.849313	8.459892
FG vs EK	2.635929	2.507352	-2.27839	7.550248	-3.581974	8.853832
FJ vs EK	3.47769	2.699745	-1.813712	8.769093	-3.068519	10.0239
G vs EK	4.183357	2.14937	-0.0293306	8.396045	-1.445962	9.812676
H vs EK	5.01769	2.691659	-0.2578655	10.29325	-1.514577	11.54996
K vs EK	2.998701	1.774723	-0.4796917	6.477093	-2.054976	8.052378
KL vs EK	1.439857	2.472807	-3.406755	6.286469	-4.719907	7.59962
L vs EK	7.33495	2.784929	1.87659	12.79331	0.6411352	14.02877
M vs EK	0.6027352	2.919813	-5.119992	6.325463	-6.327334	7.532804
FG vs F	-0.1693603	1.830203	-3.756493	3.417772	-5.305142	4.966422
FJ vs F	0.672401	2.086192	-3.41646	4.761262	-4.856604	6.201406
G vs F	1.378068	1.297243	-1.164482	3.920618	-3.026937	5.783073
H vs F	2.212401	2.075719	-1.855933	6.280735	-3.300091	7.724893
K vs F	0.1934115	1.240454	-2.237834	2.624657	-4.14263	4.529452
KL vs F	-1.365433	2.120518	-5.521572	2.790706	-6.948793	4.217927
L vs F	4.529661	2.195238	0.2270742	8.832248	-1.173216	10.23254
M vs F	-2.202554	1.961271	-6.046574	1.641466	-7.53687	3.131762
FJ vs FG	0.8417612	2.303076	-3.672184	5.355706	-5.036326	6.719849
G vs FG	1.547428	1.291028	-0.9829395	4.077796	-2.849932	5.944788
H vs FG	2.381761	2.293592	-2.113598	6.87712	-3.480796	8.244318
K vs FG	0.3627718	1.771358	-3.109026	3.83457	-4.685965	5.411508
KL vs FG	-1.196073	2.468253	-6.03376	3.641614	-7.348192	4.956047
L vs FG	4.699021	2.402192	-0.0091885	9.407231	-1.342727	10.74077
M vs FG	-2.033194	2.680338	-7.286561	3.220172	-8.545959	4.479572
G vs FJ	0.7056671	1.907222	-3.032419	4.443753	-4.54603	5.957365
H vs FJ	1.54	2.50272	-3.365242	6.445241	-4.670094	7.750094
K vs FJ	-0.4789895	2.034583	-4.4667	3.508721	-5.926954	4.968975
KL vs FJ	-2.037834	2.663438	-7.258076	3.182408	-8.521532	4.445865
L vs FJ	3.85726	2.602662	-1.243864	8.958385	-2.522362	10.23688
M vs FJ	-2.874955	2.861218	-8.48284	2.732929	-9.702029	3.952119
H vs G	0.8343329	1.89576	-2.881288	4.549953	-4.399976	6.068642
K vs G	-1.184657	1.212721	-3.561546	1.192233	-5.487756	3.118443
KL vs G	-2.743501	2.103616	-6.866512	1.37951	-8.300053	2.813051
L vs G	3.151593	2.02582	-0.8189411	7.122127	-2.282694	8.585879
M vs G	-3.580622	2.348904	-8.18439	1.023146	-9.534083	2.372838

Table A8. Prediction interval for PSQI (continued).

Comparison	Effect Size	Standard Error	LCI	UCI	LPrI	UPrI
K vs H	-2.018989	2.023843	-5.985648	1.947669	-7.450193	3.412215
KL vs H	-3.577834	2.655242	-8.782013	1.626345	-10.04746	2.891788
L vs H	2.31726	2.594275	-2.767424	7.401945	-4.048056	8.682576
M vs H	-4.414955	2.85359	-10.00789	1.177979	-11.22866	2.398752
KL vs K	-1.558844	1.721958	-4.93382	1.816131	-6.535578	3.41789
L vs K	4.336249	2.14633	0.1295203	8.542978	-1.288216	9.960714
M vs K	-2.395966	2.318629	-6.940395	2.148463	-8.299575	3.507643
L vs KL	5.895094	2.749762	0.5056601	11.28453	-0.7376277	12.52782
M vs KL	-0.8371214	2.886837	-6.495219	4.820976	-7.709161	6.034918
M vs L	-6.732215	2.941688	-12.49782	-0.9666126	-13.70088	0.2364441

Table A9. Prediction interval for change in PSQI scores.

Comparison	Effect Size	Standard Error	LCI	UCI	LPrI	UPrI
AB vs A	3.389904	0.7234206	1.972026	4.807783	-1.059394	7.839203
ABGN vs A	4.72011	2.37555	0.0641173	9.376103	-1.690278	11.1305
ACD vs A	1.35	2.098831	-2.763634	5.463634	-4.645237	7.345237
AE vs A	2.411915	1.225962	0.009073	4.814757	-2.473902	7.297732
AF vs A	3.566143	1.24162	1.132613	5.999673	-1.336093	8.46838
AG vs A	2.023825	1.219187	-0.3657373	4.413387	-2.854935	6.902585
AH vs A	2.284461	1.49396	-0.6436466	5.212569	-2.902377	7.471299
AK vs A	3.559497	1.651378	0.3228559	6.796138	-1.822217	8.941211
AL vs A	1.861225	0.904023	0.0893727	3.633078	-2.723407	6.445858
AM vs A	2.45	2.155591	-1.774881	6.67488	-3.628431	8.528431
B vs A	-4.434173	3.773614	-11.83032	2.961974	-13.20076	4.332409
BE vs A	-2.429227	4.305029	-10.86693	6.008476	-12.16117	7.302714
BF vs A	-2.193073	4.349224	-10.7174	6.331249	-12.00641	7.620265
BL vs A	0.3919972	3.088903	-5.662142	6.446136	-7.178182	7.962176
BM vs A	8.483032	3.031964	2.540491	14.42557	1.009217	15.95685
D vs A	2.999151	3.332698	-3.532817	9.531119	-4.989573	10.98787
DL vs A	-2.156729	2.727076	-7.501699	3.188242	-9.124813	4.811356
EK vs A	5.740153	2.424104	0.988997	10.49131	-0.7454029	12.22571
F vs A	3.0339	1.353247	0.381585	5.686214	-1.989723	8.057522
FG vs A	3.629612	1.701153	0.2954136	6.96381	-1.81621	9.075434
FJ vs A	2.51	2.102021	-1.609885	6.629886	-3.489884	8.509884
G vs A	1.96906	0.8173891	0.3670067	3.571113	-2.547412	6.485532
H vs A	0.8099999	2.092762	-3.291739	4.911739	-5.176406	6.796406
K vs A	2.300928	1.11327	0.1189597	4.482897	-2.471354	7.073211
KL vs A	4.62931	2.407775	-0.0898427	9.348463	-1.830895	11.08952
L vs A	-0.8811484	2.292955	-5.375257	3.61296	-7.165187	5.40289
M vs A	5.616122	2.614623	0.4915541	10.74069	-1.170303	12.40255
ABGN vs AB	1.330206	2.48327	-3.536914	6.197326	-5.247802	7.908213
ACD vs AB	-2.039905	2.220007	-6.391038	2.311229	-8.214024	4.134215
AE vs AB	-0.9779896	1.423515	-3.768028	1.812048	-6.081753	4.125773
AF vs AB	0.1762387	1.437009	-2.640246	2.992724	-4.94323	5.295707
AG vs AB	-1.366079	1.417674	-4.144669	1.41251	-6.463075	3.730917
AH vs AB	-1.105443	1.659895	-4.358777	2.14789	-6.498045	4.287158
AK vs AB	0.1695925	1.802914	-3.364054	3.70324	-5.410748	5.749933
AL vs AB	-1.528679	1.157715	-3.797759	0.7404009	-6.344749	3.28739
AM vs AB	-0.9399047	2.273743	-5.39636	3.516551	-7.194839	5.31503
B vs AB	-7.824078	3.842257	-15.35476	-0.2933933	-16.71382	1.065664
BE vs AB	-5.819131	4.36532	-14.375	2.736738	-15.66215	4.02389
BF vs AB	-5.582978	4.408913	-14.22429	3.058333	-15.50649	4.340536
BL vs AB	-2.997907	3.172409	-9.215714	3.219899	-10.7104	4.714581
BM vs AB	5.093128	3.117233	-1.016536	11.20279	-2.525201	12.71146

Table A9. Prediction interval for change in PSQI scores (continued).

Comparison	Effect Size	Standard Error	LCI	UCI	LPrI	UPrI
D vs AB	-0.3907532	3.410447	-7.075107	6.2936	-8.514821	7.733315
DL vs AB	-5.546633	2.821388	-11.07645	-0.016815	-12.66919	1.575927
EK vs AB	2.350248	2.529757	-2.607984	7.30848	-4.301033	9.001529
F vs AB	-0.3560049	1.534715	-3.363991	2.651982	-5.592103	4.880094
FG vs AB	0.2397075	1.848515	-3.383315	3.862731	-5.40235	5.881765
FJ vs AB	-0.8799045	2.223023	-5.236949	3.47714	-7.058536	5.298727
G vs AB	-1.420845	1.091481	-3.560109	0.7184196	-6.172145	3.330456
H vs AB	-2.579905	2.21427	-6.919794	1.759985	-8.745449	3.58564
K vs AB	-1.088976	1.327688	-3.691197	1.513244	-6.08415	3.906198
KL vs AB	1.239406	2.514116	-3.688171	6.166982	-5.387161	7.865972
L vs AB	-4.271053	2.404283	-8.983361	0.441256	-10.72585	2.183741
M vs AB	2.226217	2.713021	-3.091207	7.543641	-4.719008	9.171443
ACD vs ABGN	-3.37011	3.16991	-9.58302	2.8428	-11.07832	4.338104
AE vs ABGN	-2.308195	2.67324	-7.547649	2.931258	-9.188955	4.572565
AF vs ABGN	-1.153967	2.680458	-6.407568	4.099634	-8.046398	5.738464
AG vs ABGN	-2.696285	2.67014	-7.929664	2.537093	-9.572038	4.179467
AH vs ABGN	-2.435649	2.806271	-7.935839	3.064541	-9.533324	4.662026
AK vs ABGN	-1.160613	2.668631	-6.391034	4.069808	-8.033928	5.712702
AL vs ABGN	-2.858885	2.54176	-7.840643	2.122873	-9.529174	3.811404
AM vs ABGN	-2.27011	3.207773	-8.557231	4.01701	-10.04321	5.502986
B vs ABGN	-9.154284	4.459091	-17.89394	-0.4146256	-19.17062	0.8620559
BE vs ABGN	-7.149337	4.916972	-16.78642	2.487751	-18.02062	3.721945
BF vs ABGN	-6.913184	4.955712	-16.6262	2.799833	-17.85739	4.031024
BL vs ABGN	-4.328113	3.896748	-11.9656	3.309372	-13.31597	4.659748
BM vs ABGN	3.762922	3.78511	-3.655756	11.1816	-5.024251	12.55009
D vs ABGN	-1.720959	4.030109	-9.619827	6.17791	-10.9502	7.508284
DL vs ABGN	-6.876839	3.616655	-13.96535	0.2116741	-15.36377	1.610089
EK vs ABGN	1.020043	3.006963	-4.873497	6.913582	-6.411639	8.451724
F vs ABGN	-1.686211	2.638671	-6.857912	3.48549	-8.511242	5.138821
FG vs ABGN	-1.090498	2.921849	-6.817217	4.63622	-8.379589	6.198593
FJ vs ABGN	-2.21011	3.172023	-8.427161	4.006941	-9.921939	5.501719
G vs ABGN	-2.75105	2.512247	-7.674964	2.172863	-9.374668	3.872567
H vs ABGN	-3.91011	3.165895	-10.11515	2.294931	-11.61146	3.791238
K vs ABGN	-2.419182	2.098677	-6.532514	1.69415	-8.414195	3.575831
KL vs ABGN	-0.0908001	2.99393	-5.958794	5.777194	-7.500561	7.318961
L vs ABGN	-5.601259	3.301658	-12.07239	0.869872	-13.53619	2.333671
M vs ABGN	0.8960114	3.459818	-5.885108	7.677131	-7.314433	9.106456
AE vs ACD	1.061915	2.430654	-3.702079	5.825908	-5.43383	7.55766
AF vs ACD	2.216143	2.438588	-2.563402	6.995688	-4.29196	8.724247
AG vs ACD	0.6738251	2.427243	-4.083484	5.431134	-5.816613	7.164263
AH vs ACD	0.9344611	2.576239	-4.114875	5.983798	-5.790625	7.659547

Table A9. Prediction interval for change in PSQI scores (continued).

Comparison	Effect Size	Standard Error	LCI	UCI	LPrI	UPrI
AK vs ACD	2.209497	2.670607	-3.024796	7.44379	-4.667009	9.086003
AL vs ACD	0.5112254	2.285246	-3.967775	4.990226	-5.761122	6.783573
AM vs ACD	1.1	3.008598	-4.796745	6.996745	-6.334435	8.534434
B vs ACD	-5.784173	4.318015	-14.24733	2.678982	-15.54002	3.97167
BE vs ACD	-3.779227	4.789402	-13.16628	5.607828	-14.41098	6.852526
BF vs ACD	-3.543073	4.829165	-13.00806	5.921917	-14.24938	7.163238
BL vs ACD	-0.9580027	3.73449	-8.27747	6.361464	-9.654617	7.738612
BM vs ACD	7.133032	3.687533	-0.0944	14.36047	-1.479831	15.7459
D vs ACD	1.649151	3.938524	-6.070213	9.368516	-7.414137	10.71244
DL vs ACD	-3.506728	3.441226	-10.25141	3.237951	-11.68461	4.67115
EK vs ACD	4.390153	3.206458	-1.894389	10.6747	-3.380685	12.16099
F vs ACD	1.6839	2.497272	-3.210664	6.578464	-4.916121	8.28392
FG vs ACD	2.279612	2.701669	-3.015562	7.574786	-4.647182	9.206406
FJ vs ACD	1.16	2.970452	-4.661979	6.981979	-6.210352	8.530353
G vs ACD	0.6190599	2.25238	-3.795525	5.033645	-5.603641	6.841761
H vs ACD	-0.54	2.963907	-6.349152	5.269152	-7.899385	6.819385
K vs ACD	0.9509284	2.375808	-3.705569	5.607426	-5.459857	7.361714
KL vs ACD	3.27931	3.194131	-2.981072	9.539693	-4.470383	11.029
L vs ACD	-2.231148	3.108494	-8.323685	3.861388	-9.83461	5.372313
M vs ACD	4.266122	3.352812	-2.305269	10.83751	-3.757534	12.28978
AF vs AE	1.154228	1.744877	-2.265667	4.574124	-4.348836	6.657293
AG vs AE	-0.3880899	1.728985	-3.776839	3.000659	-5.870251	5.094071
AH vs AE	-0.1274538	1.932589	-3.915259	3.660351	-5.885502	5.630595
AK vs AE	1.147582	2.056698	-2.883472	5.178636	-4.786605	7.081769
AL vs AE	-0.5506896	1.523255	-3.536214	2.434835	-5.77285	4.67147
AM vs AE	0.038085	2.47983	-4.822292	4.898462	-6.534522	6.610692
B vs AE	-6.846088	3.967779	-14.62279	0.9306164	-15.9623	2.270124
BE vs AE	-4.841142	4.476203	-13.61434	3.932055	-14.88918	5.206896
BF vs AE	-4.604988	4.518723	-13.46152	4.251547	-14.73188	5.521905
BL vs AE	-2.019918	3.323314	-8.533493	4.493658	-9.992364	5.952528
BM vs AE	6.071117	3.270408	-0.3387652	12.481	-1.809798	13.95203
D vs AE	0.5872365	3.551007	-6.37261	7.547082	-7.783587	8.958059
DL vs AE	-4.568643	2.989973	-10.42888	1.291596	-11.97176	2.83447
EK vs AE	3.328238	2.716477	-1.99596	8.652436	-3.622605	10.27908
F vs AE	0.6219847	1.825951	-2.956813	4.200782	-4.989426	6.233396
FG vs AE	1.217697	2.096893	-2.892138	5.327532	-4.774718	7.210112
FJ vs AE	0.0980851	2.433408	-4.671307	4.867477	-6.401948	6.598119
G vs AE	-0.442855	1.473478	-3.330819	2.445109	-5.605265	4.719554
H vs AE	-1.601915	2.425415	-6.355641	3.151811	-8.08951	4.88568
K vs AE	-0.1109865	1.656002	-3.356691	3.134717	-5.498608	5.276635
KL vs AE	2.217395	2.701916	-3.078264	7.513054	-4.7098	9.14459

Table A9. Prediction interval for change in PSQI scores (continued).

Comparison	Effect Size	Standard Error	LCI	UCI	LPrI	UPrI
L vs AE	-3.293063	2.600137	-8.389238	1.803112	-10.0563	3.470172
M vs AE	3.204207	2.88774	-2.455659	8.864073	-4.028122	10.43653
AG vs AF	-1.542318	1.740124	-4.952899	1.868262	-7.039119	3.954483
AH vs AF	-1.281682	1.942559	-5.089028	2.525664	-7.053669	4.490305
AK vs AF	-0.0066462	2.066073	-4.056074	4.042782	-5.954364	5.941072
AL vs AF	-1.704918	1.535874	-4.715176	1.30534	-6.94243	3.532594
AM vs AF	-1.116143	2.487607	-5.991765	3.759478	-7.700964	5.468678
B vs AF	-8.000317	3.972637	-15.78654	-0.2140911	-17.12532	1.124692
BE vs AF	-5.99537	4.480509	-14.77701	2.786267	-16.05139	4.060648
BF vs AF	-5.759216	4.522989	-14.62411	3.10568	-15.89403	4.375596
BL vs AF	-3.174146	3.329113	-9.699089	3.350797	-11.15665	4.808359
BM vs AF	4.916889	3.276327	-1.504594	11.33837	-2.974246	12.80802
D vs AF	-0.5669919	3.556458	-7.537521	6.403538	-8.947434	7.813451
DL vs AF	-5.722872	2.996426	-11.59576	0.1500153	-13.13683	1.691086
EK vs AF	2.17401	2.723581	-3.164111	7.51213	-4.788386	9.136405
F vs AF	-0.5322436	1.836523	-4.131762	3.067275	-6.157988	5.0935
FG vs AF	0.0634688	2.106079	-4.06437	4.191308	-5.942331	6.069269
FJ vs AF	-1.056143	2.441334	-5.84107	3.728784	-7.568527	5.456241
G vs AF	-1.597083	1.486526	-4.510621	1.316454	-6.775029	3.580863
H vs AF	-2.756143	2.433367	-7.525455	2.013168	-9.256112	3.743826
K vs AF	-1.265215	1.667628	-4.533706	2.003277	-6.667733	4.137303
KL vs AF	1.063167	2.709058	-4.246489	6.372823	-5.87562	8.001954
L vs AF	-4.447291	2.607548	-9.557991	0.6634082	-11.22238	2.3278
M vs AF	2.049978	2.894439	-3.623018	7.722975	-5.193481	9.293438
AH vs AG	0.2606361	1.928298	-3.518759	4.040031	-5.491426	6.012698
AK vs AG	1.535672	2.052669	-2.487485	5.558829	-4.392709	7.464053
AL vs AG	-0.1625997	1.517799	-3.137431	2.812232	-5.378148	5.052948
AM vs AG	0.4261748	2.476487	-4.42765	5.28	-6.141187	6.993537
B vs AG	-6.457998	3.965685	-14.2306	1.314601	-15.57042	2.654423
BE vs AG	-4.453052	4.474346	-13.22261	4.316506	-14.49765	5.591545
BF vs AG	-4.216898	4.516884	-13.06983	4.636032	-14.34038	5.906582
BL vs AG	-1.631828	3.320814	-8.140504	4.876849	-9.59994	6.336284
BM vs AG	6.459208	3.267888	0.0542655	12.86415	-1.417358	14.33577
D vs AG	0.9753264	3.548685	-5.979968	7.930621	-7.391399	9.342052
DL vs AG	-4.180553	2.9872	-10.03536	1.674252	-11.57901	3.217902
EK vs AG	3.716328	2.713427	-1.601892	9.034548	-3.229558	10.66221
F vs AG	1.010075	1.821426	-2.559855	4.580004	-4.595216	6.615365
FG vs AG	1.605787	2.092934	-2.496288	5.707862	-4.380868	7.592442
FJ vs AG	0.486175	2.430002	-4.276541	5.248891	-6.008555	6.980906
G vs AG	-0.0547652	1.467842	-2.931682	2.822152	-5.210492	5.100961
H vs AG	-1.213825	2.421997	-5.960853	3.533203	-7.696106	5.268456

Table A9. Prediction interval for change in PSQI scores (continued).

Comparison	Effect Size	Standard Error	LCI	UCI	LPrI	UPrI
K vs AG	0.2771034	1.650993	-2.958784	3.512991	-5.10412	5.658327
KL vs AG	2.605485	2.69885	-2.684163	7.895134	-4.316736	9.527706
L vs AG	-2.904973	2.596943	-7.994888	2.184942	-9.663101	3.853154
M vs AG	3.592297	2.884883	-2.06197	9.246563	-3.635288	10.81988
AK vs AH	1.275036	2.226874	-3.089556	5.639628	-4.909361	7.459433
AL vs AH	-0.4232357	1.746187	-3.8457	2.999229	-5.92803	5.081558
AM vs AH	0.1655388	2.622687	-4.974833	5.305911	-6.633817	6.964894
B vs AH	-6.718634	4.05858	-14.67331	1.236037	-15.99963	2.562361
BE vs AH	-4.713688	4.556884	-13.64502	4.21764	-14.91146	5.484085
BF vs AH	-4.477534	4.598659	-13.49074	4.535671	-14.75301	5.797946
BL vs AH	-1.892464	3.431215	-8.617521	4.832594	-10.05283	6.267898
BM vs AH	6.198571	3.380049	-0.4262042	12.82335	-1.872481	14.26962
D vs AH	0.7146903	3.652232	-6.443554	7.872934	-7.835378	9.264758
DL vs AH	-4.441189	3.109479	-10.53566	1.653277	-12.04633	3.163948
EK vs AH	3.455692	2.847489	-2.125285	9.036668	-3.709945	10.62133
F vs AH	0.7494385	2.015738	-3.201336	4.700213	-5.125996	6.624873
FG vs AH	1.345151	2.264031	-3.092268	5.782569	-4.895112	7.585413
FJ vs AH	0.225539	2.578839	-4.828892	5.27997	-6.503689	6.954767
G vs AH	-0.3154012	1.70295	-3.653123	3.02232	-5.763559	5.132757
H vs AH	-1.474461	2.571298	-6.514112	3.56519	-8.191675	5.242753
K vs AH	0.0164673	1.863139	-3.635218	3.668152	-5.645563	5.678498
KL vs AH	2.344849	2.833602	-3.208908	7.898606	-4.797851	9.48755
L vs AH	-3.165609	2.736705	-8.529452	2.198234	-10.14938	3.81816
M vs AH	3.331661	3.011342	-2.570462	9.233783	-4.107393	10.77071
AL vs AK	-1.698272	1.882659	-5.388216	1.991673	-7.387098	3.990554
AM vs AK	-1.109497	2.715441	-6.431664	4.212669	-8.058655	5.839661
B vs AK	-7.99367	4.119151	-16.06706	0.0797172	-17.38501	1.397669
BE vs AK	-5.988724	4.610914	-15.02595	3.048502	-16.28703	4.309577
BF vs AK	-5.75257	4.652203	-14.87072	3.36558	-16.12782	4.622684
BL vs AK	-3.1675	3.502647	-10.03256	3.697563	-11.45314	5.118137
BM vs AK	4.923535	3.415123	-1.769984	11.61705	-3.2087	13.05577
D vs AK	-0.5603457	3.684763	-7.78235	6.661658	-9.168277	8.047585
DL vs AK	-5.716226	3.188104	-11.9648	0.5323443	-13.45559	2.023138
EK vs AK	2.180656	2.711933	-3.134635	7.495946	-4.762801	9.124113
F vs AK	-0.5255975	2.073674	-4.589924	3.538729	-6.484309	5.433114
FG vs AK	0.070115	2.370877	-4.576718	4.716948	-6.333072	6.473302
FJ vs AK	-1.049497	2.673115	-6.288705	4.189711	-7.930055	5.831061
G vs AK	-1.590437	1.842612	-5.20189	2.021016	-7.224457	4.043582
H vs AK	-2.749497	2.66584	-7.974447	2.475454	-9.618305	4.119311
K vs AK	-1.258569	1.648464	-4.489499	1.972361	-6.636566	4.119429
KL vs AK	1.069813	2.697411	-4.217015	6.356641	-5.850074	7.989701

Table A9. Prediction interval for change in PSQI scores (continued).

Comparison	Effect Size	Standard Error	LCI	UCI	LPrI	UPrI
L vs AK	-4.440645	2.825744	-9.979001	1.097711	-11.57038	2.689094
M vs AK	2.056625	3.05065	-3.922539	8.035789	-5.448752	9.562001
AM vs AL	0.5887745	2.337483	-3.992609	5.170158	-5.763139	6.940688
B vs AL	-6.295399	3.667359	-13.48329	0.8924936	-14.87236	2.28156
BE vs AL	-4.290452	4.212473	-12.54675	3.965843	-13.85243	5.271527
BF vs AL	-4.054298	4.257422	-12.39869	4.290095	-13.69873	5.590134
BL vs AL	-1.469228	2.957034	-7.264908	4.326452	-8.817102	5.878646
BM vs AL	6.621807	3.164006	0.4204693	12.82314	-1.07631	14.31992
D vs AL	1.137926	3.453253	-5.630324	7.906177	-7.061012	9.336864
DL vs AL	-4.017954	2.575653	-9.06614	1.030233	-10.7421	2.706197
EK vs AL	3.878927	2.587196	-1.191883	8.949739	-2.863632	10.62149
F vs AL	1.172674	1.62763	-2.017421	4.36277	-4.178862	6.52421
FG vs AL	1.768387	1.926385	-2.007259	5.544032	-3.981008	7.517781
FJ vs AL	0.6487747	2.288176	-3.835968	5.133517	-5.628014	6.925564
G vs AL	0.1078345	1.218716	-2.280805	2.496474	-4.770436	4.986105
H vs AL	-1.051225	2.279674	-5.519304	3.416853	-7.315132	5.212682
K vs AL	0.4397031	1.434109	-2.371099	3.250506	-4.676383	5.555789
KL vs AL	2.768085	2.571904	-2.272755	7.808924	-3.950095	9.486265
L vs AL	-2.742374	2.110286	-6.878458	1.39371	-8.754313	3.269566
M vs AL	3.754896	2.766639	-1.667617	9.177409	-3.277762	10.78755
B vs AM	-6.884173	4.345887	-15.40195	1.633609	-16.69136	2.923014
BE vs AM	-4.879227	4.814546	-14.31556	4.557109	-15.55811	5.79966
BF vs AM	-4.643073	4.854104	-14.15694	4.870795	-15.39619	6.110045
BL vs AM	-2.058003	3.766682	-9.440564	5.324559	-10.81218	6.696171
BM vs AM	6.033032	3.720132	-1.258292	13.32436	-2.637946	14.70401
D vs AM	0.5491515	3.969061	-7.230066	8.328368	-8.569382	9.667686
DL vs AM	-4.606728	3.476135	-11.41983	2.206371	-12.84579	3.632334
EK vs AM	3.290153	3.243894	-3.067763	9.648068	-4.545049	11.12535
F vs AM	0.5838997	2.545161	-4.404525	5.572325	-6.091782	7.259582
FG vs AM	1.179612	2.745996	-4.202441	6.561665	-5.819311	8.178535
FJ vs AM	0.0600002	3.010824	-5.841107	5.961108	-7.378182	7.498182
G vs AM	-0.48094	2.305362	-4.999368	4.037488	-6.783834	5.821954
H vs AM	-1.64	3.004368	-7.528452	4.248453	-9.067314	5.787315
K vs AM	-0.1490715	2.426096	-4.904132	4.605989	-6.637725	6.339582
KL vs AM	2.17931	3.231711	-4.154726	8.513347	-5.634921	9.993541
L vs AM	-3.331148	3.147096	-9.499343	2.837047	-11.00038	4.338082
M vs AM	3.166122	3.388632	-3.475475	9.807718	-4.919886	11.25213
BE vs B	2.004946	2.081845	-2.075395	6.085288	-3.965605	7.975498
BF vs B	2.2411	2.164504	-2.001249	6.483449	-3.850492	8.332692
BL vs B	4.82617	2.185869	0.5419464	9.110394	-1.297073	10.94941
BM vs B	12.91721	4.840936	3.429146	22.40527	2.188807	23.64561

Table A9. Prediction interval for change in PSQI scores (continued).

Comparison	Effect Size	Standard Error	LCI	UCI	LPrI	UPrI
D vs B	7.433325	5.034742	-2.434589	17.30124	-3.659913	18.52656
DL vs B	2.277445	3.35543	-4.299077	8.853966	-5.750762	10.30565
EK vs B	10.17433	4.485146	1.383602	18.96505	0.1097145	20.23894
F vs B	7.468073	4.009077	-0.3895728	15.32572	-1.722988	16.65913
FG vs B	8.063786	4.139282	-0.0490586	16.17663	-1.3643	17.49187
FJ vs B	6.944173	4.319567	-1.522022	15.41037	-2.814525	16.70287
G vs B	6.403233	3.861096	-1.164377	13.97084	-2.520398	15.32686
H vs B	5.244173	4.315069	-3.213206	13.70155	-4.506245	14.99459
K vs B	6.735102	3.934414	-0.9762082	14.44641	-2.320759	15.79096
KL vs B	9.063483	4.476343	0.2900129	17.83695	-0.9848132	19.11178
L vs B	3.553025	3.011992	-2.350371	9.456421	-3.887123	10.99317
M vs B	10.05029	4.591071	1.051961	19.04863	-0.2110632	20.31165
BF vs BE	0.2361536	3.003189	-5.649988	6.122295	-7.189177	7.661484
BL vs BE	2.821224	3.016365	-3.090744	8.733191	-4.62629	10.26874
BM vs BE	10.91226	5.265726	0.5916257	21.23289	-0.6184238	22.44294
D vs BE	5.428378	5.444432	-5.242512	16.09927	-6.442511	17.29927
DL vs BE	0.2724985	3.945507	-7.460553	8.005549	-8.803414	9.348412
EK vs BE	8.169379	4.940612	-1.514043	17.8528	-2.746395	19.08515
F vs BE	5.463127	4.512857	-3.381911	14.30816	-4.652881	15.57913
FG vs BE	6.058839	4.628903	-3.013645	15.13132	-4.272976	16.39065
FJ vs BE	4.939227	4.790801	-4.450569	14.32902	-5.695147	15.5736
G vs BE	4.398287	4.381914	-4.190106	12.98668	-5.475357	14.27193
H vs BE	3.239227	4.786746	-6.142622	12.62108	-7.387549	13.866
K vs BE	4.730155	4.446654	-3.985127	13.44544	-5.263159	14.72347
KL vs BE	7.058537	4.932622	-2.609226	16.7263	-3.842196	17.95927
L vs BE	1.548079	3.657894	-5.621262	8.71742	-7.012051	10.10821
M vs BE	8.045348	5.036977	-1.826945	17.91764	-3.052108	19.1428
BL vs BF	2.58507	3.075701	-3.443193	8.613334	-4.962716	10.13286
BM vs BF	10.67611	5.301911	0.2845499	21.06766	-0.9233454	22.27556
D vs BF	5.192225	5.479438	-5.547276	15.93172	-6.745474	17.12992
DL vs BF	0.0363449	3.992239	-7.788299	7.860989	-9.124179	9.196868
EK vs BF	7.933226	4.979168	-1.825765	17.69222	-3.055179	18.92163
F vs BF	5.226973	4.555029	-3.700721	14.15467	-4.967354	15.4213
FG vs BF	5.822685	4.670036	-3.330418	14.97579	-4.585841	16.23121
FJ vs BF	4.703073	4.830553	-4.764637	14.17078	-6.005841	15.41199
G vs BF	4.162133	4.425342	-4.511377	12.83564	-5.791751	14.11602
H vs BF	3.003073	4.826531	-6.456754	12.4629	-7.698296	13.70444
K vs BF	4.494001	4.489454	-4.305168	13.29317	-5.578598	14.5666
KL vs BF	6.822383	4.971241	-2.921068	16.56584	-4.15108	17.79585
L vs BF	1.311925	3.708255	-5.956121	8.57997	-7.337866	9.961716
M vs BF	7.809195	5.074794	-2.137219	17.75561	-3.359699	18.97809

Table A9. Prediction interval for change in PSQI scores (continued).

Comparison	Effect Size	Standard Error	LCI	UCI	LPrI	UPrI
BM vs BL	8.091035	4.328462	-0.3925941	16.57466	-1.684045	17.86611
D vs BL	2.607154	4.544179	-6.299274	11.51358	-7.56701	12.78132
DL vs BL	-2.548726	2.552764	-7.552052	2.454601	-9.236472	4.13902
EK vs BL	5.348155	3.926535	-2.347712	13.04402	-3.69347	14.38978
F vs BL	2.641902	3.372488	-3.968053	9.251858	-5.415981	10.69979
FG vs BL	3.237615	3.526312	-3.673831	10.14906	-5.089671	11.5649
FJ vs BL	2.118003	3.736284	-5.204978	9.440985	-6.581815	10.81782
G vs BL	1.577063	3.195193	-4.6854	7.839526	-6.174451	9.328576
H vs BL	0.4180028	3.731083	-6.894785	7.730791	-8.272526	9.108532
K vs BL	1.908931	3.283407	-4.526429	8.344292	-5.994436	9.812298
KL vs BL	4.237313	3.916477	-3.438841	11.91347	-4.786148	13.26077
L vs BL	-1.273146	2.080829	-5.351496	2.805205	-7.242223	4.695932
M vs BL	5.224124	4.047083	-2.708012	13.15626	-4.035963	14.48421
D vs BM	-5.483881	2.579447	-10.5395	-0.4282589	-12.21408	1.246316
DL vs BM	-10.63976	4.077979	-18.63245	-2.647069	-19.95606	-1.323463
EK vs BM	-2.74288	3.815766	-10.22164	4.735885	-11.58503	6.099274
F vs BM	-5.449133	2.71703	-10.77441	-0.1238519	-12.40087	1.502608
FG vs BM	-4.85342	3.476705	-11.66764	1.960797	-13.09348	3.386642
FJ vs BM	-5.973032	3.68935	-13.20403	1.25796	-14.58913	2.643066
G vs BM	-6.513972	3.140275	-12.6688	-0.3591475	-14.17156	1.143618
H vs BM	-7.673032	3.684082	-14.8937	-0.4523635	-16.27975	0.933686
K vs BM	-6.182104	3.150092	-12.35617	-0.0080367	-13.85645	1.492241
KL vs BM	-3.853722	3.80543	-11.31223	3.604784	-12.67733	4.969883
L vs BM	-9.364181	3.801532	-16.81505	-1.913315	-18.18079	-0.5475678
M vs BM	-2.866911	1.536543	-5.87848	0.1446585	-8.105239	2.371417
DL vs D	-5.155879	4.306271	-13.59602	3.284257	-14.89011	4.578346
EK vs D	2.741001	4.058916	-5.214328	10.69633	-6.540604	12.02261
F vs D	0.0347483	3.049768	-5.942687	6.012183	-7.469138	7.538634
FG vs D	0.6304607	3.741858	-6.703447	7.964368	-8.079318	9.34024
FJ vs D	-0.4891513	3.940224	-8.211849	7.233546	-9.555514	8.577211
G vs D	-1.030092	3.431526	-7.755758	5.695575	-9.190997	7.130814
H vs D	-2.189151	3.935293	-9.902184	5.523881	-11.2466	6.868298
K vs D	-0.6982229	3.440611	-7.441697	6.045251	-8.875025	7.478579
KL vs D	1.630159	4.049201	-6.306129	9.566446	-7.633779	10.8941
L vs D	-3.8803	4.045447	-11.80923	4.048631	-13.13741	5.376814
M vs D	2.61697	2.071859	-1.443799	6.67774	-3.339114	8.573055
EK vs DL	7.896881	3.64873	0.745502	15.04826	-0.6469639	16.44073
F vs DL	5.190628	3.044394	-0.7762756	11.15753	-2.304177	12.68543
FG vs DL	5.78634	3.21416	-0.5132971	12.08598	-1.997722	13.5704
FJ vs DL	4.666728	3.443172	-2.081765	11.41522	-3.514556	12.84801
G vs DL	4.125788	2.846936	-1.454105	9.705681	-3.038935	11.29051

Table A9. Prediction interval for change in PSQI scores (continued).

Comparison	Effect Size	Standard Error	LCI	UCI	LPrI	UPrI
H vs DL	2.966728	3.437528	-3.770702	9.70416	-5.204678	11.13813
K vs DL	4.457657	2.94556	-1.315533	10.23085	-2.871022	11.78633
KL vs DL	6.786038	3.637902	-0.3441193	13.9162	-1.73858	15.31066
L vs DL	1.27558	1.478749	-1.622714	4.173874	-3.893095	6.444255
M vs DL	7.77285	3.778008	0.368091	15.17761	-1.001601	16.5473
F vs EK	-2.706253	2.682463	-7.963783	2.551277	-9.601928	4.189422
FG vs EK	-2.110541	2.961459	-7.914894	3.693813	-9.465824	5.244743
FJ vs EK	-3.230153	3.208546	-9.518788	3.058483	-11.00458	4.544271
G vs EK	-3.771093	2.558207	-8.785087	1.242901	-10.46748	2.925299
H vs EK	-4.930153	3.202488	-11.20691	1.346609	-12.69418	2.833874
K vs EK	-3.439224	2.153458	-7.659924	0.7814757	-9.51451	2.636061
KL vs EK	-1.110843	3.032581	-7.054593	4.832907	-8.585699	6.364014
L vs EK	-6.621301	3.336762	-13.16124	-0.081367	-14.61708	1.374477
M vs EK	-0.1240311	3.493331	-6.970834	6.722772	-8.393291	8.145229
FG vs F	0.5957124	2.17388	-3.665014	4.856439	-5.509752	6.701177
FJ vs F	-0.5238996	2.499954	-5.423718	4.375919	-7.128141	6.080342
G vs F	-1.06484	1.581041	-4.163623	2.033944	-6.357961	4.228281
H vs F	-2.2239	2.492174	-7.108471	2.660672	-8.815899	4.3681
K vs F	-0.7329712	1.599566	-3.868062	2.40212	-6.049192	4.58325
KL vs F	1.595411	2.66774	-3.633264	6.824086	-5.276466	8.467287
L vs F	-3.915048	2.662669	-9.133783	1.303687	-10.77874	2.948642
M vs F	2.582222	2.241579	-1.811193	6.975636	-3.624234	8.788678
FJ vs FG	-1.119612	2.704147	-6.419643	4.180419	-8.050427	5.811203
G vs FG	-1.660552	1.492366	-4.585536	1.264431	-6.845481	3.524377
H vs FG	-2.819612	2.696957	-8.105551	2.466326	-9.738763	4.099539
K vs FG	-1.328684	2.033059	-5.313405	2.656038	-7.228889	4.571522
KL vs FG	0.9996982	2.948109	-4.77849	6.777886	-6.333243	8.33264
L vs FG	-4.51076	2.855041	-10.10654	1.085018	-11.68889	2.667365
M vs FG	1.98651	3.119431	-4.127462	8.100482	-5.635561	9.608581
G vs FJ	-0.5409402	2.255353	-4.961351	3.87947	-6.768118	5.686238
H vs FJ	-1.7	2.966167	-7.513581	4.113581	-9.06317	5.663171
K vs FJ	-0.2090716	2.378626	-4.871093	4.45295	-6.624202	6.206059
KL vs FJ	2.11931	3.196228	-4.145182	8.383802	-5.633978	9.872599
L vs FJ	-3.391148	3.110648	-9.487907	2.705611	-10.99827	4.215978
M vs FJ	3.106122	3.35481	-3.469184	9.681428	-4.921007	11.13325
H vs G	-1.15906	2.246727	-5.562563	3.244443	-7.373253	5.055133
K vs G	0.3318686	1.381128	-2.375092	3.038829	-4.723218	5.386955
KL vs G	2.66025	2.542741	-2.32343	7.64393	-4.011593	9.332093
L vs G	-2.850208	2.434257	-7.621265	1.920848	-9.351564	3.651148
M vs G	3.647062	2.739476	-1.722213	9.016336	-3.341226	10.63535
K vs H	1.490928	2.370448	-3.155065	6.136921	-4.911599	7.893456

Table A9. Prediction interval for change in PSQI scores (continued).

Comparison	Effect Size	Standard Error	LCI	UCI	LPrI	UPrI
KL vs H	3.81931	3.190147	-2.433263	10.07188	-3.923553	11.56217
L vs H	-1.691148	3.1044	-7.77566	4.393363	-9.287649	5.905352
M vs H	4.806122	3.349016	-1.757829	11.37007	-3.210938	12.82318
KL vs K	2.328382	2.135221	-1.856574	6.513338	-3.720069	8.376833
L vs K	-3.182077	2.548935	-8.177897	1.813744	-9.863745	3.499591
M vs K	3.315193	2.750703	-2.076086	8.706472	-3.691415	10.3218
L vs KL	-5.510458	3.32492	-12.02718	1.006265	-13.48569	2.464773
M vs KL	0.9868114	3.482038	-5.837858	7.811481	-7.262613	9.236236
M vs L	6.49727	3.477782	-0.3190579	13.3136	-1.744683	14.73922

Table A10. League table for total effective rate.

AK	0.98 (0.80,1.19)	0.97 (0.80,1.17)	0.95 (0.76,1.20)	0.94 (0.73,1.20)	0.96 (0.65,1.43)	0.91 (0.75,1.11)	0.91 (0.72,1.15)	0.90 (0.71,1.13)	0.92 (0.66,1.27)	0.89 (0.68,1.18)	0.88 (0.73,1.06)	0.91 (0.60,1.38)	0.89 (0.69,1.14)	0.88 (0.71,1.09)	0.87 (0.68,1.11)	0.86 (0.66,1.13)	0.85 (0.65,1.11)	0.85 (0.64,1.13)	0.85 (0.64,1.13)	0.84 (0.62,1.14)	0.83 (0.69,1.01)	0.83 (0.68,1.01)	0.83 (0.63,1.10)	0.83 (0.66,1.04)	0.83 (0.68,1.01)	0.83 (0.69,1.00)	0.81 (0.63,1.04)	**0.81 (0.66,0.99)**	0.81 (0.65,1.01)	**0.79 (0.68,0.92)**	**0.76 (0.62,0.94)**	**0.75 (0.59,0.96)**	**0.74 (0.60,0.91)**	**0.74 (0.61,0.89)**	**0.72 (0.59,0.89)**	**0.72 (0.58,0.90)**	**0.70 (0.57,0.86)**	**0.70 (0.58,0.83)**	**0.63 (0.48,0.83)**
1.02 (0.84,1.25)	ABGN	0.99 (0.84,1.17)	0.98 (0.79,1.21)	0.96 (0.76,1.21)	0.98 (0.67,1.45)	0.94 (0.79,1.11)	0.93 (0.75,1.16)	0.92 (0.74,1.14)	0.94 (0.68,1.29)	0.91 (0.70,1.19)	0.90 (0.76,1.06)	0.93 (0.62,1.40)	0.91 (0.71,1.15)	0.90 (0.74,1.09)	0.89 (0.71,1.12)	0.88 (0.68,1.14)	0.87 (0.68,1.12)	0.87 (0.66,1.14)	0.87 (0.66,1.14)	0.86 (0.64,1.15)	0.85 (0.72,1.02)	0.85 (0.71,1.02)	0.85 (0.65,1.11)	0.85 (0.69,1.05)	0.85 (0.71,1.01)	0.85 (0.72,1.00)	0.83 (0.65,1.05)	**0.83 (0.69,0.99)**	0.83 (0.67,1.01)	**0.81 (0.71,0.92)**	**0.78 (0.64,0.94)**	**0.77 (0.61,0.97)**	**0.75 (0.62,0.91)**	**0.75 (0.63,0.90)**	**0.74 (0.62,0.89)**	**0.74 (0.60,0.91)**	**0.71 (0.59,0.86)**	**0.71 (0.61,0.83)**	**0.64 (0.49,0.84)**
1.03 (0.85,1.25)	1.01 (0.85,1.20)	KL	0.99 (0.80,1.21)	0.97 (0.77,1.22)	0.99 (0.68,1.46)	0.94 (0.80,1.11)	0.94 (0.76,1.16)	0.93 (0.75,1.14)	0.95 (0.69,1.30)	0.92 (0.71,1.20)	0.91 (0.78,1.06)	0.94 (0.63,1.41)	0.92 (0.73,1.16)	0.91 (0.76,1.09)	0.90 (0.72,1.12)	0.89 (0.69,1.14)	0.88 (0.69,1.13)	0.88 (0.67,1.15)	0.88 (0.67,1.15)	0.87 (0.65,1.16)	0.86 (0.73,1.02)	0.86 (0.73,1.02)	0.86 (0.66,1.12)	0.86 (0.70,1.05)	0.86 (0.73,1.01)	0.85 (0.73,1.00)	0.83 (0.66,1.05)	0.84 (0.70,1.00)	0.83 (0.68,1.01)	**0.82 (0.73,0.92)**	**0.78 (0.65,0.94)**	**0.78 (0.62,0.97)**	**0.76 (0.63,0.92)**	**0.76 (0.65,0.90)**	**0.75 (0.63,0.89)**	**0.74 (0.61,0.91)**	**0.72 (0.60,0.86)**	**0.72 (0.62,0.83)**	**0.65 (0.50,0.84)**
1.05 (0.83,1.32)	1.03 (0.83,1.27)	1.02 (0.82,1.25)	FG	0.98 (0.77,1.25)	1.01 (0.68,1.50)	0.96 (0.78,1.18)	0.96 (0.77,1.18)	0.94 (0.75,1.19)	0.96 (0.70,1.32)	0.94 (0.71,1.23)	0.92 (0.78,1.09)	0.96 (0.64,1.43)	0.93 (0.73,1.18)	0.92 (0.76,1.11)	0.91 (0.73,1.14)	0.90 (0.70,1.16)	0.89 (0.70,1.15)	0.89 (0.68,1.17)	0.89 (0.68,1.17)	0.88 (0.66,1.18)	0.87 (0.74,1.04)	0.87 (0.71,1.07)	0.87 (0.67,1.14)	0.87 (0.70,1.08)	0.87 (0.73,1.03)	0.87 (0.74,1.02)	0.85 (0.67,1.07)	**0.85 (0.76,0.95)**	0.85 (0.69,1.04)	**0.83 (0.70,0.99)**	**0.80 (0.65,0.98)**	**0.79 (0.63,0.99)**	**0.77 (0.64,0.93)**	**0.77 (0.65,0.92)**	**0.76 (0.63,0.92)**	**0.76 (0.61,0.93)**	**0.73 (0.61,0.88)**	**0.73 (0.63,0.85)**	**0.66 (0.50,0.87)**
1.07 (0.83,1.37)	1.04 (0.83,1.32)	1.03 (0.82,1.30)	1.02 (0.80,1.30)	LM	1.03 (0.70,1.51)	0.98 (0.78,1.23)	0.97 (0.76,1.25)	0.96 (0.78,1.18)	0.98 (0.70,1.37)	0.95 (0.73,1.24)	0.94 (0.77,1.15)	0.98 (0.64,1.48)	0.95 (0.73,1.24)	0.94 (0.75,1.17)	0.93 (0.72,1.20)	0.92 (0.69,1.22)	0.91 (0.69,1.20)	0.91 (0.68,1.22)	0.91 (0.68,1.22)	0.90 (0.66,1.22)	0.89 (0.72,1.10)	0.89 (0.71,1.11)	0.89 (0.67,1.18)	0.89 (0.72,1.09)	0.89 (0.72,1.09)	0.88 (0.72,1.08)	0.86 (0.66,1.12)	0.86 (0.70,1.07)	0.86 (0.70,1.06)	0.84 (0.69,1.03)	**0.81 (0.67,0.98)**	0.80 (0.62,1.04)	**0.79 (0.63,0.98)**	**0.79 (0.64,0.97)**	**0.77 (0.65,0.93)**	**0.77 (0.62,0.96)**	**0.75 (0.60,0.93)**	**0.75 (0.62,0.90)**	**0.67 (0.52,0.87)**
1.04 (0.70,1.55)	1.02 (0.69,1.50)	1.01 (0.69,1.48)	0.99 (0.67,1.47)	0.97 (0.66,1.43)	DLM	0.95 (0.65,1.40)	0.95 (0.64,1.41)	0.94 (0.66,1.33)	0.95 (0.60,1.51)	0.93 (0.61,1.42)	0.92 (0.63,1.33)	0.95 (0.56,1.60)	0.92 (0.61,1.39)	0.91 (0.62,1.34)	0.90 (0.60,1.35)	0.90 (0.59,1.36)	0.89 (0.58,1.35)	0.88 (0.58,1.36)	0.88 (0.58,1.36)	0.87 (0.56,1.36)	0.87 (0.60,1.26)	0.87 (0.59,1.27)	0.87 (0.56,1.33)	0.87 (0.59,1.28)	0.86 (0.60,1.25)	0.86 (0.59,1.25)	0.84 (0.56,1.27)	0.84 (0.57,1.23)	0.84 (0.57,1.24)	0.82 (0.57,1.19)	0.79 (0.56,1.11)	0.78 (0.52,1.17)	0.77 (0.52,1.13)	0.77 (0.53,1.12)	0.75 (0.52,1.10)	0.75 (0.51,1.11)	0.73 (0.50,1.06)	0.73 (0.50,1.05)	**0.66 (0.49,0.87)**
1.09 (0.90,1.33)	1.07 (0.90,1.27)	1.06 (0.90,1.25)	1.04 (0.84,1.29)	1.02 (0.81,1.29)	1.05 (0.72,1.55)	EK	1.00 (0.81,1.23)	0.98 (0.80,1.21)	1.00 (0.73,1.37)	0.98 (0.75,1.27)	0.96 (0.82,1.13)	1.00 (0.67,1.49)	0.97 (0.77,1.23)	0.96 (0.80,1.16)	0.95 (0.76,1.19)	0.94 (0.73,1.21)	0.93 (0.73,1.20)	0.93 (0.71,1.22)	0.93 (0.71,1.22)	0.92 (0.69,1.23)	0.91 (0.77,1.08)	0.91 (0.77,1.08)	0.91 (0.70,1.19)	0.91 (0.74,1.12)	0.91 (0.77,1.07)	0.90 (0.77,1.06)	0.88 (0.70,1.12)	0.88 (0.74,1.06)	0.88 (0.72,1.08)	**0.86 (0.77,0.97)**	0.83 (0.69,1.00)	0.82 (0.66,1.03)	**0.81 (0.67,0.97)**	**0.81 (0.68,0.95)**	**0.79 (0.66,0.95)**	**0.79 (0.64,0.96)**	**0.76 (0.64,0.92)**	**0.76 (0.66,0.89)**	**0.69 (0.53,0.89)**
1.10 (0.87,1.39)	1.07 (0.86,1.33)	1.06 (0.86,1.31)	1.05 (0.84,1.29)	1.03 (0.80,1.31)	1.05 (0.71,1.57)	1.00 (0.81,1.24)	AF	0.99 (0.78,1.24)	1.00 (0.73,1.38)	0.98 (0.75,1.29)	0.97 (0.82,1.14)	1.00 (0.67,1.50)	0.97 (0.77,1.23)	0.96 (0.80,1.17)	0.95 (0.76,1.19)	0.94 (0.73,1.22)	0.93 (0.73,1.20)	0.93 (0.71,1.22)	0.93 (0.71,1.22)	0.92 (0.69,1.24)	0.91 (0.77,1.09)	0.91 (0.74,1.13)	0.91 (0.70,1.20)	0.91 (0.73,1.13)	0.91 (0.76,1.08)	0.91 (0.77,1.07)	0.89 (0.70,1.12)	0.89 (0.74,1.06)	0.88 (0.72,1.09)	0.87 (0.73,1.03)	0.83 (0.68,1.03)	0.82 (0.66,1.03)	**0.81 (0.67,0.98)**	**0.81 (0.67,0.97)**	**0.79 (0.65,0.97)**	**0.79 (0.64,0.97)**	**0.77 (0.64,0.92)**	**0.77 (0.66,0.89)**	**0.69 (0.52,0.91)**
1.11 (0.88,1.40)	1.09 (0.88,1.35)	1.08 (0.87,1.33)	1.06 (0.84,1.33)	1.04 (0.84,1.28)	1.07 (0.75,1.52)	1.02 (0.82,1.25)	1.01 (0.80,1.28)	BM	1.02 (0.73,1.41)	0.99 (0.76,1.31)	0.98 (0.81,1.18)	1.02 (0.67,1.53)	0.99 (0.77,1.27)	0.98 (0.79,1.20)	0.97 (0.76,1.23)	0.96 (0.73,1.25)	0.95 (0.73,1.24)	0.95 (0.71,1.26)	0.95 (0.71,1.26)	0.93 (0.69,1.26)	0.93 (0.76,1.12)	0.93 (0.75,1.14)	0.93 (0.70,1.22)	0.93 (0.75,1.15)	0.92 (0.77,1.10)	0.92 (0.77,1.10)	0.90 (0.70,1.16)	0.90 (0.74,1.10)	0.90 (0.72,1.11)	0.88 (0.74,1.04)	**0.84 (0.77,0.93)**	0.84 (0.66,1.06)	0.82 (0.67,1.01)	**0.82 (0.67,0.99)**	**0.81 (0.66,0.98)**	0.80 (0.64,1.00)	**0.78 (0.63,0.95)**	**0.78 (0.65,0.92)**	**0.70 (0.57,0.86)**
1.09 (0.78,1.52)	1.07 (0.78,1.47)	1.06 (0.77,1.44)	1.04 (0.76,1.43)	1.02 (0.73,1.43)	1.05 (0.66,1.66)	1.00 (0.73,1.37)	1.00 (0.73,1.37)	0.98 (0.71,1.36)	H	0.98 (0.68,1.40)	0.96 (0.72,1.28)	1.00 (0.63,1.59)	0.97 (0.69,1.35)	0.96 (0.71,1.29)	0.95 (0.69,1.31)	0.94 (0.67,1.33)	0.93 (0.66,1.31)	0.93 (0.65,1.32)	0.93 (0.65,1.32)	0.92 (0.63,1.33)	0.91 (0.68,1.22)	0.91 (0.66,1.24)	0.91 (0.64,1.30)	0.91 (0.66,1.25)	0.91 (0.68,1.21)	0.90 (0.68,1.20)	0.88 (0.63,1.23)	0.88 (0.66,1.18)	0.88 (0.64,1.20)	0.86 (0.64,1.15)	0.83 (0.61,1.13)	0.82 (0.59,1.13)	0.80 (0.60,1.08)	0.80 (0.60,1.08)	0.79 (0.58,1.07)	0.79 (0.57,1.07)	0.76 (0.57,1.02)	0.76 (0.58,1.01)	**0.69 (0.48,0.99)**
1.12 (0.85,1.48)	1.09 (0.84,1.43)	1.08 (0.83,1.41)	1.07 (0.81,1.40)	1.05 (0.81,1.36)	1.08 (0.70,1.64)	1.02 (0.79,1.33)	1.02 (0.78,1.34)	1.01 (0.77,1.32)	1.03 (0.72,1.47)	KI	0.98 (0.78,1.24)	1.02 (0.66,1.58)	0.99 (0.74,1.33)	0.98 (0.76,1.27)	0.97 (0.73,1.29)	0.96 (0.71,1.31)	0.95 (0.70,1.29)	0.95 (0.69,1.31)	0.95 (0.69,1.31)	0.94 (0.68,1.29)	0.93 (0.73,1.19)	0.93 (0.73,1.19)	0.93 (0.69,1.25)	0.93 (0.75,1.15)	0.93 (0.73,1.18)	0.93 (0.73,1.17)	0.90 (0.68,1.21)	0.90 (0.71,1.16)	0.90 (0.72,1.12)	0.88 (0.70,1.12)	0.85 (0.66,1.10)	0.84 (0.63,1.11)	0.82 (0.64,1.06)	0.82 (0.65,1.05)	**0.81 (0.67,0.98)**	0.81 (0.63,1.03)	0.78 (0.61,1.00)	**0.78 (0.62,0.98)**	**0.70 (0.51,0.96)**
1.14 (0.94,1.37)	1.11 (0.94,1.31)	1.10 (0.94,1.29)	1.08 (0.92,1.27)	1.06 (0.87,1.30)	1.09 (0.75,1.58)	1.04 (0.89,1.22)	1.04 (0.88,1.22)	1.02 (0.85,1.23)	1.04 (0.78,1.38)	1.02 (0.81,1.28)	AL	1.04 (0.71,1.51)	1.01 (0.83,1.22)	1.00 (0.88,1.14)	0.99 (0.83,1.18)	0.98 (0.79,1.21)	0.97 (0.79,1.19)	0.97 (0.77,1.22)	0.97 (0.77,1.22)	0.95 (0.74,1.23)	0.95 (0.86,1.05)	0.95 (0.81,1.10)	0.94 (0.75,1.19)	0.95 (0.81,1.11)	0.94 (0.85,1.05)	0.94 (0.86,1.02)	0.92 (0.76,1.11)	0.92 (0.82,1.03)	0.92 (0.79,1.07)	0.90 (0.81,1.00)	0.86 (0.74,1.01)	0.85 (0.71,1.02)	**0.84 (0.74,0.95)**	**0.84 (0.75,0.94)**	**0.82 (0.72,0.93)**	**0.82 (0.70,0.95)**	**0.79 (0.70,0.89)**	**0.79 (0.75,0.84)**	**0.72 (0.56,0.91)**
1.10 (0.72,1.66)	1.07 (0.72,1.60)	1.06 (0.71,1.58)	1.04 (0.70,1.56)	1.03 (0.67,1.56)	1.05 (0.62,1.78)	1.00 (0.67,1.50)	1.00 (0.67,1.49)	0.98 (0.65,1.49)	1.00 (0.63,1.60)	0.98 (0.63,1.51)	0.96 (0.66,1.41)	GL	0.97 (0.64,1.47)	0.96 (0.65,1.42)	0.95 (0.63,1.43)	0.94 (0.62,1.44)	0.93 (0.61,1.43)	0.93 (0.60,1.44)	0.93 (0.60,1.44)	0.92 (0.59,1.44)	0.91 (0.62,1.34)	0.91 (0.61,1.36)	0.91 (0.59,1.41)	0.91 (0.61,1.37)	0.91 (0.62,1.33)	0.91 (0.62,1.32)	0.88 (0.58,1.34)	0.89 (0.60,1.30)	0.88 (0.59,1.32)	0.86 (0.59,1.27)	0.83 (0.56,1.24)	0.82 (0.55,1.24)	0.81 (0.55,1.19)	0.81 (0.55,1.19)	0.79 (0.54,1.17)	0.79 (0.53,1.18)	0.76 (0.52,1.13)	0.76 (0.53,1.11)	0.69 (0.44,1.07)
1.13 (0.87,1.46)	1.10 (0.87,1.40)	1.09 (0.86,1.38)	1.07 (0.85,1.36)	1.06 (0.81,1.38)	1.08 (0.72,1.63)	1.03 (0.81,1.31)	1.03 (0.81,1.31)	1.01 (0.79,1.31)	1.03 (0.74,1.44)	1.01 (0.75,1.35)	0.99 (0.82,1.20)	1.03 (0.68,1.56)	AJ	0.99 (0.80,1.23)	0.98 (0.76,1.25)	0.97 (0.74,1.28)	0.96 (0.73,1.26)	0.96 (0.72,1.28)	0.96 (0.72,1.28)	0.95 (0.69,1.29)	0.94 (0.77,1.15)	0.94 (0.74,1.18)	0.94 (0.70,1.25)	0.94 (0.74,1.19)	0.94 (0.76,1.15)	0.93 (0.77,1.13)	0.91 (0.70,1.18)	0.91 (0.74,1.12)	0.91 (0.72,1.15)	0.89 (0.73,1.09)	0.86 (0.68,1.08)	0.85 (0.66,1.09)	0.83 (0.67,1.03)	0.83 (0.68,1.02)	0.82 (0.65,1.02)	0.81 (0.64,1.03)	**0.79 (0.64,0.97)**	**0.79 (0.66,0.95)**	**0.71 (0.53,0.96)**
1.14 (0.92,1.40)	1.11 (0.92,1.35)	1.10 (0.92,1.32)	1.08 (0.90,1.31)	1.06 (0.85,1.33)	1.09 (0.74,1.60)	1.04 (0.86,1.25)	1.04 (0.86,1.25)	1.02 (0.83,1.26)	1.04 (0.77,1.41)	1.02 (0.79,1.31)	1.00 (0.88,1.14)	1.04 (0.70,1.53)	1.01 (0.81,1.25)	AG	0.99 (0.81,1.21)	0.98 (0.78,1.24)	0.97 (0.77,1.22)	0.97 (0.75,1.24)	0.97 (0.75,1.24)	0.96 (0.72,1.26)	0.95 (0.83,1.09)	0.95 (0.79,1.14)	0.95 (0.74,1.22)	0.95 (0.78,1.15)	0.94 (0.82,1.09)	0.94 (0.83,1.07)	0.92 (0.74,1.14)	0.92 (0.79,1.07)	0.92 (0.76,1.10)	0.90 (0.78,1.04)	0.86 (0.72,1.04)	0.85 (0.70,1.05)	**0.84 (0.71,0.98)**	**0.84 (0.72,0.97)**	**0.82 (0.70,0.97)**	**0.82 (0.68,0.98)**	**0.79 (0.68,0.92)**	**0.79 (0.71,0.89)**	**0.72 (0.55,0.93)**
1.15 (0.90,1.47)	1.13 (0.90,1.41)	1.11 (0.89,1.39)	1.10 (0.88,1.37)	1.08 (0.84,1.39)	1.11 (0.74,1.66)	1.05 (0.84,1.32)	1.05 (0.84,1.32)	1.03 (0.81,1.32)	1.05 (0.76,1.46)	1.03 (0.78,1.36)	1.01 (0.85,1.21)	1.05 (0.70,1.58)	1.02 (0.80,1.31)	1.01 (0.83,1.24)	ACF	0.99 (0.76,1.29)	0.98 (0.76,1.27)	0.98 (0.74,1.29)	0.98 (0.74,1.29)	0.97 (0.71,1.31)	0.96 (0.80,1.15)	0.96 (0.77,1.19)	0.96 (0.72,1.27)	0.96 (0.76,1.20)	0.96 (0.79,1.15)	0.95 (0.80,1.14)	0.93 (0.73,1.19)	0.93 (0.77,1.13)	0.93 (0.74,1.16)	0.91 (0.75,1.10)	0.87 (0.70,1.09)	0.86 (0.68,1.10)	0.85 (0.69,1.04)	0.85 (0.70,1.03)	0.83 (0.68,1.03)	0.83 (0.67,1.03)	**0.80 (0.66,0.98)**	**0.80 (0.68,0.95)**	**0.72 (0.54,0.97)**
1.16 (0.89,1.52)	1.14 (0.88,1.47)	1.12 (0.87,1.44)	1.11 (0.86,1.43)	1.09 (0.82,1.44)	1.12 (0.73,1.70)	1.06 (0.82,1.37)	1.06 (0.82,1.37)	1.04 (0.80,1.37)	1.06 (0.75,1.50)	1.04 (0.77,1.41)	1.02 (0.83,1.27)	1.06 (0.69,1.62)	1.03 (0.78,1.35)	1.02 (0.81,1.29)	1.01 (0.78,1.31)	FH	0.99 (0.74,1.32)	0.99 (0.73,1.34)	0.99 (0.73,1.34)	0.98 (0.71,1.35)	0.97 (0.78,1.21)	0.97 (0.75,1.24)	0.97 (0.71,1.31)	0.97 (0.75,1.25)	0.96 (0.77,1.20)	0.96 (0.78,1.19)	0.94 (0.71,1.23)	0.94 (0.75,1.18)	0.94 (0.73,1.20)	0.92 (0.73,1.15)	0.88 (0.69,1.13)	0.87 (0.67,1.14)	0.86 (0.68,1.08)	0.86 (0.68,1.07)	0.84 (0.66,1.07)	0.84 (0.65,1.07)	0.81 (0.65,1.02)	**0.81 (0.66,0.99)**	0.73 (0.54,1.00)
1.17 (0.90,1.53)	1.15 (0.89,1.48)	1.14 (0.89,1.45)	1.12 (0.87,1.44)	1.10 (0.83,1.45)	1.13 (0.74,1.71)	1.07 (0.84,1.38)	1.07 (0.83,1.38)	1.05 (0.81,1.38)	1.08 (0.76,1.51)	1.05 (0.78,1.42)	1.03 (0.84,1.27)	1.07 (0.70,1.64)	1.04 (0.79,1.37)	1.03 (0.82,1.30)	1.02 (0.79,1.32)	1.01 (0.76,1.34)	ACD	1.00 (0.74,1.35)	1.00 (0.74,1.35)	0.99 (0.71,1.36)	0.98 (0.79,1.21)	0.98 (0.76,1.25)	0.98 (0.72,1.32)	0.98 (0.76,1.26)	0.97 (0.78,1.21)	0.97 (0.79,1.20)	0.95 (0.72,1.24)	0.95 (0.76,1.19)	0.95 (0.74,1.21)	0.93 (0.74,1.15)	0.89 (0.70,1.14)	0.88 (0.68,1.14)	0.86 (0.69,1.09)	0.86 (0.69,1.08)	0.85 (0.67,1.08)	0.84 (0.66,1.08)	0.82 (0.65,1.03)	0.82 (0.67,1.00)	0.74 (0.54,1.01)
1.18 (0.88,1.56)	1.15 (0.88,1.51)	1.14 (0.87,1.49)	1.12 (0.86,1.47)	1.10 (0.82,1.48)	1.13 (0.74,1.74)	1.08 (0.82,1.41)	1.07 (0.82,1.41)	1.06 (0.80,1.40)	1.08 (0.75,1.54)	1.05 (0.77,1.45)	1.04 (0.82,1.31)	1.07 (0.70,1.66)	1.04 (0.78,1.39)	1.03 (0.81,1.33)	1.02 (0.77,1.35)	1.01 (0.75,1.37)	1.00 (0.74,1.35)	FJ	1.00 (0.73,1.37)	0.99 (0.71,1.38)	0.98 (0.77,1.24)	0.98 (0.75,1.28)	0.98 (0.71,1.34)	0.98 (0.75,1.29)	0.98 (0.77,1.24)	0.97 (0.77,1.23)	0.95 (0.71,1.27)	0.95 (0.74,1.21)	0.95 (0.73,1.24)	0.93 (0.73,1.18)	0.89 (0.69,1.17)	0.88 (0.67,1.17)	0.87 (0.68,1.11)	0.87 (0.68,1.10)	0.85 (0.66,1.10)	0.85 (0.65,1.10)	0.82 (0.64,1.05)	0.82 (0.66,1.03)	0.74 (0.54,1.02)
1.18 (0.88,1.56)	1.15 (0.88,1.51)	1.14 (0.87,1.49)	1.12 (0.86,1.47)	1.10 (0.82,1.48)	1.13 (0.74,1.74)	1.08 (0.82,1.41)	1.07 (0.82,1.41)	1.06 (0.80,1.40)	1.08 (0.75,1.54)	1.05 (0.77,1.45)	1.04 (0.82,1.31)	1.07 (0.70,1.66)	1.04 (0.78,1.39)	1.03 (0.81,1.33)	1.02 (0.77,1.35)	1.01 (0.75,1.37)	1.00 (0.74,1.35)	1.00 (0.73,1.37)	KM	0.99 (0.71,1.38)	0.98 (0.77,1.24)	0.98 (0.75,1.28)	0.98 (0.71,1.34)	0.98 (0.75,1.29)	0.98 (0.77,1.24)	0.97 (0.77,1.23)	0.95 (0.71,1.27)	0.95 (0.74,1.21)	0.95 (0.73,1.24)	0.93 (0.73,1.18)	0.89 (0.69,1.17)	0.88 (0.67,1.17)	0.87 (0.68,1.11)	0.87 (0.68,1.10)	0.85 (0.66,1.10)	0.85 (0.65,1.10)	0.82 (0.64,1.05)	0.82 (0.66,1.03)	0.74 (0.54,1.02)
1.19 (0.88,1.61)	1.16 (0.87,1.56)	1.15 (0.86,1.54)	1.13 (0.85,1.52)	1.11 (0.82,1.51)	1.14 (0.74,1.78)	1.09 (0.81,1.45)	1.09 (0.81,1.46)	1.07 (0.79,1.45)	1.09 (0.75,1.59)	1.06 (0.77,1.46)	1.05 (0.81,1.36)	1.09 (0.69,1.70)	1.06 (0.77,1.44)	1.05 (0.79,1.38)	1.03 (0.76,1.40)	1.02 (0.74,1.42)	1.01 (0.74,1.40)	1.01 (0.72,1.42)	1.01 (0.72,1.42)	BE	0.99 (0.76,1.29)	0.99 (0.75,1.31)	0.99 (0.76,1.30)	0.99 (0.75,1.30)	0.99 (0.76,1.29)	0.98 (0.76,1.27)	0.96 (0.70,1.31)	0.96 (0.73,1.26)	0.96 (0.75,1.23)	0.94 (0.72,1.22)	0.90 (0.68,1.20)	0.89 (0.66,1.21)	0.88 (0.67,1.16)	0.88 (0.67,1.15)	0.86 (0.67,1.11)	0.86 (0.70,1.05)	0.83 (0.63,1.09)	0.83 (0.65,1.07)	0.75 (0.53,1.05)
1.20 (0.99,1.46)	1.17 (0.98,1.40)	1.16 (0.98,1.37)	1.14 (0.96,1.36)	1.12 (0.91,1.38)	1.15 (0.79,1.68)	1.10 (0.93,1.30)	1.09 (0.92,1.30)	1.08 (0.89,1.31)	1.10 (0.82,1.47)	1.07 (0.84,1.36)	1.06 (0.95,1.17)	1.10 (0.75,1.60)	1.06 (0.87,1.30)	1.05 (0.92,1.21)	1.04 (0.87,1.25)	1.03 (0.83,1.29)	1.02 (0.82,1.27)	1.02 (0.80,1.29)	1.02 (0.80,1.29)	1.01 (0.77,1.31)	AE	1.00 (0.85,1.18)	1.00 (0.79,1.27)	1.00 (0.84,1.19)	1.00 (0.88,1.12)	0.99 (0.90,1.10)	0.97 (0.79,1.18)	0.97 (0.85,1.10)	0.97 (0.82,1.14)	0.95 (0.84,1.07)	0.91 (0.77,1.08)	0.90 (0.75,1.09)	0.88 (0.77,1.01)	0.88 (0.78,1.00)	0.87 (0.75,1.01)	0.86 (0.73,1.02)	**0.84 (0.73,0.95)**	**0.84 (0.77,0.91)**	**0.76 (0.59,0.97)**
1.20 (0.99,1.47)	1.17 (0.98,1.40)	1.16 (0.98,1.38)	1.15 (0.93,1.41)	1.13 (0.90,1.40)	1.16 (0.79,1.70)	1.10 (0.92,1.31)	1.10 (0.89,1.35)	1.08 (0.88,1.33)	1.10 (0.81,1.51)	1.07 (0.84,1.37)	1.06 (0.91,1.23)	1.10 (0.74,1.64)	1.07 (0.84,1.34)	1.06 (0.88,1.27)	1.04 (0.84,1.30)	1.03 (0.81,1.33)	1.02 (0.80,1.31)	1.02 (0.78,1.33)	1.02 (0.78,1.33)	1.01 (0.76,1.34)	1.00 (0.85,1.18)	AC	1.00 (0.77,1.29)	1.00 (0.83,1.20)	1.00 (0.85,1.18)	0.99 (0.85,1.16)	0.97 (0.77,1.22)	0.97 (0.82,1.16)	0.97 (0.81,1.16)	0.95 (0.84,1.08)	0.91 (0.76,1.10)	0.90 (0.72,1.13)	0.89 (0.74,1.06)	0.89 (0.75,1.04)	0.87 (0.75,1.01)	0.87 (0.72,1.05)	0.84 (0.70,1.00)	0.84 (0.73,0.97)	0.76 (0.58,0.98)
1.20 (0.91,1.59)	1.18 (0.90,1.54)	1.16 (0.89,1.51)	1.15 (0.88,1.50)	1.13 (0.85,1.49)	1.16 (0.75,1.77)	1.10 (0.84,1.43)	1.10 (0.84,1.44)	1.08 (0.82,1.43)	1.10 (0.77,1.57)	1.07 (0.80,1.45)	1.06 (0.84,1.33)	1.10 (0.71,1.70)	1.07 (0.80,1.42)	1.06 (0.82,1.36)	1.04 (0.79,1.38)	1.04 (0.76,1.40)	1.02 (0.76,1.38)	1.02 (0.74,1.40)	1.02 (0.74,1.40)	1.01 (0.77,1.32)	1.00 (0.79,1.27)	1.00 (0.78,1.29)	BF	1.00 (0.78,1.28)	1.00 (0.79,1.27)	0.99 (0.79,1.25)	0.97 (0.73,1.30)	0.97 (0.76,1.24)	0.97 (0.78,1.21)	0.95 (0.75,1.20)	0.91 (0.70,1.18)	0.90 (0.68,1.20)	0.89 (0.69,1.14)	0.89 (0.69,1.13)	0.87 (0.69,1.09)	0.87 (0.73,1.03)	0.84 (0.66,1.07)	0.84 (0.67,1.05)	0.76 (0.55,1.04)
1.20 (0.96,1.51)	1.17 (0.95,1.45)	1.16 (0.95,1.42)	1.15 (0.92,1.42)	1.13 (0.92,1.38)	1.16 (0.78,1.70)	1.10 (0.90,1.35)	1.10 (0.88,1.36)	1.08 (0.87,1.34)	1.10 (0.80,1.51)	1.07 (0.87,1.33)	1.06 (0.90,1.24)	1.10 (0.73,1.64)	1.07 (0.84,1.36)	1.06 (0.87,1.28)	1.04 (0.83,1.31)	1.03 (0.80,1.34)	1.02 (0.79,1.32)	1.02 (0.78,1.34)	1.02 (0.78,1.34)	1.01 (0.77,1.33)	1.00 (0.84,1.19)	1.00 (0.83,1.20)	1.00 (0.78,1.28)	DL	1.00 (0.84,1.19)	0.99 (0.84,1.17)	0.97 (0.76,1.23)	0.97 (0.81,1.17)	0.97 (0.84,1.12)	0.95 (0.80,1.12)	0.91 (0.75,1.11)	0.90 (0.72,1.14)	0.89 (0.73,1.07)	0.89 (0.74,1.06)	**0.87 (0.79,0.96)**	0.87 (0.72,1.03)	0.84 (0.70,1.01)	**0.84 (0.72,0.98)**	**0.76 (0.58,0.99)**
1.20 (0.99,1.46)	1.18 (0.99,1.40)	1.17 (0.99,1.38)	1.15 (0.97,1.36)	1.13 (0.92,1.38)	1.16 (0.80,1.67)	1.10 (0.93,1.30)	1.10 (0.92,1.31)	1.08 (0.91,1.29)	1.10 (0.83,1.48)	1.08 (0.85,1.37)	1.06 (0.95,1.18)	1.10 (0.75,1.61)	1.07 (0.87,1.31)	1.06 (0.92,1.22)	1.05 (0.87,1.26)	1.04 (0.83,1.30)	1.03 (0.83,1.28)	1.02 (0.81,1.30)	1.02 (0.81,1.30)	1.01 (0.78,1.32)	1.00 (0.89,1.13)	1.00 (0.85,1.18)	1.00 (0.79,1.27)	1.00 (0.84,1.19)	AM	1.00 (0.90,1.10)	0.97 (0.79,1.19)	0.97 (0.85,1.11)	0.97 (0.82,1.15)	0.95 (0.84,1.07)	0.92 (0.79,1.06)	0.91 (0.75,1.09)	0.89 (0.77,1.02)	0.89 (0.78,1.01)	0.87 (0.75,1.01)	0.87 (0.73,1.03)	**0.84 (0.73,0.96)**	**0.84 (0.77,0.92)**	**0.76 (0.60,0.96)**
1.21 (1.00,1.46)	1.18 (1.00,1.39)	1.17 (1.00,1.37)	1.15 (0.98,1.35)	1.13 (0.93,1.38)	1.16 (0.80,1.69)	1.11 (0.94,1.30)	1.10 (0.94,1.30)	1.09 (0.91,1.30)	1.11 (0.83,1.47)	1.08 (0.86,1.37)	1.06 (0.98,1.16)	1.10 (0.76,1.61)	1.07 (0.88,1.30)	1.06 (0.94,1.21)	1.05 (0.88,1.25)	1.04 (0.84,1.29)	1.03 (0.84,1.27)	1.03 (0.82,1.29)	1.03 (0.82,1.29)	1.02 (0.79,1.31)	1.01 (0.91,1.12)	1.01 (0.86,1.18)	1.01 (0.80,1.26)	1.01 (0.85,1.19)	1.00 (0.91,1.11)	AB	0.98 (0.80,1.18)	0.98 (0.87,1.10)	0.98 (0.84,1.14)	0.95 (0.86,1.06)	0.92 (0.79,1.07)	0.91 (0.76,1.09)	0.89 (0.78,1.01)	0.89 (0.80,1.00)	0.88 (0.76,1.00)	0.87 (0.75,1.01)	**0.84 (0.75,0.95)**	**0.84 (0.79,0.90)**	**0.76 (0.60,0.97)**
1.24 (0.96,1.60)	1.21 (0.95,1.54)	1.20 (0.95,1.51)	1.18 (0.93,1.50)	1.16 (0.89,1.51)	1.19 (0.79,1.80)	1.13 (0.89,1.43)	1.13 (0.89,1.43)	1.11 (0.86,1.43)	1.14 (0.81,1.58)	1.11 (0.83,1.48)	1.09 (0.90,1.32)	1.13 (0.75,1.71)	1.10 (0.85,1.42)	1.09 (0.88,1.35)	1.08 (0.84,1.38)	1.07 (0.81,1.40)	1.06 (0.80,1.39)	1.05 (0.79,1.41)	1.05 (0.79,1.41)	1.04 (0.76,1.42)	1.03 (0.85,1.26)	1.03 (0.82,1.30)	1.03 (0.77,1.38)	1.03 (0.81,1.31)	1.03 (0.84,1.26)	1.02 (0.84,1.24)	AHK	1.00 (0.81,1.23)	1.00 (0.79,1.26)	0.98 (0.80,1.20)	0.94 (0.75,1.19)	0.93 (0.73,1.19)	0.91 (0.74,1.13)	0.91 (0.74,1.12)	0.90 (0.72,1.12)	0.89 (0.71,1.13)	0.86 (0.70,1.07)	0.86 (0.72,1.04)	0.78 (0.58,1.05)
**1.24 (1.01,1.52)**	**1.21 (1.01,1.45)**	1.20 (1.00,1.43)	**1.18 (1.05,1.32)**	1.16 (0.93,1.44)	1.19 (0.81,1.74)	1.13 (0.95,1.35)	1.13 (0.94,1.35)	1.11 (0.91,1.36)	1.13 (0.84,1.52)	1.11 (0.86,1.42)	1.09 (0.97,1.22)	1.13 (0.77,1.66)	1.10 (0.89,1.35)	1.09 (0.94,1.26)	1.07 (0.89,1.30)	1.07 (0.85,1.34)	1.05 (0.84,1.32)	1.05 (0.82,1.34)	1.05 (0.82,1.34)	1.04 (0.79,1.36)	1.03 (0.91,1.17)	1.03 (0.86,1.23)	1.03 (0.81,1.31)	1.03 (0.86,1.24)	1.03 (0.90,1.17)	1.02 (0.91,1.15)	1.00 (0.81,1.23)	G	1.00 (0.84,1.19)	0.98 (0.85,1.12)	0.94 (0.79,1.12)	0.93 (0.77,1.13)	0.91 (0.78,1.06)	0.91 (0.79,1.05)	0.90 (0.76,1.05)	0.89 (0.75,1.06)	0.86 (0.75,1.00)	**0.86 (0.78,0.95)**	0.78 (0.60,1.00)
1.24 (0.99,1.55)	1.21 (0.99,1.49)	1.20 (0.99,1.46)	1.18 (0.96,1.46)	1.16 (0.94,1.43)	1.19 (0.81,1.76)	1.13 (0.93,1.38)	1.13 (0.92,1.40)	1.11 (0.90,1.38)	1.14 (0.83,1.55)	1.11 (0.89,1.38)	1.09 (0.94,1.27)	1.13 (0.76,1.69)	1.10 (0.87,1.39)	1.09 (0.91,1.31)	1.08 (0.86,1.34)	1.07 (0.83,1.37)	1.06 (0.82,1.35)	1.05 (0.81,1.38)	1.05 (0.81,1.38)	1.04 (0.81,1.33)	1.03 (0.88,1.22)	1.03 (0.86,1.23)	1.03 (0.83,1.28)	1.03 (0.89,1.19)	1.03 (0.87,1.22)	1.03 (0.88,1.20)	1.00 (0.79,1.26)	1.00 (0.84,1.20)	BL	0.98 (0.83,1.15)	0.94 (0.78,1.14)	0.93 (0.75,1.16)	0.91 (0.76,1.10)	0.91 (0.77,1.08)	0.90 (0.80,1.00)	0.89 (0.78,1.02)	0.87 (0.72,1.03)	0.87 (0.75,1.00)	0.78 (0.60,1.02)
**1.27 (1.09,1.48)**	**1.24 (1.09,1.40)**	**1.23 (1.09,1.37)**	**1.21 (1.01,1.44)**	1.19 (0.97,1.44)	1.22 (0.84,1.76)	**1.16 (1.03,1.30)**	1.16 (0.97,1.38)	1.14 (0.96,1.35)	1.16 (0.87,1.55)	1.13 (0.90,1.43)	1.11 (1.00,1.24)	1.16 (0.79,1.70)	1.12 (0.92,1.38)	1.11 (0.96,1.29)	1.10 (0.91,1.33)	1.09 (0.87,1.36)	1.08 (0.87,1.34)	1.08 (0.85,1.37)	1.08 (0.85,1.37)	1.06 (0.82,1.39)	1.06 (0.94,1.19)	1.05 (0.93,1.20)	1.05 (0.83,1.34)	1.05 (0.89,1.24)	1.05 (0.93,1.18)	1.05 (0.94,1.17)	1.02 (0.83,1.25)	1.02 (0.90,1.17)	1.02 (0.87,1.20)	K	0.96 (0.83,1.11)	0.95 (0.79,1.15)	0.93 (0.81,1.08)	0.93 (0.83,1.05)	0.92 (0.80,1.05)	0.91 (0.77,1.07)	0.88 (0.77,1.01)	**0.88 (0.81,0.97)**	0.80 (0.63,1.01)
**1.32 (1.07,1.62)**	**1.29 (1.06,1.56)**	**1.27 (1.06,1.53)**	**1.26 (1.02,1.54)**	**1.23 (1.02,1.48)**	1.27 (0.90,1.77)	1.20 (1.00,1.45)	1.20 (0.97,1.48)	**1.18 (1.07,1.31)**	1.21 (0.88,1.65)	1.18 (0.91,1.52)	1.16 (0.99,1.35)	1.20 (0.81,1.79)	1.17 (0.92,1.47)	1.16 (0.96,1.39)	1.14 (0.92,1.43)	1.13 (0.88,1.46)	1.12 (0.88,1.44)	1.12 (0.86,1.46)	1.12 (0.86,1.46)	1.11 (0.83,1.47)	1.10 (0.93,1.30)	1.10 (0.91,1.32)	1.10 (0.84,1.42)	1.10 (0.90,1.33)	1.09 (0.94,1.26)	1.09 (0.93,1.27)	1.06 (0.84,1.34)	1.06 (0.89,1.27)	1.06 (0.88,1.28)	1.04 (0.90,1.20)	M	0.99 (0.79,1.23)	0.97 (0.81,1.16)	0.97 (0.82,1.15)	0.95 (0.80,1.13)	0.95 (0.78,1.15)	0.92 (0.77,1.10)	0.92 (0.80,1.06)	0.83 (0.69,1.00)
**1.33 (1.04,1.70)**	**1.30 (1.04,1.63)**	**1.29 (1.03,1.61)**	**1.27 (1.01,1.59)**	**1.25 (0.97,1.61)**	1.28 (0.85,1.92)	1.22 (0.97,1.52)	1.21 (0.97,1.52)	1.20 (0.94,1.52)	1.22 (0.88,1.69)	1.19 (0.90,1.58)	1.17 (0.98,1.40)	1.21 (0.81,1.83)	1.18 (0.92,1.51)	1.17 (0.96,1.43)	1.16 (0.91,1.46)	1.15 (0.88,1.49)	1.13 (0.87,1.47)	1.13 (0.86,1.50)	1.13 (0.86,1.50)	1.12 (0.83,1.51)	1.11 (0.92,1.34)	1.11 (0.89,1.38)	1.11 (0.84,1.46)	1.11 (0.88,1.39)	1.10 (0.91,1.33)	1.10 (0.92,1.32)	1.07 (0.84,1.38)	1.08 (0.89,1.31)	1.07 (0.86,1.34)	1.05 (0.87,1.27)	1.01 (0.81,1.26)	AH	0.98 (0.80,1.20)	0.98 (0.81,1.19)	0.96 (0.78,1.19)	0.96 (0.77,1.19)	0.93 (0.76,1.13)	0.93 (0.79,1.10)	0.84 (0.63,1.12)
**1.36 (1.10,1.67)**	**1.33 (1.10,1.61)**	**1.31 (1.09,1.58)**	**1.29 (1.07,1.56)**	**1.27 (1.02,1.59)**	1.30 (0.89,1.92)	**1.24 (1.03,1.49)**	**1.24 (1.02,1.50)**	1.22 (0.99,1.50)	1.24 (0.92,1.68)	1.21 (0.94,1.56)	**1.19 (1.05,1.36)**	1.24 (0.84,1.83)	1.20 (0.97,1.49)	**1.19 (1.02,1.40)**	1.18 (0.96,1.44)	1.17 (0.93,1.47)	1.16 (0.92,1.46)	1.15 (0.90,1.48)	1.15 (0.90,1.48)	1.14 (0.87,1.50)	1.13 (0.99,1.30)	1.13 (0.94,1.35)	1.13 (0.88,1.45)	1.13 (0.93,1.37)	1.13 (0.98,1.30)	1.12 (0.99,1.27)	1.10 (0.88,1.36)	1.10 (0.94,1.27)	1.09 (0.91,1.31)	1.07 (0.93,1.24)	1.03 (0.86,1.24)	1.02 (0.83,1.25)	CE	1.00 (0.86,1.16)	0.98 (0.83,1.16)	0.98 (0.81,1.17)	0.95 (0.81,1.10)	0.95 (0.85,1.06)	0.85 (0.66,1.11)
**1.36 (1.12,1.65)**	**1.33 (1.12,1.58)**	**1.31 (1.11,1.55)**	**1.29 (1.08,1.55)**	**1.27 (1.03,1.57)**	1.31 (0.90,1.90)	**1.24 (1.05,1.47)**	**1.24 (1.03,1.48)**	**1.22 (1.01,1.48)**	1.24 (0.93,1.67)	1.21 (0.95,1.55)	**1.20 (1.07,1.34)**	1.24 (0.84,1.82)	1.20 (0.98,1.48)	**1.19 (1.03,1.38)**	1.18 (0.97,1.43)	1.17 (0.93,1.46)	1.16 (0.93,1.44)	1.15 (0.91,1.47)	1.15 (0.91,1.47)	1.14 (0.87,1.49)	1.13 (1.00,1.28)	1.13 (0.96,1.33)	1.13 (0.89,1.44)	1.13 (0.94,1.35)	1.13 (0.99,1.28)	1.12 (1.00,1.26)	1.10 (0.89,1.35)	1.10 (0.96,1.26)	1.10 (0.92,1.30)	1.07 (0.95,1.21)	1.03 (0.87,1.22)	1.02 (0.84,1.24)	1.00 (0.86,1.16)	F	0.98 (0.84,1.15)	0.98 (0.82,1.16)	0.95 (0.82,1.09)	0.95 (0.86,1.04)	0.86 (0.67,1.10)
**1.38 (1.12,1.70)**	**1.35 (1.12,1.63)**	**1.34 (1.12,1.60)**	**1.32 (1.08,1.60)**	**1.29 (1.08,1.55)**	1.33 (0.91,1.94)	**1.26 (1.05,1.51)**	**1.26 (1.03,1.53)**	**1.24 (1.02,1.51)**	1.27 (0.93,1.71)	**1.23 (1.02,1.49)**	**1.22 (1.07,1.38)**	1.26 (0.85,1.87)	1.22 (0.98,1.53)	**1.21 (1.03,1.43)**	1.20 (0.98,1.48)	1.19 (0.94,1.51)	1.18 (0.93,1.49)	1.17 (0.91,1.51)	1.17 (0.91,1.51)	1.16 (0.90,1.50)	1.15 (0.99,1.33)	1.15 (0.99,1.34)	1.15 (0.91,1.44)	1.15 (1.04,1.26)	1.15 (0.99,1.33)	1.14 (1.00,1.31)	1.11 (0.89,1.39)	1.12 (0.95,1.31)	1.11 (1.00,1.24)	1.09 (0.95,1.25)	1.05 (0.89,1.24)	1.04 (0.84,1.28)	1.02 (0.86,1.20)	1.02 (0.87,1.18)	L	0.99 (0.86,1.15)	0.96 (0.82,1.13)	0.96 (0.85,1.09)	0.87 (0.68,1.12)
**1.39 (1.11,1.74)**	**1.36 (1.10,1.67)**	**1.34 (1.10,1.64)**	**1.32 (1.08,1.63)**	**1.30 (1.04,1.62)**	1.34 (0.90,1.97)	**1.27 (1.04,1.55)**	**1.27 (1.03,1.56)**	**1.25 (1.00,1.55)**	1.27 (0.93,1.74)	1.24 (0.97,1.58)	**1.22 (1.05,1.42)**	1.27 (0.85,1.89)	1.23 (0.98,1.55)	**1.22 (1.02,1.47)**	1.21 (0.97,1.50)	1.20 (0.93,1.53)	1.18 (0.92,1.51)	1.18 (0.91,1.54)	1.18 (0.91,1.54)	1.17 (0.95,1.44)	1.16 (0.98,1.37)	1.16 (0.96,1.40)	1.16 (0.97,1.37)	1.16 (0.97,1.38)	1.15 (0.97,1.36)	1.15 (0.99,1.34)	1.12 (0.89,1.41)	1.12 (0.94,1.34)	1.12 (0.98,1.28)	1.10 (0.93,1.29)	1.05 (0.87,1.28)	1.04 (0.84,1.30)	1.02 (0.85,1.23)	1.02 (0.86,1.21)	1.01 (0.87,1.17)	B	0.97 (0.81,1.16)	0.97 (0.84,1.12)	0.87 (0.67,1.14)
**1.43 (1.17,1.76)**	**1.40 (1.16,1.69)**	**1.39 (1.16,1.66)**	**1.37 (1.14,1.64)**	**1.34 (1.08,1.67)**	1.38 (0.94,2.02)	**1.31 (1.09,1.57)**	**1.31 (1.09,1.57)**	**1.29 (1.05,1.58)**	1.31 (0.98,1.76)	1.28 (1.00,1.64)	**1.26 (1.12,1.42)**	1.31 (0.89,1.93)	**1.27 (1.03,1.57)**	**1.26 (1.08,1.47)**	**1.24 (1.02,1.51)**	1.23 (0.98,1.55)	1.22 (0.97,1.53)	1.22 (0.95,1.56)	1.22 (0.95,1.56)	1.20 (0.92,1.58)	**1.19 (1.05,1.36)**	1.19 (1.00,1.42)	1.19 (0.93,1.52)	1.19 (0.99,1.44)	**1.19 (1.04,1.36)**	**1.18 (1.05,1.33)**	1.16 (0.94,1.43)	1.16 (1.00,1.34)	1.16 (0.97,1.38)	1.13 (0.99,1.30)	1.09 (0.91,1.30)	1.08 (0.88,1.31)	1.06 (0.91,1.23)	1.05 (0.92,1.21)	1.04 (0.88,1.22)	1.03 (0.86,1.23)	BH	1.00 (0.90,1.11)	0.90 (0.70,1.16)
**1.43 (1.20,1.71)**	**1.40 (1.20,1.63)**	**1.39 (1.20,1.60)**	**1.37 (1.17,1.59)**	**1.34 (1.11,1.63)**	1.38 (0.95,1.99)	**1.31 (1.13,1.52)**	**1.31 (1.12,1.52)**	**1.29 (1.08,1.53)**	1.31 (0.99,1.73)	**1.28 (1.02,1.61)**	**1.26 (1.19,1.34)**	1.31 (0.90,1.90)	**1.27 (1.06,1.53)**	**1.26 (1.13,1.41)**	**1.24 (1.05,1.47)**	**1.23 (1.01,1.51)**	1.22 (1.00,1.49)	1.22 (0.97,1.52)	1.22 (0.97,1.52)	1.20 (0.94,1.55)	**1.19 (1.10,1.30)**	**1.19 (1.03,1.38)**	1.19 (0.95,1.49)	**1.19 (1.02,1.39)**	**1.19 (1.09,1.30)**	**1.18 (1.12,1.26)**	1.16 (0.96,1.39)	**1.16 (1.05,1.28)**	1.16 (1.00,1.34)	**1.13 (1.03,1.24)**	1.09 (0.94,1.26)	1.08 (0.91,1.27)	1.06 (0.94,1.18)	1.05 (0.96,1.16)	1.04 (0.92,1.17)	1.03 (0.89,1.19)	1.00 (0.90,1.11)	A	0.90 (0.71,1.14)
**1.59 (1.20,2.10)**	**1.55 (1.19,2.02)**	**1.54 (1.18,1.99)**	**1.51 (1.15,2.00)**	**1.49 (1.15,1.93)**	**1.53 (1.15,2.03)**	**1.45 (1.12,1.88)**	**1.45 (1.10,1.91)**	**1.43 (1.16,1.76)**	**1.45 (1.01,2.09)**	**1.42 (1.04,1.94)**	**1.40 (1.10,1.78)**	1.45 (0.93,2.25)	**1.41 (1.05,1.90)**	**1.40 (1.08,1.81)**	**1.38 (1.04,1.84)**	1.37 (1.00,1.86)	1.35 (0.99,1.84)	1.35 (0.98,1.86)	1.35 (0.98,1.86)	1.33 (0.95,1.87)	**1.32 (1.03,1.70)**	**1.32 (1.02,1.71)**	1.32 (0.96,1.82)	**1.32 (1.01,1.72)**	**1.32 (1.04,1.67)**	**1.31 (1.03,1.67)**	1.28 (0.95,1.72)	1.28 (1.00,1.65)	1.28 (0.98,1.67)	1.25 (0.99,1.58)	1.21 (1.00,1.45)	1.19 (0.89,1.59)	1.17 (0.90,1.52)	1.17 (0.91,1.50)	1.15 (0.90,1.48)	1.14 (0.87,1.50)	1.11 (0.86,1.43)	1.11 (0.88,1.40)	D

Bold and underlined values indicate statistically significant differences.

Table A11. League table for PSQI.

BM	2.26 (-4.00,8.52)	**2.86 (0.32,5.41)**	3.24 (-3.02,9.50)	3.70 (-2.50,9.90)	4.39 (-1.41,10.19)	4.90 (-0.94,10.73)	5.05 (-0.43,10.53)	**5.07 (0.46,9.67)**	**5.26 (0.05,10.47)**	**5.51 (1.26,9.76)**	5.73 (-0.44,11.90)	5.74 (-0.42,11.90)	**6.00 (0.79,11.22)**	**6.07 (0.60,11.53)**	**6.22 (0.58,11.86)**	6.67 (-0.07,13.42)	**6.44 (1.19,11.70)**	**6.52 (1.05,11.98)**	**6.67 (0.52,12.82)**	**6.72 (1.42,12.01)**	**7.28 (1.14,13.42)**	**7.34 (1.24,13.44)**	**8.20 (1.07,15.33)**	**8.21 (3.13,13.29)**	**10.77 (2.04,19.50)**	**9.60 (3.30,15.90)**	**11.30 (2.66,19.93)**	**13.10 (5.16,21.05)**
-2.26 (-8.52,4.00)	EK	0.60 (-5.12,6.33)	0.98 (-3.93,5.89)	1.44 (-3.41,6.29)	2.13 (-2.18,6.45)	2.64 (-2.28,7.55)	2.79 (-1.69,7.27)	2.81 (-1.44,7.05)	3.00 (-0.48,6.48)	3.25 (-3.41,9.90)	3.47 (-1.84,8.78)	3.48 (-1.81,8.77)	3.74 (-0.42,7.90)	3.81 (-0.66,8.27)	3.96 (-0.73,8.64)	4.41 (-1.56,10.38)	4.18 (-0.03,8.40)	4.26 (-0.21,8.72)	4.41 (-0.88,9.69)	**4.45 (0.20,8.71)**	5.02 (-0.26,10.29)	5.08 (-0.15,10.30)	5.94 (-0.46,12.34)	**5.95 (1.96,9.93)**	**8.51 (0.37,16.65)**	**7.33 (1.88,12.79)**	**9.04 (0.99,17.08)**	**10.84 (3.55,18.14)**
**-2.86 (-5.41,-0.32)**	-0.60 (-6.33,5.12)	M	0.38 (-5.34,6.09)	0.84 (-4.82,6.50)	1.53 (-3.68,6.74)	2.03 (-3.22,7.29)	2.19 (-2.66,7.04)	2.20 (-1.64,6.05)	2.40 (-2.15,6.94)	2.64 (-0.76,6.05)	2.86 (-2.76,8.49)	2.87 (-2.73,8.48)	3.14 (-1.41,7.70)	3.20 (-1.63,8.04)	3.35 (-1.68,8.39)	3.81 (-2.44,10.06)	3.58 (-1.02,8.18)	3.65 (-1.18,8.49)	3.80 (-1.80,9.41)	3.85 (-0.79,8.50)	4.41 (-1.18,10.01)	4.47 (-1.07,10.02)	5.34 (-1.33,12.00)	**5.34 (0.95,9.74)**	7.91 (-0.44,16.26)	**6.73 (0.97,12.50)**	**8.43 (0.18,16.69)**	**10.24 (2.71,17.77)**
-3.24 (-9.50,3.02)	-0.98 (-5.89,3.93)	-0.38 (-6.09,5.34)	ABGN	0.46 (-4.38,5.30)	1.15 (-3.15,5.46)	1.66 (-3.25,6.56)	1.81 (-2.66,6.28)	1.83 (-2.41,6.06)	2.02 (-1.45,5.49)	2.27 (-4.38,8.92)	2.49 (-2.81,7.79)	2.50 (-2.79,7.78)	2.76 (-1.39,6.91)	2.83 (-1.63,7.29)	2.98 (-1.70,7.65)	3.43 (-2.53,9.39)	3.20 (-1.00,7.41)	3.28 (-1.18,7.73)	3.43 (-1.85,8.71)	3.48 (-0.77,7.72)	4.04 (-1.23,9.31)	4.10 (-1.12,9.32)	4.96 (-1.43,11.35)	**4.97 (0.99,8.95)**	7.53 (-0.61,15.67)	**6.36 (0.90,11.81)**	**8.06 (0.02,16.10)**	**9.86 (2.57,17.15)**
-3.70 (-9.90,2.50)	-1.44 (-6.29,3.41)	-0.84 (-6.50,4.82)	-0.46 (-5.30,4.38)	KL	0.69 (-3.54,4.92)	1.20 (-3.64,6.03)	1.35 (-3.05,5.74)	1.37 (-2.79,5.52)	1.56 (-1.82,4.93)	1.81 (-4.79,8.41)	2.03 (-3.21,7.27)	2.04 (-3.18,7.26)	2.30 (-1.76,6.37)	2.37 (-2.02,6.75)	2.52 (-2.09,7.12)	2.97 (-2.93,8.88)	2.74 (-1.38,6.87)	2.82 (-1.57,7.20)	2.97 (-2.24,8.18)	3.01 (-1.15,7.18)	3.58 (-1.63,8.78)	3.64 (-1.52,8.79)	4.50 (-1.84,10.84)	**4.51 (0.62,8.40)**	7.07 (-1.03,15.17)	**5.90 (0.51,11.28)**	7.60 (-0.40,15.59)	**9.40 (2.16,16.64)**
-4.39 (-10.19,1.41)	-2.13 (-6.45,2.18)	-1.53 (-6.74,3.68)	-1.15 (-5.46,3.15)	-0.69 (-4.92,3.54)	AK	0.50 (-3.80,4.81)	0.66 (-3.15,4.46)	0.67 (-2.85,4.20)	0.87 (-1.68,3.42)	1.12 (-5.11,7.34)	1.34 (-3.42,6.09)	1.35 (-3.39,6.08)	1.61 (-1.81,5.03)	1.68 (-2.12,5.47)	1.83 (-2.22,5.87)	2.28 (-3.20,7.76)	2.05 (-1.44,5.54)	2.12 (-1.66,5.91)	2.28 (-2.45,7.00)	2.32 (-1.22,5.86)	2.89 (-1.83,7.60)	2.95 (-1.71,7.61)	3.81 (-2.14,9.75)	**3.82 (0.61,7.03)**	6.38 (-1.41,14.17)	**5.20 (0.28,10.12)**	6.91 (-0.78,14.59)	**8.71 (1.81,15.61)**
-4.90 (-10.73,0.94)	-2.64 (-7.55,2.28)	-2.03 (-7.29,3.22)	-1.66 (-6.56,3.25)	-1.20 (-6.03,3.64)	-0.50 (-4.81,3.80)	FG	0.15 (-3.38,3.68)	0.17 (-3.42,3.76)	0.36 (-3.11,3.83)	0.61 (-5.65,6.87)	0.83 (-3.70,5.37)	0.84 (-3.67,5.36)	1.11 (-2.00,4.22)	1.17 (-2.34,4.68)	1.32 (-2.46,5.10)	1.78 (-3.51,7.06)	1.55 (-0.98,4.08)	1.62 (-1.89,5.13)	1.77 (-2.73,6.28)	1.82 (-1.42,5.06)	2.38 (-2.11,6.88)	2.44 (-1.99,6.88)	3.30 (-2.47,9.07)	**3.31 (0.44,6.19)**	5.88 (-1.78,13.54)	4.70 (-0.01,9.41)	6.40 (-1.15,13.95)	**8.21 (1.46,14.96)**
-5.05 (-10.53,0.43)	-2.79 (-7.27,1.69)	-2.19 (-7.04,2.66)	-1.81 (-6.28,2.66)	-1.35 (-5.74,3.05)	-0.66 (-4.46,3.15)	-0.15 (-3.68,3.38)	AF	0.02 (-2.95,2.98)	0.21 (-2.61,3.03)	0.46 (-5.47,6.38)	0.68 (-3.38,4.74)	0.69 (-3.35,4.73)	0.95 (-1.41,3.32)	1.02 (-1.86,3.89)	1.17 (-2.03,4.37)	1.62 (-3.27,6.51)	1.39 (-1.06,3.85)	1.47 (-1.40,4.34)	1.62 (-2.41,5.65)	1.67 (-0.87,4.20)	2.23 (-1.79,6.25)	2.29 (-1.66,6.24)	3.15 (-2.26,8.56)	**3.16 (1.11,5.21)**	5.72 (-1.67,13.11)	**4.55 (0.29,8.80)**	6.25 (-1.03,13.53)	**8.05 (1.61,14.50)**
**-5.07 (-9.67,-0.46)**	-2.81 (-7.05,1.44)	-2.20 (-6.05,1.64)	-1.83 (-6.06,2.41)	-1.37 (-5.52,2.79)	-0.67 (-4.20,2.85)	-0.17 (-3.76,3.42)	-0.02 (-2.98,2.95)	F	0.19 (-2.24,2.62)	0.44 (-4.69,5.57)	0.66 (-3.45,4.77)	0.67 (-3.42,4.76)	0.94 (-1.51,3.39)	1.00 (-1.94,3.95)	1.15 (-2.11,4.42)	1.61 (-3.33,6.54)	1.38 (-1.16,3.92)	1.45 (-1.49,4.39)	1.60 (-2.48,5.68)	1.65 (-0.96,4.26)	2.21 (-1.86,6.28)	2.27 (-1.73,6.27)	3.13 (-2.31,8.58)	**3.14 (1.00,5.29)**	5.71 (-1.71,13.12)	**4.53 (0.23,8.83)**	6.23 (-1.08,13.54)	**8.04 (1.56,14.51)**
**-5.26 (-10.47,-0.05)**	-3.00 (-6.48,0.48)	-2.40 (-6.94,2.15)	-2.02 (-5.49,1.45)	-1.56 (-4.93,1.82)	-0.87 (-3.42,1.68)	-0.36 (-3.83,3.11)	-0.21 (-3.03,2.61)	-0.19 (-2.62,2.24)	K	0.25 (-5.43,5.92)	0.47 (-3.54,4.48)	0.48 (-3.51,4.47)	0.74 (-1.54,3.02)	0.81 (-2.00,3.61)	0.96 (-2.18,4.09)	1.41 (-3.44,6.26)	1.18 (-1.19,3.56)	1.26 (-1.54,4.06)	1.41 (-2.57,5.39)	1.46 (-1.00,3.91)	2.02 (-1.95,5.99)	2.08 (-1.82,5.98)	2.94 (-2.43,8.31)	**2.95 (1.00,4.90)**	5.51 (-1.85,12.88)	**4.34 (0.13,8.54)**	6.04 (-1.21,13.29)	**7.84 (1.43,14.25)**
**-5.51 (-9.76,-1.26)**	-3.25 (-9.90,3.41)	-2.64 (-6.05,0.76)	-2.27 (-8.92,4.38)	-1.81 (-8.41,4.79)	-1.12 (-7.34,5.11)	-0.61 (-6.87,5.65)	-0.46 (-6.38,5.47)	-0.44 (-5.57,4.69)	-0.25 (-5.92,5.43)	D	0.22 (-6.35,6.79)	0.23 (-6.33,6.79)	0.50 (-5.19,6.18)	0.56 (-5.35,6.47)	0.71 (-5.37,6.79)	1.16 (-5.95,8.28)	0.94 (-4.79,6.66)	1.01 (-4.90,6.92)	1.16 (-5.39,7.71)	1.21 (-4.55,6.96)	1.77 (-4.77,8.32)	1.83 (-4.67,8.34)	2.69 (-4.79,10.17)	2.70 (-2.86,8.26)	5.26 (-3.75,14.28)	4.09 (-2.61,10.78)	5.79 (-3.14,14.72)	7.60 (-0.66,15.8[illegible])
-5.73 (-11.90,0.44)	-3.47 (-8.78,1.84)	-2.86 (-8.49,2.76)	-2.49 (-7.79,2.81)	-2.03 (-7.27,3.21)	-1.34 (-6.09,3.42)	-0.83 (-5.37,3.70)	-0.68 (-4.74,3.38)	-0.66 (-4.77,3.45)	-0.47 (-4.48,3.54)	-0.22 (-6.79,6.35)	AM	0.01 (-4.93,4.95)	0.28 (-3.43,3.98)	0.34 (-3.71,4.39)	0.49 (-3.80,4.77)	0.94 (-4.72,6.60)	0.72 (-3.05,4.48)	0.79 (-3.26,4.83)	0.94 (-3.99,5.87)	0.99 (-2.82,4.80)	1.55 (-3.37,6.47)	1.61 (-3.26,6.48)	2.47 (-3.64,8.58)	2.48 (-1.03,5.99)	5.04 (-2.88,12.96)	3.87 (-1.25,8.99)	5.57 (-2.25,13.39)	**7.37 (0.33,14.42)**
-5.74 (-11.90,0.42)	-3.48 (-8.77,1.81)	-2.87 (-8.48,2.73)	-2.50 (-7.78,2.79)	-2.04 (-7.26,3.18)	-1.35 (-6.08,3.39)	-0.84 (-5.36,3.67)	-0.69 (-4.73,3.35)	-0.67 (-4.76,3.42)	-0.48 (-4.47,3.51)	-0.23 (-6.79,6.33)	-0.01 (-4.95,4.93)	FJ	0.27 (-3.41,3.94)	0.33 (-3.69,4.35)	0.48 (-3.78,4.74)	0.93 (-4.71,6.58)	0.71 (-3.03,4.44)	0.78 (-3.24,4.80)	0.93 (-3.98,5.84)	0.98 (-2.81,4.76)	1.54 (-3.37,6.45)	1.60 (-3.25,6.45)	2.46 (-3.64,8.56)	2.47 (-1.01,5.95)	5.03 (-2.87,12.94)	3.86 (-1.24,8.96)	5.56 (-2.25,13.36)	**7.36 (0.33,14.39)**
**-6.00 (-11.22,-0.79)**	-3.74 (-7.90,0.42)	-3.14 (-7.70,1.41)	-2.76 (-6.91,1.39)	-2.30 (-6.37,1.76)	-1.61 (-5.03,1.81)	-1.11 (-4.22,2.00)	-0.95 (-3.32,1.41)	-0.94 (-3.39,1.51)	-0.74 (-3.02,1.54)	-0.50 (-6.18,5.19)	-0.28 (-3.98,3.43)	-0.27 (-3.94,3.41)	AB	0.06 (-2.28,2.41)	0.21 (-2.52,2.95)	0.67 (-3.93,5.27)	0.44 (-1.37,2.25)	0.51 (-1.83,2.85)	0.66 (-3.00,4.33)	0.71 (-1.20,2.62)	1.27 (-2.38,4.93)	1.33 (-2.25,4.92)	2.19 (-2.95,7.34)	**2.20 (1.02,3.39)**	4.77 (-2.43,11.97)	3.59 (-0.32,7.51)	5.29 (-1.79,12.38)	**7.10 (0.88,13.32)**
**-6.07 (-11.53,-0.60)**	-3.81 (-8.27,0.66)	-3.20 (-8.04,1.63)	-2.83 (-7.29,1.63)	-2.37 (-6.75,2.02)	-1.68 (-5.47,2.12)	-1.17 (-4.68,2.34)	-1.02 (-3.89,1.86)	-1.00 (-3.95,1.94)	-0.81 (-3.61,2.00)	-0.56 (-6.47,5.35)	-0.34 (-4.39,3.71)	-0.33 (-4.35,3.69)	-0.06 (-2.41,2.28)	AE	0.15 (-3.03,3.33)	0.60 (-4.27,5.48)	0.38 (-2.06,2.81)	0.45 (-2.40,3.30)	0.60 (-3.41,4.61)	0.65 (-1.86,3.16)	1.21 (-2.79,5.21)	1.27 (-2.66,5.21)	2.13 (-3.27,7.53)	**2.14 (0.12,4.16)**	4.70 (-2.68,12.09)	3.53 (-0.71,7.77)	5.23 (-2.04,12.50)	**7.04 (0.60,13.47)**
**-6.22 (-11.86,-0.58)**	-3.96 (-8.64,0.73)	-3.35 (-8.39,1.68)	-2.98 (-7.65,1.70)	-2.52 (-7.12,2.09)	-1.83 (-5.87,2.22)	-1.32 (-5.10,2.46)	-1.17 (-4.37,2.03)	-1.15 (-4.42,2.11)	-0.96 (-4.09,2.18)	-0.71 (-6.79,5.37)	-0.49 (-4.77,3.80)	-0.48 (-4.74,3.78)	-0.21 (-2.95,2.52)	-0.15 (-3.33,3.03)	AH	0.45 (-4.62,5.53)	0.23 (-2.59,3.04)	0.30 (-2.88,3.48)	0.45 (-3.80,4.70)	0.50 (-2.38,3.38)	1.06 (-3.18,5.30)	1.12 (-3.06,5.30)	1.98 (-3.60,7.56)	1.99 (-0.47,4.45)	4.55 (-2.96,12.07)	3.38 (-1.09,7.85)	5.08 (-2.33,12.49)	**6.89 (0.30,13.47)**
-6.67 (-13.42,0.07)	-4.41 (-10.38,1.56)	-3.81 (-10.06,2.44)	-3.43 (-9.39,2.53)	-2.97 (-8.88,2.93)	-2.28 (-7.76,3.20)	-1.78 (-7.06,3.51)	-1.62 (-6.51,3.27)	-1.61 (-6.54,3.33)	-1.41 (-6.26,3.44)	-1.16 (-8.28,5.95)	-0.94 (-6.60,4.72)	-0.93 (-6.58,4.71)	-0.67 (-5.27,3.93)	-0.60 (-5.48,4.27)	-0.45 (-5.53,4.62)	DL	-0.23 (-4.87,4.42)	-0.16 (-5.03,4.72)	-0.00 (-5.64,5.63)	0.04 (-4.15,4.23)	0.61 (-5.02,6.23)	0.67 (-4.91,6.25)	1.53 (-2.62,5.67)	1.54 (-2.90,5.98)	4.10 (-2.43,10.63)	**2.92 (0.51,5.34)**	4.62 (-1.78,11.03)	**6.43 (1.00,11.87)**
**-6.44 (-11.70,-1.19)**	-4.18 (-8.40,0.03)	-3.58 (-8.18,1.02)	-3.20 (-7.41,1.00)	-2.74 (-6.87,1.38)	-2.05 (-5.54,1.44)	-1.55 (-4.08,0.98)	-1.39 (-3.85,1.06)	-1.38 (-3.92,1.16)	-1.18 (-3.56,1.19)	-0.94 (-6.66,4.79)	-0.72 (-4.48,3.05)	-0.71 (-4.44,3.03)	-0.44 (-2.25,1.37)	-0.38 (-2.81,2.06)	-0.23 (-3.04,2.59)	0.23 (-4.42,4.87)	G	0.07 (-2.36,2.50)	0.22 (-3.50,3.95)	0.27 (-1.75,2.29)	0.83 (-2.88,4.55)	0.89 (-2.75,4.54)	1.75 (-3.43,6.94)	**1.76 (0.40,3.13)**	4.33 (-2.90,11.56)	3.15 (-0.82,7.12)	4.85 (-2.26,11.97)	**6.66 (0.40,12.92)**
**-6.52 (-11.98,-1.05)**	-4.26 (-8.72,0.21)	-3.65 (-8.49,1.18)	-3.28 (-7.73,1.18)	-2.82 (-7.20,1.57)	-2.12 (-5.91,1.66)	-1.62 (-5.13,1.89)	-1.47 (-4.34,1.40)	-1.45 (-4.39,1.49)	-1.26 (-4.06,1.54)	-1.01 (-6.92,4.90)	-0.79 (-4.83,3.26)	-0.78 (-4.80,3.24)	-0.51 (-2.85,1.83)	-0.45 (-3.30,2.40)	-0.30 (-3.48,2.88)	0.16 (-4.72,5.03)	-0.07 (-2.50,2.36)	AG	0.15 (-3.86,4.16)	0.20 (-2.31,2.71)	0.76 (-3.24,4.76)	0.82 (-3.11,4.76)	1.68 (-3.71,7.08)	1.69 (-0.32,3.71)	4.26 (-3.13,11.64)	3.08 (-1.16,7.32)	4.78 (-2.49,12.05)	**6.59 (0.16,13.02)**
**-6.67 (-12.82,-0.52)**	-4.41 (-9.69,0.88)	-3.80 (-9.41,1.80)	-3.43 (-8.71,1.85)	-2.97 (-8.18,2.24)	-2.28 (-7.00,2.45)	-1.77 (-6.28,2.73)	-1.62 (-5.65,2.41)	-1.60 (-5.68,2.48)	-1.41 (-5.39,2.57)	-1.16 (-7.71,5.39)	-0.94 (-5.87,3.99)	-0.93 (-5.84,3.98)	-0.66 (-4.33,3.00)	-0.60 (-4.61,3.41)	-0.45 (-4.70,3.80)	0.00 (-5.63,5.64)	-0.22 (-3.95,3.50)	-0.15 (-4.16,3.86)	ACD	0.05 (-3.73,3.82)	0.61 (-4.29,5.51)	0.67 (-4.17,5.51)	1.53 (-4.56,7.62)	1.54 (-1.93,5.01)	4.10 (-3.80,12.01)	2.93 (-2.17,8.02)	4.63 (-3.17,12.43)	6.43 (-0.59,13.4[illegible])
**-6.72 (-12.01,-1.42)**	**-4.45 (-8.71,-0.20)**	-3.85 (-8.50,0.79)	-3.48 (-7.72,0.77)	-3.01 (-7.18,1.15)	-2.32 (-5.86,1.22)	-1.82 (-5.06,1.42)	-1.67 (-4.20,0.87)	-1.65 (-4.26,0.96)	-1.46 (-3.91,1.00)	-1.21 (-6.96,4.55)	-0.99 (-4.80,2.82)	-0.98 (-4.76,2.81)	-0.71 (-2.62,1.20)	-0.65 (-3.16,1.86)	-0.50 (-3.38,2.38)	-0.04 (-4.23,4.15)	-0.27 (-2.29,1.75)	-0.20 (-2.71,2.31)	-0.05 (-3.82,3.73)	AL	0.56 (-3.20,4.33)	0.62 (-3.07,4.32)	1.48 (-3.30,6.27)	1.49 (0.00,2.99)	4.06 (-2.89,11.01)	2.88 (-0.55,6.31)	4.58 (-2.25,11.41)	**6.39 (0.46,12.32)**
**-7.28 (-13.42,-1.14)**	-5.02 (-10.29,0.26)	-4.41 (-10.01,1.18)	-4.04 (-9.31,1.23)	-3.58 (-8.78,1.63)	-2.89 (-7.60,1.83)	-2.38 (-6.88,2.11)	-2.23 (-6.25,1.79)	-2.21 (-6.28,1.86)	-2.02 (-5.99,1.95)	-1.77 (-8.32,4.77)	-1.55 (-6.47,3.37)	-1.54 (-6.45,3.37)	-1.27 (-4.93,2.38)	-1.21 (-5.21,2.79)	-1.06 (-5.30,3.18)	-0.61 (-6.23,5.02)	-0.83 (-4.55,2.88)	-0.76 (-4.76,3.24)	-0.61 (-5.51,4.29)	-0.56 (-4.33,3.20)	H	0.06 (-4.77,4.89)	0.92 (-5.16,7.00)	0.93 (-2.53,4.39)	3.49 (-4.40,11.39)	2.32 (-2.77,7.40)	4.02 (-3.78,11.81)	5.82 (-1.19,12.8[illegible])
**-7.34 (-13.44,-1.24)**	-5.08 (-10.30,0.15)	-4.47 (-10.02,1.07)	-4.10 (-9.32,1.12)	-3.64 (-8.79,1.52)	-2.95 (-7.61,1.71)	-2.44 (-6.88,1.99)	-2.29 (-6.24,1.66)	-2.27 (-6.27,1.73)	-2.08 (-5.98,1.82)	-1.83 (-8.34,4.67)	-1.61 (-6.48,3.26)	-1.60 (-6.45,3.25)	-1.33 (-4.92,2.25)	-1.27 (-5.21,2.66)	-1.12 (-5.30,3.06)	-0.67 (-6.25,4.91)	-0.89 (-4.54,2.75)	-0.82 (-4.76,3.11)	-0.67 (-5.51,4.17)	-0.62 (-4.32,3.07)	-0.06 (-4.89,4.77)	AJ	0.86 (-5.18,6.90)	0.87 (-2.51,4.25)	3.43 (-4.43,11.30)	2.26 (-2.77,7.29)	3.96 (-3.80,11.72)	5.76 (-1.22,12.7[illegible])
**-8.20 (-15.33,-1.07)**	-5.94 (-12.34,0.46)	-5.34 (-12.00,1.33)	-4.96 (-11.35,1.43)	-4.50 (-10.84,1.84)	-3.81 (-9.75,2.14)	-3.30 (-9.07,2.47)	-3.15 (-8.56,2.26)	-3.13 (-8.58,2.31)	-2.94 (-8.31,2.43)	-2.69 (-10.17,4.79)	-2.47 (-8.58,3.64)	-2.46 (-8.56,3.64)	-2.19 (-7.34,2.95)	-2.13 (-7.53,3.27)	-1.98 (-7.56,3.60)	-1.53 (-5.67,2.62)	-1.75 (-6.94,3.43)	-1.68 (-7.08,3.71)	-1.53 (-7.62,4.56)	-1.48 (-6.27,3.30)	-0.92 (-7.00,5.16)	-0.86 (-6.90,5.18)	BL	0.01 (-4.99,5.01)	2.57 (-2.49,7.63)	1.40 (-1.97,4.76)	3.10 (-1.80,8.00)	**4.90 (1.37,8.43)**
**-8.21 (-13.29,-3.13)**	**-5.95 (-9.93,-1.96)**	**-5.34 (-9.74,-0.95)**	**-4.97 (-8.95,-0.99)**	**-4.51 (-8.40,-0.62)**	**-3.82 (-7.03,-0.61)**	**-3.31 (-6.19,-0.44)**	**-3.16 (-5.21,-1.11)**	**-3.14 (-5.29,-1.00)**	-2.95 (-4.90,-1.00)	-2.70 (-8.26,2.86)	-2.48 (-5.99,1.03)	-2.47 (-5.95,1.01)	**-2.20 (-3.39,-1.02)**	**-2.14 (-4.16,-0.12)**	-1.99 (-4.45,0.47)	-1.54 (-5.98,2.90)	**-1.76 (-3.13,-0.40)**	-1.69 (-3.71,0.32)	-1.54 (-5.01,1.93)	-1.49 (-2.99,-0.00)	-0.93 (-4.39,2.53)	-0.87 (-4.25,2.51)	-0.01 (-5.01,4.99)	A	2.56 (-4.54,9.66)	1.39 (-2.34,5.12)	3.09 (-3.90,10.07)	4.89 (-1.21,11.0[illegible])
**-10.77 (-19.50,-2.04)**	**-8.51 (-16.65,-0.37)**	-7.91 (-16.26,0.44)	-7.53 (-15.67,0.61)	-7.07 (-15.17,1.03)	-6.38 (-14.17,1.41)	-5.88 (-13.54,1.78)	-5.72 (-13.11,1.67)	-5.71 (-13.12,1.71)	-5.51 (-12.88,1.85)	-5.26 (-14.28,3.75)	-5.04 (-12.96,2.88)	-5.03 (-12.94,2.87)	-4.77 (-11.97,2.43)	-4.70 (-12.09,2.68)	-4.55 (-12.07,2.96)	-4.10 (-10.63,2.43)	-4.33 (-11.56,2.90)	-4.26 (-11.64,3.13)	-4.10 (-12.01,3.80)	-4.06 (-11.01,2.89)	-3.49 (-11.39,4.40)	-3.43 (-11.30,4.43)	-2.57 (-7.63,2.49)	-2.56 (-9.66,4.54)	BF	-1.18 (-7.24,4.89)	0.52 (-4.45,5.50)	2.33 (-1.29,5.95)
**-9.60 (-15.90,-3.30)**	**-7.33 (-12.79,-1.88)**	**-6.73 (-12.50,-0.97)**	**-6.36 (-11.81,-0.90)**	**-5.90 (-11.28,-0.51)**	**-5.20 (-10.12,-0.28)**	-4.70 (-9.41,0.01)	**-4.55 (-8.80,-0.29)**	**-4.53 (-8.83,-0.23)**	**-4.34 (-8.54,-0.13)**	-4.09 (-10.78,2.61)	-3.87 (-8.99,1.25)	-3.86 (-8.96,1.24)	-3.59 (-7.51,0.32)	-3.53 (-7.77,0.71)	-3.38 (-7.85,1.09)	**-2.92 (-5.34,-0.51)**	-3.15 (-7.12,0.82)	-3.08 (-7.32,1.16)	-2.93 (-8.02,2.17)	-2.88 (-6.31,0.55)	-2.32 (-7.40,2.77)	-2.26 (-7.29,2.77)	-1.40 (-4.76,1.97)	-1.39 (-5.12,2.34)	1.18 (-4.89,7.24)	L	1.70 (-4.24,7.64)	3.51 (-1.36,8.38)
**-11.30 (-19.93,-2.66)**	**-9.04 (-17.08,-0.99)**	**-8.43 (-16.69,-0.18)**	**-8.06 (-16.10,-0.02)**	-7.60 (-15.59,0.40)	-6.91 (-14.59,0.78)	-6.40 (-13.95,1.15)	-6.25 (-13.53,1.03)	-6.23 (-13.54,1.08)	-6.04 (-13.29,1.21)	-5.79 (-14.72,3.14)	-5.57 (-13.39,2.25)	-5.56 (-13.36,2.25)	-5.29 (-12.38,1.79)	-5.23 (-12.50,2.04)	-5.08 (-12.49,2.33)	-4.62 (-11.03,1.78)	-4.85 (-11.97,2.26)	-4.78 (-12.05,2.49)	-4.63 (-12.43,3.17)	-4.58 (-11.41,2.25)	-4.02 (-11.81,3.78)	-3.96 (-11.72,3.80)	-3.10 (-8.00,1.80)	-3.09 (-10.07,3.90)	-0.52 (-5.50,4.45)	-1.70 (-7.64,4.24)	BE	1.81 (-1.60,5.21)
**-13.10 (-21.05,-5.16)**	**-10.84 (-18.14,-3.55)**	**-10.24 (-17.77,-2.71)**	**-9.86 (-17.15,-2.57)**	**-9.40 (-16.64,-2.16)**	**-8.71 (-15.61,-1.81)**	**-8.21 (-14.96,-1.46)**	**-8.05 (-14.50,-1.61)**	**-8.04 (-14.51,-1.56)**	**-7.84 (-14.25,-1.43)**	-7.60 (-15.85,0.66)	**-7.37 (-14.42,-0.33)**	**-7.36 (-14.39,-0.33)**	**-7.10 (-13.32,-0.88)**	**-7.04 (-13.47,-0.60)**	**-6.89 (-13.47,-0.30)**	**-6.43 (-11.87,-1.00)**	**-6.66 (-12.92,-0.40)**	**-6.59 (-13.02,-0.16)**	-6.43 (-13.46,0.59)	-6.39 (-12.32,-0.46)	-5.82 (-12.84,1.19)	-5.76 (-12.74,1.22)	**-4.90 (-8.43,-1.37)**	-4.89 (-11.00,1.21)	-2.33 (-5.95,1.29)	-3.51 (-8.38,1.36)	-1.81 (-5.21,1.60)	B

Bold and underlined values indicate statistically significant differences.

Table A12. League table for change in PSQI scores.

B	2.28 (-4.30,8.85)	2.00 (-2.08,6.09)	4.43 (-2.96,11.83)	3.55 (-2.35,9.46)	2.24 (-2.00,6.48)	5.24 (-3.21,13.70)	4.83 (0.54,9.11)	5.78 (-2.68,14.25)	6.30 (-0.89,13.48)	6.40 (-1.16,13.97)	6.46 (-1.31,14.23)	6.72 (-1.24,14.67)	6.74 (-0.98,14.45)	6.85 (-0.93,14.62)	6.88 (-1.63,15.40)	6.94 (-1.52,15.41)	7.43 (-2.43,17.30)	7.47 (-0.39,15.33)	**7.82 (0.29,15.35)**	7.99 (-0.08,16.07)	8.06 (-0.05,16.18)	**8.00 (0.21,15.79)**	**9.06 (0.29,17.84)**	**9.15 (0.41,17.89)**	**10.05 (1.05,19.05)**	**10.17 (1.38,18.97)**	**12.92 (3.43,22.41)**
-2.28 (-8.85,4.30)	DL	-0.27 (-8.01,7.46)	2.16 (-3.19,7.50)	1.28 (-1.62,4.17)	-0.04 (-7.86,7.79)	2.97 (-3.77,9.70)	2.55 (-2.45,7.55)	3.51 (-3.24,10.25)	4.02 (-1.03,9.07)	4.13 (-1.45,9.71)	4.18 (-1.67,10.04)	4.44 (-1.65,10.54)	4.46 (-1.32,10.23)	4.57 (-1.29,10.43)	4.61 (-2.21,11.42)	4.67 (-2.08,11.42)	5.16 (-3.28,13.60)	5.19 (-0.78,11.16)	**5.55 (0.02,11.08)**	5.72 (-0.53,11.96)	5.79 (-0.51,12.09)	5.72 (-0.15,11.60)	6.79 (-0.34,13.92)	6.88 (-0.21,13.97)	**7.77 (0.37,15.18)**	**7.90 (0.75,15.05)**	**10.64 (2.65,18.63)**
-2.00 (-6.09,2.08)	0.27 (-7.46,8.01)	BE	2.43 (-6.01,10.87)	1.55 (-5.62,8.72)	0.24 (-5.65,6.12)	3.24 (-6.14,12.62)	2.82 (-3.09,8.73)	3.78 (-5.61,13.17)	4.29 (-3.97,12.55)	4.40 (-4.19,12.99)	4.45 (-4.32,13.22)	4.71 (-4.22,13.65)	4.73 (-3.99,13.45)	4.84 (-3.93,13.61)	4.88 (-4.56,14.32)	4.94 (-4.45,14.33)	5.43 (-5.24,16.10)	5.46 (-3.38,14.31)	5.82 (-2.74,14.38)	5.99 (-3.05,15.03)	6.06 (-3.01,15.13)	6.00 (-2.79,14.78)	7.06 (-2.61,16.73)	7.15 (-2.49,16.79)	8.05 (-1.83,17.92)	8.17 (-1.51,17.85)	**10.91 (0.59,21.23)**
-4.43 (-11.83,2.96)	-2.16 (-7.50,3.19)	-2.43 (-10.87,6.01)	A	-0.88 (-5.38,3.61)	-2.19 (-10.72,6.33)	0.81 (-3.29,4.91)	0.39 (-5.66,6.45)	1.35 (-2.76,5.46)	**1.86 (0.09,3.63)**	**1.97 (0.37,3.57)**	2.02 (-0.37,4.41)	2.28 (-0.64,5.21)	**2.30 (0.12,4.48)**	**2.41 (0.01,4.81)**	2.45 (-1.77,6.67)	2.51 (-1.61,6.63)	3.00 (-3.53,9.53)	**3.03 (0.38,5.69)**	**3.39 (1.97,4.81)**	**3.56 (0.32,6.80)**	**3.63 (0.30,6.96)**	**3.57 (1.13,6.00)**	4.63 (-0.09,9.35)	**4.72 (0.06,9.38)**	**5.62 (0.49,10.74)**	**5.74 (0.99,10.49)**	**8.48 (2.54,14.43)**
-3.55 (-9.46,2.35)	-1.28 (-4.17,1.62)	-1.55 (-8.72,5.62)	0.88 (-3.61,5.38)	L	-1.31 (-8.58,5.96)	1.69 (-4.39,7.78)	1.27 (-2.81,5.35)	2.23 (-3.86,8.32)	2.74 (-1.39,6.88)	2.85 (-1.92,7.62)	2.90 (-2.18,7.99)	3.17 (-2.20,8.53)	3.18 (-1.81,8.18)	3.29 (-1.80,8.39)	3.33 (-2.84,9.50)	3.39 (-2.71,9.49)	3.88 (-4.05,11.81)	3.92 (-1.30,9.13)	4.27 (-0.44,8.98)	4.44 (-1.10,9.98)	4.51 (-1.09,10.11)	4.45 (-0.66,9.56)	5.51 (-1.01,12.03)	5.60 (-0.87,12.07)	6.50 (-0.32,13.31)	**6.62 (0.08,13.16)**	**9.36 (1.91,16.82)**
-2.24 (-6.48,2.00)	0.04 (-7.79,7.86)	-0.24 (-6.12,5.65)	2.19 (-6.33,10.72)	1.31 (-5.96,8.58)	BF	3.00 (-6.46,12.46)	2.59 (-3.44,8.61)	3.54 (-5.92,13.01)	4.05 (-4.29,12.40)	4.16 (-4.51,12.84)	4.22 (-4.64,13.07)	4.48 (-4.54,13.49)	4.49 (-4.31,13.29)	4.60 (-4.25,13.46)	4.64 (-4.87,14.16)	4.70 (-4.76,14.17)	5.19 (-5.55,15.93)	5.23 (-3.70,14.15)	5.58 (-3.06,14.22)	5.75 (-3.37,14.87)	5.82 (-3.33,14.98)	5.76 (-3.11,14.62)	6.82 (-2.92,16.57)	6.91 (-2.80,16.63)	7.81 (-2.14,17.76)	7.93 (-1.83,17.69)	**10.68 (0.28,21.07)**
-5.24 (-13.70,3.21)	-2.97 (-9.70,3.77)	-3.24 (-12.62,6.14)	-0.81 (-4.91,3.29)	-1.69 (-7.78,4.39)	-3.00 (-12.46,6.46)	H	-0.42 (-7.73,6.89)	0.54 (-5.27,6.35)	1.05 (-3.42,5.52)	1.16 (-3.24,5.56)	1.21 (-3.53,5.96)	1.47 (-3.57,6.51)	1.49 (-3.16,6.14)	1.60 (-3.15,6.36)	1.64 (-4.25,7.53)	1.70 (-4.11,7.51)	2.19 (-5.52,9.90)	2.22 (-2.66,7.11)	2.58 (-1.76,6.92)	2.75 (-2.48,7.97)	2.82 (-2.47,8.11)	2.76 (-2.01,7.53)	3.82 (-2.43,10.07)	3.91 (-2.29,10.12)	4.81 (-1.76,11.37)	4.93 (-1.35,11.21)	**7.67 (0.45,14.89)**
**-4.83 (-9.11,-0.54)**	-2.55 (-7.55,2.45)	-2.82 (-8.73,3.09)	-0.39 (-6.45,5.66)	-1.27 (-5.35,2.81)	-2.59 (-8.61,3.44)	0.42 (-6.89,7.73)	BL	0.96 (-6.36,8.28)	1.47 (-4.33,7.26)	1.58 (-4.69,7.84)	1.63 (-4.88,8.14)	1.89 (-4.83,8.62)	1.91 (-4.53,8.34)	2.02 (-4.49,8.53)	2.06 (-5.32,9.44)	2.12 (-5.20,9.44)	2.61 (-6.30,11.51)	2.64 (-3.97,9.25)	3.00 (-3.22,9.22)	3.17 (-3.70,10.03)	3.24 (-3.67,10.15)	3.17 (-3.35,9.70)	4.24 (-3.44,11.91)	4.33 (-3.31,11.97)	5.22 (-2.71,13.16)	5.35 (-2.35,13.04)	8.09 (-0.39,16.57)
-5.78 (-14.25,2.68)	-3.51 (-10.25,3.24)	-3.78 (-13.17,5.61)	-1.35 (-5.46,2.76)	-2.23 (-8.32,3.86)	-3.54 (-13.01,5.92)	-0.54 (-6.35,5.27)	-0.96 (-8.28,6.36)	ACD	0.51 (-3.97,4.99)	0.62 (-3.80,5.03)	0.67 (-4.08,5.43)	0.93 (-4.11,5.98)	0.95 (-3.71,5.61)	1.06 (-3.70,5.83)	1.10 (-4.80,7.00)	1.16 (-4.66,6.98)	1.65 (-6.07,9.37)	1.68 (-3.21,6.58)	2.04 (-2.31,6.39)	2.21 (-3.02,7.44)	2.28 (-3.02,7.57)	2.22 (-2.56,7.00)	3.28 (-2.98,9.54)	3.37 (-2.84,9.58)	4.27 (-2.31,10.84)	4.39 (-1.89,10.67)	7.13 (-0.09,14.36)
-6.30 (-13.48,0.89)	-4.02 (-9.07,1.03)	-4.29 (-12.55,3.97)	**-1.86 (-3.63,-0.09)**	-2.74 (-6.88,1.39)	-4.05 (-12.40,4.29)	-1.05 (-5.52,3.42)	-1.47 (-7.26,4.33)	-0.51 (-4.99,3.97)	AL	0.11 (-2.28,2.50)	0.16 (-2.81,3.14)	0.42 (-3.00,3.85)	0.44 (-2.37,3.25)	0.55 (-2.43,3.54)	0.59 (-3.99,5.17)	0.65 (-3.84,5.13)	1.14 (-5.63,7.91)	1.17 (-2.02,4.36)	1.53 (-0.74,3.80)	1.70 (-1.99,5.39)	1.77 (-2.01,5.54)	1.70 (-1.31,4.72)	2.77 (-2.27,7.81)	2.86 (-2.12,7.84)	3.75 (-1.67,9.18)	3.88 (-1.19,8.95)	**6.62 (0.42,12.82)**
-6.40 (-13.97,1.16)	-4.13 (-9.71,1.45)	-4.40 (-12.99,4.19)	**-1.97 (-3.57,-0.37)**	-2.85 (-7.62,1.92)	-4.16 (-12.84,4.51)	-1.16 (-5.56,3.24)	-1.58 (-7.84,4.69)	-0.62 (-5.03,3.80)	-0.11 (-2.50,2.28)	G	0.05 (-2.82,2.93)	0.32 (-3.02,3.65)	0.33 (-2.38,3.04)	0.44 (-2.45,3.33)	0.48 (-4.04,5.00)	0.54 (-3.88,4.96)	1.03 (-5.70,7.76)	1.06 (-2.03,4.16)	1.42 (-0.72,3.56)	1.59 (-2.02,5.20)	1.66 (-1.26,4.59)	1.60 (-1.32,4.51)	2.66 (-2.32,7.64)	2.75 (-2.17,7.67)	3.65 (-1.72,9.02)	3.77 (-1.24,8.79)	**6.51 (0.36,12.67)**
-6.46 (-14.23,1.31)	-4.18 (-10.04,1.67)	-4.45 (-13.22,4.32)	-2.02 (-4.41,0.37)	-2.90 (-7.99,2.18)	-4.22 (-13.07,4.64)	-1.21 (-5.96,3.53)	-1.63 (-8.14,4.88)	-0.67 (-5.43,4.08)	-0.16 (-3.14,2.81)	-0.05 (-2.93,2.82)	AG	0.26 (-3.52,4.04)	0.28 (-2.96,3.51)	0.39 (-3.00,3.78)	0.43 (-4.43,5.28)	0.49 (-4.28,5.25)	0.98 (-5.98,7.93)	1.01 (-2.56,4.58)	1.37 (-1.41,4.14)	1.54 (-2.49,5.56)	1.61 (-2.50,5.71)	1.54 (-1.87,4.95)	2.61 (-2.68,7.90)	2.70 (-2.54,7.93)	3.59 (-2.06,9.25)	3.72 (-1.60,9.03)	**6.46 (0.05,12.86)**
-6.72 (-14.67,1.24)	-4.44 (-10.54,1.65)	-4.71 (-13.65,4.22)	-2.28 (-5.21,0.64)	-3.17 (-8.53,2.20)	-4.48 (-13.49,4.54)	-1.47 (-6.51,3.57)	-1.89 (-8.62,4.83)	-0.93 (-5.98,4.11)	-0.42 (-3.85,3.00)	-0.32 (-3.65,3.02)	-0.26 (-4.04,3.52)	AH	0.02 (-3.64,3.67)	0.13 (-3.66,3.92)	0.17 (-4.97,5.31)	0.23 (-4.83,5.28)	0.71 (-6.44,7.87)	0.75 (-3.20,4.70)	1.11 (-2.15,4.36)	1.28 (-3.09,5.64)	1.35 (-3.09,5.78)	1.28 (-2.53,5.09)	2.34 (-3.21,7.90)	2.44 (-3.06,7.94)	3.33 (-2.57,9.23)	3.46 (-2.13,9.04)	6.20 (-0.43,12.82)
-6.74 (-14.45,0.98)	-4.46 (-10.23,1.32)	-4.73 (-13.45,3.99)	**-2.30 (-4.48,-0.12)**	-3.18 (-8.18,1.81)	-4.49 (-13.29,4.31)	-1.49 (-6.14,3.16)	-1.91 (-8.34,4.53)	-0.95 (-5.61,3.71)	-0.44 (-3.25,2.37)	-0.33 (-3.04,2.38)	-0.28 (-3.51,2.96)	-0.02 (-3.67,3.64)	K	0.11 (-3.13,3.36)	0.15 (-4.61,4.90)	0.21 (-4.45,4.87)	0.70 (-6.05,7.44)	0.73 (-2.40,3.87)	1.09 (-1.51,3.69)	1.26 (-1.97,4.49)	1.33 (-2.66,5.31)	1.27 (-2.00,4.53)	2.33 (-1.86,6.51)	2.42 (-1.69,6.53)	3.32 (-2.08,8.71)	3.44 (-0.78,7.66)	**6.18 (0.01,12.36)**
-6.85 (-14.62,0.93)	-4.57 (-10.43,1.29)	-4.84 (-13.61,3.93)	**-2.41 (-4.81,-0.01)**	-3.29 (-8.39,1.80)	-4.60 (-13.46,4.25)	-1.60 (-6.36,3.15)	-2.02 (-8.53,4.49)	-1.06 (-5.83,3.70)	-0.55 (-3.54,2.43)	-0.44 (-3.33,2.45)	-0.39 (-3.78,3.00)	-0.13 (-3.92,3.66)	-0.11 (-3.36,3.13)	AE	0.04 (-4.82,4.90)	0.10 (-4.67,4.87)	0.59 (-6.37,7.55)	0.62 (-2.96,4.20)	0.98 (-1.81,3.77)	1.15 (-2.88,5.18)	1.22 (-2.89,5.33)	1.15 (-2.27,4.57)	2.22 (-3.08,7.51)	2.31 (-2.93,7.55)	3.20 (-2.46,8.86)	3.33 (-2.00,8.65)	6.07 (-0.34,12.48)
-6.88 (-15.40,1.63)	-4.61 (-11.42,2.21)	-4.88 (-14.32,4.56)	-2.45 (-6.67,1.77)	-3.33 (-9.50,2.84)	-4.64 (-14.16,4.87)	-1.64 (-7.53,4.25)	-2.06 (-9.44,5.32)	-1.10 (-7.00,4.80)	-0.59 (-5.17,3.99)	-0.48 (-5.00,4.04)	-0.43 (-5.28,4.43)	-0.17 (-5.31,4.97)	-0.15 (-4.90,4.61)	-0.04 (-4.90,4.82)	AM	0.06 (-5.84,5.96)	0.55 (-7.23,8.33)	0.58 (-4.40,5.57)	0.94 (-3.52,5.40)	1.11 (-4.21,6.43)	1.18 (-4.20,6.56)	1.12 (-3.76,5.99)	2.18 (-4.15,8.51)	2.27 (-4.02,8.56)	3.17 (-3.48,9.81)	3.29 (-3.07,9.65)	6.03 (-1.26,13.32)
-6.94 (-15.41,1.52)	-4.67 (-11.42,2.08)	-4.94 (-14.33,4.45)	-2.51 (-6.63,1.61)	-3.39 (-9.49,2.71)	-4.70 (-14.17,4.76)	-1.70 (-7.51,4.11)	-2.12 (-9.44,5.20)	-1.16 (-6.98,4.66)	-0.65 (-5.13,3.84)	-0.54 (-4.96,3.88)	-0.49 (-5.25,4.28)	-0.23 (-5.28,4.83)	-0.21 (-4.87,4.45)	-0.10 (-4.87,4.67)	-0.06 (-5.96,5.84)	FJ	0.49 (-7.23,8.21)	0.52 (-4.38,5.42)	0.88 (-3.48,5.24)	1.05 (-4.19,6.29)	1.12 (-4.18,6.42)	1.06 (-3.73,5.84)	2.12 (-4.15,8.38)	2.21 (-4.01,8.43)	3.11 (-3.47,9.68)	3.23 (-3.06,9.52)	5.97 (-1.26,13.20)
-7.43 (-17.30,2.43)	-5.16 (-13.60,3.28)	-5.43 (-16.10,5.24)	-3.00 (-9.53,3.53)	-3.88 (-11.81,4.05)	-5.19 (-15.93,5.55)	-2.19 (-9.90,5.52)	-2.61 (-11.51,6.30)	-1.65 (-9.37,6.07)	-1.14 (-7.91,5.63)	-1.03 (-7.76,5.70)	-0.98 (-7.93,5.98)	-0.71 (-7.87,6.44)	-0.70 (-7.44,6.05)	-0.59 (-7.55,6.37)	-0.55 (-8.33,7.23)	-0.49 (-8.21,7.23)	D	0.03 (-5.94,6.01)	0.39 (-6.29,7.08)	0.56 (-6.66,7.78)	0.63 (-6.70,7.96)	0.57 (-6.40,7.54)	1.63 (-6.31,9.57)	1.72 (-6.18,9.62)	2.62 (-1.44,6.68)	2.74 (-5.21,10.70)	**5.48 (0.43,10.54)**
-7.47 (-15.33,0.39)	-5.19 (-11.16,0.78)	-5.46 (-14.31,3.38)	**-3.03 (-5.69,-0.38)**	-3.92 (-9.13,1.30)	-5.23 (-14.15,3.70)	-2.22 (-7.11,2.66)	-2.64 (-9.25,3.97)	-1.68 (-6.58,3.21)	-1.17 (-4.36,2.02)	-1.06 (-4.16,2.03)	-1.01 (-4.58,2.56)	-0.75 (-4.70,3.20)	-0.73 (-3.87,2.40)	-0.62 (-4.20,2.96)	-0.58 (-5.57,4.40)	-0.52 (-5.42,4.38)	-0.03 (-6.01,5.94)	F	0.36 (-2.65,3.36)	0.53 (-3.54,4.59)	0.60 (-3.67,4.86)	0.53 (-3.07,4.13)	1.60 (-3.63,6.82)	1.69 (-3.49,6.86)	2.58 (-1.81,6.98)	2.71 (-2.55,7.96)	**5.45 (0.12,10.77)**
**-7.82 (-15.35,-0.29)**	**-5.55 (-11.08,-0.02)**	-5.82 (-14.38,2.74)	**-3.39 (-4.81,-1.97)**	-4.27 (-8.98,0.44)	-5.58 (-14.22,3.06)	-2.58 (-6.92,1.76)	-3.00 (-9.22,3.22)	-2.04 (-6.39,2.31)	-1.53 (-3.80,0.74)	-1.42 (-3.56,0.72)	-1.37 (-4.14,1.41)	-1.11 (-4.36,2.15)	-1.09 (-3.69,1.51)	-0.98 (-3.77,1.81)	-0.94 (-5.40,3.52)	-0.88 (-5.24,3.48)	-0.39 (-7.08,6.29)	-0.36 (-3.36,2.65)	AB	0.17 (-3.36,3.70)	0.24 (-3.38,3.86)	0.18 (-2.64,2.99)	1.24 (-3.69,6.17)	1.33 (-3.54,6.20)	2.23 (-3.09,7.54)	2.35 (-2.61,7.31)	5.09 (-1.02,11.20)
-7.99 (-16.07,0.08)	-5.72 (-11.96,0.53)	-5.99 (-15.03,3.05)	**-3.56 (-6.80,-0.32)**	-4.44 (-9.98,1.10)	-5.75 (-14.87,3.37)	-2.75 (-7.97,2.48)	-3.17 (-10.03,3.70)	-2.21 (-7.44,3.02)	-1.70 (-5.39,1.99)	-1.59 (-5.20,2.02)	-1.54 (-5.56,2.49)	-1.28 (-5.64,3.09)	-1.26 (-4.49,1.97)	-1.15 (-5.18,2.88)	-1.11 (-6.43,4.21)	-1.05 (-6.29,4.19)	-0.56 (-7.78,6.66)	-0.53 (-4.59,3.54)	-0.17 (-3.70,3.36)	AK	0.07 (-4.58,4.72)	0.01 (-4.04,4.06)	1.07 (-4.22,6.36)	1.16 (-4.07,6.39)	2.06 (-3.92,8.04)	2.18 (-3.13,7.50)	4.92 (-1.77,11.62)
-8.06 (-16.18,0.05)	-5.79 (-12.09,0.51)	-6.06 (-15.13,3.01)	**-3.63 (-6.96,-0.30)**	-4.51 (-10.11,1.09)	-5.82 (-14.98,3.33)	-2.82 (-8.11,2.47)	-3.24 (-10.15,3.67)	-2.28 (-7.57,3.02)	-1.77 (-5.54,2.01)	-1.66 (-4.59,1.26)	-1.61 (-5.71,2.50)	-1.35 (-5.78,3.09)	-1.33 (-5.31,2.66)	-1.22 (-5.33,2.89)	-1.18 (-6.56,4.20)	-1.12 (-6.42,4.18)	-0.63 (-7.96,6.70)	-0.60 (-4.86,3.67)	-0.24 (-3.86,3.38)	-0.07 (-4.72,4.58)	FG	-0.06 (-4.19,4.06)	1.00 (-4.78,6.78)	1.09 (-4.64,6.82)	1.99 (-4.13,8.10)	2.11 (-3.69,7.91)	4.85 (-1.96,11.67)
**-8.00 (-15.79,-0.21)**	-5.72 (-11.60,0.15)	-6.00 (-14.78,2.79)	**-3.57 (-6.00,-1.13)**	-4.45 (-9.56,0.66)	-5.76 (-14.62,3.11)	-2.76 (-7.53,2.01)	-3.17 (-9.70,3.35)	-2.22 (-7.00,2.56)	-1.70 (-4.72,1.31)	-1.60 (-4.51,1.32)	-1.54 (-4.95,1.87)	-1.28 (-5.09,2.53)	-1.27 (-4.53,2.00)	-1.15 (-4.57,2.27)	-1.12 (-5.99,3.76)	-1.06 (-5.84,3.73)	-0.57 (-7.54,6.40)	-0.53 (-4.13,3.07)	-0.18 (-2.99,2.64)	-0.01 (-4.06,4.04)	0.06 (-4.06,4.19)	AF	1.06 (-4.25,6.37)	1.15 (-4.10,6.41)	2.05 (-3.62,7.72)	2.17 (-3.16,7.51)	4.92 (-1.50,11.34)
**-9.06 (-17.84,-0.29)**	-6.79 (-13.92,0.34)	-7.06 (-16.73,2.61)	-4.63 (-9.35,0.09)	-5.51 (-12.03,1.01)	-6.82 (-16.57,2.92)	-3.82 (-10.07,2.43)	-4.24 (-11.91,3.44)	-3.28 (-9.54,2.98)	-2.77 (-7.81,2.27)	-2.66 (-7.64,2.32)	-2.61 (-7.90,2.68)	-2.34 (-7.90,3.21)	-2.33 (-6.51,1.86)	-2.22 (-7.51,3.08)	-2.18 (-8.51,4.15)	-2.12 (-8.38,4.15)	-1.63 (-9.57,6.31)	-1.60 (-6.82,3.63)	-1.24 (-6.17,3.69)	-1.07 (-6.36,4.22)	-1.00 (-6.78,4.78)	-1.06 (-6.37,4.25)	KL	0.09 (-5.78,5.96)	0.99 (-5.84,7.81)	1.11 (-4.83,7.05)	3.85 (-3.60,11.31)
**-9.15 (-17.89,-0.41)**	-6.88 (-13.97,0.21)	-7.15 (-16.79,2.49)	**-4.72 (-9.38,-0.06)**	-5.60 (-12.07,0.87)	-6.91 (-16.63,2.80)	-3.91 (-10.12,2.29)	-4.33 (-11.97,3.31)	-3.37 (-9.58,2.84)	-2.86 (-7.84,2.12)	-2.75 (-7.67,2.17)	-2.70 (-7.93,2.54)	-2.44 (-7.94,3.06)	-2.42 (-6.53,1.69)	-2.31 (-7.55,2.93)	-2.27 (-8.56,4.02)	-2.21 (-8.43,4.01)	-1.72 (-9.62,6.18)	-1.69 (-6.86,3.49)	-1.33 (-6.20,3.54)	-1.16 (-6.39,4.07)	-1.09 (-6.82,4.64)	-1.15 (-6.41,4.10)	-0.09 (-5.96,5.78)	ABGN	0.90 (-5.89,7.68)	1.02 (-4.87,6.91)	3.76 (-3.66,11.18)
**-10.05 (-19.05,-1.05)**	**-7.77 (-15.18,-0.37)**	-8.05 (-17.92,1.83)	**-5.62 (-10.74,-0.49)**	-6.50 (-13.31,0.32)	-7.81 (-17.76,2.14)	-4.81 (-11.37,1.76)	-5.22 (-13.16,2.71)	-4.27 (-10.84,2.31)	-3.75 (-9.18,1.67)	-3.65 (-9.02,1.72)	-3.59 (-9.25,2.06)	-3.33 (-9.23,2.57)	-3.32 (-8.71,2.08)	-3.20 (-8.86,2.46)	-3.17 (-9.81,3.48)	-3.11 (-9.68,3.47)	-2.62 (-6.68,1.44)	-2.58 (-6.98,1.81)	-2.23 (-7.54,3.09)	-2.06 (-8.04,3.92)	-1.99 (-8.10,4.13)	-2.05 (-7.72,3.62)	-0.99 (-7.81,5.84)	-0.90 (-7.68,5.89)	M	0.12 (-6.72,6.97)	2.87 (-0.14,5.88)
**-10.17 (-18.97,-1.38)**	**-7.90 (-15.05,-0.75)**	-8.17 (-17.85,1.51)	**-5.74 (-10.49,-0.99)**	**-6.62 (-13.16,-0.08)**	-7.93 (-17.69,1.83)	-4.93 (-11.21,1.35)	-5.35 (-13.04,2.35)	-4.39 (-10.67,1.89)	-3.88 (-8.95,1.19)	-3.77 (-8.79,1.24)	-3.72 (-9.03,1.60)	-3.46 (-9.04,2.13)	-3.44 (-7.66,0.78)	-3.33 (-8.65,2.00)	-3.29 (-9.65,3.07)	-3.23 (-9.52,3.06)	-2.74 (-10.70,5.21)	-2.71 (-7.96,2.55)	-2.35 (-7.31,2.61)	-2.18 (-7.50,3.13)	-2.11 (-7.91,3.69)	-2.17 (-7.51,3.16)	-1.11 (-7.05,4.83)	-1.02 (-6.91,4.87)	-0.12 (-6.97,6.72)	EK	2.74 (-4.74,10.22)
**-12.92 (-22.41,-3.43)**	**-10.64 (-18.63,-2.65)**	**-10.91 (-21.23,-0.59)**	**-8.48 (-14.43,-2.54)**	**-9.36 (-16.82,-1.91)**	**-10.68 (-21.07,-0.28)**	**-7.67 (-14.89,-0.45)**	-8.09 (-16.57,0.39)	-7.13 (-14.36,0.09)	**-6.62 (-12.82,-0.42)**	**-6.51 (-12.67,-0.36)**	**-6.46 (-12.86,-0.05)**	-6.20 (-12.82,0.43)	**-6.18 (-12.36,-0.01)**	-6.07 (-12.48,0.34)	-6.03 (-13.32,1.26)	-5.97 (-13.20,1.26)	**-5.48 (-10.54,-0.43)**	**-5.45 (-10.77,-0.12)**	-5.09 (-11.20,1.02)	-4.92 (-11.62,1.77)	-4.85 (-11.67,1.96)	-4.92 (-11.34,1.50)	-3.85 (-11.31,3.60)	-3.76 (-11.18,3.66)	-2.87 (-5.88,0.14)	-2.74 (-10.22,4.74)	BM

Bold and underlined values indicate statistically significant differences.

Table A13. League table for SQ.

KL	0.39 (-1.03,1.81)	1.11 (-0.01,2.23)	1.14 (-0.62,2.91)	1.25 (-0.50,3.00)	1.32 (-0.44,3.09)	1.36 (-0.10,2.81)	1.39 (-0.36,3.15)	1.51 (-0.21,3.24)	1.65 (-0.12,3.43)	**1.64 (0.08,3.20)**	**1.72 (0.39,3.06)**	**1.98 (0.41,3.55)**
-0.39 (-1.81,1.03)	F	0.72 (-0.16,1.59)	0.75 (-0.69,2.20)	0.86 (-0.57,2.29)	0.93 (-0.51,2.38)	0.97 (-0.08,2.01)	1.00 (-0.43,2.43)	1.12 (-0.28,2.52)	1.26 (-0.20,2.72)	**1.25 (0.05,2.44)**	**1.33 (0.46,2.20)**	**1.59 (0.38,2.79)**
-1.11 (-2.23,0.01)	-0.72 (-1.59,0.16)	K	0.03 (-1.33,1.40)	0.14 (-1.20,1.49)	0.21 (-1.15,1.58)	0.25 (-0.68,1.18)	0.28 (-1.06,1.63)	0.40 (-0.91,1.72)	0.54 (-0.84,1.92)	0.53 (-0.56,1.62)	0.61 (-0.11,1.34)	0.87 (-0.23,1.97)
-1.14 (-2.91,0.62)	-0.75 (-2.20,0.69)	-0.03 (-1.40,1.33)	AL	0.11 (-1.51,1.73)	0.18 (-1.45,1.81)	0.21 (-1.07,1.50)	0.25 (-1.37,1.87)	0.37 (-1.22,1.96)	0.51 (-1.13,2.15)	0.49 (-0.91,1.90)	0.58 (-0.57,1.73)	0.84 (-0.58,2.25)
-1.25 (-3.00,0.50)	-0.86 (-2.29,0.57)	-0.14 (-1.49,1.20)	-0.11 (-1.73,1.51)	FJ	0.07 (-1.55,1.69)	0.10 (-1.17,1.38)	0.14 (-1.46,1.74)	0.26 (-1.32,1.84)	0.40 (-1.23,2.03)	0.38 (-1.01,1.78)	0.47 (-0.66,1.60)	0.73 (-0.68,2.13)
-1.32 (-3.09,0.44)	-0.93 (-2.38,0.51)	-0.21 (-1.58,1.15)	-0.18 (-1.81,1.45)	-0.07 (-1.69,1.55)	AE	0.03 (-1.25,1.32)	0.07 (-1.55,1.69)	0.19 (-1.40,1.78)	0.33 (-1.31,1.97)	0.31 (-1.09,1.72)	0.40 (-0.75,1.55)	0.66 (-0.76,2.07)
-1.36 (-2.81,0.10)	-0.97 (-2.01,0.08)	-0.25 (-1.18,0.68)	-0.21 (-1.50,1.07)	-0.10 (-1.38,1.17)	-0.03 (-1.32,1.25)	G	0.04 (-1.24,1.31)	0.16 (-1.08,1.40)	0.30 (-1.01,1.60)	0.28 (-0.71,1.27)	0.37 (-0.21,0.94)	0.62 (-0.39,1.63)
-1.39 (-3.15,0.36)	-1.00 (-2.43,0.43)	-0.28 (-1.63,1.06)	-0.25 (-1.87,1.37)	-0.14 (-1.74,1.46)	-0.07 (-1.69,1.55)	-0.04 (-1.31,1.24)	ACD	0.12 (-1.46,1.70)	0.26 (-1.37,1.89)	0.24 (-1.15,1.64)	0.33 (-0.81,1.47)	0.59 (-0.82,1.99)
-1.51 (-3.24,0.21)	-1.12 (-2.52,0.28)	-0.40 (-1.72,0.91)	-0.37 (-1.96,1.22)	-0.26 (-1.84,1.32)	-0.19 (-1.78,1.40)	-0.16 (-1.40,1.08)	-0.12 (-1.70,1.46)	AJ	0.14 (-1.47,1.75)	0.12 (-1.24,1.49)	0.21 (-0.89,1.31)	0.47 (-0.91,1.84)
-1.65 (-3.43,0.12)	-1.26 (-2.72,0.20)	-0.54 (-1.92,0.84)	-0.51 (-2.15,1.13)	-0.40 (-2.03,1.23)	-0.33 (-1.97,1.31)	-0.30 (-1.60,1.01)	-0.26 (-1.89,1.37)	-0.14 (-1.75,1.47)	GL	-0.02 (-1.44,1.41)	0.07 (-1.10,1.24)	0.33 (-1.11,1.76)
**-1.64 (-3.20,-0.08)**	**-1.25 (-2.44,-0.05)**	-0.53 (-1.62,0.56)	-0.49 (-1.90,0.91)	-0.38 (-1.78,1.01)	-0.31 (-1.72,1.09)	-0.28 (-1.27,0.71)	-0.24 (-1.64,1.15)	-0.12 (-1.49,1.24)	0.02 (-1.41,1.44)	AB	0.09 (-0.72,0.89)	0.34 (-0.82,1.50)
**-1.72 (-3.06,-0.39)**	**-1.33 (-2.20,-0.46)**	-0.61 (-1.34,0.11)	-0.58 (-1.73,0.57)	-0.47 (-1.60,0.66)	-0.40 (-1.55,0.75)	-0.37 (-0.94,0.21)	-0.33 (-1.47,0.81)	-0.21 (-1.31,0.89)	-0.07 (-1.24,1.10)	-0.09 (-0.89,0.72)	A	0.26 (-0.57,1.08)
**-1.98 (-3.55,-0.41)**	**-1.59 (-2.79,-0.38)**	-0.87 (-1.97,0.23)	-0.84 (-2.25,0.58)	-0.73 (-2.13,0.68)	-0.66 (-2.07,0.76)	-0.62 (-1.63,0.39)	-0.59 (-1.99,0.82)	-0.47 (-1.84,0.91)	-0.33 (-1.76,1.11)	-0.34 (-1.50,0.82)	-0.26 (-1.08,0.57)	AH

Bold and underlined values indicate statistically significant differences.

Table A14. League table for SL.

F	0.81<br>(-0.47,2.10)	0.71<br>(-1.38,2.79)	1.05<br>(-1.09,3.20)	1.14<br>(-0.59,2.88)	1.32<br>(-0.20,2.84)	1.40<br>(-0.68,3.49)	1.40<br>(-0.70,3.50)	1.53<br>(-0.53,3.60)	1.60<br>(-0.14,3.34)	1.70<br>(-0.41,3.82)	1.84<br>(-0.24,3.93)	**1.70<br>(0.43,2.98)**
-0.81<br>(-2.10,0.47)	K	-0.11<br>(-1.75,1.53)	0.24<br>(-1.77,2.25)	0.33<br>(-1.24,1.90)	0.51<br>(-0.83,1.84)	0.59<br>(-1.36,2.54)	0.59<br>(-1.38,2.56)	0.72<br>(-1.21,2.65)	0.79<br>(-0.80,2.37)	0.89<br>(-1.09,2.87)	1.03<br>(-0.93,2.99)	0.89<br>(-0.16,1.94)
-0.71<br>(-2.79,1.38)	0.11<br>(-1.53,1.75)	KL	0.35<br>(-2.25,2.95)	0.44<br>(-1.83,2.71)	0.62<br>(-1.50,2.73)	0.70<br>(-1.85,3.25)	0.70<br>(-1.86,3.26)	0.83<br>(-1.71,3.36)	0.89<br>(-1.38,3.17)	1.00<br>(-1.57,3.57)	1.14<br>(-1.41,3.69)	1.00<br>(-0.95,2.94)
-1.05<br>(-3.20,1.09)	-0.24<br>(-2.25,1.77)	-0.35<br>(-2.95,2.25)	AL	0.09<br>(-1.99,2.17)	0.27<br>(-1.64,2.18)	0.35<br>(-2.03,2.73)	0.35<br>(-2.04,2.74)	0.48<br>(-1.89,2.85)	0.55<br>(-1.54,2.63)	0.65<br>(-1.76,3.06)	0.79<br>(-1.60,3.18)	0.65<br>(-1.07,2.37)
-1.14<br>(-2.88,0.59)	-0.33<br>(-1.90,1.24)	-0.44<br>(-2.71,1.83)	-0.09<br>(-2.17,1.99)	AH	0.18<br>(-1.26,1.61)	0.26<br>(-1.76,2.28)	0.26<br>(-1.77,2.30)	0.39<br>(-1.61,2.40)	0.46<br>(-1.21,2.12)	0.56<br>(-1.49,2.61)	0.70<br>(-1.32,2.73)	0.56<br>(-0.61,1.73)
-1.32<br>(-2.84,0.20)	-0.51<br>(-1.84,0.83)	-0.62<br>(-2.73,1.50)	-0.27<br>(-2.18,1.64)	-0.18<br>(-1.61,1.26)	G	0.08<br>(-1.76,1.93)	0.08<br>(-1.78,1.94)	0.21<br>(-1.61,2.04)	0.28<br>(-1.17,1.73)	0.38<br>(-1.49,2.26)	0.52<br>(-1.33,2.37)	0.38<br>(-0.45,1.21)
-1.40<br>(-3.49,0.68)	-0.59<br>(-2.54,1.36)	-0.70<br>(-3.25,1.85)	-0.35<br>(-2.73,2.03)	-0.26<br>(-2.28,1.76)	-0.08<br>(-1.93,1.76)	ACD	0.00<br>(-2.34,2.34)	0.13<br>(-2.18,2.44)	0.20<br>(-1.83,2.22)	0.30<br>(-2.05,2.65)	0.44<br>(-1.89,2.77)	0.30<br>(-1.34,1.94)
-1.40<br>(-3.50,0.70)	-0.59<br>(-2.56,1.38)	-0.70<br>(-3.26,1.86)	-0.35<br>(-2.74,2.04)	-0.26<br>(-2.30,1.77)	-0.08<br>(-1.94,1.78)	-0.00<br>(-2.34,2.34)	FJ	0.13<br>(-2.20,2.46)	0.20<br>(-1.85,2.24)	0.30<br>(-2.07,2.67)	0.44<br>(-1.91,2.79)	0.30<br>(-1.37,1.97)
-1.53<br>(-3.60,0.53)	-0.72<br>(-2.65,1.21)	-0.83<br>(-3.36,1.71)	-0.48<br>(-2.85,1.89)	-0.39<br>(-2.40,1.61)	-0.21<br>(-2.04,1.61)	-0.13<br>(-2.44,2.18)	-0.13<br>(-2.46,2.20)	AJ	0.07<br>(-1.95,2.08)	0.17<br>(-2.17,2.51)	0.31<br>(-2.01,2.63)	0.17<br>(-1.45,1.79)
-1.60<br>(-3.34,0.14)	-0.79<br>(-2.37,0.80)	-0.89<br>(-3.17,1.38)	-0.55<br>(-2.63,1.54)	-0.46<br>(-2.12,1.21)	-0.28<br>(-1.73,1.17)	-0.20<br>(-2.22,1.83)	-0.20<br>(-2.24,1.85)	-0.07<br>(-2.08,1.95)	AB	0.10<br>(-1.96,2.17)	0.24<br>(-1.79,2.28)	0.10<br>(-1.08,1.29)
-1.70<br>(-3.82,0.41)	-0.89<br>(-2.87,1.09)	-1.00<br>(-3.57,1.57)	-0.65<br>(-3.06,1.76)	-0.56<br>(-2.61,1.49)	-0.38<br>(-2.26,1.49)	-0.30<br>(-2.65,2.05)	-0.30<br>(-2.67,2.07)	-0.17<br>(-2.51,2.17)	-0.10<br>(-2.17,1.96)	GL	0.14<br>(-2.22,2.50)	-0.00<br>(-1.68,1.68)
-1.84<br>(-3.93,0.24)	-1.03<br>(-2.99,0.93)	-1.14<br>(-3.69,1.41)	-0.79<br>(-3.18,1.60)	-0.70<br>(-2.73,1.32)	-0.52<br>(-2.37,1.33)	-0.44<br>(-2.77,1.89)	-0.44<br>(-2.79,1.91)	-0.31<br>(-2.63,2.01)	-0.24<br>(-2.28,1.79)	-0.14<br>(-2.50,2.22)	AE	-0.14<br>(-1.79,1.51)
**-1.70<br>(-2.98,-0.43)**	-0.89<br>(-1.94,0.16)	-1.00<br>(-2.94,0.95)	-0.65<br>(-2.37,1.07)	-0.56<br>(-1.73,0.61)	-0.38<br>(-1.21,0.45)	-0.30<br>(-1.94,1.34)	-0.30<br>(-1.97,1.37)	-0.17<br>(-1.79,1.45)	-0.10<br>(-1.29,1.08)	0.00<br>(-1.68,1.68)	0.14<br>(-1.51,1.79)	A

Bold and underlined values indicate statistically significant differences.

Table A15. League table for SD.

KL	0.09 (-0.90,1.08)	0.45 (-1.11,2.02)	0.26 (-1.03,1.55)	0.13 (-1.43,1.69)	-0.65 (-1.92,0.62)	0.24 (-1.33,1.82)	0.33 (-1.20,1.87)	0.39 (-1.00,1.78)	0.06 (-1.54,1.66)	0.25 (-1.30,1.81)	0.38 (-1.02,1.77)	0.56 (-0.62,1.75)
-0.09 (-1.08,0.90)	K	0.36 (-0.85,1.57)	0.17 (-0.66,1.00)	0.04 (-1.16,1.25)	-0.74 (-1.54,0.06)	0.15 (-1.07,1.38)	0.24 (-0.93,1.42)	0.30 (-0.67,1.28)	-0.03 (-1.28,1.23)	0.16 (-1.04,1.36)	0.29 (-0.69,1.27)	0.47 (-0.18,1.12)
-0.45 (-2.02,1.11)	-0.36 (-1.57,0.85)	GL	-0.19 (-1.34,0.95)	-0.32 (-1.76,1.12)	-1.10 (-2.40,0.19)	-0.21 (-1.67,1.25)	-0.12 (-1.53,1.29)	-0.06 (-1.31,1.19)	-0.39 (-1.87,1.09)	-0.20 (-1.63,1.23)	-0.08 (-1.33,1.18)	0.11 (-0.91,1.13)
-0.26 (-1.55,1.03)	-0.17 (-1.00,0.66)	0.19 (-0.95,1.34)	G	-0.13 (-1.27,1.01)	-0.91 (-1.86,0.04)	-0.02 (-1.18,1.15)	0.07 (-1.04,1.18)	0.13 (-0.76,1.03)	-0.20 (-1.39,0.99)	-0.01 (-1.14,1.13)	0.12 (-0.78,1.02)	0.30 (-0.22,0.82)
-0.13 (-1.69,1.43)	-0.04 (-1.25,1.16)	0.32 (-1.12,1.76)	0.13 (-1.01,1.27)	FJ	-0.78 (-2.08,0.51)	0.11 (-1.35,1.57)	0.20 (-1.21,1.61)	0.26 (-0.99,1.51)	-0.07 (-1.55,1.41)	0.12 (-1.31,1.55)	0.24 (-1.01,1.50)	0.43 (-0.59,1.45)
0.65 (-0.62,1.92)	0.74 (-0.06,1.54)	1.10 (-0.19,2.40)	0.91 (-0.04,1.86)	0.78 (-0.51,2.08)	F	0.89 (-0.42,2.21)	0.98 (-0.28,2.24)	1.04 (-0.04,2.12)	0.71 (-0.62,2.05)	0.90 (-0.38,2.19)	1.03 (-0.06,2.11)	**1.21 (0.41,2.01)**
-0.24 (-1.82,1.33)	-0.15 (-1.38,1.07)	0.21 (-1.25,1.67)	0.02 (-1.15,1.18)	-0.11 (-1.57,1.35)	-0.89 (-2.21,0.42)	AL	0.09 (-1.34,1.52)	0.15 (-1.12,1.42)	-0.18 (-1.68,1.32)	0.01 (-1.44,1.46)	0.13 (-1.14,1.41)	0.32 (-0.72,1.36)
-0.33 (-1.87,1.20)	-0.24 (-1.42,0.93)	0.12 (-1.29,1.53)	-0.07 (-1.18,1.04)	-0.20 (-1.61,1.21)	-0.98 (-2.24,0.28)	-0.09 (-1.52,1.34)	AJ	0.06 (-1.16,1.28)	-0.27 (-1.72,1.18)	-0.08 (-1.49,1.33)	0.04 (-1.18,1.27)	0.23 (-0.75,1.21)
-0.39 (-1.78,1.00)	-0.30 (-1.28,0.67)	0.06 (-1.19,1.31)	-0.13 (-1.03,0.76)	-0.26 (-1.51,0.99)	-1.04 (-2.12,0.04)	-0.15 (-1.42,1.12)	-0.06 (-1.28,1.16)	AH	-0.33 (-1.63,0.97)	-0.14 (-1.38,1.11)	-0.02 (-1.05,1.02)	0.17 (-0.56,0.90)
-0.06 (-1.66,1.54)	0.03 (-1.23,1.28)	0.39 (-1.09,1.87)	0.20 (-0.99,1.39)	0.07 (-1.41,1.55)	-0.71 (-2.05,0.62)	0.18 (-1.32,1.68)	0.27 (-1.18,1.72)	0.33 (-0.97,1.63)	AE	0.19 (-1.28,1.66)	0.31 (-0.99,1.61)	0.50 (-0.57,1.57)
-0.25 (-1.81,1.30)	-0.16 (-1.36,1.04)	0.20 (-1.23,1.63)	0.01 (-1.13,1.14)	-0.12 (-1.55,1.31)	-0.90 (-2.19,0.38)	-0.01 (-1.46,1.44)	0.08 (-1.33,1.49)	0.14 (-1.11,1.38)	-0.19 (-1.66,1.28)	ACD	0.12 (-1.12,1.37)	0.31 (-0.70,1.32)
-0.38 (-1.77,1.02)	-0.29 (-1.27,0.69)	0.08 (-1.18,1.33)	-0.12 (-1.02,0.78)	-0.24 (-1.50,1.01)	-1.03 (-2.11,0.06)	-0.13 (-1.41,1.14)	-0.04 (-1.27,1.18)	0.02 (-1.02,1.05)	-0.31 (-1.61,0.99)	-0.12 (-1.37,1.12)	AB	0.19 (-0.55,0.92)
-0.56 (-1.75,0.62)	-0.47 (-1.12,0.18)	-0.11 (-1.13,0.91)	-0.30 (-0.82,0.22)	-0.43 (-1.45,0.59)	**-1.21 (-2.01,-0.41)**	-0.32 (-1.36,0.72)	-0.23 (-1.21,0.75)	-0.17 (-0.90,0.56)	-0.50 (-1.57,0.57)	-0.31 (-1.32,0.70)	-0.19 (-0.92,0.55)	A

Bold and underlined values indicate statistically significant differences.

Table A16. League table for SE.

AH	0.07 (-0.81,0.95)	0.08 (-0.73,0.89)	0.15 (-0.60,0.90)	0.16 (-0.69,1.01)	0.20 (-0.57,0.97)	0.22 (-0.38,0.82)	0.25 (-0.38,0.87)	0.28 (-0.49,1.04)	0.36 (-0.35,1.06)	0.50 (-0.06,1.06)	0.58 (-0.07,1.23)	**0.55 (0.10,0.99)**
-0.07 (-0.95,0.81)	KL	0.01 (-1.01,1.02)	0.08 (-0.89,1.05)	0.09 (-0.96,1.14)	0.13 (-0.86,1.11)	0.15 (-0.49,0.79)	0.18 (-0.70,1.05)	0.21 (-0.78,1.19)	0.29 (-0.65,1.22)	0.43 (-0.40,1.26)	0.51 (-0.30,1.31)	0.48 (-0.28,1.24)
-0.08 (-0.89,0.73)	-0.01 (-1.02,1.01)	AE	0.07 (-0.84,0.98)	0.08 (-0.91,1.07)	0.12 (-0.80,1.04)	0.14 (-0.65,0.94)	0.17 (-0.64,0.98)	0.20 (-0.72,1.12)	0.28 (-0.59,1.15)	0.42 (-0.34,1.18)	0.50 (-0.33,1.33)	0.47 (-0.21,1.15)
-0.15 (-0.90,0.60)	-0.08 (-1.05,0.89)	-0.07 (-0.98,0.84)	FJ	0.01 (-0.93,0.95)	0.05 (-0.82,0.92)	0.07 (-0.66,0.80)	0.10 (-0.65,0.85)	0.13 (-0.74,1.00)	0.21 (-0.61,1.03)	0.35 (-0.34,1.05)	0.43 (-0.34,1.20)	0.40 (-0.20,1.00)
-0.16 (-1.01,0.69)	-0.09 (-1.14,0.96)	-0.08 (-1.07,0.91)	-0.01 (-0.95,0.93)	AL	0.04 (-0.92,1.00)	0.06 (-0.77,0.90)	0.09 (-0.76,0.94)	0.12 (-0.84,1.08)	0.20 (-0.71,1.11)	0.34 (-0.46,1.15)	0.42 (-0.45,1.29)	0.39 (-0.33,1.11)
-0.20 (-0.97,0.57)	-0.13 (-1.11,0.86)	-0.12 (-1.04,0.80)	-0.05 (-0.92,0.82)	-0.04 (-1.00,0.92)	ACD	0.02 (-0.73,0.77)	0.05 (-0.72,0.82)	0.08 (-0.81,0.97)	0.16 (-0.67,0.99)	0.30 (-0.41,1.02)	0.38 (-0.41,1.17)	0.35 (-0.28,0.98)
-0.22 (-0.82,0.38)	-0.15 (-0.79,0.49)	-0.14 (-0.94,0.65)	-0.07 (-0.80,0.66)	-0.06 (-0.90,0.77)	-0.02 (-0.77,0.73)	K	0.03 (-0.58,0.63)	0.06 (-0.69,0.80)	0.14 (-0.55,0.82)	0.28 (-0.25,0.81)	0.36 (-0.13,0.85)	0.33 (-0.08,0.74)
-0.25 (-0.87,0.38)	-0.18 (-1.05,0.70)	-0.17 (-0.98,0.64)	-0.10 (-0.85,0.65)	-0.09 (-0.94,0.76)	-0.05 (-0.82,0.72)	-0.03 (-0.63,0.58)	AB	0.03 (-0.73,0.79)	0.11 (-0.59,0.81)	0.25 (-0.31,0.81)	0.33 (-0.32,0.98)	0.30 (-0.14,0.74)
-0.28 (-1.04,0.49)	-0.21 (-1.19,0.78)	-0.20 (-1.12,0.72)	-0.13 (-1.00,0.74)	-0.12 (-1.08,0.84)	-0.08 (-0.97,0.81)	-0.06 (-0.80,0.69)	-0.03 (-0.79,0.73)	GL	0.08 (-0.75,0.91)	0.22 (-0.49,0.94)	0.30 (-0.48,1.09)	0.27 (-0.35,0.89)
-0.36 (-1.06,0.35)	-0.29 (-1.22,0.65)	-0.28 (-1.15,0.59)	-0.21 (-1.03,0.61)	-0.20 (-1.11,0.71)	-0.16 (-0.99,0.67)	-0.14 (-0.82,0.55)	-0.11 (-0.81,0.59)	-0.08 (-0.91,0.75)	AJ	0.14 (-0.50,0.79)	0.22 (-0.50,0.95)	0.19 (-0.36,0.74)
-0.50 (-1.06,0.06)	-0.43 (-1.26,0.40)	-0.42 (-1.18,0.34)	-0.35 (-1.05,0.34)	-0.34 (-1.15,0.46)	-0.30 (-1.02,0.41)	-0.28 (-0.81,0.25)	-0.25 (-0.81,0.31)	-0.22 (-0.94,0.49)	-0.14 (-0.79,0.50)	G	0.08 (-0.51,0.67)	0.05 (-0.30,0.39)
-0.58 (-1.23,0.07)	-0.51 (-1.31,0.30)	-0.50 (-1.33,0.33)	-0.43 (-1.20,0.34)	-0.42 (-1.29,0.45)	-0.38 (-1.17,0.41)	-0.36 (-0.85,0.13)	-0.33 (-0.98,0.32)	-0.30 (-1.09,0.48)	-0.22 (-0.95,0.50)	-0.08 (-0.67,0.51)	F	-0.03 (-0.51,0.45)
**-0.55 (-0.99,-0.10)**	-0.48 (-1.24,0.28)	-0.47 (-1.15,0.21)	-0.40 (-1.00,0.20)	-0.39 (-1.11,0.33)	-0.35 (-0.98,0.28)	-0.33 (-0.74,0.08)	-0.30 (-0.74,0.14)	-0.27 (-0.89,0.35)	-0.19 (-0.74,0.36)	-0.05 (-0.39,0.30)	0.03 (-0.45,0.51)	A

Bold and underlined values indicate statistically significant differences.

Table A17. League table for SDI.

KL	0.09 (-0.64,0.81)	0.18 (-0.62,0.99)	0.33 (-0.58,1.24)	0.37 (-0.57,1.31)	0.46 (-0.43,1.35)	0.46 (-0.40,1.32)	0.46 (-0.14,1.06)	0.48 (-0.43,1.39)	0.68 (-0.12,1.47)	**0.77 (0.02,1.53)**	**0.80 (0.09,1.51)**
-0.09 (-0.81,0.64)	F	0.10 (-0.47,0.67)	0.24 (-0.46,0.95)	0.28 (-0.47,1.03)	0.37 (-0.32,1.07)	0.37 (-0.27,1.02)	0.37 (-0.03,0.78)	0.39 (-0.31,1.10)	**0.59 (0.03,1.15)**	**0.69 (0.19,1.18)**	**0.71 (0.29,1.13)**
-0.18 (-0.99,0.62)	-0.10 (-0.67,0.47)	AH	0.15 (-0.54,0.84)	0.19 (-0.54,0.92)	0.28 (-0.39,0.95)	0.28 (-0.35,0.90)	0.28 (-0.25,0.81)	0.30 (-0.39,0.99)	0.49 (-0.04,1.03)	**0.59 (0.12,1.06)**	**0.62 (0.23,1.00)**
-0.33 (-1.24,0.58)	-0.24 (-0.95,0.46)	-0.15 (-0.84,0.54)	FJ	0.04 (-0.80,0.88)	0.13 (-0.66,0.92)	0.13 (-0.62,0.88)	0.13 (-0.55,0.81)	0.15 (-0.66,0.96)	0.35 (-0.33,1.03)	0.44 (-0.19,1.07)	0.47 (-0.10,1.04)
-0.37 (-1.31,0.57)	-0.28 (-1.03,0.47)	-0.19 (-0.92,0.54)	-0.04 (-0.88,0.80)	AE	0.09 (-0.74,0.92)	0.09 (-0.70,0.88)	0.09 (-0.63,0.81)	0.11 (-0.73,0.95)	0.31 (-0.42,1.03)	0.40 (-0.28,1.08)	0.43 (-0.19,1.05)
-0.46 (-1.35,0.43)	-0.37 (-1.07,0.32)	-0.28 (-0.95,0.39)	-0.13 (-0.92,0.66)	-0.09 (-0.92,0.74)	AL	-0.00 (-0.74,0.74)	0.00 (-0.66,0.66)	0.02 (-0.77,0.81)	0.22 (-0.45,0.88)	0.31 (-0.30,0.92)	0.34 (-0.21,0.89)
-0.46 (-1.32,0.40)	-0.37 (-1.02,0.27)	-0.28 (-0.90,0.35)	-0.13 (-0.88,0.62)	-0.09 (-0.88,0.70)	0.00 (-0.74,0.74)	AJ	0.00 (-0.61,0.61)	0.02 (-0.73,0.77)	0.22 (-0.40,0.83)	0.31 (-0.25,0.87)	0.34 (-0.15,0.83)
-0.46 (-1.06,0.14)	-0.37 (-0.78,0.03)	-0.28 (-0.81,0.25)	-0.13 (-0.81,0.55)	-0.09 (-0.81,0.63)	-0.00 (-0.66,0.66)	-0.00 (-0.61,0.61)	K	0.02 (-0.66,0.70)	0.22 (-0.30,0.74)	0.31 (-0.14,0.77)	0.34 (-0.03,0.71)
-0.48 (-1.39,0.43)	-0.39 (-1.10,0.31)	-0.30 (-0.99,0.39)	-0.15 (-0.96,0.66)	-0.11 (-0.95,0.73)	-0.02 (-0.81,0.77)	-0.02 (-0.77,0.73)	-0.02 (-0.70,0.66)	ACD	0.20 (-0.48,0.88)	0.29 (-0.34,0.92)	0.32 (-0.25,0.89)
-0.68 (-1.47,0.12)	**-0.59 (-1.15,-0.03)**	-0.49 (-1.03,0.04)	-0.35 (-1.03,0.33)	-0.31 (-1.03,0.42)	-0.22 (-0.88,0.45)	-0.22 (-0.83,0.40)	-0.22 (-0.74,0.30)	-0.20 (-0.88,0.48)	AB	0.09 (-0.36,0.55)	0.12 (-0.25,0.49)
**-0.77 (-1.53,-0.02)**	**-0.69 (-1.18,-0.19)**	**-0.59 (-1.06,-0.12)**	-0.44 (-1.07,0.19)	-0.40 (-1.08,0.28)	-0.31 (-0.92,0.30)	-0.31 (-0.87,0.25)	-0.31 (-0.77,0.14)	-0.29 (-0.92,0.34)	-0.09 (-0.55,0.36)	G	0.03 (-0.24,0.30)
**-0.80 (-1.51,-0.09)**	**-0.71 (-1.13,-0.29)**	**-0.62 (-1.00,-0.23)**	-0.47 (-1.04,0.10)	-0.43 (-1.05,0.19)	-0.34 (-0.89,0.21)	-0.34 (-0.83,0.15)	-0.34 (-0.71,0.03)	-0.32 (-0.89,0.25)	-0.12 (-0.49,0.25)	-0.03 (-0.30,0.24)	A

Bold and underlined values indicate statistically significant differences.

Table A18. League table for DD.

KL	0.21<br>(-0.92,1.33)	0.20<br>(-0.83,1.22)	0.36<br>(-0.46,1.18)	0.44<br>(-0.84,1.72)	0.48<br>(-0.80,1.75)	0.49<br>(-0.78,1.76)	0.52<br>(-0.53,1.58)	0.60<br>(-0.63,1.83)	0.63<br>(-0.51,1.76)	0.92<br>(-0.34,2.18)	0.84<br>(-0.14,1.82)
-0.21<br>(-1.33,0.92)	AH	-0.01<br>(-0.84,0.82)	0.15<br>(-0.62,0.93)	0.23<br>(-0.76,1.23)	0.27<br>(-0.71,1.26)	0.28<br>(-0.69,1.26)	0.32<br>(-0.36,1.00)	0.39<br>(-0.54,1.33)	0.42<br>(-0.38,1.22)	0.71<br>(-0.26,1.68)	**0.63**<br>**(0.07,1.19)**
-0.20<br>(-1.22,0.83)	0.01<br>(-0.82,0.84)	F	0.16<br>(-0.46,0.78)	0.24<br>(-0.78,1.27)	0.28<br>(-0.74,1.30)	0.29<br>(-0.72,1.30)	0.33<br>(-0.40,1.06)	0.40<br>(-0.57,1.37)	0.43<br>(-0.41,1.27)	0.72<br>(-0.28,1.73)	**0.64**<br>**(0.03,1.26)**
-0.36<br>(-1.18,0.46)	-0.15<br>(-0.93,0.62)	-0.16<br>(-0.78,0.46)	K	0.08<br>(-0.91,1.07)	0.12<br>(-0.86,1.10)	0.13<br>(-0.84,1.10)	0.16<br>(-0.51,0.83)	0.24<br>(-0.68,1.16)	0.27<br>(-0.52,1.05)	0.56<br>(-0.40,1.52)	0.48<br>(-0.06,1.02)
-0.44<br>(-1.72,0.84)	-0.23<br>(-1.23,0.76)	-0.24<br>(-1.27,0.78)	-0.08<br>(-1.07,0.91)	AE	0.04<br>(-1.12,1.20)	0.05<br>(-1.10,1.20)	0.09<br>(-0.83,1.00)	0.16<br>(-0.95,1.27)	0.19<br>(-0.82,1.19)	0.48<br>(-0.66,1.62)	0.40<br>(-0.42,1.22)
-0.48<br>(-1.75,0.80)	-0.27<br>(-1.26,0.71)	-0.28<br>(-1.30,0.74)	-0.12<br>(-1.10,0.86)	-0.04<br>(-1.20,1.12)	FJ	0.01<br>(-1.13,1.15)	0.05<br>(-0.86,0.95)	0.12<br>(-0.99,1.23)	0.15<br>(-0.85,1.14)	0.44<br>(-0.70,1.58)	0.36<br>(-0.45,1.17)
-0.49<br>(-1.76,0.78)	-0.28<br>(-1.26,0.69)	-0.29<br>(-1.30,0.72)	-0.13<br>(-1.10,0.84)	-0.05<br>(-1.20,1.10)	-0.01<br>(-1.15,1.13)	AL	0.04<br>(-0.86,0.93)	0.11<br>(-0.99,1.21)	0.14<br>(-0.85,1.12)	0.43<br>(-0.70,1.56)	0.35<br>(-0.45,1.15)
-0.52<br>(-1.58,0.53)	-0.32<br>(-1.00,0.36)	-0.33<br>(-1.06,0.40)	-0.16<br>(-0.83,0.51)	-0.09<br>(-1.00,0.83)	-0.05<br>(-0.95,0.86)	-0.04<br>(-0.93,0.86)	G	0.07<br>(-0.77,0.92)	0.10<br>(-0.59,0.80)	0.39<br>(-0.49,1.28)	0.31<br>(-0.08,0.71)
-0.60<br>(-1.83,0.63)	-0.39<br>(-1.33,0.54)	-0.40<br>(-1.37,0.57)	-0.24<br>(-1.16,0.68)	-0.16<br>(-1.27,0.95)	-0.12<br>(-1.23,0.99)	-0.11<br>(-1.21,0.99)	-0.07<br>(-0.92,0.77)	AJ	0.03<br>(-0.91,0.97)	0.32<br>(-0.77,1.41)	0.24<br>(-0.51,0.99)
-0.63<br>(-1.76,0.51)	-0.42<br>(-1.22,0.38)	-0.43<br>(-1.27,0.41)	-0.27<br>(-1.05,0.52)	-0.19<br>(-1.19,0.82)	-0.15<br>(-1.14,0.85)	-0.14<br>(-1.12,0.85)	-0.10<br>(-0.80,0.59)	-0.03<br>(-0.97,0.91)	AB	0.29<br>(-0.69,1.27)	0.21<br>(-0.36,0.79)
-0.92<br>(-2.18,0.34)	-0.71<br>(-1.68,0.26)	-0.72<br>(-1.73,0.28)	-0.56<br>(-1.52,0.40)	-0.48<br>(-1.62,0.66)	-0.44<br>(-1.58,0.70)	-0.43<br>(-1.56,0.70)	-0.39<br>(-1.28,0.49)	-0.32<br>(-1.41,0.77)	-0.29<br>(-1.27,0.69)	ACD	-0.08<br>(-0.88,0.72)
-0.84<br>(-1.82,0.14)	**-0.63**<br>**(-1.19,-0.07)**	**-0.64**<br>**(-1.26,-0.03)**	-0.48<br>(-1.02,0.06)	-0.40<br>(-1.22,0.42)	-0.36<br>(-1.17,0.45)	-0.35<br>(-1.15,0.45)	-0.31<br>(-0.71,0.08)	-0.24<br>(-0.99,0.51)	-0.21<br>(-0.79,0.36)	0.08<br>(-0.72,0.88)	A

Bold and underlined values indicate statistically significant differences.

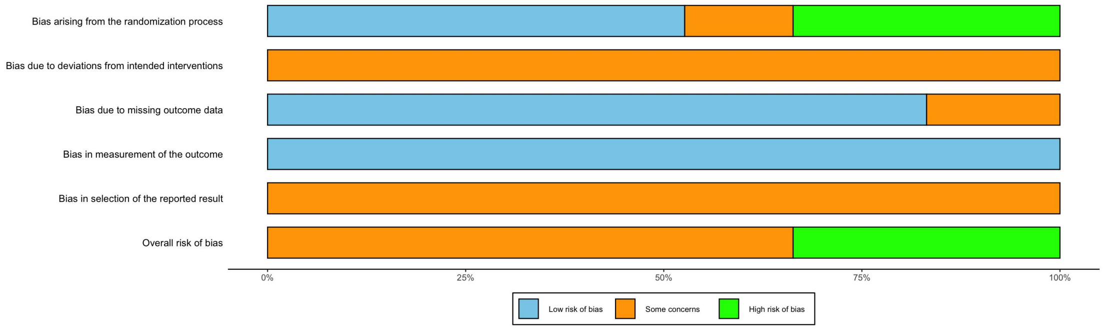

Fig. A1. The risk of bias charts for the included studies.

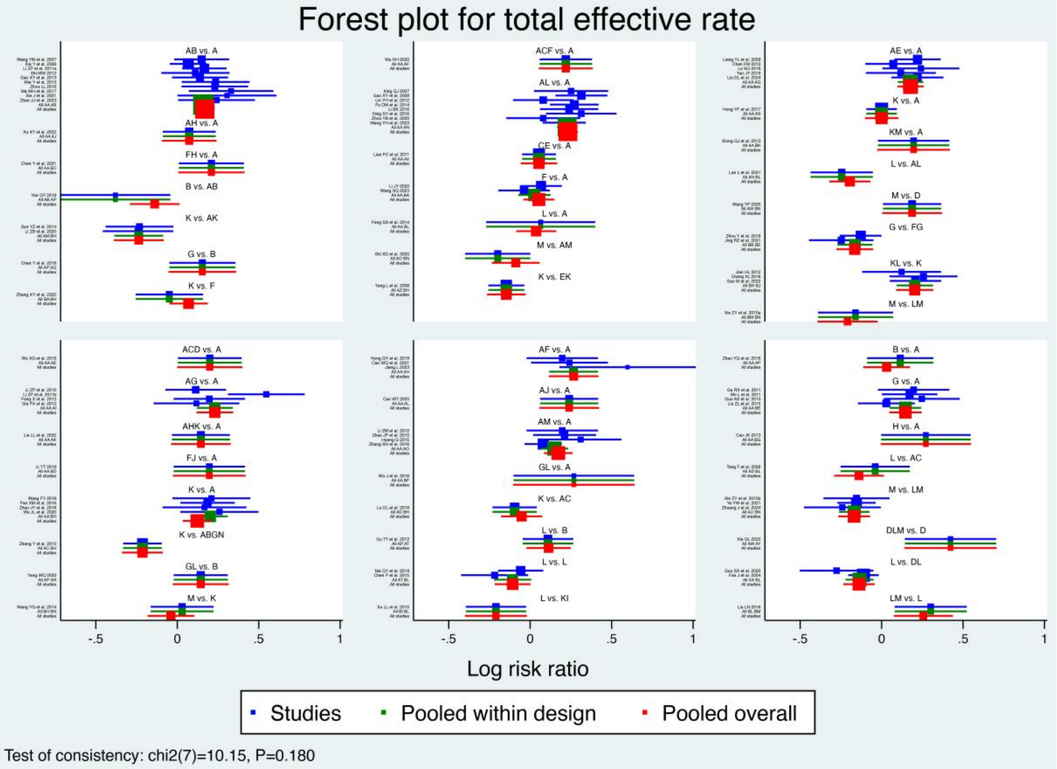

Fig. A2. Forest plot for total effective rate.

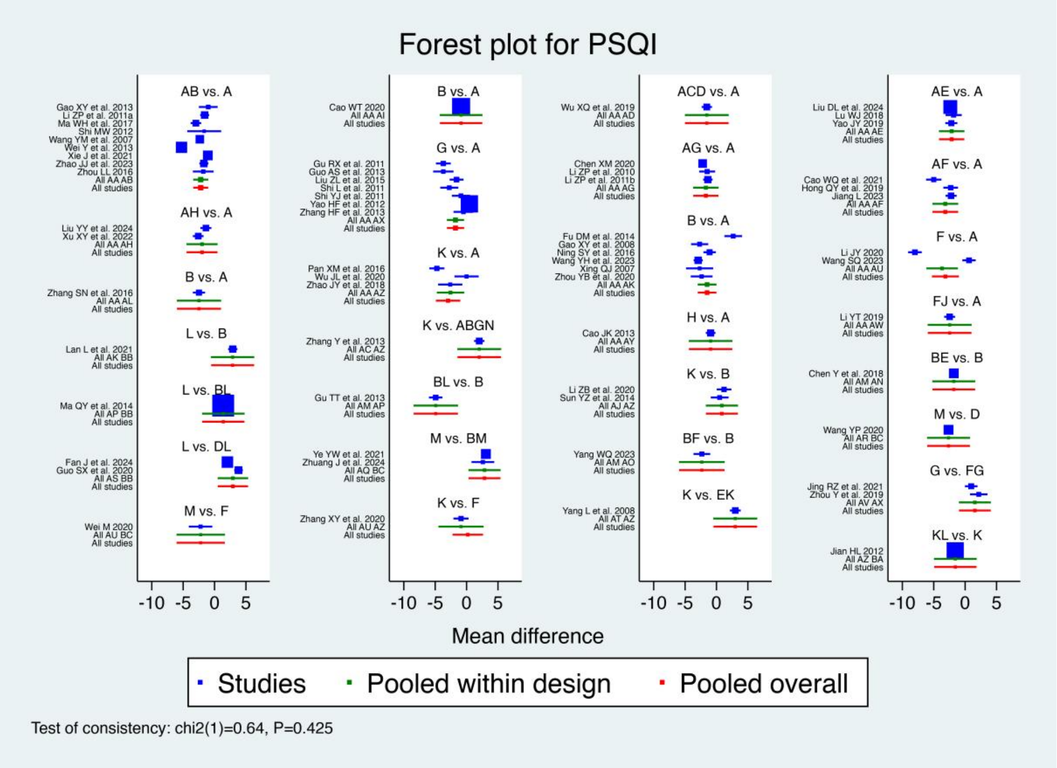

Fig. A3. Forest plot for PSQI.

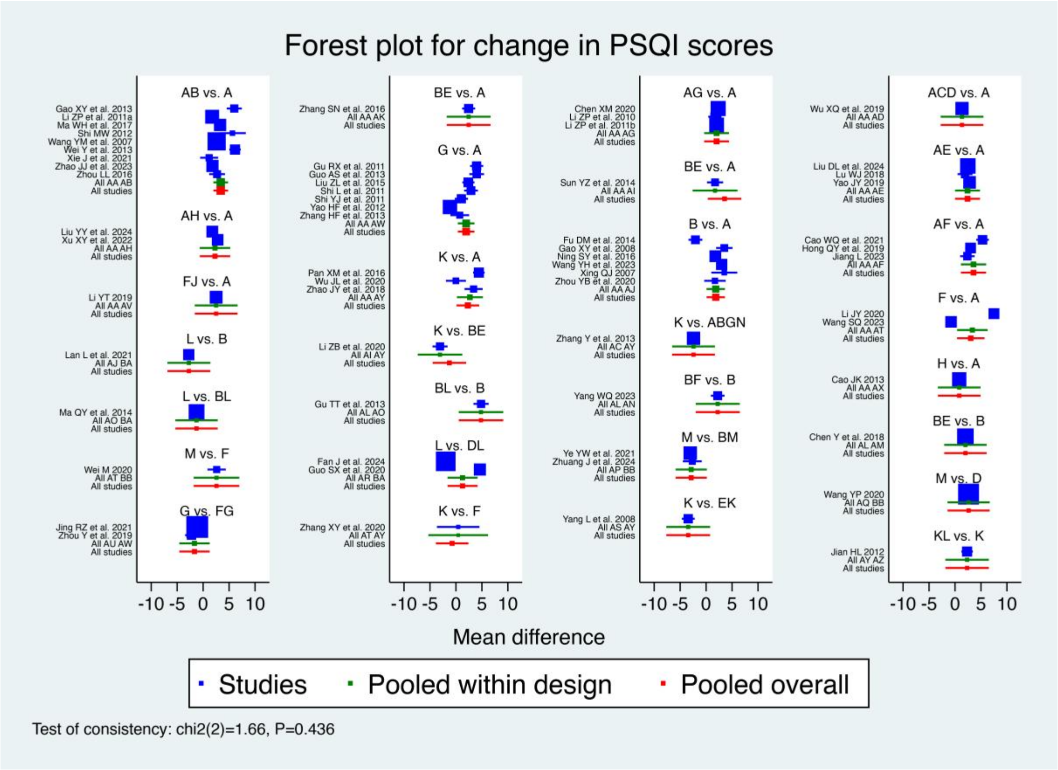

Fig. A4. Forest plot for change in PSQI scores.

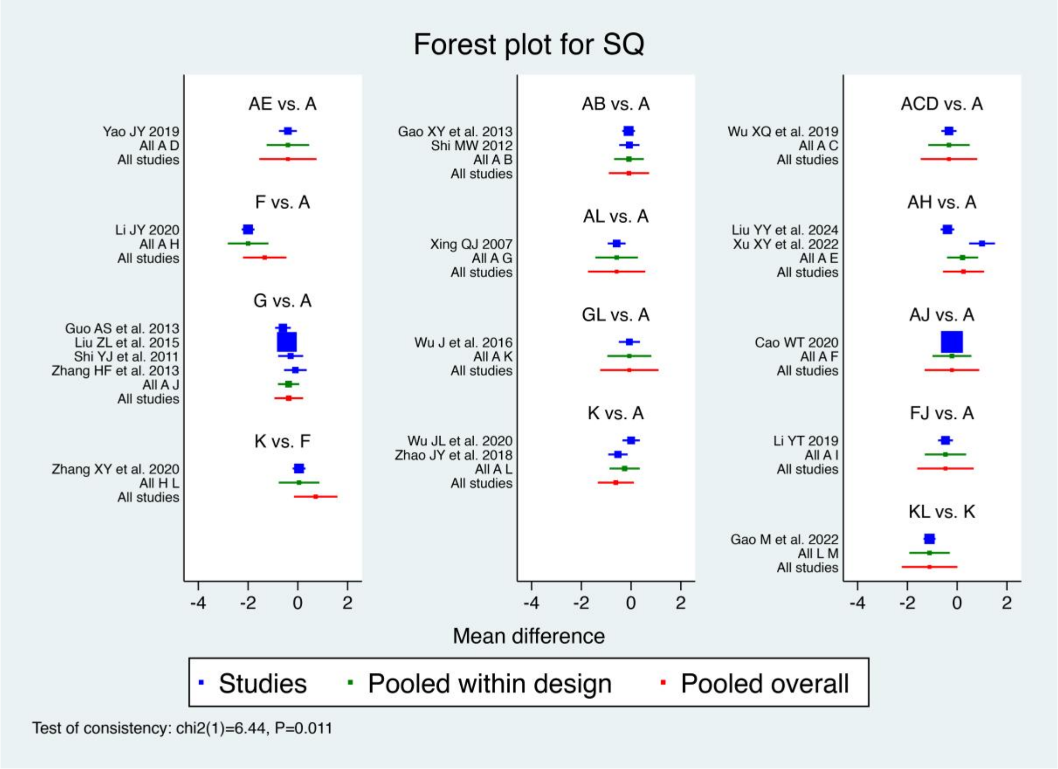

Fig. A5. Forest plot for SQ.

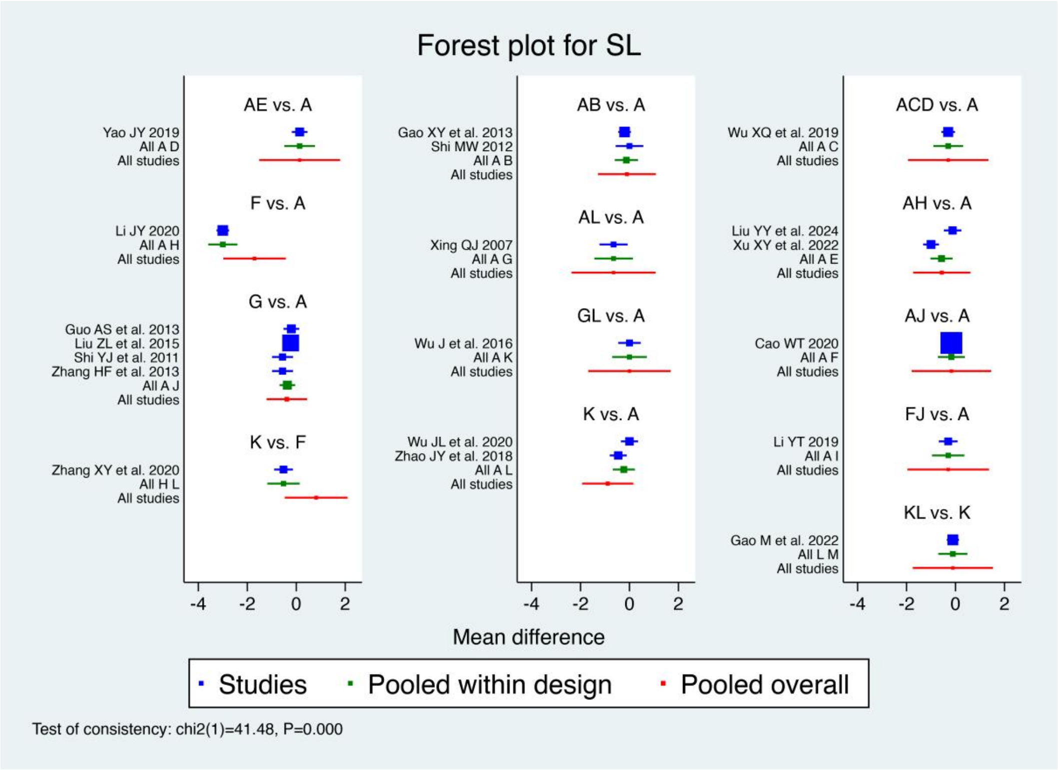

Fig. A6. Forest plot for SL.

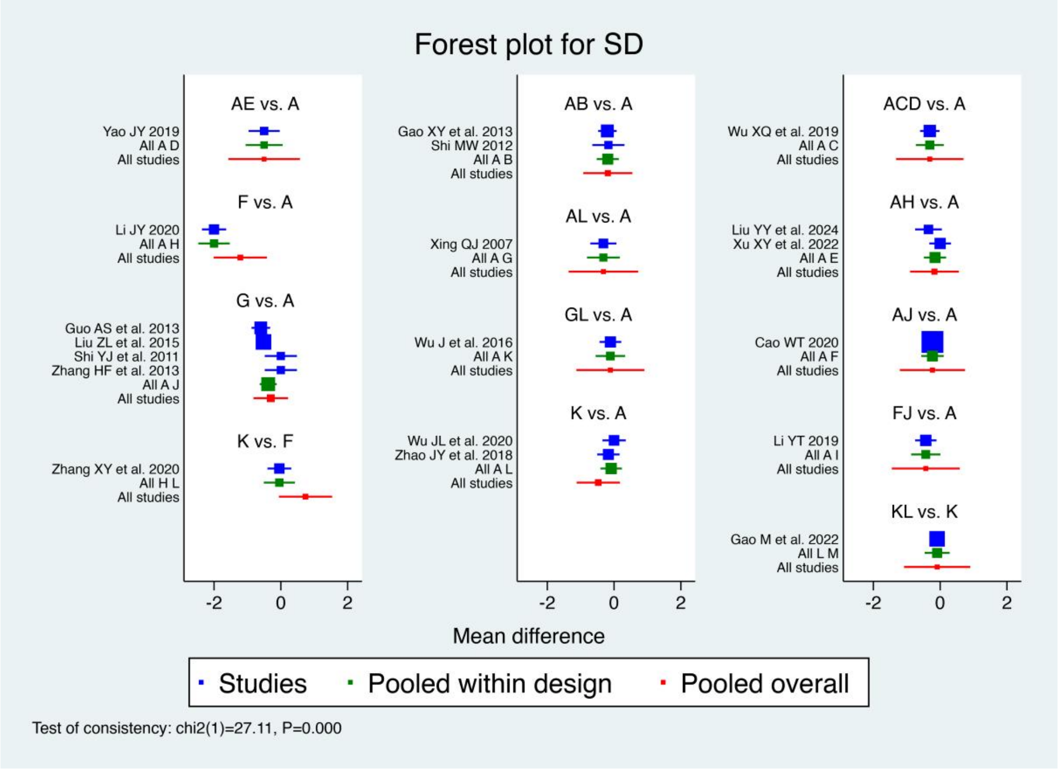

Fig. A7. Forest plot for SD.

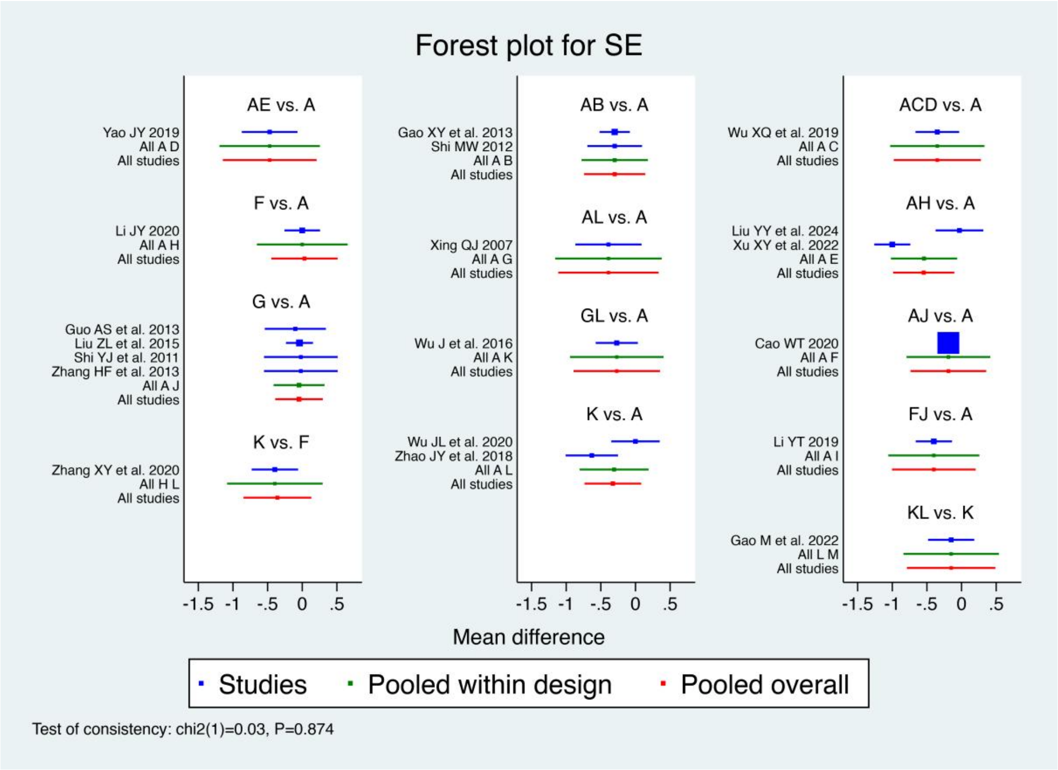

Fig. A8. Forest plot for SE.

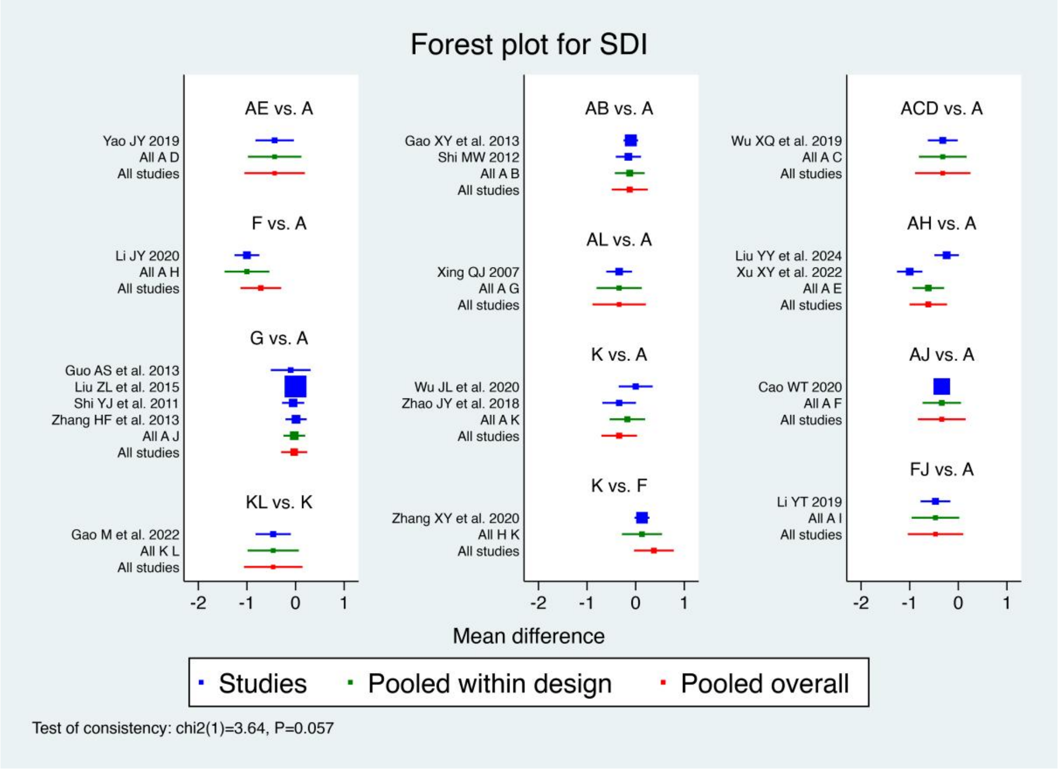

Fig. A9. Forest plot for SDI.

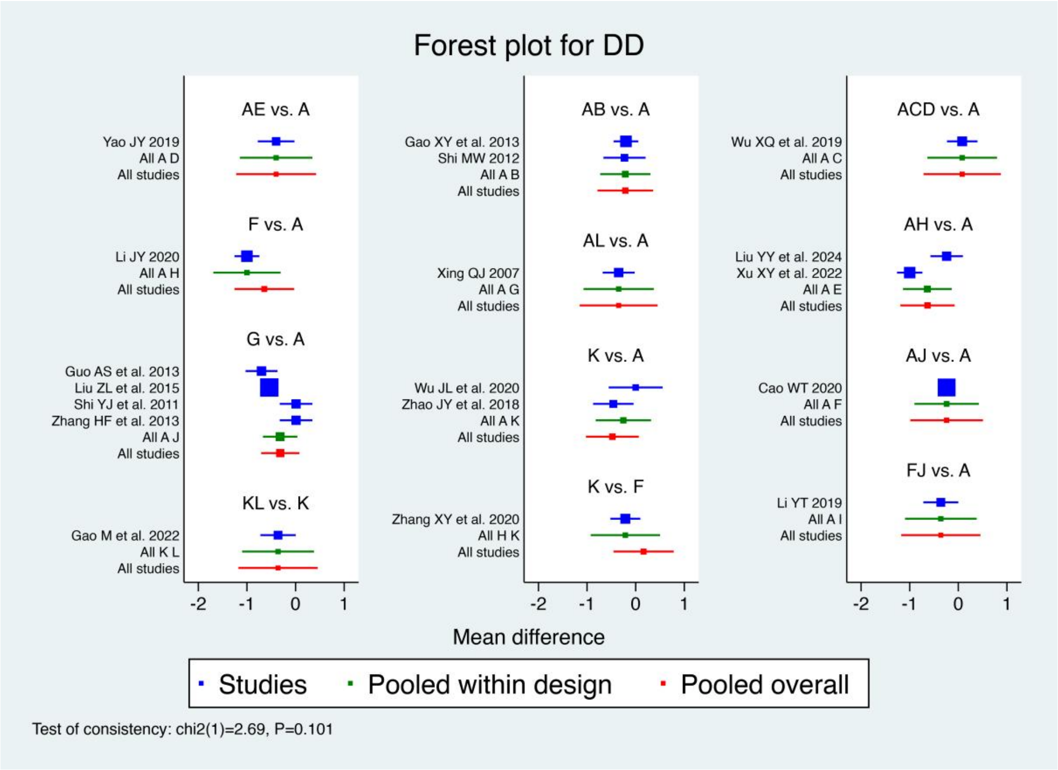

Fig. A10. Forest plot for DD.

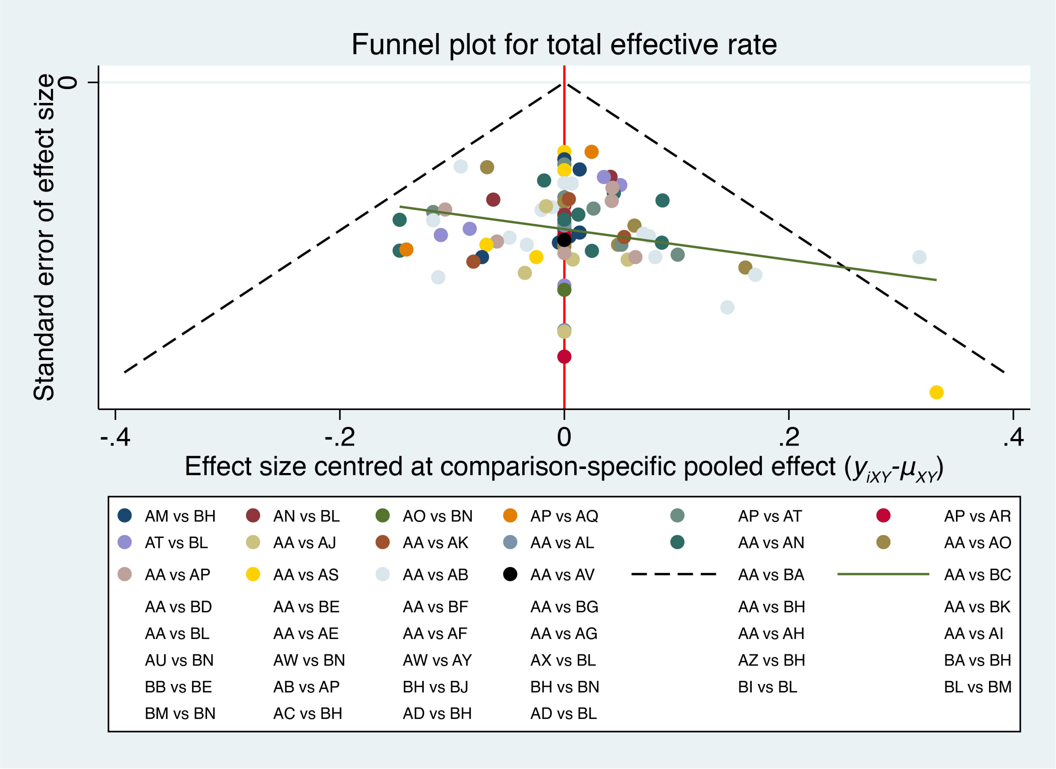

Fig. A11. Funnel plot for total effective rate.

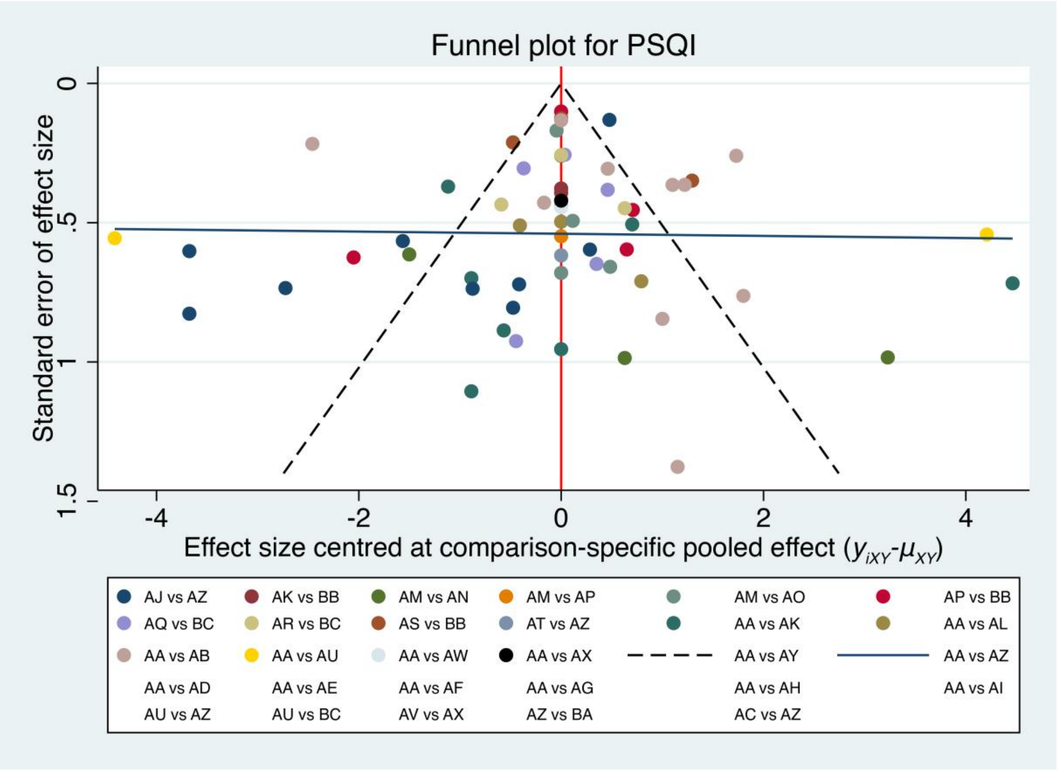

Fig. A12. Funnel plot for PSQI.

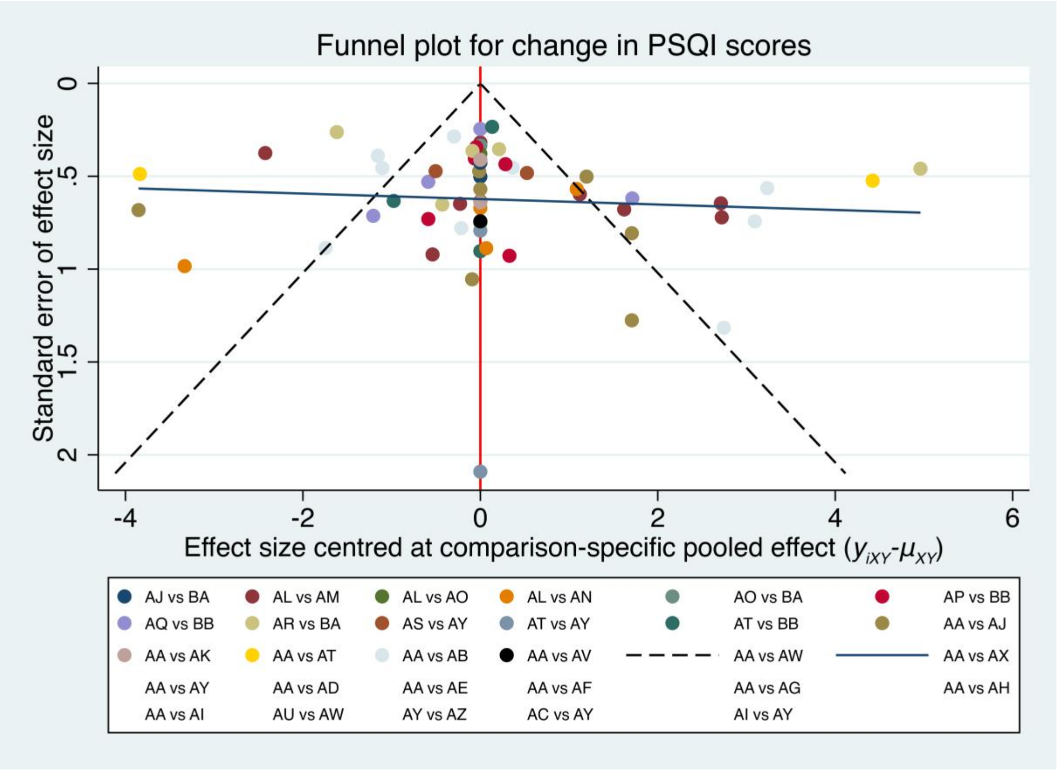

Fig. A13. Funnel plot for change in PSQI scores.

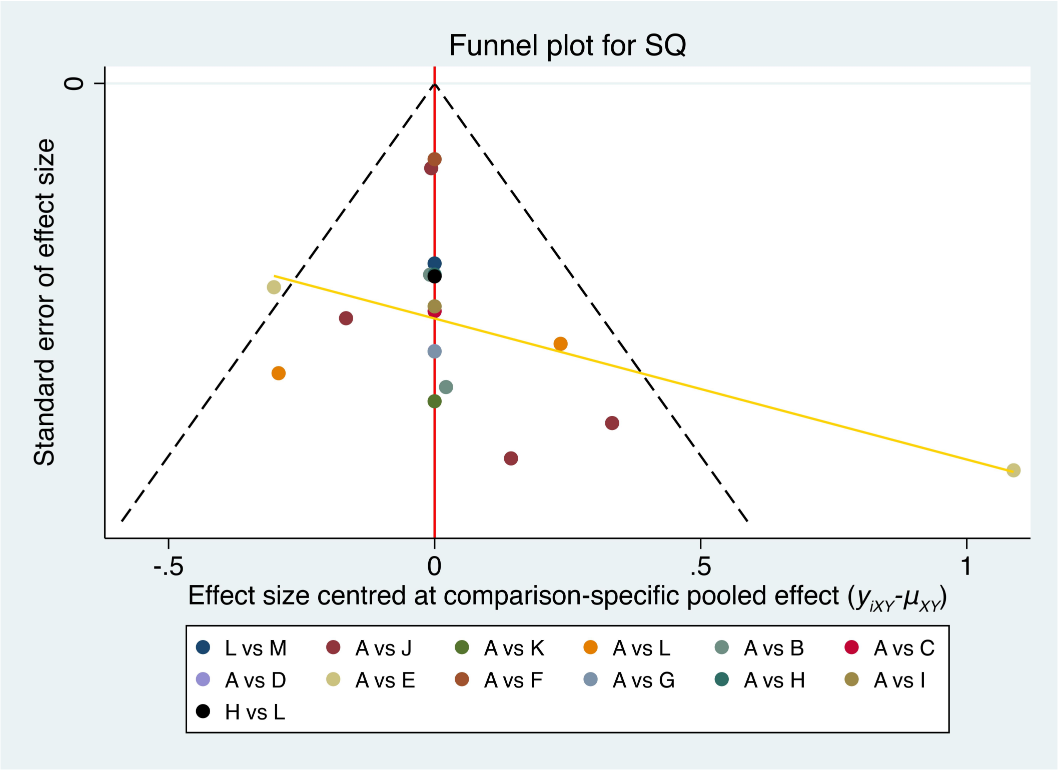

Fig. A14. Funnel plot for SQ.

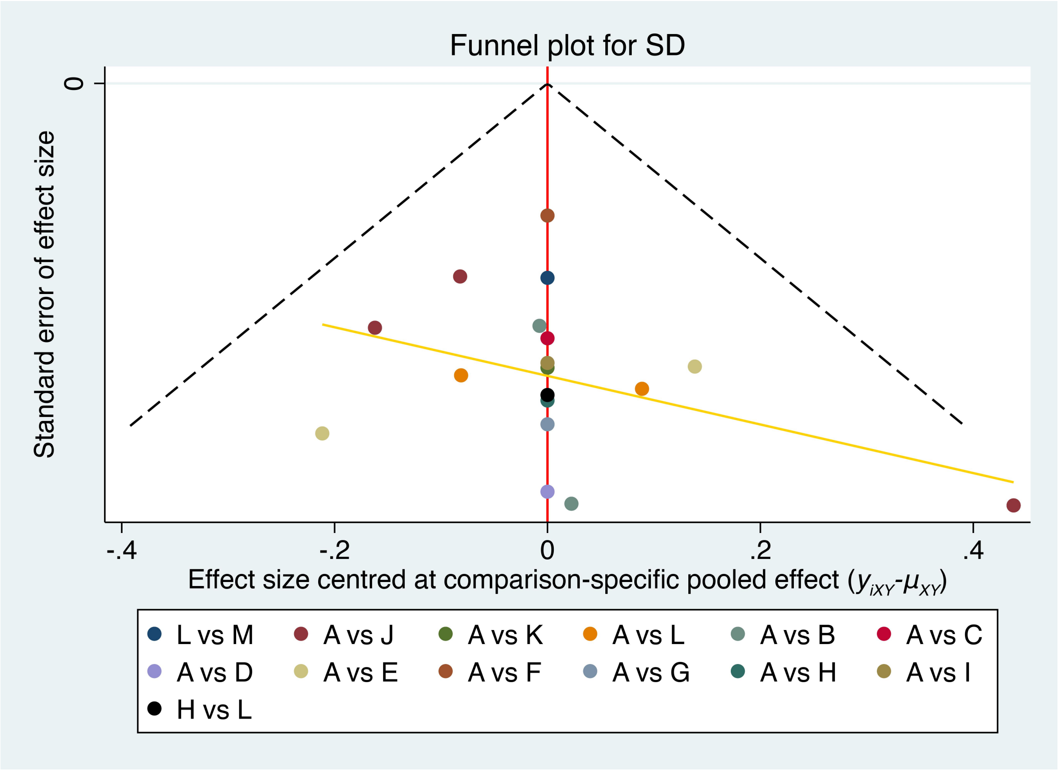

Fig. A15. Funnel plot for SD.

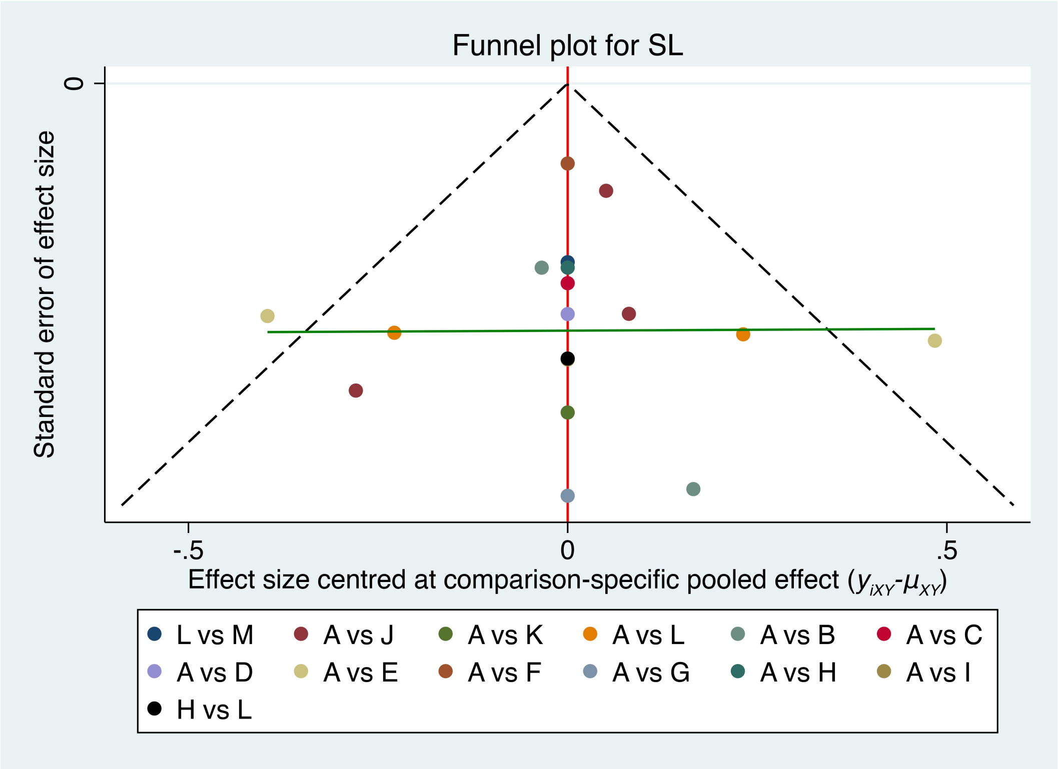

Fig. A16. Funnel plot for SL.

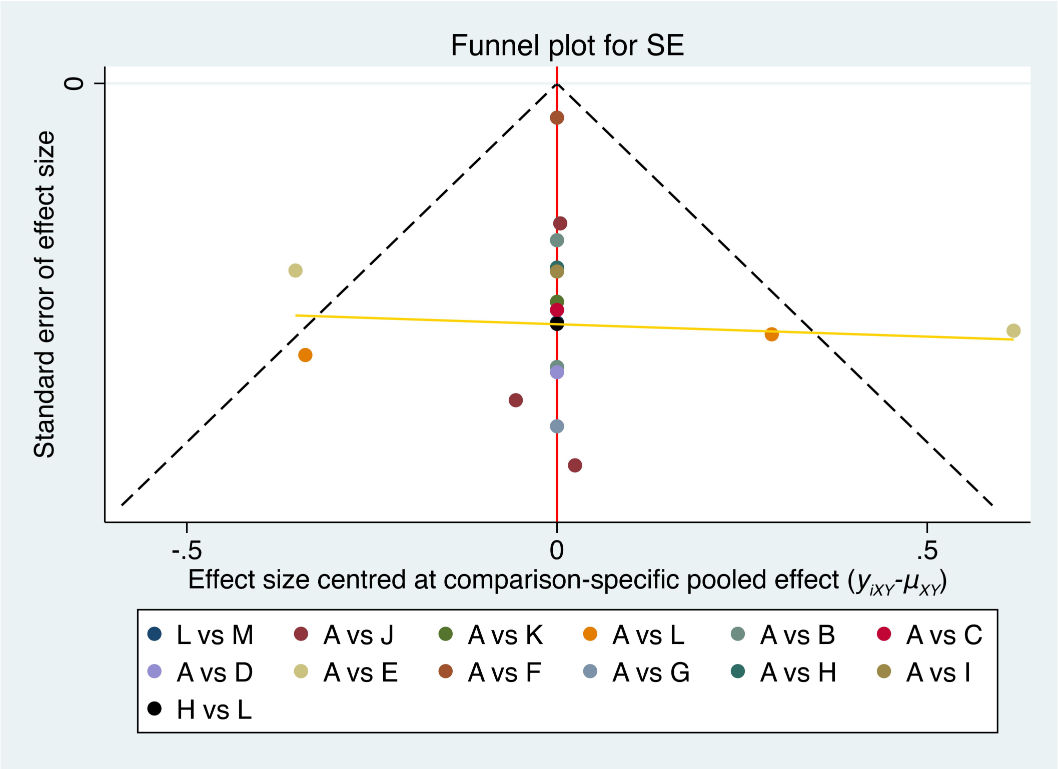

Fig. A17. Funnel plot for SE.

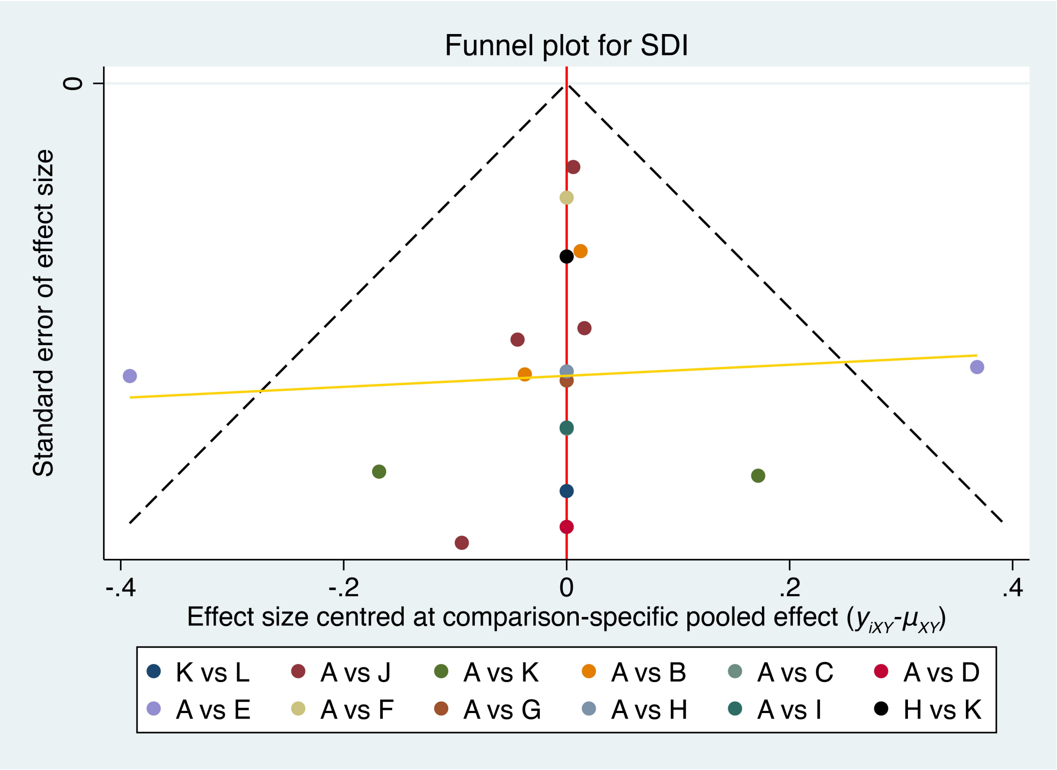

Fig. A18. Funnel plot for SDI.

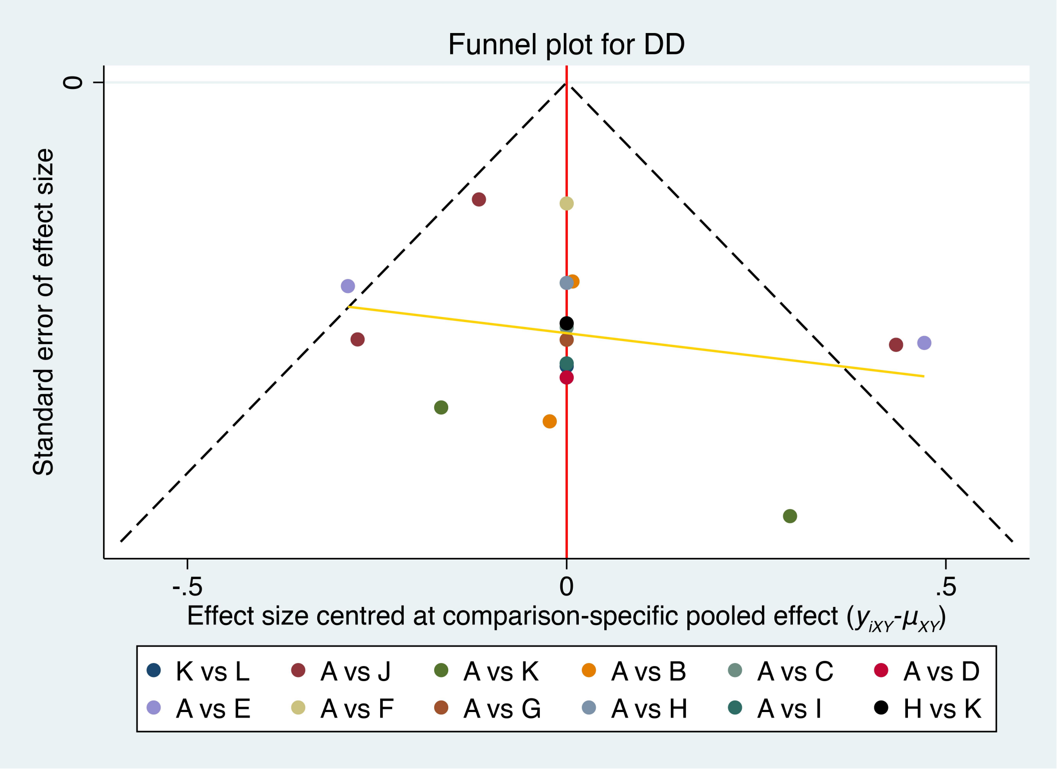

Fig. A19. Funnel plot for DD.

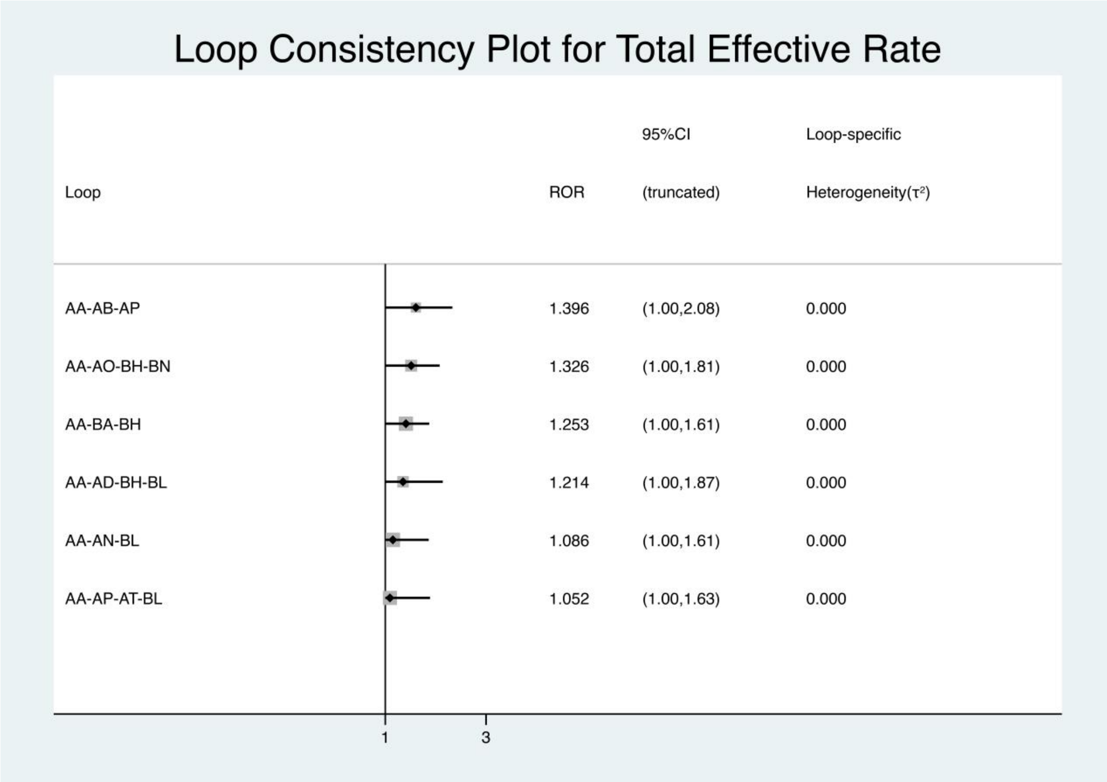

Fig. A20. Loop consistency plot for total effective rate.

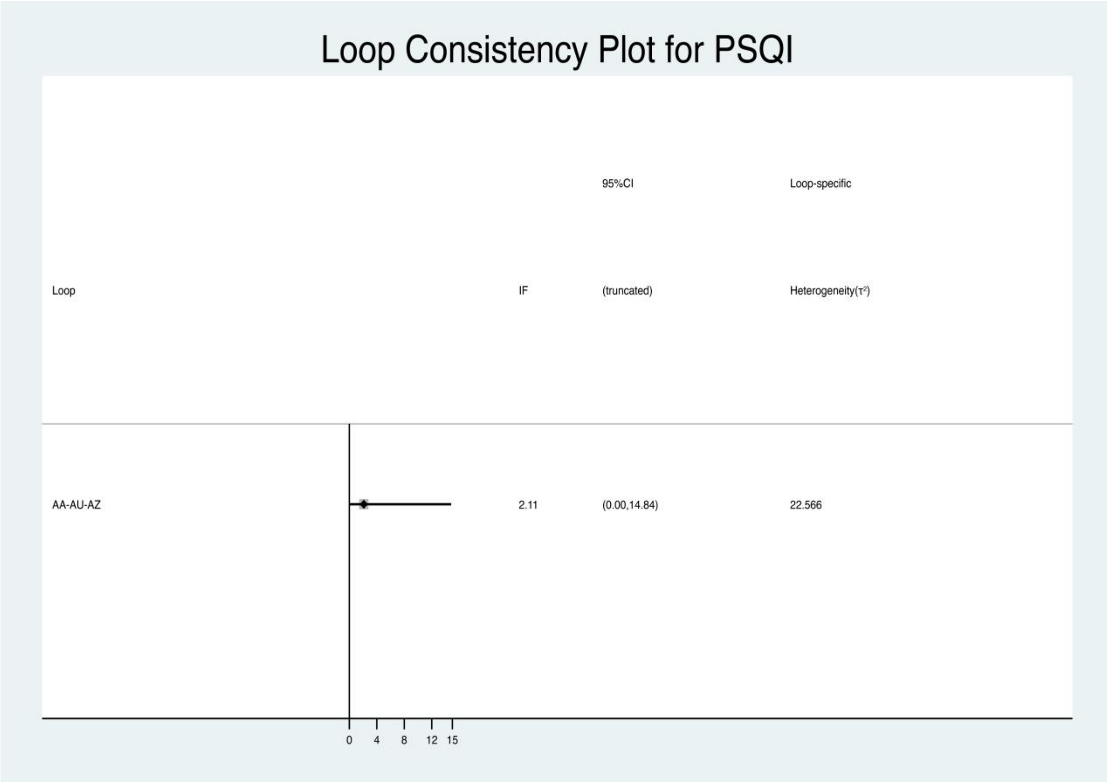

Fig. A21. Loop consistency plot for PSQI.

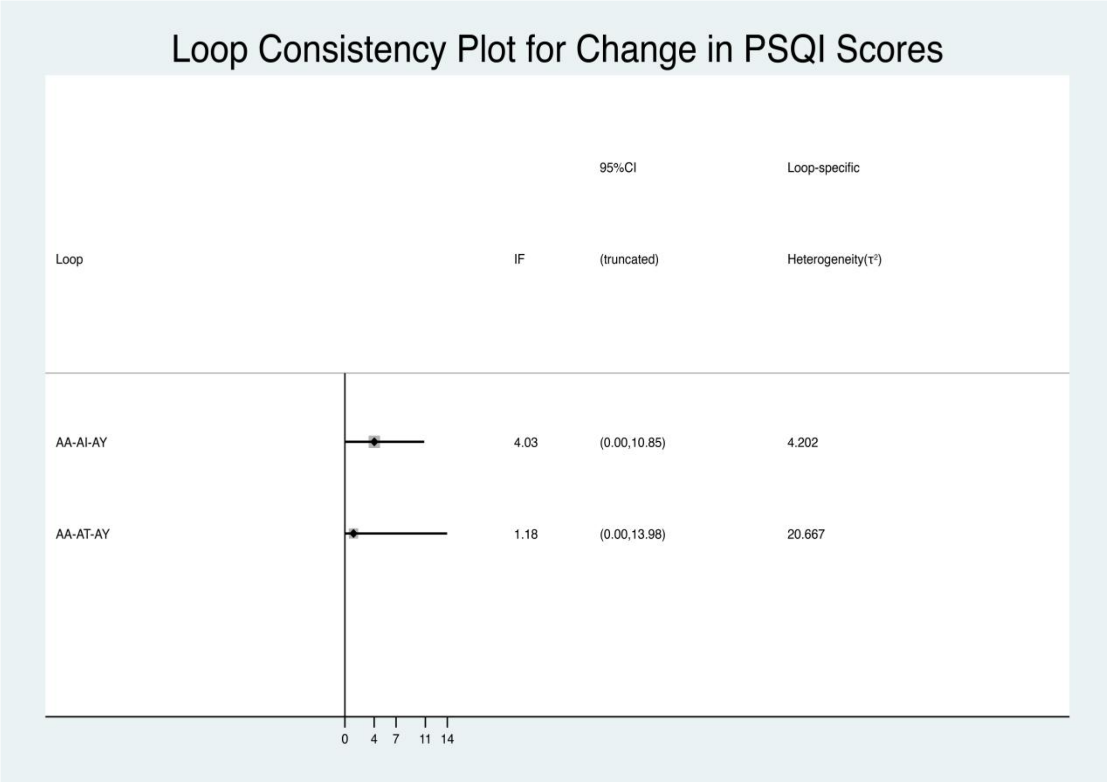

Fig. A22. Loop consistency plot for change in PSQI scores.

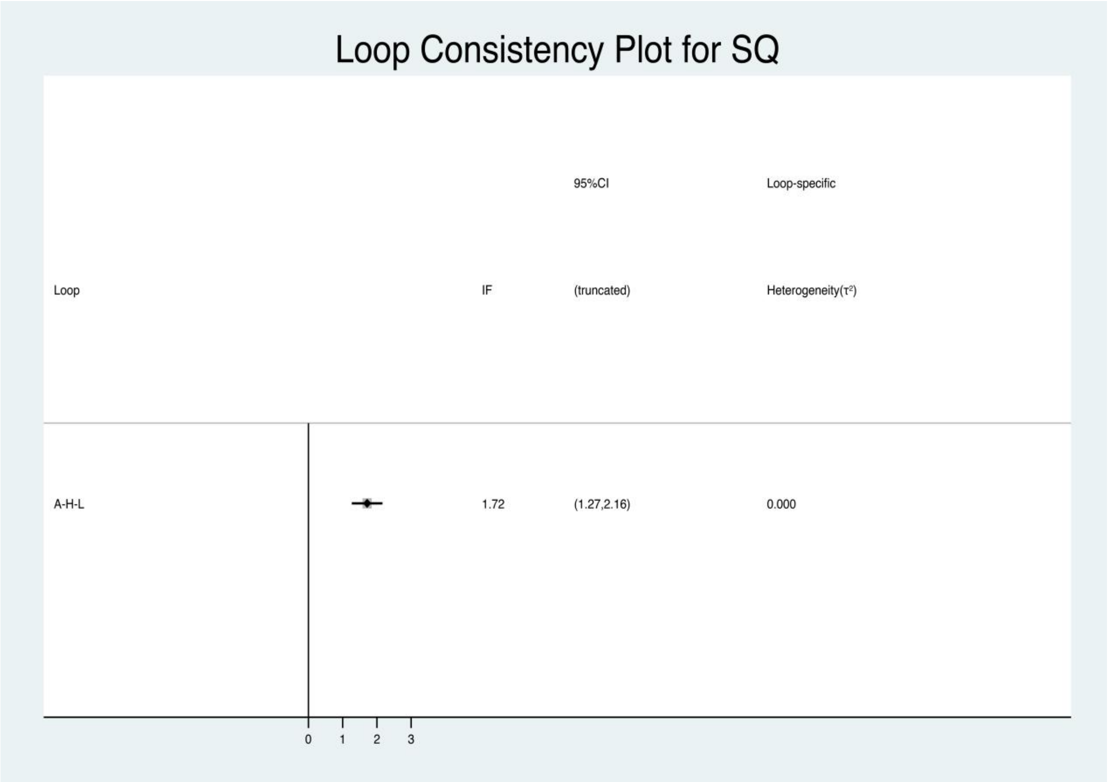

Fig. A23. Loop consistency plot for SQ.

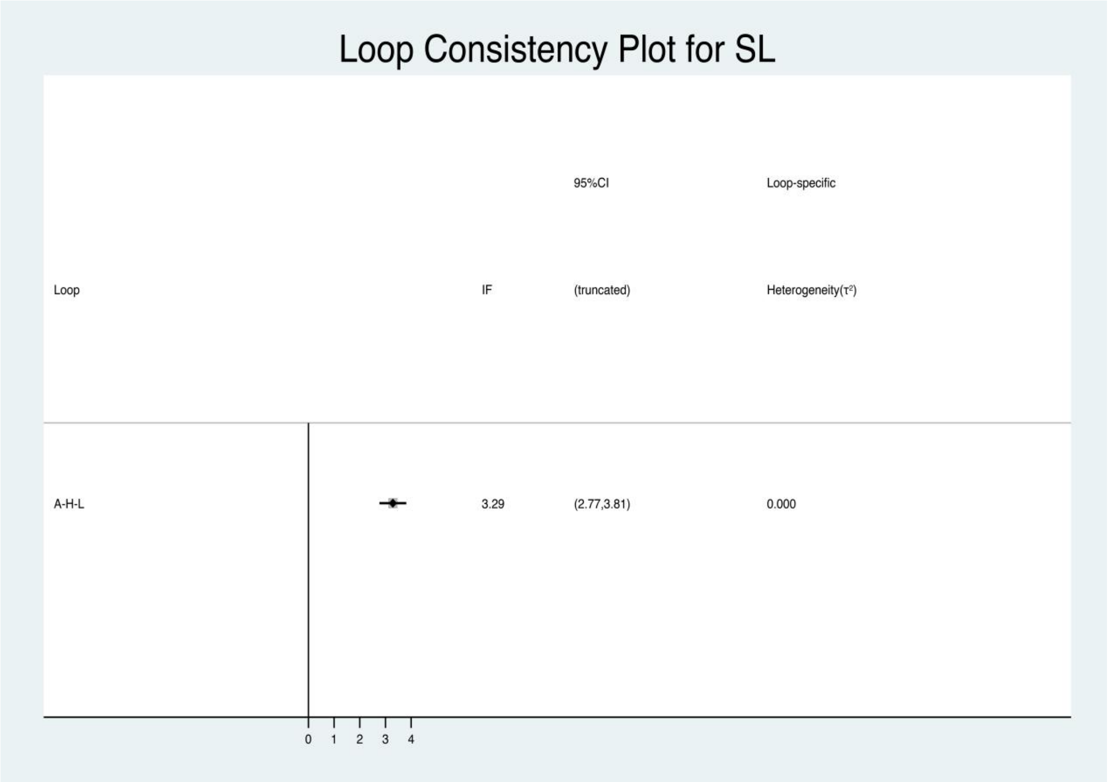

Fig. A24. Loop consistency plot for SL.

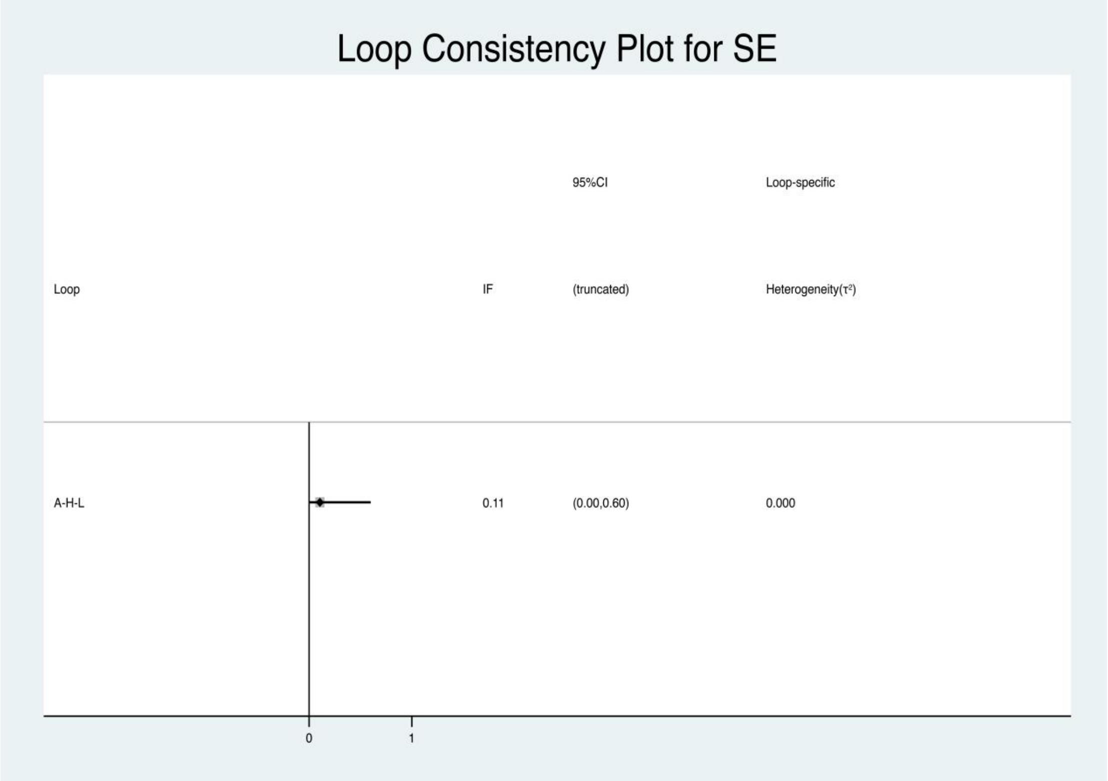

Fig. A25. Loop consistency plot for SE.

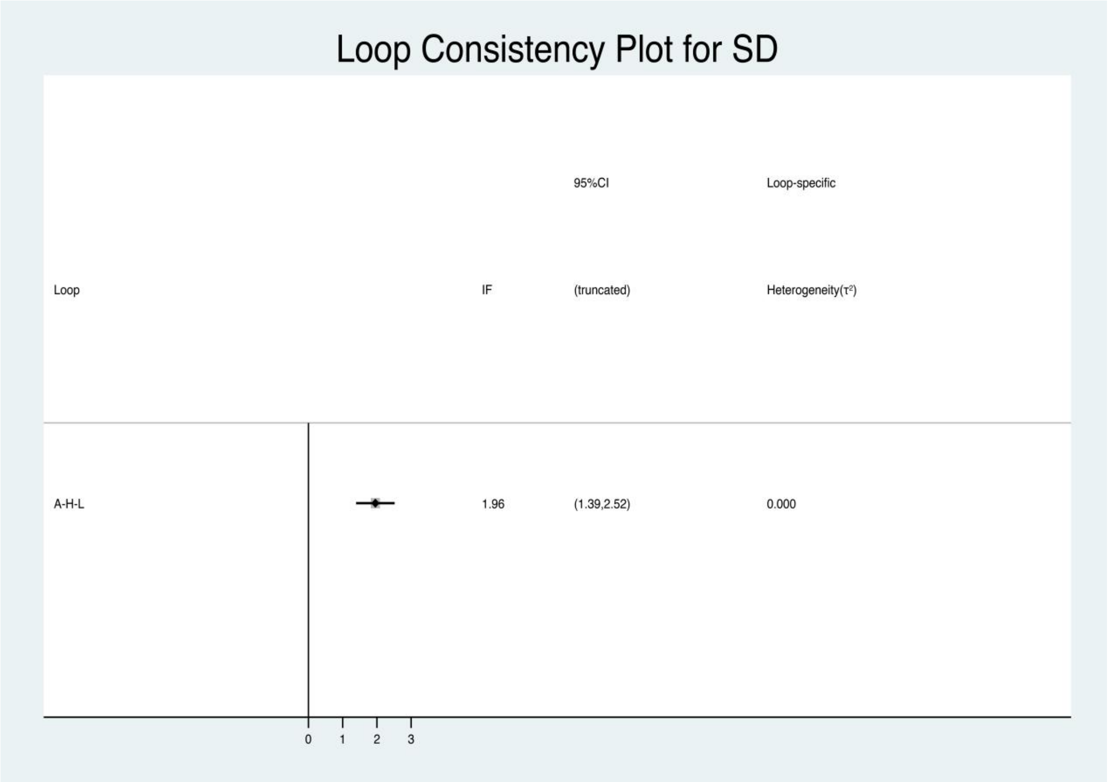

Fig. A26. Loop consistency plot for SD.

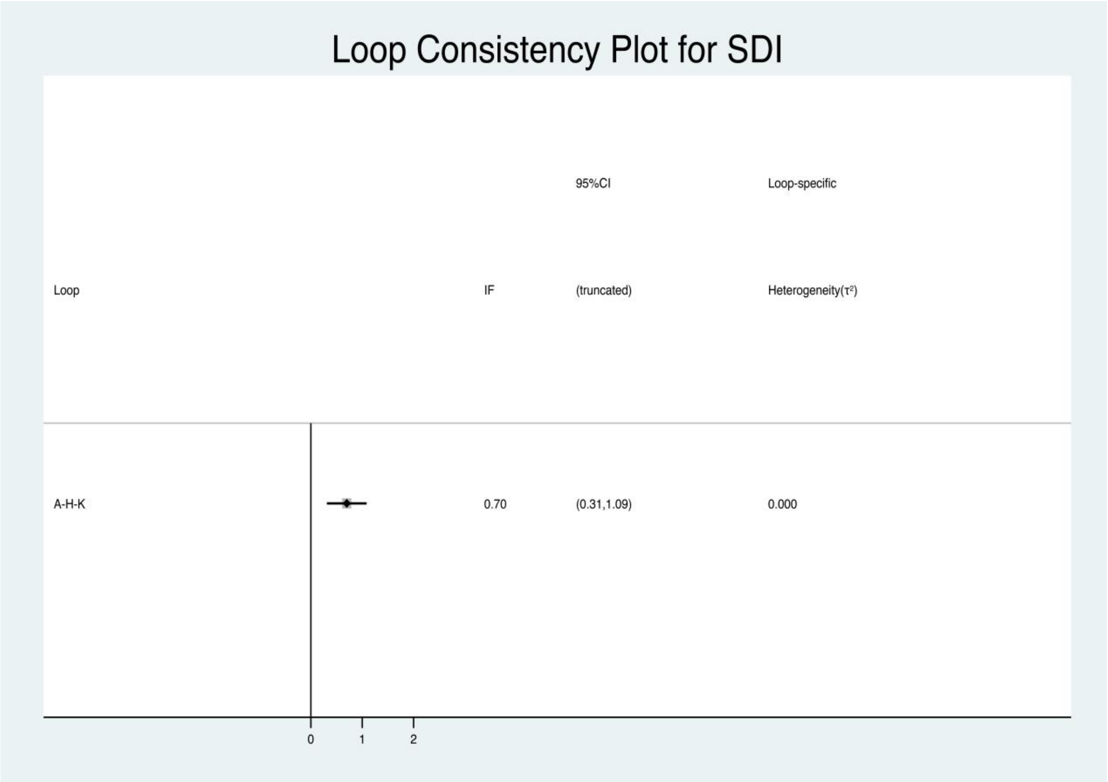

Fig. A27. Loop consistency plot for SDI.

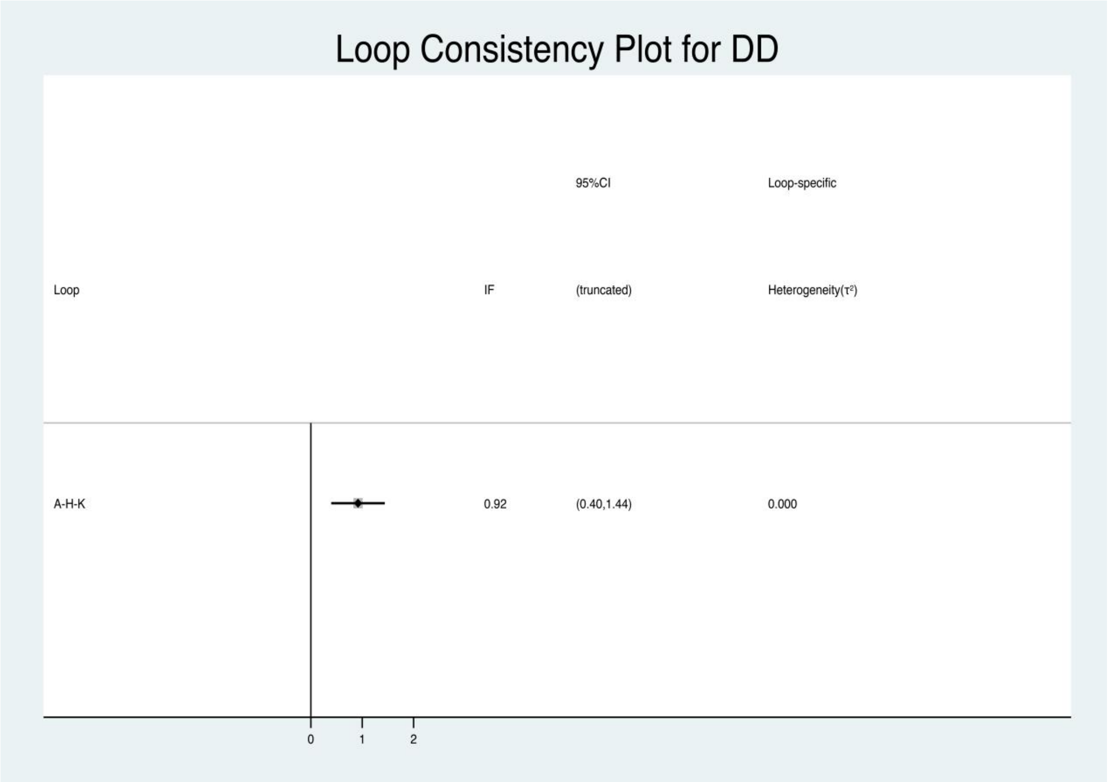

Fig. A28. Loop consistency plot for DD.

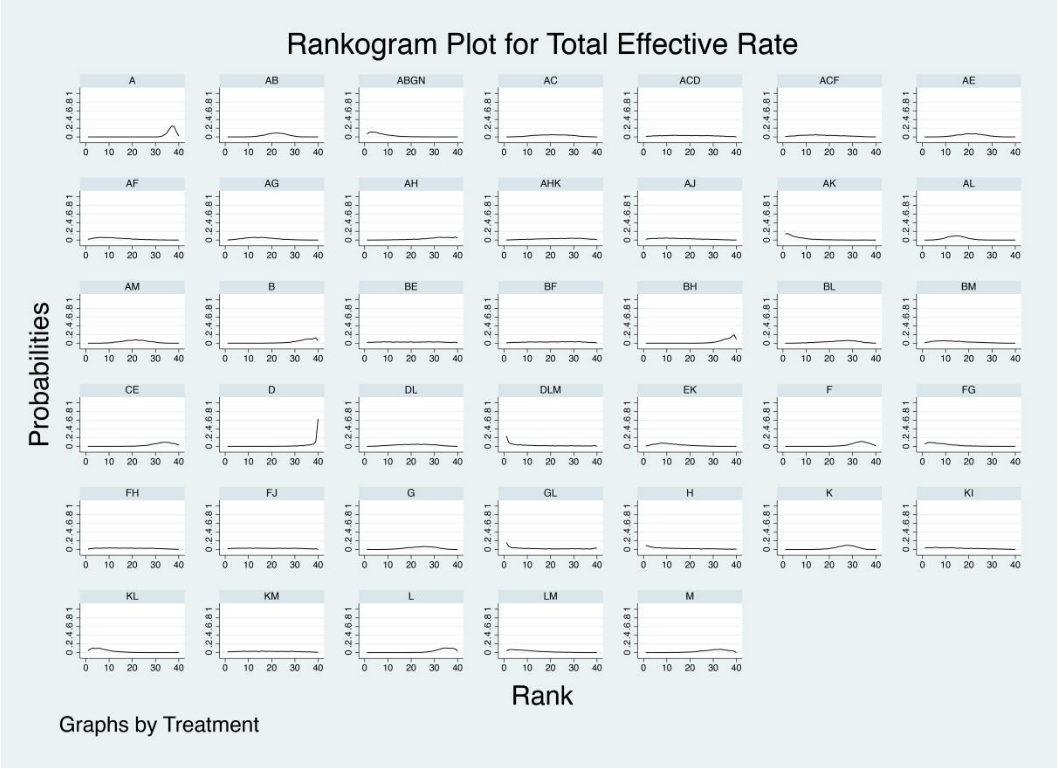

Fig. A29. Rankogram plot for total effective rate.

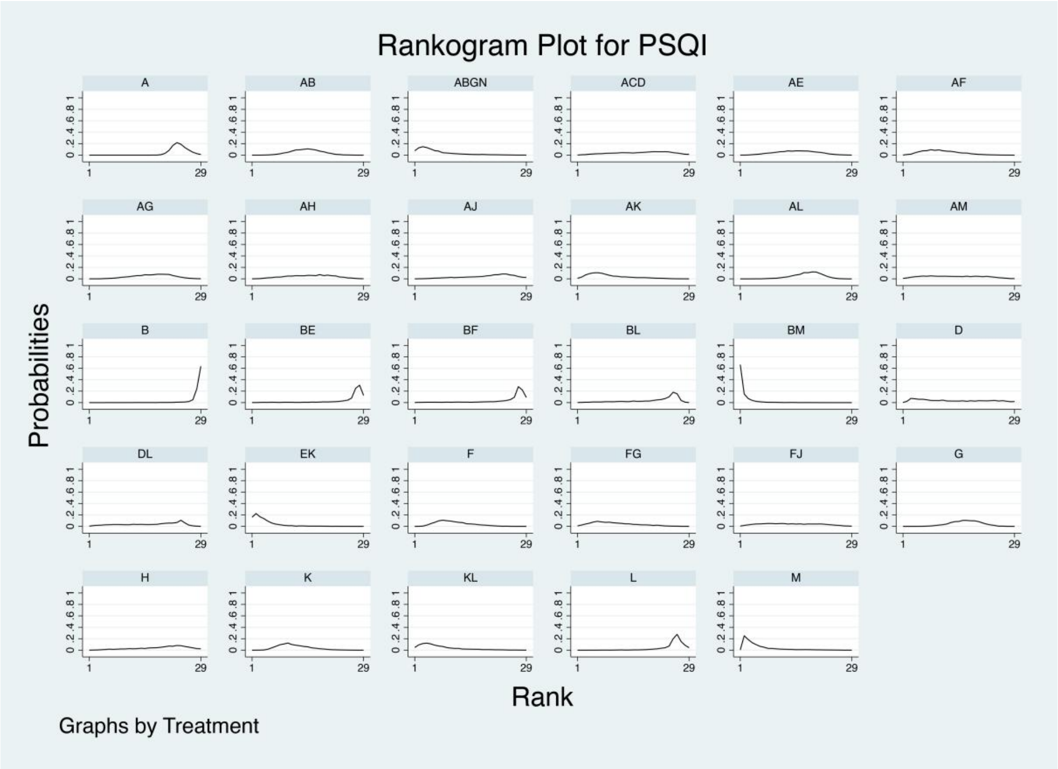

Fig. A30. Rankogram plot for PSQI.

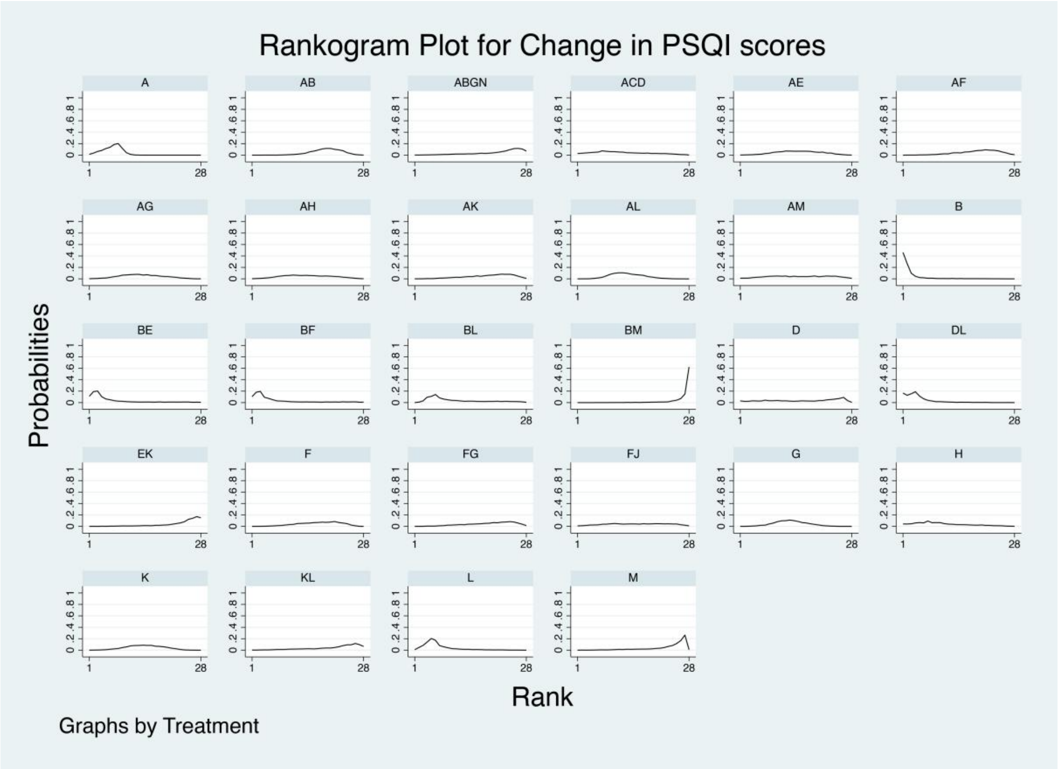

Fig. A31. Rankogram plot for change in PSQI scores.

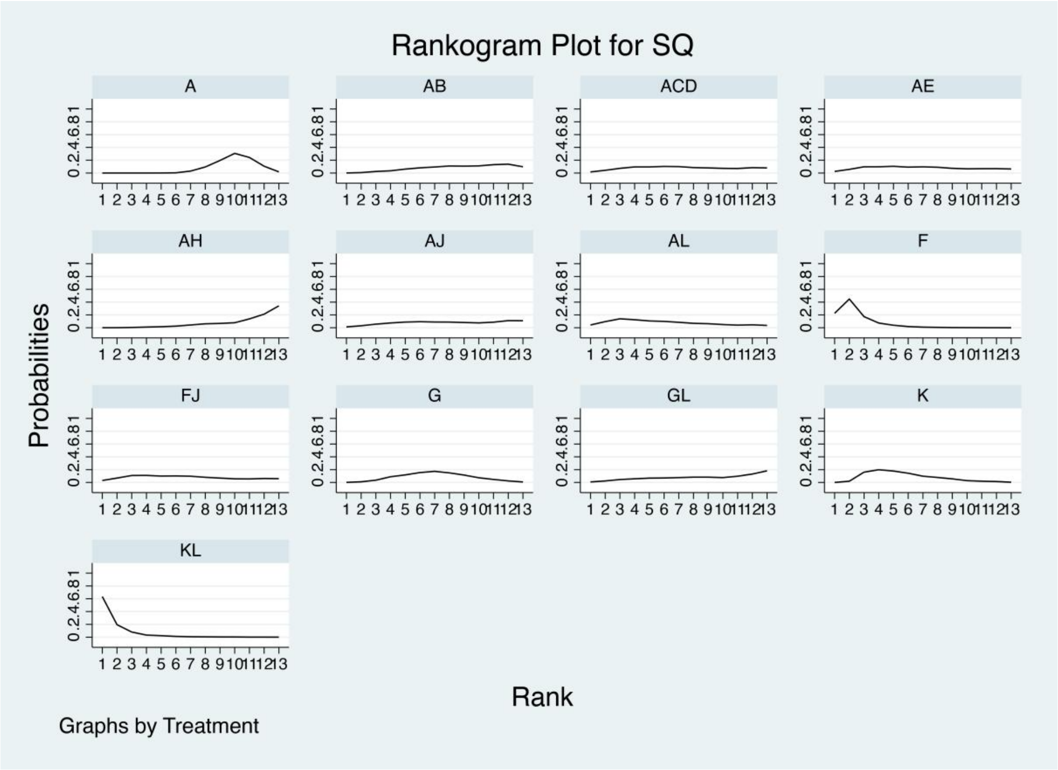

Fig. A32. Rankogram plot for SQ.

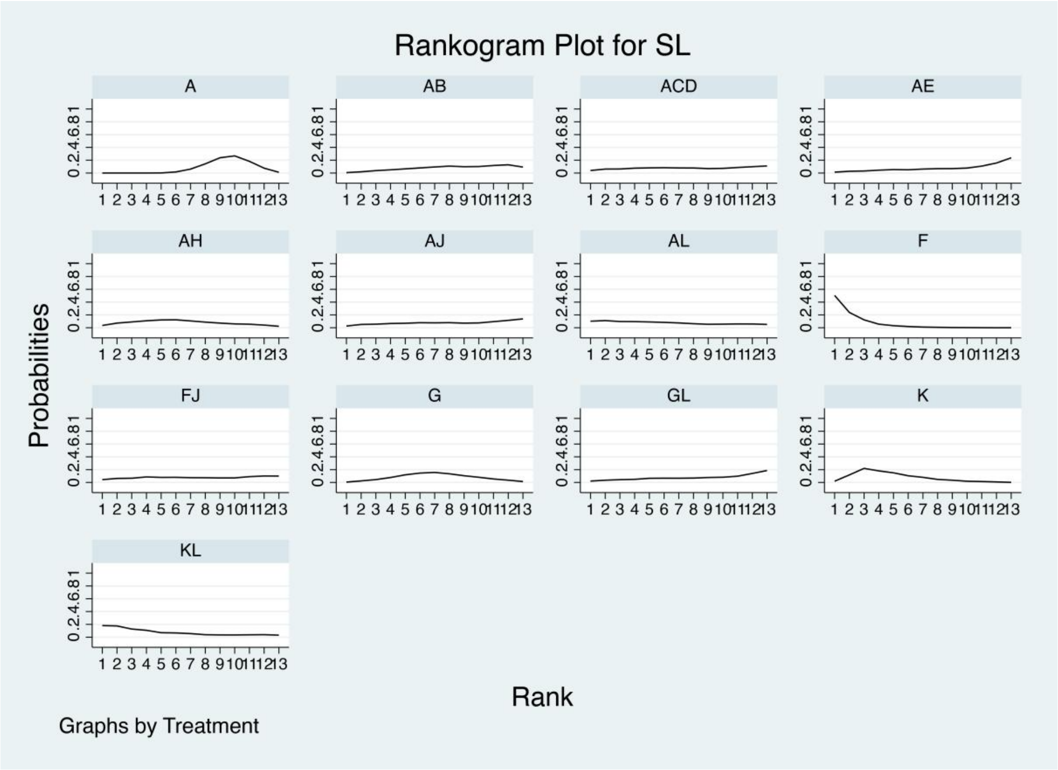

Fig. A33. Rankogram plot for SL.

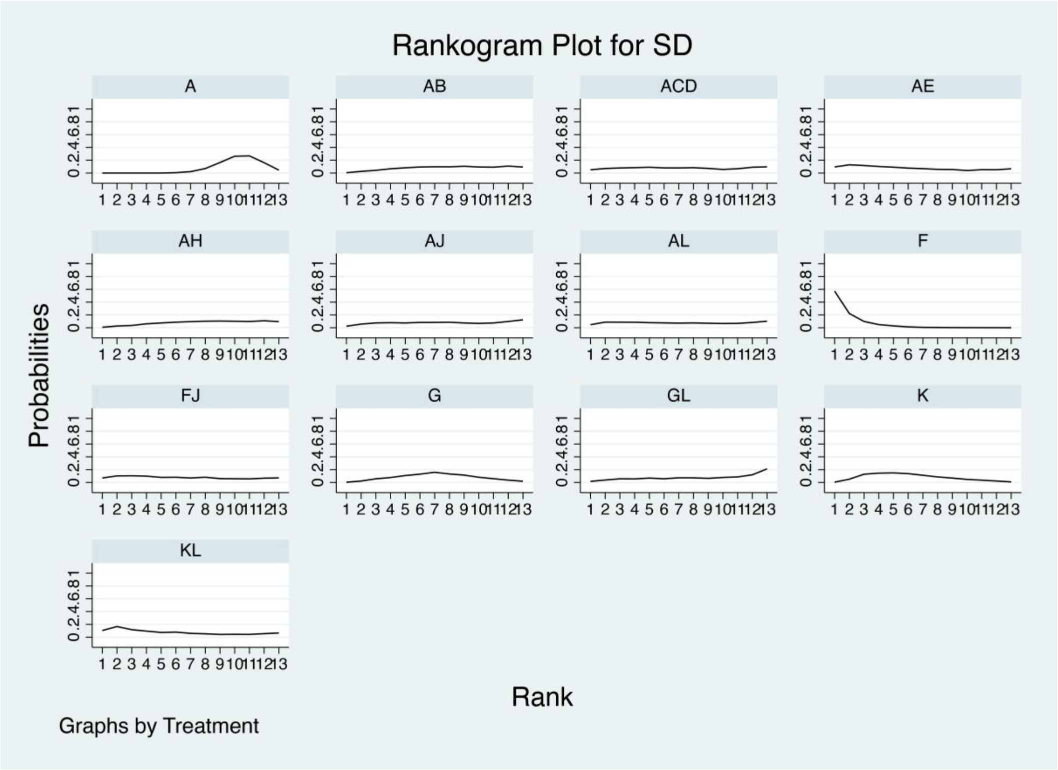

Fig. A34. Rankogramplot for SD.

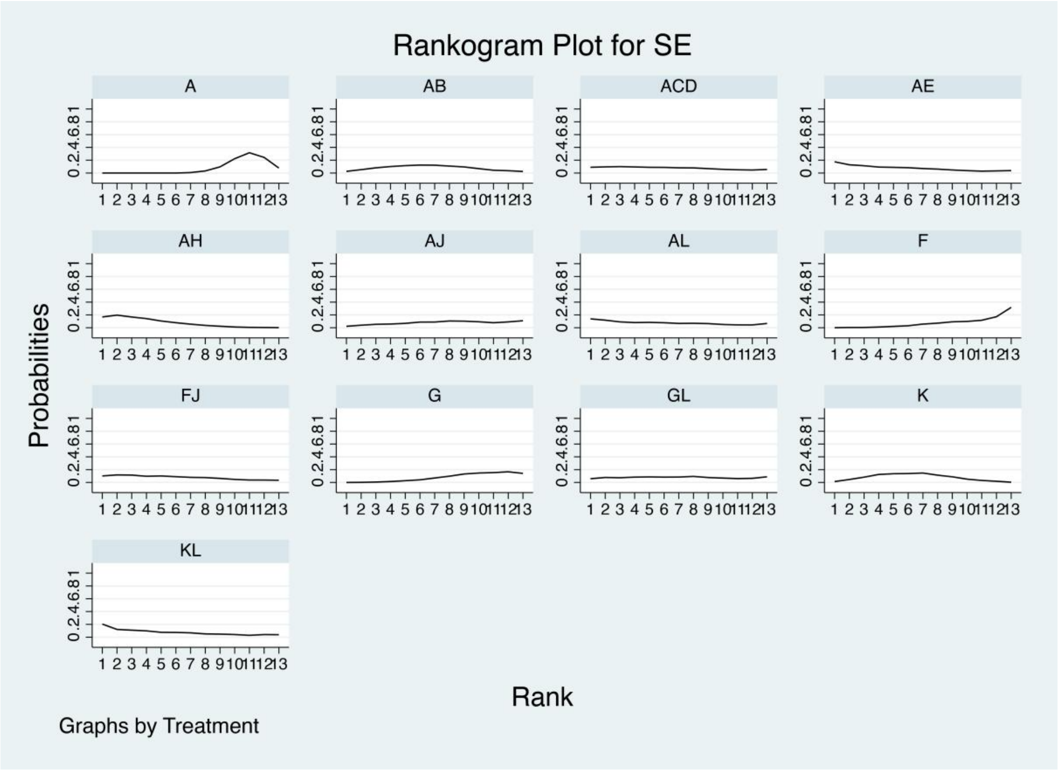

Fig. A35. Rankogram plot for SE.

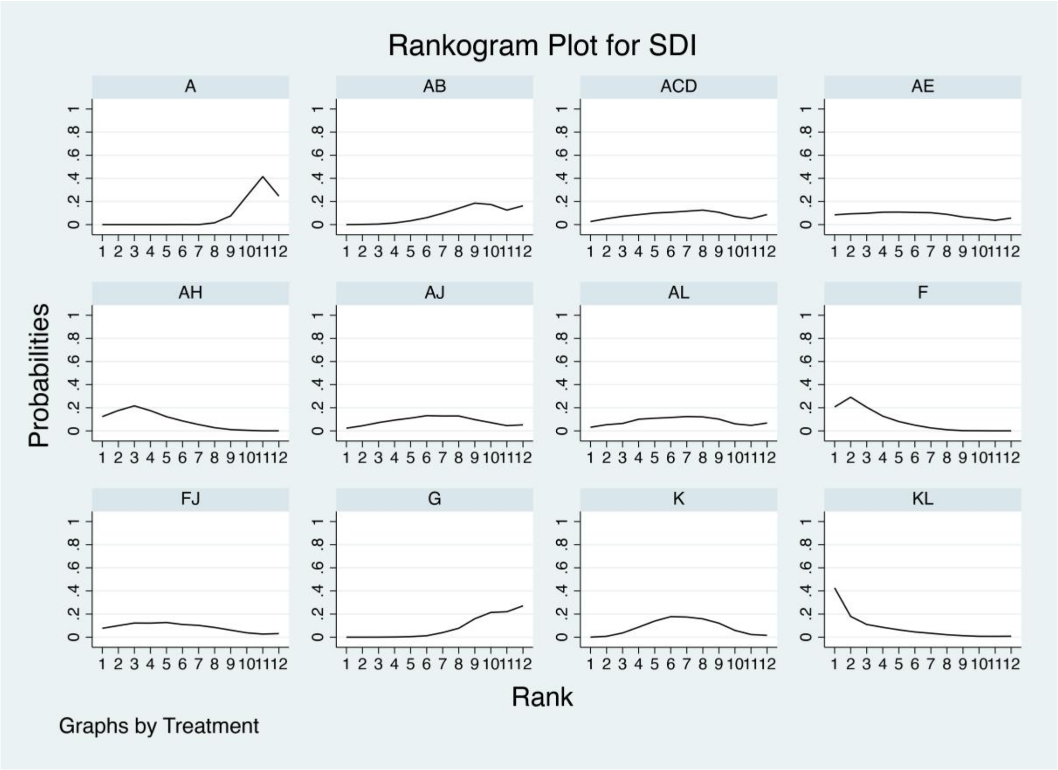

Fig. A36. Rankogram plot for SDI.

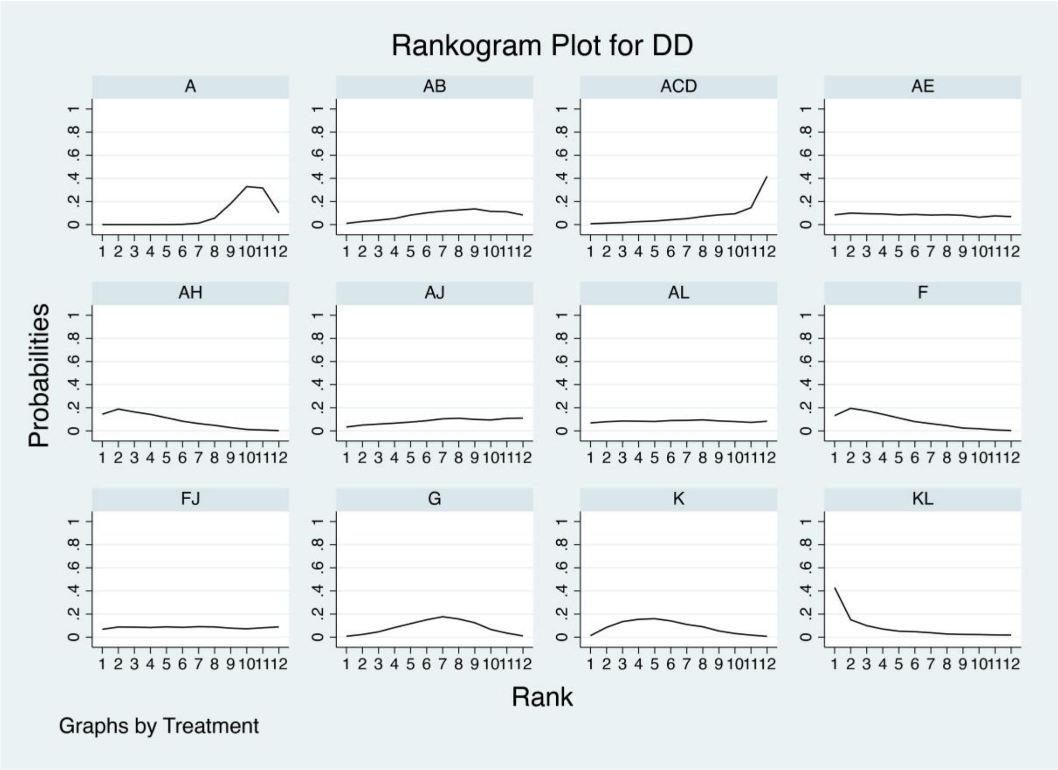

Fig. A37. Rankogram plot for DD.

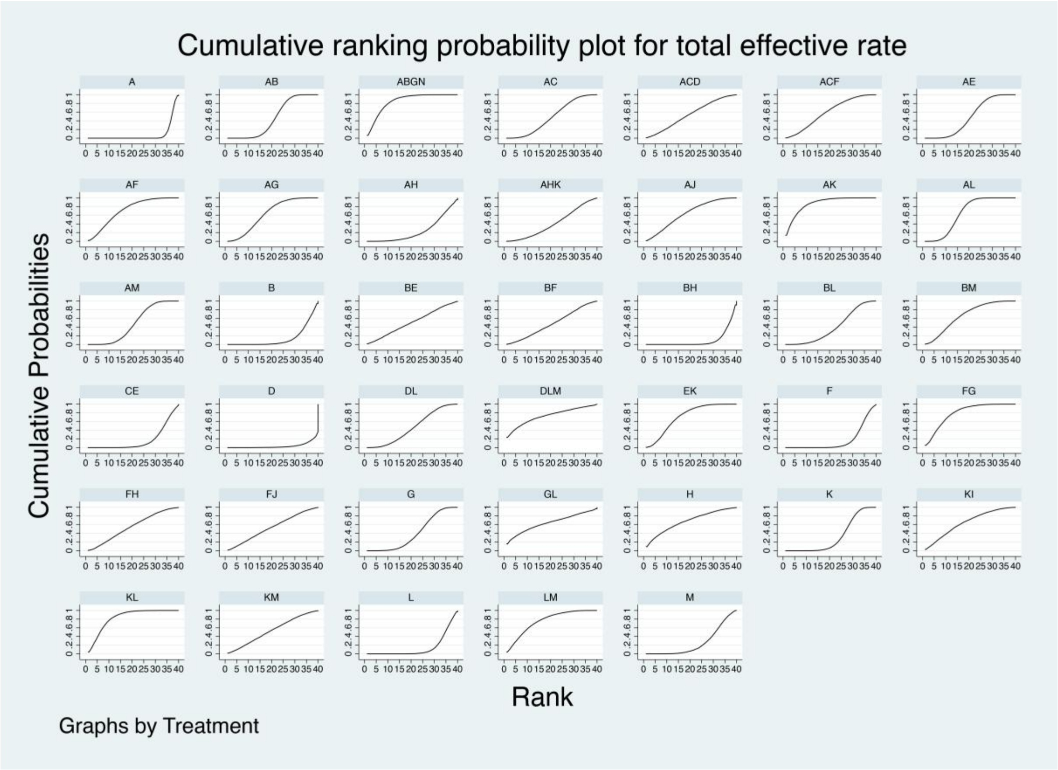

Fig. A38. Cumulative ranking probability plot for total effective rate.

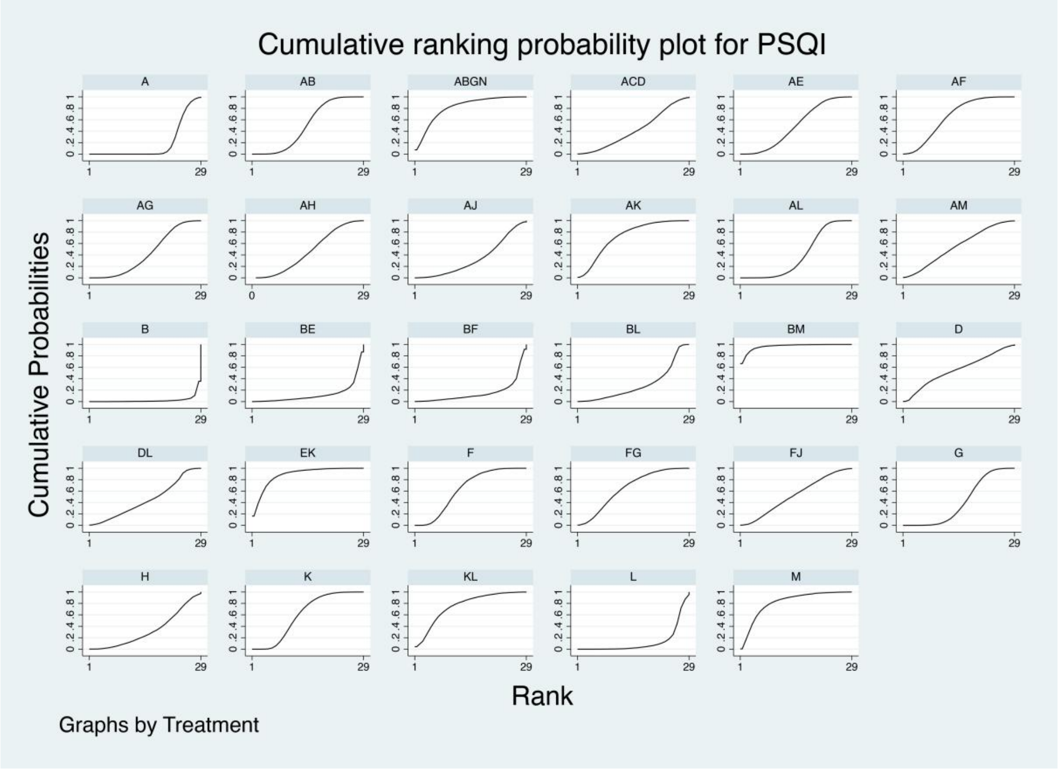

Fig. A39. Cumulative ranking probability plot for PSQI.

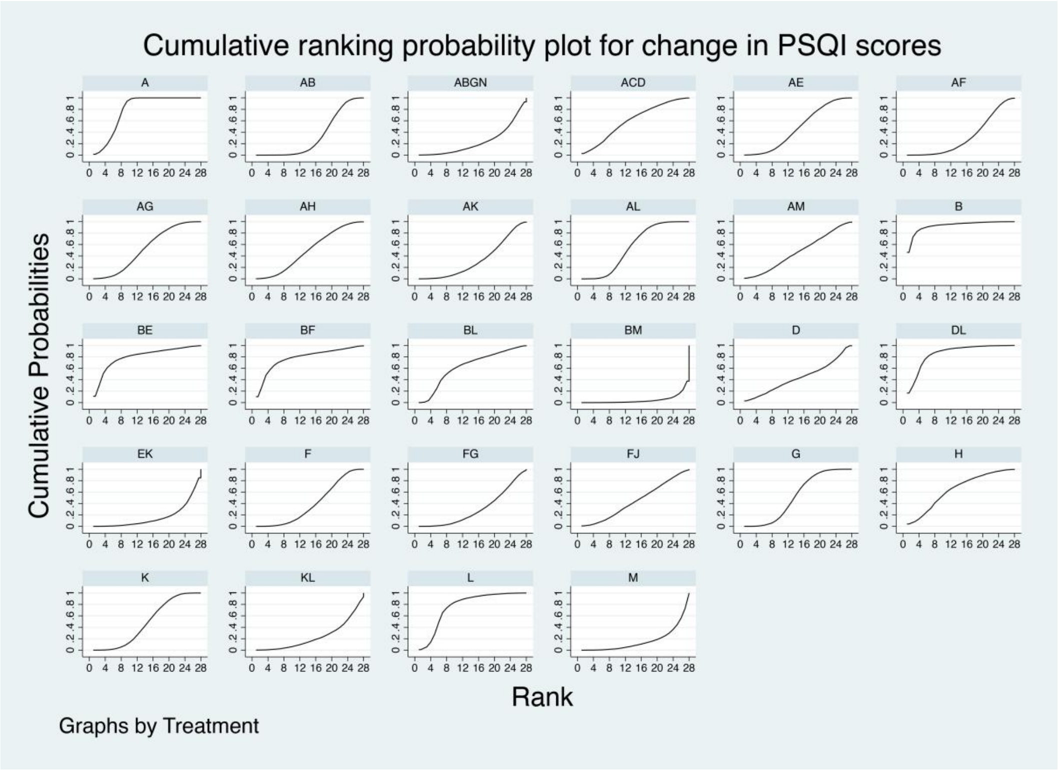

Fig. A40. Cumulative ranking probability plot for change in PSQI scores.

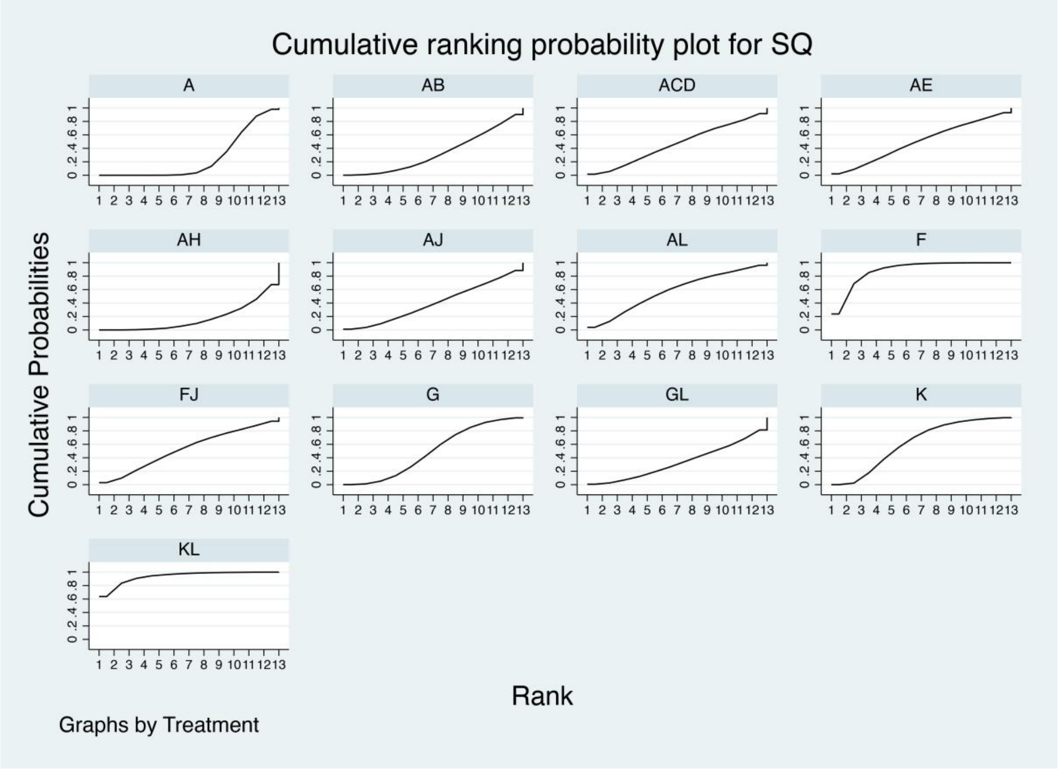

Fig. A41. Cumulative ranking probability plot for SQ.

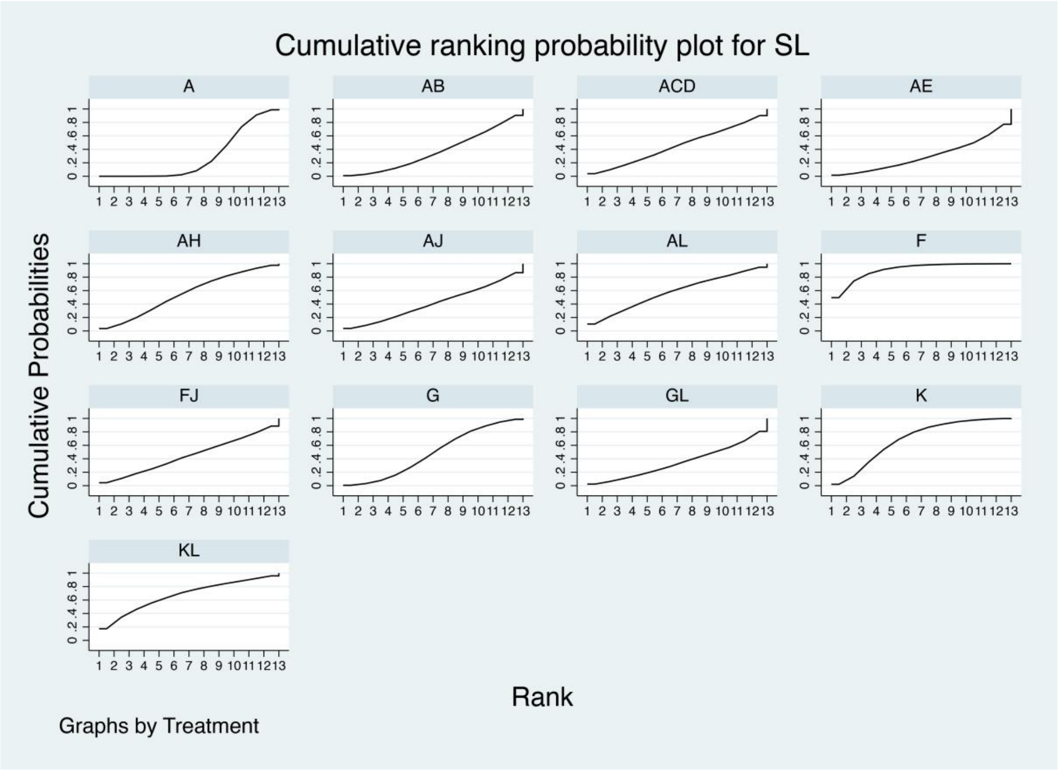

Fig. A42. Cumulative ranking probability plot for SL.

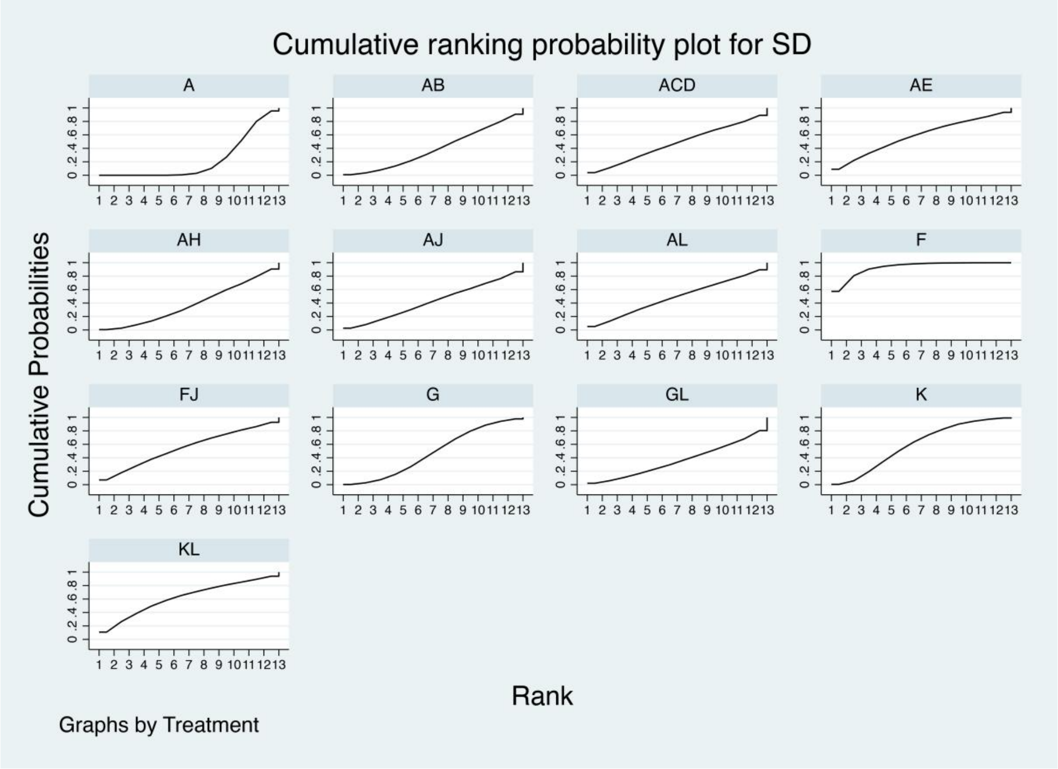

Fig. A43. Cumulative ranking probability plot for SD.

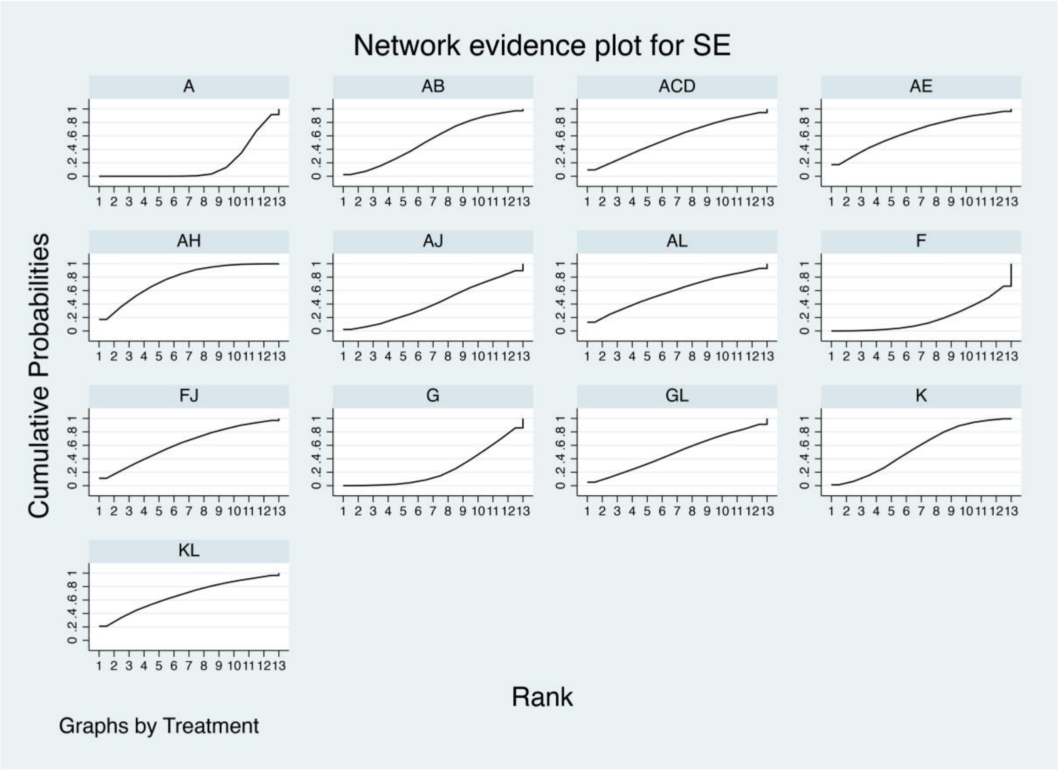

Fig. A44. Cumulative ranking probability plot for SE.

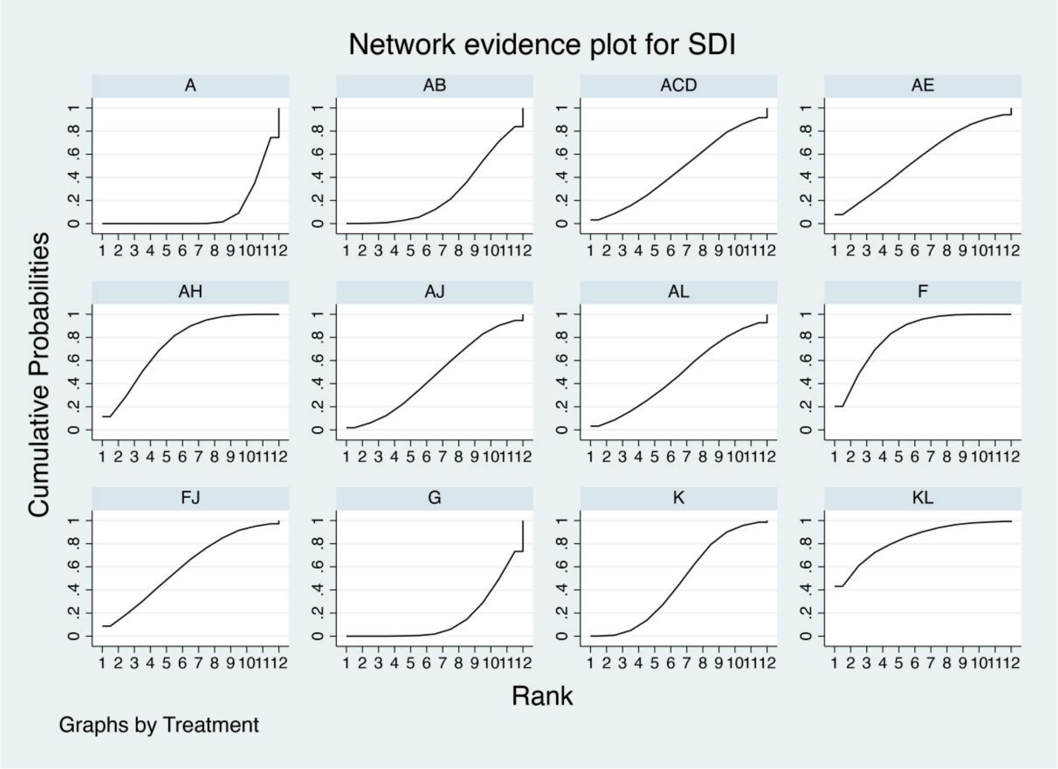

Fig. A45. Cumulative ranking probability plot for SDI.

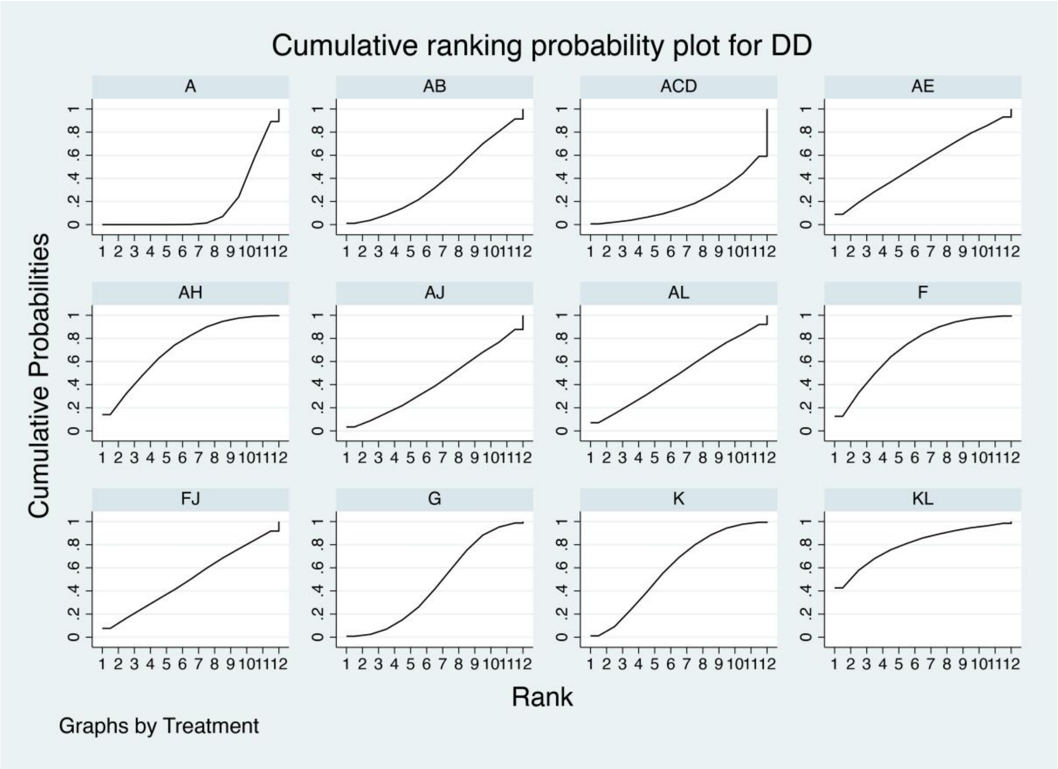

Fig. A46. Cumulative ranking probability plot for DD.

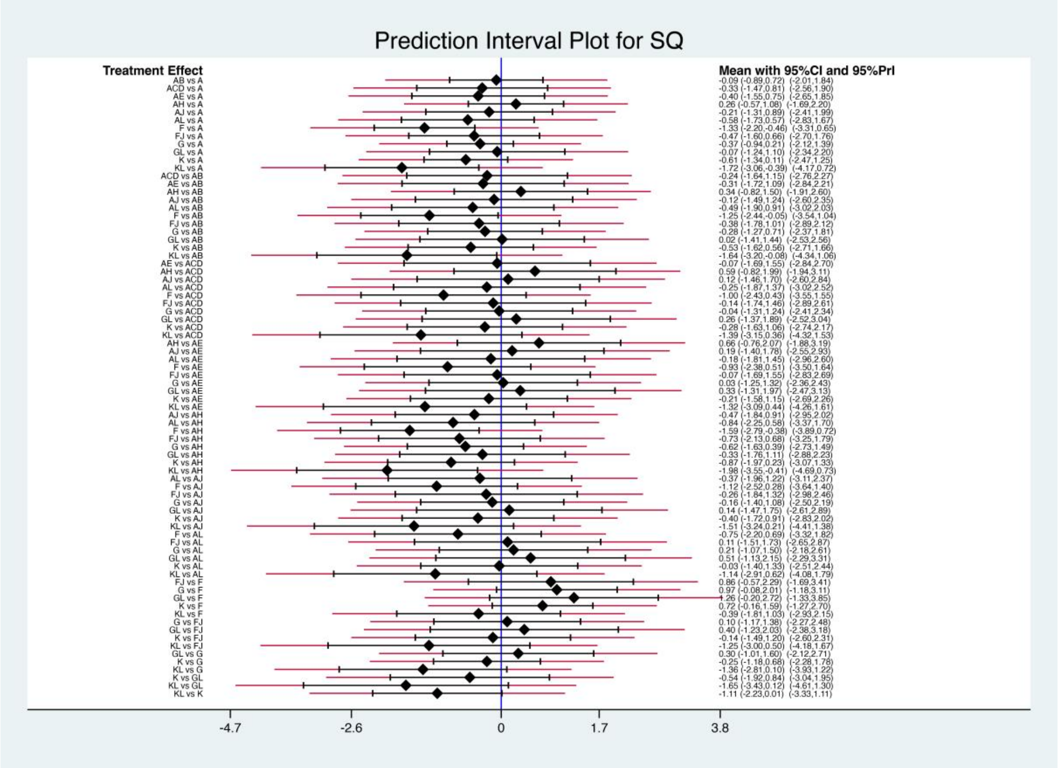

Fig. A47. Prediction interval plot for SQ.

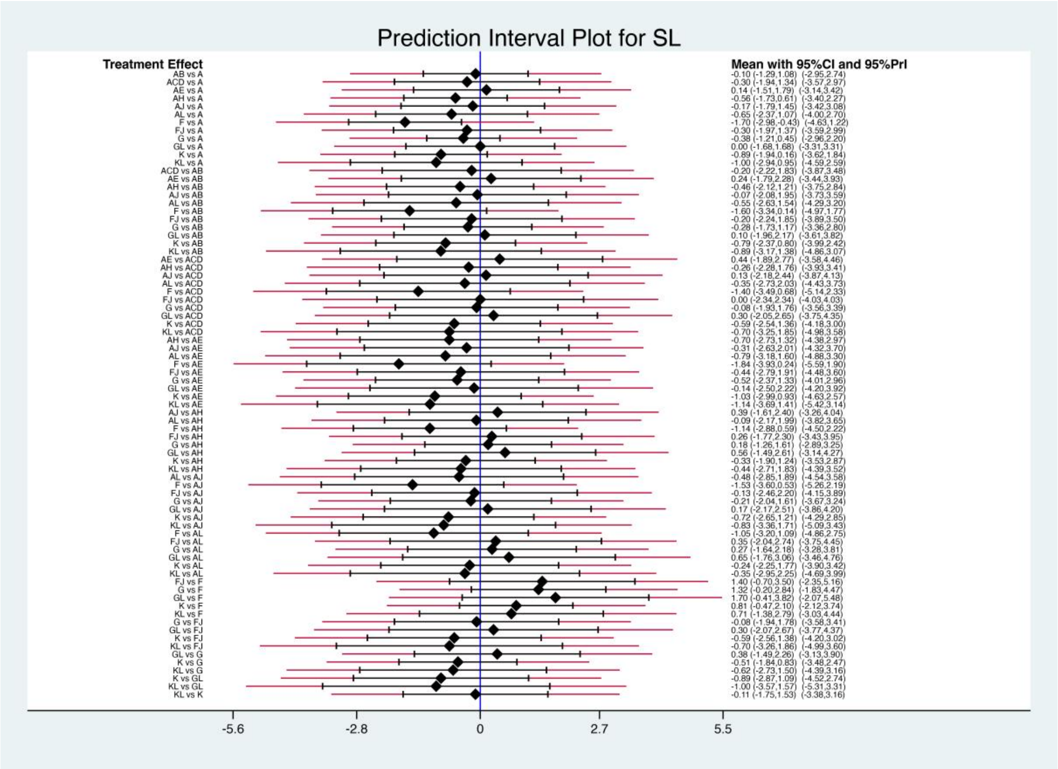

Fig. A48. Prediction interval plot for SL.

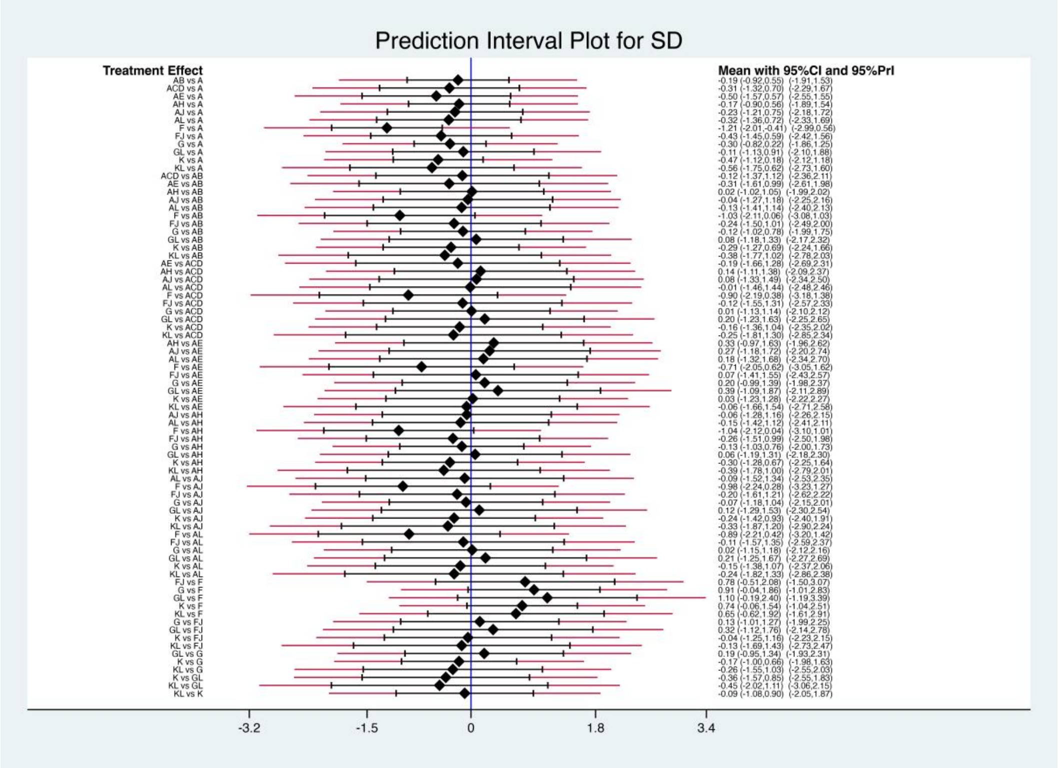

Fig. A49. Prediction interval plot for SD.

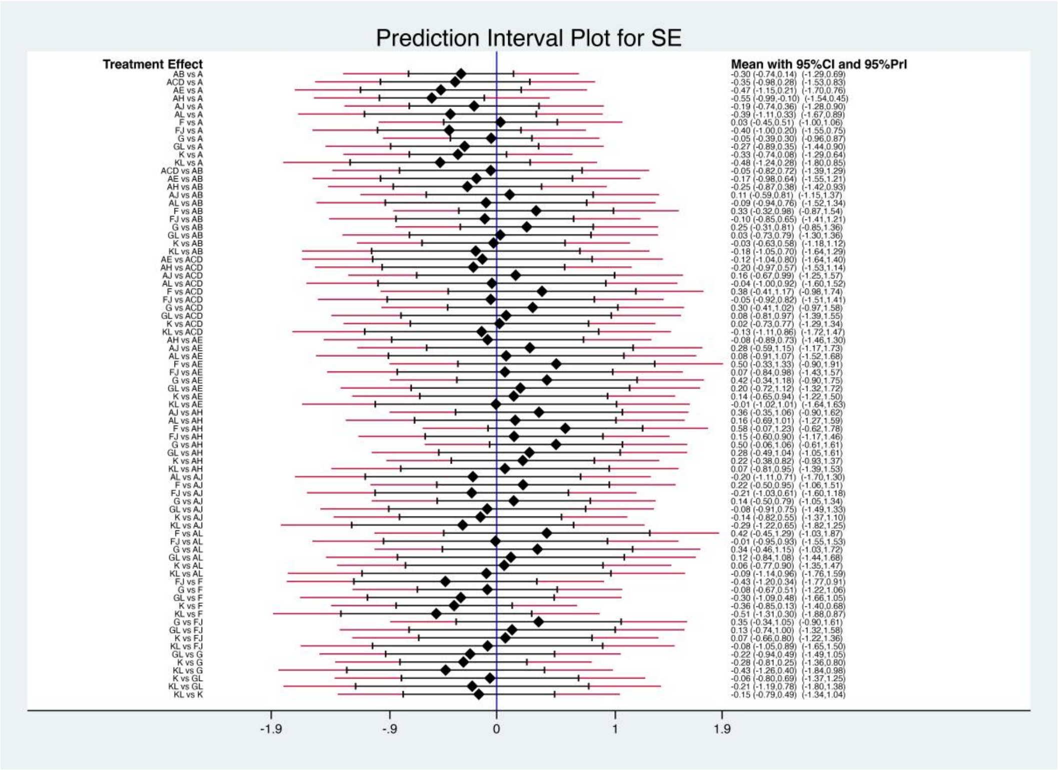

Fig. A50. Prediction interval plot for SE.

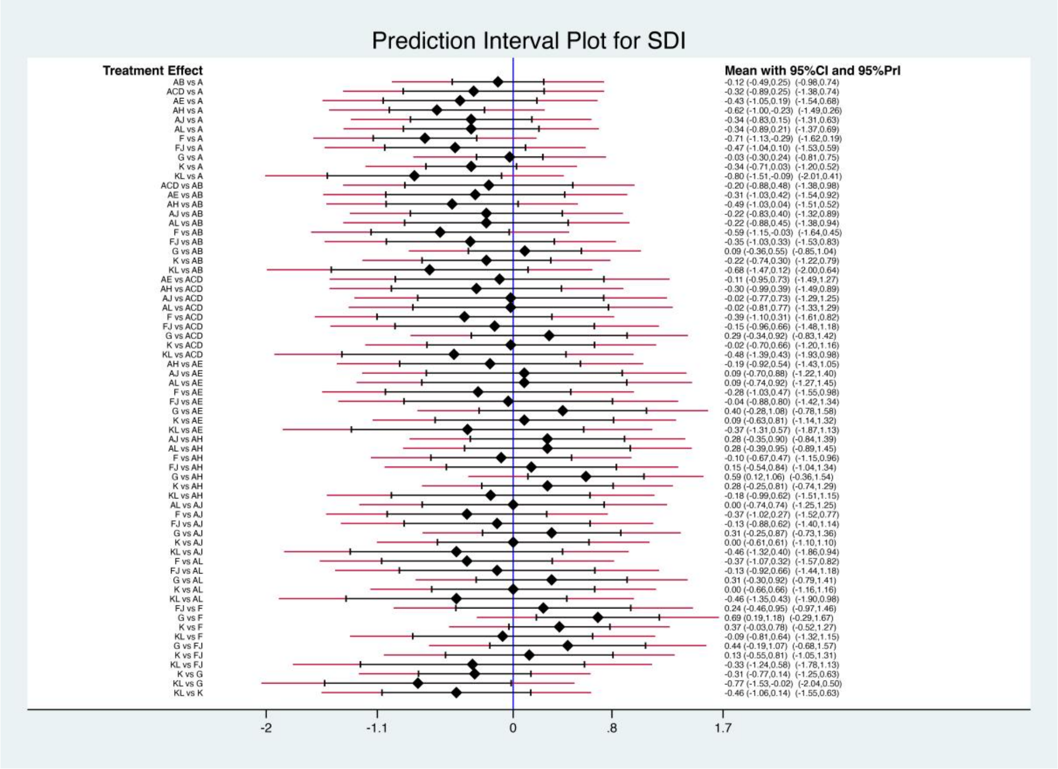

Fig. A51. Prediction interval plot for SDI.

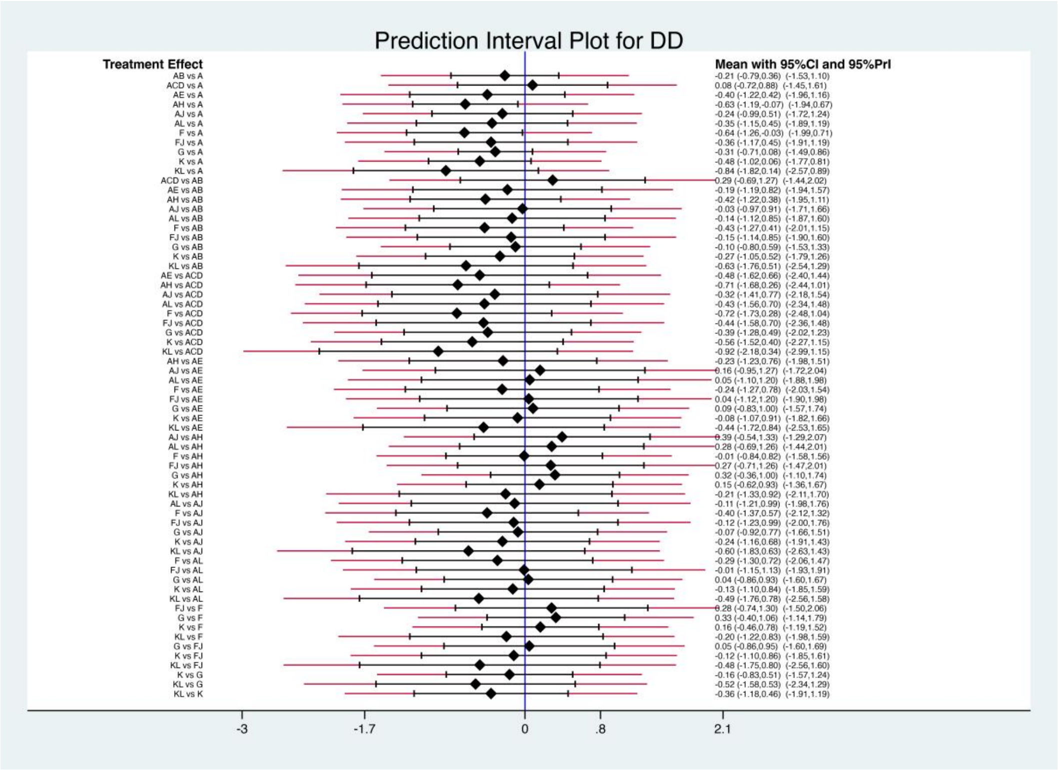

Fig. A52. Prediction interval plot for DD.
